# Structural and functional insights into the *Diabrotica virgifera virgifera* ATP-binding cassette transporter gene family

**DOI:** 10.1186/s12864-019-6218-8

**Published:** 2019-11-27

**Authors:** Folukemi Adedipe, Nathaniel Grubbs, Brad Coates, Brian Wiegmman, Marcé Lorenzen

**Affiliations:** 1 Department of Entomology and Plant Pathology, North Carolina State University, Box 7613, 1566 Thomas Hall, Raleigh, NC 27695-7613 USA; 2 USDA-ARS, Corn Insects & Crop Genetics Research Unit, Ames, IA 50011 USA

**Keywords:** ATP-binding cassette (ABC) transporter, Phylogenetic, Transcriptome, RNA interference (RNAi), Corn rootworm

## Abstract

**Background:**

The western corn rootworm, *Diabrotica virgifera virgifera*, is a pervasive pest of maize in North America and Europe, which has adapted to current pest management strategies. In advance of an assembled and annotated *D. v. virgifera* genome, we developed transcriptomic resources to use in identifying candidate genes likely to be involved in the evolution of resistance, starting with members of the ATP-binding cassette (ABC) transporter family.

**Results:**

In this study, 65 putative *D. v. virgifera* ABC (*Dvv*ABC) transporters were identified within a combined transcriptome assembly generated from embryonic, larval, adult male, and adult female RNA-sequence libraries. Phylogenetic analysis placed the deduced amino-acid sequences of the *Dvv*ABC transporters into eight subfamilies (A to H). To supplement our sequence data with functional analysis, we identified orthologs of *Tribolium castaneum* ABC genes which had previously been shown to exhibit overt RNA interference (RNAi) phenotypes. We identified eight such *D. v. virgifera* genes, and found that they were functionally similar to their *T. castaneum* counterparts. Interestingly, depletion of *DvvABCB_39715* and *DvvABCG_3712* transcripts in adult females produced detrimental reproductive and developmental phenotypes, demonstrating the potential of these genes as targets for RNAi-mediated insect control tactics.

**Conclusions:**

By combining sequence data from four libraries covering three distinct life stages, we have produced a relatively comprehensive de novo transcriptome assembly for *D. v. virgifera*. Moreover, we have identified 65 members of the ABC transporter family and provided the first insights into the developmental and physiological roles of ABC transporters in this pest species.

## Background

The western corn rootworm, *Diabrotica virgifera virgifera* (Coleoptera: Chrysomelidae), is a major pest of maize in Europe and North America [1–3], where costs of management, as well as crop losses attributed to damage by this pest, are estimated at over 1 billion U.S. dollars annually in North America alone (reviewed in [4]). The notorious difficulty facing efforts to control *D. v. virgifera* feeding on maize has arisen via intra-species adaptations that overcome various pest management methods [5]. For example, changes in oviposition preference within “soybean variant” populations of *D. v. virgifera* in the Midwest United States circumvent the cultural-control practice of corn-soybean rotation [3, 5–7]. Additionally, adapted phenotypes within North American *D. v. virgifera* populations can survive high exposures to organochlorine [8], pyrethroid [9], and carbamate and organophosphate insecticides [10]. In some instances resistant phenotypes have persisted for decades despite the removal of selection pressures [11]. More recently, field populations of *D. v. virgifera* have developed high levels of resistance to transgenic maize hybrids that express *Bacillus thuringiensis* (Bt) crystal toxins Cry3Bb1 [12], mCry3A [13], Cry3.1Ab [14], and Cry34/35Ab1 [14–17]. However, RNA interference (RNAi) shows great potential as a novel insect pest control technology [18], especially in instances where target species are sensitive to oral RNAi [19]. *D. v. virgifera* is highly sensitive to oral RNAi [20–22], suggesting that it could suppress feeding damage caused by this pest [23].

ATP-binding cassette (ABC) proteins comprise one of the largest gene families, and are found across prokaryotic and eukaryotic domains [24]. Most of these proteins function as transmembrane transporters, which actively move a myriad of molecules across cellular membranes [25]. ABC transporter proteins have a two-domain structure: a highly conserved nucleotide-binding domain (NBD) and a variable transmembrane domain (TMD) [26]. The NBD binds and hydrolyzes ATP to provide the energy required for translocating a substrate across cell membranes, while the TMD forms a channel through which the substrate is transported [27]. Each NBD possesses several highly conserved, characteristic motifs, including Walker A, Walker B, Q-loop, D-loop, H-loop, and ABC signature motifs, while each TMD is made up of five to six transmembrane α-helices that dictate substrate specificities [27]. ABC transporter proteins require two NBDs and two TMDs for functionality. Some ABC transporters are full-transporters (FT) in that two TMDs and two NBDs are encoded in a single protein, whereas most are half-transporters (HT; one TMD and one NBD) and form functional units following homodimerization or heterodimerization [24, 27, 28]. Due to the relatively conserved sequence of the NBD, it has been used for the phylogenetic classification of the ABC transporter superfamily into eight subfamilies designated A to H (ABCA to ABCH) [29].

Among insect species, ABC transporters are implicated in diverse functions, including transportation of eye pigments [30–34], and resistance to chemical insecticides [35, 36]. Within the model species for Coleoptera, the red flour beetle, *Tribolium castaneum*, Broehan et al. [30] reported that RNAi-mediated knockdown of some ABC transporters resulted in mortality, or phenotypes characterized by arrested growth, abnormal cuticle formation, defective eye pigmentation, or abnormal egg-laying or -hatching. Changes in the expression level or structure of some ABCA, ABCC and ABCG subfamily members have been associated with Bt toxin resistance in species of Lepidoptera [37], while paralogs of an ABCB transporter were linked to Bt Cry3Aa resistance in the coleopteran species, *Chrysomela tremula* [38], and were found to be in proximity to a quantitative trait locus (QTL) for Cry3Bb1 resistance in *D. v. virgifera* [39].

Similar investigations of ABC transporters in *D. v. virgifera* are arguably limited due to the dearth of genomic resources available for this species, which are currently comprised of Sanger and Roche 454 read-based transcriptome assemblies [40–43]. Complicating the development of genomic tools is the 2.58 GB size and complex repetitive structure of the *D. v. virgifera* genome [4, 44]. Regardless, RNA sequencing (RNA-seq) has become an expeditious and cost-effective method for obtaining a wealth of transcriptome sequence data in non-model insects [45]. In the following, a de novo transcriptome assembly approach was used for the first prediction, annotation, and functional analysis of the ABC transporter gene family in *D. v. virgifera*. Specifically, eight ABC transporters were identified as putative orthologs to those previously reported to have a defining RNAi phenotype in the model coleopteran species, *T. castaneum* [30] (*DvvABCA_50718, DvvABCB_39715, DvvABCE_2830, DvvABCF_2701, DvvABCG_3712, DvvABCG_14042, Dvvw* and *DvvABCH_5118*). Subsequent RNAi-mediated knockdown demonstrated conservation of function with *T. castaneum*, as well as established potential new insecticidal targets for the control of this devastating agricultural pest.

## Results

### Transcriptome sequencing, assembly, and annotation

**Fig. 1:**
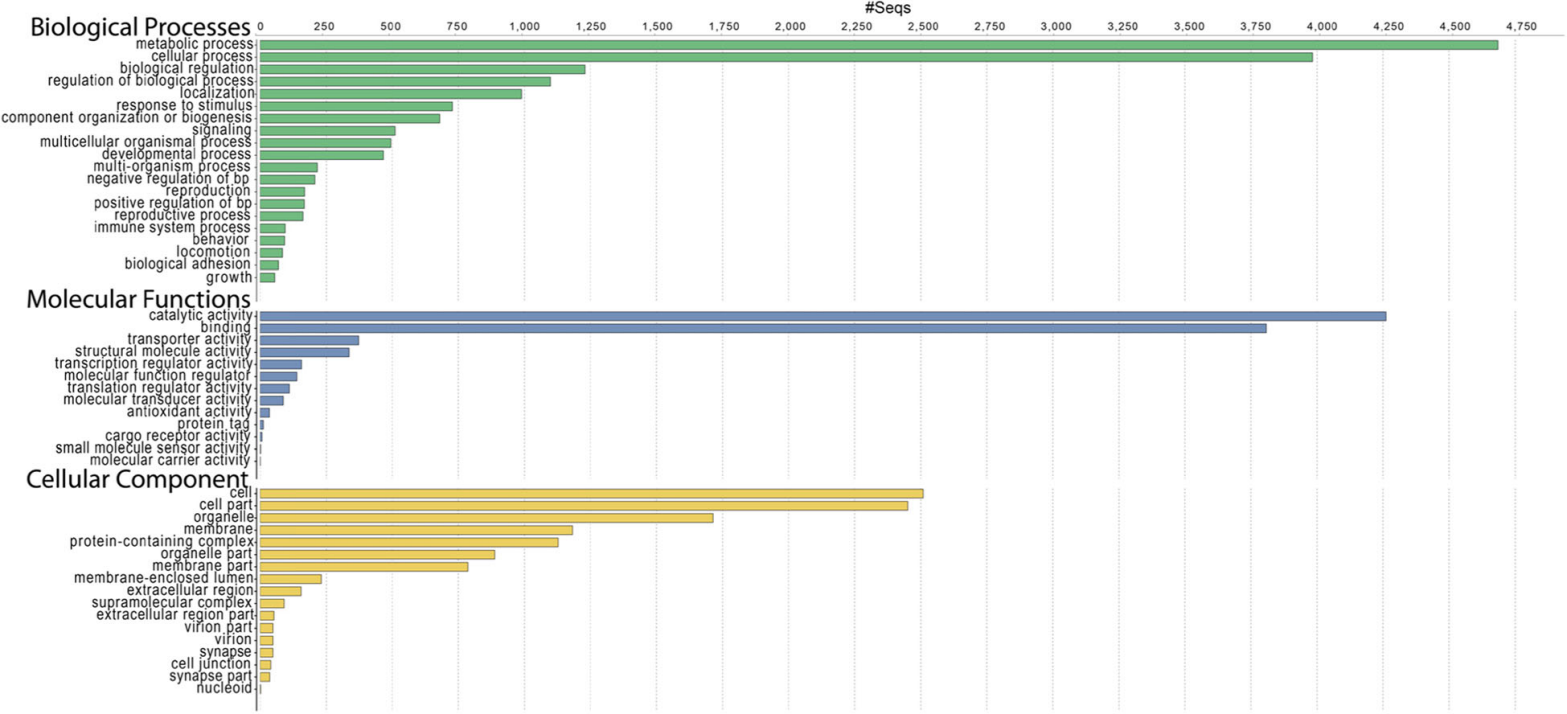
Gene ontology classification of the *D. v. virgifera* transcriptome. GO Distribution by Level (2) – Top 20

Over 22 million raw Illumina (MiSeq) sequencing reads were generated across four libraries (Table 1; NCBI SRA database accession SRP161473: experiments SRX4669438 to SRX4669441). DNASTAR assembled 13,070,671 reads into a combined transcriptome containing 25,296 contigs with an N50 of 1604 bp (Additional file 1: Table S1). Analogously, assemblies from Trinity and SOAPdenovo-Trans respectively produced 162,897 and 133,180 contigs, each with an N50 ≤ 439 bp (Additional file 1: Table S1). Clustering by CD-HIT-EST reduced complexity 3.5 to 34.9% across assemblies, and the number of predicted open reading frames (ORFs) within clustered transcripts ranged from 18,305 to 40,087 (Additional file 1: Table S1). BLASTx query of transcripts by Blast2GO against the arthropod-specific section of NCBI’s non-redundant (nr) protein database generated annotations for 18,343 DNASTAR contigs (*E*-value cutoff of 10^− 6^), with a subset of these receiving gene ontology (GO) mapping and additional annotation terms (Additional file 2: Figure S1). Sequences lacking identity to known arthropod proteins above *E*-value thresholds were attributed to poor sequence conservation and/or novel sequences, as well as non-coding RNAs. The distribution of top BLASTx hits by species showed that *T. castaneum* was the most frequent, representing 65% of the matches (Additional file 3: Figure S2). Among ontologies assigned via mapping at GO level 2, a majority of the associated terms were assigned to cell structural component, metabolic process, and catalytic activity respectively for GO Cellular Component, Biological Process and Molecular Function (Fig. 1). The DNASTAR assembly showed a high degree of completeness based on a BUSCO score of 928, or 89.6%, of the 1066 genes in the arthropod reference set (v. 9.0) being represented, with analogous levels of representation in both SOAPdenovo-Trans and Trinity assemblies (Additional file 1: Table S1).

**Table 1:** Paired-end RNA-sequencing libraries and sequencing

			MiSeq	Raw read data		Trimmed read data	
ID	Lib_name	Insert	Lanes	Length	Count	Paired	Unpaired
Af1	DvvAdultF_R1	600 to 700-bp	1	300-bp	3,462,470	2,753,272	559,611
Af2	DvvAdultF_R2			300	3,462,470	2,753,272	72,301
Am1	DvvAdultM_R1	600 to 700-bp	1	300	2,223,027	1,859,087	308,133
Am2	DvvAdultM_R2			300	2,223,027	1,859,087	23,335
E1	DvvEggs_R1	600 to 700-bp	1	300	2,690,038	2,146,192	425,023
E2	DvvEggs_R2			300	2,690,038	2,146,192	62,149
L1	DvvLarvae_R1	600 to 700-bp	1	300	2,652,196	2,071,100	445,600
L2	DvvLarvae_R2			300	2,652,196	2,071,100	62,923
				Totals	22,055,462	17,659,302	1,959,075

### Bioinformatic analysis of the *D. v. virgifera* ABC transporter family

**Fig. 2:**
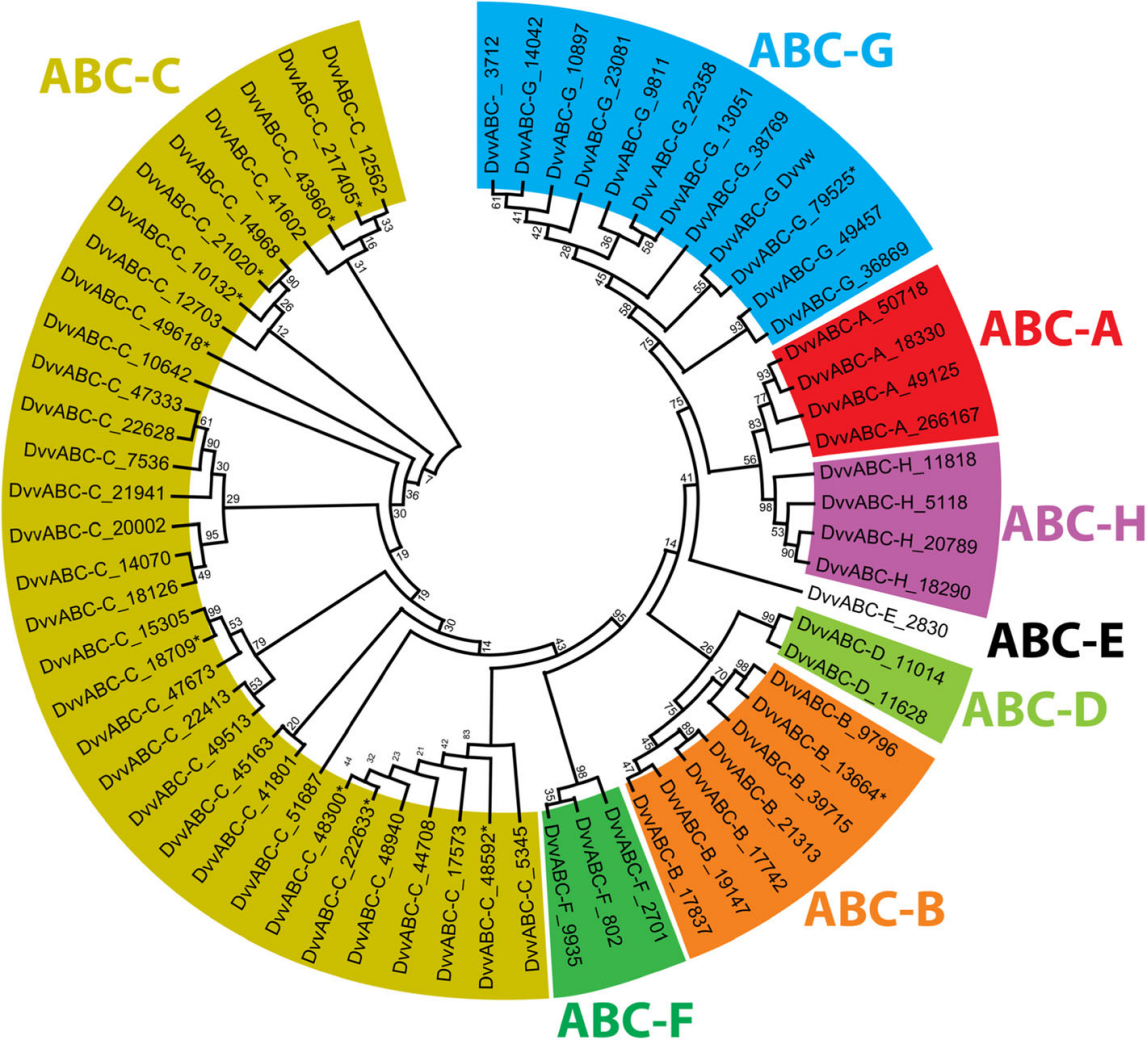
Intraspecific phylogenetic relationships among *D. v. virgifera* ABC transporters*.* Clades corresponding to subfamilies A-H are indicated by color. Bootstrap values are given at the internodes as percentage of 1000 pseudoreplicates

Results of BLASTx queries identified 65 putative *D. v. virgifera* ABC transcripts that shared ≥37% amino-acid identity with putative *T. castaneum* orthologs from ABC transporter subfamilies A through H (Table 2; Additional file 4: Table S2). Predictions of protein structural domains identified both FTs and HTs. Four *Dvv*ABCA and 32 *Dvv*ABCC subfamily members were predicted for *D. v. virgifera*, all of which are FTs. The *Dvv*ABCB subfamily contained seven members, which included both full- and half-transporters. The number of assembled *D. v. virgifera* paralogs within subfamilies ABCD, ABCE and ABCF contained a smaller number compared to *Dvv*ABCB, but each had predicted orthologous relationships to *T. castaneum* ABC transporters. Specifically, the *Dvv*ABCD subfamily contained two predicted ABC transporter proteins which were both HTs. One *Dvv*ABCE and three *Dvv*ABCF members were identified, and each of these had two predicted NBD motifs with no TMDs, suggesting that, like their counterparts in other species, they probably do not function as transmembrane transporters. The *Dvv*ABCG subfamily contained the second largest number of predicted members with 12, all of which were HTs with only a single NBD and a reverse domain organization. The *Dvv*ABCH subfamily contained four members, which were similar to those of the ABCG subfamily in being HTs with a reverse domain organization. The phylogenetic relationships predicted among NBD regions of deduced *D. v. virgifera* ABC transporter protein sequences formed distinct clades corresponding to the eight known ABC transporter subfamilies A to H (Fig. 2; Additional file 5: Figure S3).

**Table 2:** Classification of *D. v. virgifera* ATP binding cassette (ABC) transporters

*Diabrotica virgifera virgifera* transcript		Nearest *Tribolium castaneum* ortholog		
Gene ID	Length (aa)	Published Name	Accession	Identity (%)
*DvvABCA_18330*	1756	*TcABCA-UD*	XP_008199148.1	58
*DvvABCA_50718*^b^	1707	*TcABCA-UD*^c^	XP_008199148.1	56
*DvvABCA_49125*	1643	*TcABCA-7A*	XP_008195104.1	41
*DvvABCA_266167*	1640	*TcABCA-6A*	XP_008195056.1	52
*DvvABCB_21313*	1246	*TcABCB-3A*	XP_00819082.1	59
*DvvABCB_17742*	1256	*TcABCB-3B*	XP_008191266.1	63
*DvvABCB_19147*	666	*TcABCB-4A*	XP_008192744.1	72
*DvvABCB_39715*^b^	715	*TcABCB-5A*	XP_001813375.1	75
*DvvABCB_9796*	833	*TcABCB-6A*	XP_008194672.1	75
*DvvABCB_13664*^a^	657	*TcABCB-6A*	XP_008194672.1	77
*DvvABCB_17837*	681	*TcABCB-7A*	XP_972133.2	69
*DvvABCC_41801*	1267	*TcABCC-5U*	XP_969849.1	36
*DvvABCC_44708*	1256	*TcABCC-5P*	XP_015836131.1	43
*DvvABCC_48952*^a^	1251	*TcABCC-5N*	XP_971802.2	48
*DvvABCC_17573*	1284	*TcABCC-5N*	XP_971802.2	45
*DvvABCC_51687*	1555	*TcABCC-9A*	XP_008197311.1	71
*DvvABCC_21020*^a^	1296	*TcABCC-5H*	XP_968748.1	52
*DvvABCC_222633*^a^	1233	*TcABCC-5P*	XP_015836131.1	46
*DvvABCC_18126*	1342	*TcABCC-5U*	XP_969849.1	56
*DvvABCC_49513*	1373	*TcABCC-5T*	XP_969781.1	55
*DvvABCC_14070*	1342	*TcABCC-5U*	XP_969849.1	54
*DvvABCC_22628*	1349	*TcABCC-5R*	XP_008193834.1	55
*DvvABCC_20002*	1344	*TcABCC-5U*	XP_969849.1	56
*DvvABCC_7536*	1363	*TcABCC-5R*	XP_008193834.1	60
*DvvABCC_47333*	1376	*TcABCC-5R*	XP_008193834.1	58
*DvvABCC_49618*^a^	1033	*TcABCC-5I*	XP_015835265.1	73
*DvvABCC_45163*	1535	*TcABCC-4A*	XP_008192060.1	60
*DvvABCC_43960*^a^	1081	*TcABCC-5H*	XP_968748.1	49
*DvvABCC_48940*	1223	*TcABCC-5Q*	XP_015836083.1	43
*DvvABCC_217405*^a^	1164	*TcABCC-5H*	XP_968748.1	52
*DvvABCC_10132*^a^	870	*TcABCC-5B*	XP_973693.2	55
*DvvABCC_48300*^a^	1257	*TcABCC-5P*	XP_015836131.1	47
*DvvABCC_47673*	1323	*TcABCC-5T*	XP_969781.1	71
*DvvABCC_5345*	1257	*TcABCC-5N*	XP_971802.2	43
*DvvABCC_22413*	1330	*TcABCC-5T*	XP_969781.1	54
*DvvABCC_18709*^a^	1259	*TcABCC-5T*	XP_969781.1	63
*DvvABCC_21941*	1328	*TcABCC-5R*	XP_008193834.1	55
*DvvABCC_15305*	1323	*TcABCC-5T*	XP_969781.1	63
*DvvABCC_12562*	1319	*TcABCC-5H*	XP_968748.1	53
*DvvABCC_10642*	1306	*TcABCC-7B*	XP_972534.1	63
*DvvABCC_41602*	1307	*TcABCC-5H*	XP_968748.1	54
*DvvABCC_12703*	1317	*TcABCC-5H*	XP_968748.1	55
*DvvABCC_14968*	1309	*TcABCC-5H*	XP_968748.1	56
*DvvABCD_11014*	754	*TcABCD-6A*	XP_971218.1	75
*DvvABCD_11628*	657	*TcABCD-9A*	XP_015838765.1	80
*DvvABCE_2830*^b^	608	*TcABCE-3A*	XP_968009.1	91
*DvvABCF_2701*^b^	921	*TcABCF-2A*	XP_971562.1	90
*Dvv BCF_802*	623	*TcABCF-5A*	XP_966990.1	92
*DvvABCF_9935*	710	*TcABCF-9A*	XP_972814.1	83
*DvvABCG_9811*	659	*TcABCG-4A*	XP_008192053.1	68
*DvvABCG_3712*^b^	667	*TcABCG-4C*	XP_001813184.1	77
*DvvABCG_14042*^b^	719	*TcABCG-4D*^c^	XP_973458.1	76
*DvvABCG_10897*	651	*TcABCG-4G*	XP_008192849.1	62
*DvvABCG_22358*	640	*TcABCG-4B*	XP_015834971.1	62
*DvvABCG_23081*	603	*TcABCG-4F*	XP_971735.1	53
*DvvABCG_13051*	637	*TcABCG-4E*	KYB28165.1	60
*DvvABCG_38769*	621	*TcABCG-4H*	XP_973526.1	53
*DvvABCG Dvvw*^b^	657	*Tcw*	NP_001034521.1	60
*DvvABCG_49457*	940	*TcABCG-9C*^c^	XP_968472.1	73
*DvvABCG_36869*	642	*TcABCG-9D*	XP_968555.2	71
*DvvABCG_79525*^a^	651	*Tcst*	NP_001306193.1	63
*DvvABCH_20789*	713	*TcABCH-9A*	XP_973444.1	55
*DvvABCH_5118*^b^	795	*TcABCH-9C*	XP_008198312.1	83
*DvvABCH_18290*	703	*TcABCH-9A*	XP_973444.1	43
*DvvABCH_11818*	762	*TcABCH-9B*	XP_967359.1	71

^a^Incomplete sequences, ^b^RNAi targets, ^c^not ortholog with phenotype in [30] – see text for details

### Gene expression across developmental stages

**Fig. 3:**
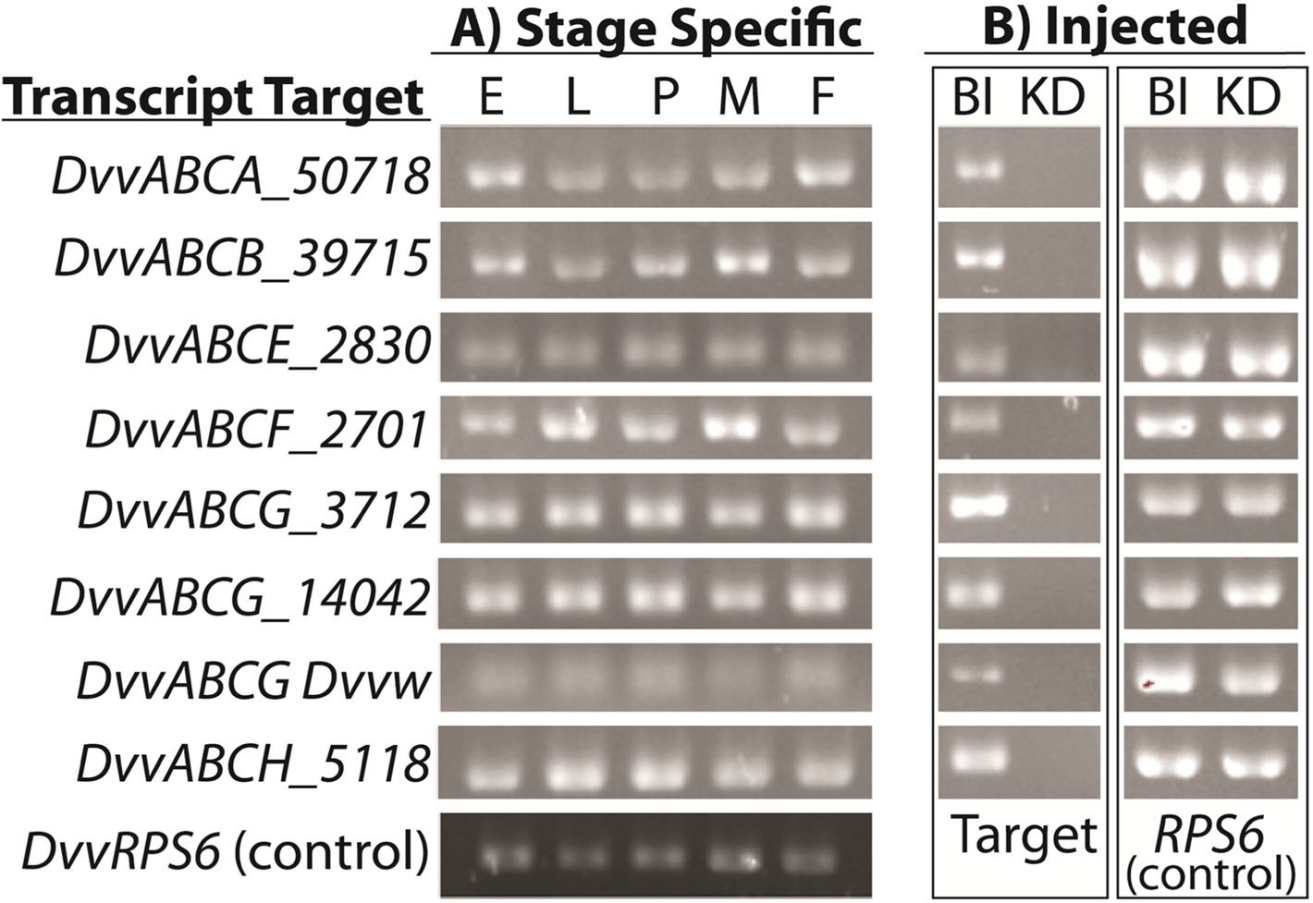
Semi-quantitative PCR results of select *D. v. virgifera* ABC genes. **a** Developmental stage-specific expression profile of select transcripts. RNA isolated from Eggs (E), Larvae (L), Pupae (P), and adult Males (M) and Females (F) for each of the eight genes. *DvvRPS6* was used as a positive control. **b** Assessment of target RNA levels in injected individuals. RNA was isolated 5 days after injection from pools of buffer injected (BI) individuals, and of dsRNA injected (KD) individuals. *DvvRPS6* was used as a control to assess template quality

Since prior research in *T. castaneum* revealed that only 10 ABC transporters had obvious phenotypic consequences following RNAi-mediated knockdown [30], our study focused on functional analysis of their predicted *D. v. virgifera* orthologs. From this list, our initial predictions from the *D. v. virgifera* transcriptome (DNASTAR assembly) identified eight orthologs (Table 3). Differences resided in that *T. castaneum* has two closely related ABCA genes (*TcABCA-9A* and *TcABCA-9B*) which appear to represent a *T. castaneum*-specific duplication (Additional file 5: Figure S3). We were unable to identify a direct ortholog for these genes, but the closest homolog we found in the *D. v. virgifera* transcriptome appeared to be *DvvABCA_50718*. Analogously, we were unable to identify a direct ortholog to TcABCG-8A, so we targeted *DvvABCG_14042*, the closest identifiable homolog according to BLASTp results. Finally, while the ABCG genes *TcABCG-9A* and *TcABCG-9B* represent the orthologs of the *T. castaneum* eye-color genes *scarlet* and *white*, respectively [32], results of BLASTx searches of the DNASTAR assembly resulted only in the identification of an ortholog of *white, Dvvw* [46]. Semi-quantitative PCR of these eight *D. v. virgifera* ABC transcripts showed that all are expressed across all of the developmental stages examined (Fig. 3a).

**Table 3:** Results of RNAi knockdown of selected ABC transporters

Transcript	Stage	KD	Phenotype	Figure
*DvvABCA_50718*	Pre-pupal	60%	Deformed wings & elytra	4A
*DvvABCB_39715*	Larval	100%	Lethal	NS
	Pre-pupal	100%	Defect in pupal-adult molt	4B
	Eclosed females	0%	Malformed ovaries; low egg lay	4J
*DvvABCE_2830*	Larval	100%	Lethal	4G
*DvvABCF_2701*	Larval	100%	Lethal	4H
*DvvABCG_3712*	Pre-pupal	80%	Lethal; pupal developmental arrest	4C
	Eclosed females	0%	Prevented embryonic development	4I
*Dvvw*	Pre-pupal	0%	Pigmentation defect; white eyes	4E
*DvvABCG_14042*	Larval	100%	Lethal at molting	NS
	Pre-pupal	80%	Lethal pupal developmental arrest	4D
*DvvABCH_5118*	Larval	100%	Lethal at molting	NS

*KD* knockdown, *NS* image not shown

### RNAi knockdown phenotypes

**Fig. 4:**
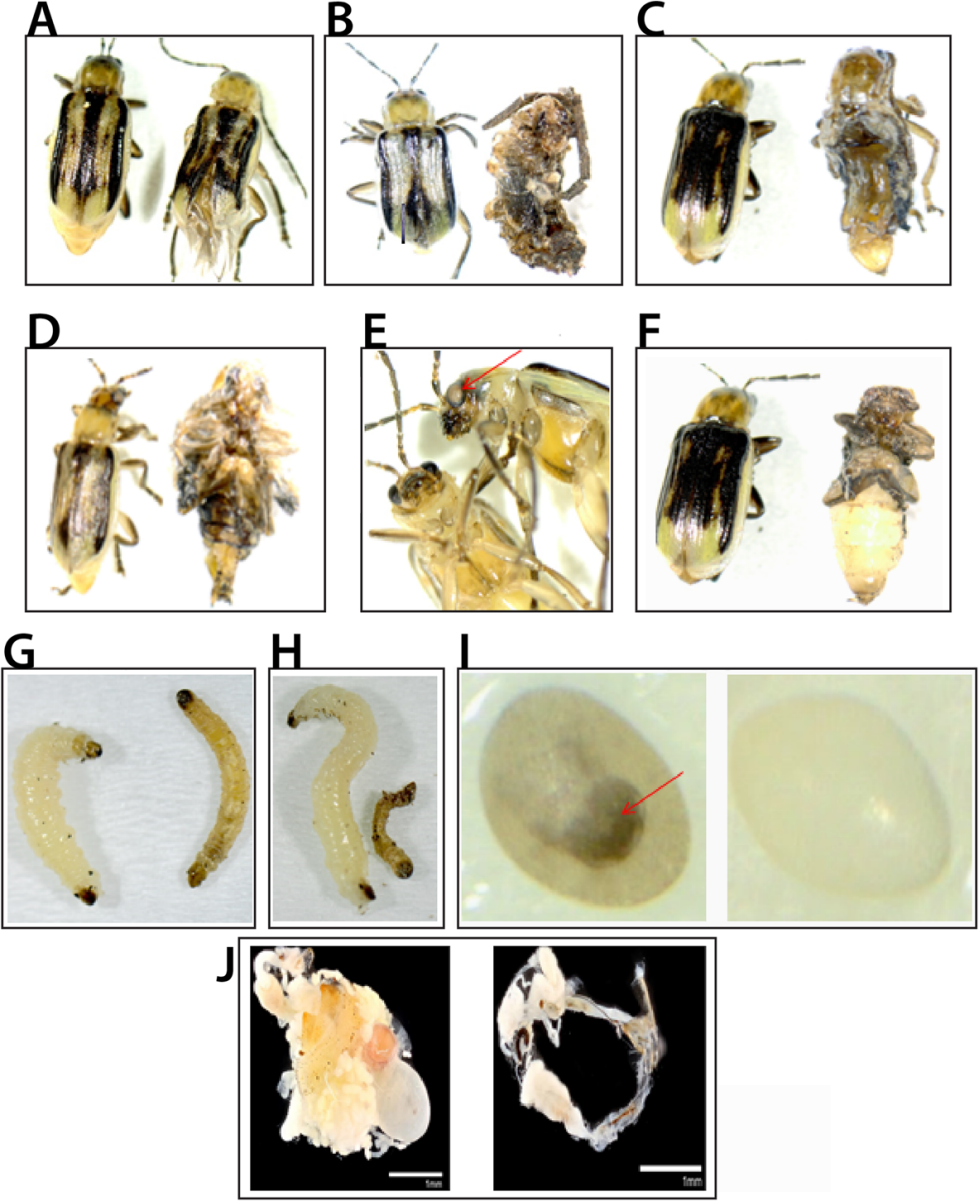
Effects of *DvvABC* transporter-specific RNAi on *D. v. virgifera* development. Buffer-injected controls (right) are shown next to dsRNA-injected individuals. **a** Injection of *DvvABCA_50718*-specific dsRNA into pre-pupae (PP) caused defects in adult wing development, while **b** PP injection of dsRNA for *DvvABCB_39715* caused molting defects during eclosion. **c**, **d** and **f** Injection of dsRNAs for *DvvABCG_3712, DvvABCG_14042* or *DvvABCH_5118* into PP each resulted in severe molting defects during eclosion. **e** PP injection of *Dvvw-*specific dsRNA caused loss of eye pigmentation (arrow), while (**g**-**h**) larval injection of *DvvABCE_2830* or *DvvABCF_2701* resulted in a reduction in body mass and death prior to molting. **i** Injection of *DvvABCG_3712-*specific dsRNA into adult females interfered with embryonic development (arrow indicates location of head capsule in a control embryo). **j** *DvvABCB_39715*-specific dsRNA injected into adult females disrupted ovary development

**Fig. 5:**
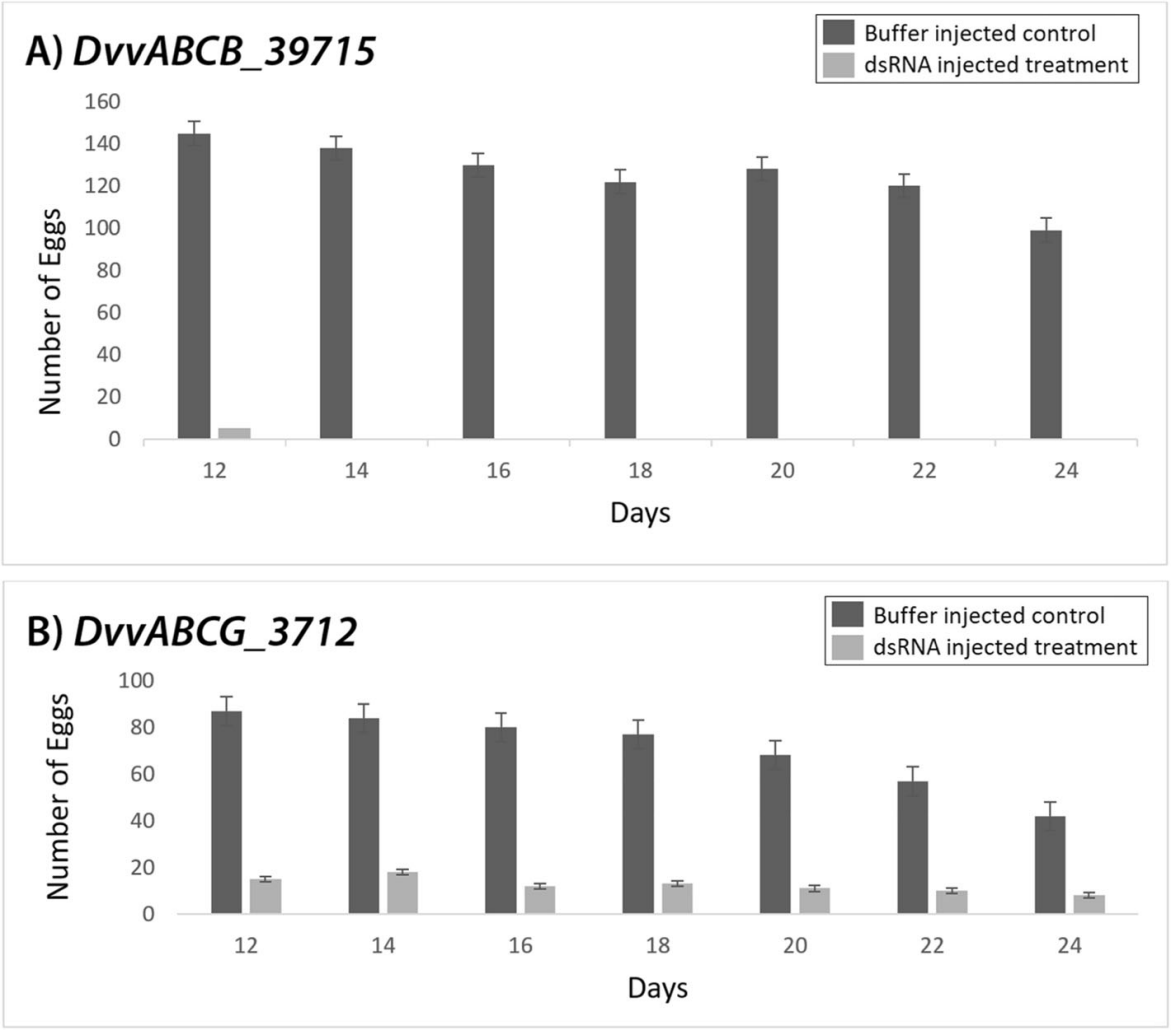
Effects of injected dsRNA on egg laying. Effects on oviposition following injections of dsRNA targeting **a** *DvvABCB_39715* and **b** *DvvABCG_3712*. Females injected with *DvvABCB_39715* dsRNA failed to lay eggs, and those injected with *DvvABCG_3712* dsRNA laid fewer eggs and those that were laid failed to develop. In both cases, buffer-injected females lay near-normal numbers. The eggs laid within a period of 2 weeks were counted every other day

Different growth stages of *D. v. virgifera* were microinjected with dsRNAs (Table 3), after which the level of each corresponding transcript was below or nearly below semi-quantitative PCR detection limits. Specifically, the level of each targeted *D. v. virgifera* transcript was reduced at 5-days post-injection as compared to buffer-injected controls (Fig. 3b). Moreover, injection of each of the eight dsRNAs resulted in defined phenotypes among dsRNA treated cohorts (Table 3; Fig. 4). The knockdown of *DvvABCA_50718* led to approximately 60% mortality among treated pre-pupae, compared to 5% for the buffer-treated control group. In addition, the adults that survived pre-pupal injection and successfully eclosed had defects in their wings and elytra (Fig. 4a), while no phenotypic effects were observed among buffer-injected controls. Injection of *DvvABCB_39715* dsRNA into larvae resulted in 100% mortality, and injections into pre-pupae led to defects in their development, which caused individuals to be unable to complete the pupal-adult molt and ultimately resulted in 100% mortality (Fig. 4b). Knockdown of *DvvABCB_39715* in newly-eclosed adult female *D. v. virgifera* resulted in significant reduction in egg laying compared to untreated females (Fig. 5a). Upon further investigation, we discovered that injection of this dsRNA also affected ovary development, causing underdeveloped ovaries, hence the failure to produce eggs (Table 3; Fig. 4j).

RNAi-mediated knockdown of *DvvABCE_2830* and *DvvABCF_2701* in larvae resulted in 100% mortality. Prior to death, it was noted that the body mass of treated individuals was less than that of similarly-aged larvae treated with buffer alone (Fig. 4g, h). Analogously, injection of *DvvABCE_2830* and *DvvABCF_2701* dsRNA separately into pre-pupae both caused 100% mortality with no adult eclosion (results not shown). Injection of dsRNA specific for *DvvABCH_5118* into early-instar *D. v. virgifera* larvae and pre-pupae caused development to arrest as individuals prepared to molt, thus resulting in 100% mortality (Fig. 4f). Affected individuals appeared to desiccate prior to death (personal observation).

Injection of dsRNA targeting *DvvABCG_3712, DvvABCG_14042,* and *Dvvw* resulted in phenotypes similar to those seen with RNAi knockdown of the corresponding *T. castaneum* orthologs [30]. Specifically, injection of dsRNA targeting *Dvvw*, gave the expected white-eye phenotype (Fig. 4e); indeed, we had identified this *white* ortholog previously [46]. Injection of *DvvABCG_3712* dsRNA into pre-pupae caused developmental defects that resulted in 80% mortality (Table 3; Fig. 4c). Interestingly, adult females treated with *DvvABCG_3712* dsRNA produced fewer eggs compared to females injected with buffer alone (Fig. 5b), and the eggs that were laid lacked obvious signs of embryonic development (Fig. 4i) and ultimately failed to hatch (Additional file 6: Figure S4). Injection of *DvvABCG_14042* dsRNA into larvae and pre-pupae resulted in molting defects; about 80% of these died during their next molt (Table 3), while the 20% that survived through subsequent larval molts died following pupation (Fig. 4d).

## Discussion

In recent years, ABC transporters have become a major focus for research in arthropods. This is in part due to their overall role in xenobiotic transport and insecticide resistance [25, 47–50], but more specifically, due to their suspected role in susceptibility to Bt toxins [38, 51, 52]. For example, Gahan et al. [53] reported genetic linkage of *Heliothis virescens HvABCC2* with resistance to Cry1Ac, while changes in the structure, splicing, or expression level of *ABCC2* orthologs were later associated with Cry1Ac resistance in *Helicoverpa armigera* [54], *Bombyx mori* [55], and *Spodoptera exigua* [56]. Indeed, expression of the *P. xylostella ABCC2* ortholog in *Drosophila melanogaster* conferred susceptibility to this lepidopteran-specific toxin [57]. An *ABCC2* ortholog is also linked to Cry1F resistance in *Ostrinia nubilalis* [58] and *S. frugiperda* [59]. Additionally, structural mutations in a member of subfamily A, *HaABCA2*, were implicated in Cry2Ab resistance in *H. armigera* [60], and, more recently, researchers were able to recapitulate an *ABCA2* resistance allele in a susceptible population of *H. armigera* [61], providing further evidence for the importance of normal *ABCA2* function in Cry2Ab toxicity. Reduced expression of ABCG members have been associated with Cry1Ac resistance in *P. xylostella* [62], as well as Cry1Ac and Cry1Ab resistance in *O. furnacalis* [63]. More recent studies in species of Coleoptera have implicated ABCB subfamily members in Cry3Aa resistance in *C. tremula* [38] and in Cry3Ab1 resistance in *D. v. virgifera* [39].

The study of ABC transporters in several arthropod species have relied on genomic data, including *T. castaneum* [30], *Aethina tumida* [64], *B. mori* [33], *D. melanogaster* [28], *Bemisia tabaci* [50], *Daphnia pulex* [65], and *Tetranychus urticae* [66]. Due to the status of *D. v. virgifera* as a major pest of cultivated maize (see Introduction) and current fragmented state of the unpublished draft genome assembly of this species (GenBank accession PXMJ00000000.2), the Illumina-based transcriptome assemblies reported here represent a particularly valuable genetic tool for gene discovery, characterization, and genome annotation. In particular, the 65 ABC transporter genes we identified are expected to be useful in downstream studies on insecticide resistance traits in *D. v. virgifera*.

Broehan et al. [30] previously identified 73 ABC transporters in *T. castaneum*, and a 74th ABC transporter was more recently reported by Grubbs et al. [32]. There are several possible reasons for why the 65 *Dvv*ABC transporters we identified are comparatively fewer than in *T. castaneum*. Firstly, our transcriptome was derived from lower-throughput sequencing data (Illumina MiSeq), therefore genes expressed at very low levels may not have been represented within our raw Illumina data. Secondly, our RNA-seq libraries were not comprehensive of all possible life/growth stages or conditions, such that transcripts not expressed during growth states or under conditions used in this study would have been missed. Regardless, BLASTx analyses of the 65 putative *D. v. virgifera* ABC transporters identified in this study demonstrate their greatest sequence similarity to *T. castaneum* and *A. glabripennis* orthologs. This is probably a consequence of the extensive publicly available genomic data for both *T. castaneum* and *A. glabripennis*, as well as their close phylogenetic relationships to *D. v. virgifera*. Furthermore, the putative one-to-one relationship among orthologs from *D. v. virgifera* and *T. castaneum* may suggest the retention of copy number without extensive gene loss or gain across evolutionary time.

Despite the relatively large amount of genomic and transcriptomic data available for model and some non-model coleopteran species, there is a comparative overall dearth of functional data available to support automated computational annotations. To partially address this shortfall, we generated functional information based on RNAi knockdown of eight *D. v. virgifera* ABC transporters, each of which demonstrated fairly conserved roles relative to their *T. castaneum* orthologs [30]. While some *D. v. virgifera* RNAi-mediated loss-of-function phenotypes include visible developmental defects, such as loss of eye pigmentation, others cause growth arrest and/or death. For example, knockdown of *DvvABCA_50718* led to death during the pupal-to-adult molt and also caused deformation of wings and elytra in surviving adult beetles, which was the same as previously seen in *T. castaneum* [30] (Table 3; Fig. 4a). Since subfamily A transporter members are implicated in mammals with lipid transport, which can impact cell physiology [67], it is conceivable that the effects of the knockdown of *DvvABCA_50718*, and of its homologs, *TcABCA-9A/9B*, in *T. castaneum* [30], could be the result of disrupting critical lipid transport. *DvvABCB_39715* RNAi also recapitulated the lethal effects of its *T. castaneum* ortholog; the effects on female fecundity could make this gene a particularly interesting target for RNAi-based pest control. It is worth noting that *D. v. virgifera* is predicted to have one more ABCB HT subfamily member compared to other insects [25], especially other beetles [30, 64]. While ABCB FTs have been implicated in chemical insecticide resistance among insects [47], HTs are known to be mitochondrial transporters in humans, with roles in iron metabolism and transportation of Fe/S protein precursors [68, 69]. These possibilities were outside the scope of our research, but future investigations into the function of *Dvv*ABCBs could be beneficial for deciphering mechanisms of resistance evolution in *D. v. virgifera*.

RNAi knockdown of *DvvABCE_2830* and *DvvABCF_2701* resulted in 100% larval mortality. ABCE and ABCF subfamilies are highly conserved across all phyla, and due to their lack of TMDs are considered non-transporters. Instead, they appear to play roles in regulating translation [70, 71], indicating that ABCE and ABCF proteins are essential. Thus, given that these genes are highly conserved across taxa in sequence, function, and RNAi phenotype [30], it may not be surprising that lethal RNAi knockdown phenotypes were obtained in *D. v. virgifera*.

The phenotypes observed following independent RNAi knockdown of *DvvABCG_14042* and *DvvABCH_5118* involved molting defects that resulted in near complete mortality. While these results are consistent with functional analysis of their *T. castaneum* orthologs, RNAi knockdown of the *DvvABCG_14042* homolog *TcABCG-8A* in *T. castaneum* produced an additional phenotype of premature development of compound eyes [30]. In contrast, we did not observe any analogous eye phenotypes in *D. v. virgifera* following RNAi knockdown. It is likely that since the injected *D. v. virgifera* larvae died prior to reaching the next stage of development, there was no opportunity for compound eyes to form. In other species, orthologs of *DvvABCH_5118* are known to transport cuticular lipids that are deposited in the outer epicuticle layer to form a waterproof barrier [30, 62]. Therefore, it could be that cuticular lipid deposition may be reduced following RNAi knockdown of this ABCH transporter, which could promote desiccation and subsequent mortality of affected individuals.

The ABCG proteins are HTs, and, with 12 predicted members, form the second largest subfamily of ABC transporters identified in *D. v. virgifera* (Table 2). Among insects, some of the first ABCGs to be characterized were the pigment transporters (*white*, *scarlet* and *brown*) in *D. melanogaster* [34, 72]. Mutants of *white* are characterized by white eyes (i.e. complete loss of eye pigmentation), *scarlet* mutants by bright red eyes (i.e. loss of brown pigments), and *brown* mutants by dark brown eyes (i.e. loss of red pigments) [34]. Studies have revealed that some ABCG proteins perform other crucial physiological roles in the transport of lipids, sterols, and drugs [73]. In the current study, RNAi-mediated knockdown of *Dvvw* resulted in a white-eyed phenotype consistent with prior observations in *T. castaneum* [30], and with our own previous findings in *D. v. virgifera* [46]. Our findings support a prediction that *Dvvw* is part of the ommochrome pathway, where it is likely acting within a heterodimeric complex to import ommochrome pigments into the pigment granules of the compound eye. As mentioned above, loss of *white* function in *D. melanogaster*, results in white-eyed flies, while mutations in *scarlet* lead to red-eyed flies. However, RNAi-mediated knockdown of the corresponding gene, *ABCG-9A* (*scarlet*), in *T. castaneum* produces white-eyed beetles [30, 32]*.* This finding was not surprising, since a previous report of RNAi targeting *vermilion*, a pivotal gene in the ommochrome pathway, also generates a white-eyed phenotype in *T. castaneum* [74], leading the authors to conclude that the *T. castaneum* eye is pigmented by ommochromes alone, and that the ommochrome biosynthetic pathway in *T. castaneum* produces red pigments as end products, rather than brown pigments as in *D. melanogaster.* Unfortunately, our initial survey of the *D. v. virgifera* transcriptome failed to identify a *scarlet* ortholog in our DNASTAR assembly, thus its function was not assessed. We did identify a *scarlet* ortholog from the Trinity assembly (See Table 2 and Additional file 5: Figure S3) after we had completed our functional analyses, but we were still unable to find any evidence of a *brown* ortholog. So, it will be interesting to investigate in future studies if pigmentation of the *D. v. virgifera* eye is more similar to that of *T. castaneum* or *D. melanogaster*. Specifically, in *D. melanogaster* a third ABCG transporter, *brown*, is required for wild-type pigmentation of the eye. In flies, Brown heterodimerizes with White and transports pteridine-based pigments into the eye. Although an ortholog of *brown* has been identified in the *T. castaneum* genome, no function has been identified [32].

## Conclusion

This study provides a relatively large transcriptomic resource comprising genes expressed across several life stages of the arthropod pest species, *D. v. virgifera*. Due to potential omission of orthologs from our assembly, undoubtedly additional research will need to be performed in order to identify the full compliment of ABC transporters encoded by *D. v. virgifera,* and further functional assays will be needed to validate putative biochemical roles. Regardless, our work represents the initial description of the ABC transporter gene family in *D. v. virgifera*. Furthermore, the knockdown of ABC transporters *DvvABCB_39715* and *DvvABCG_3712*, each of which reduced egg production and/or prevented embryonic development, could provide novel targets for *D. v. virgifera* population suppression and use as an insecticidal control agent. This research is a contribution to a growing set of genomic resources for arthropods, and provides information that may facilitate the development of methods to enhance the control of a devastating agricultural pest species.

## Methods

### Insect rearing

All *D. v. virgifera* used in this study are nondiapausing, from a colony previously established at North Carolina State University using beetles obtained from both Dr. Wade French (USDA-ARS-NGIRL, Brookings, SD) and Crop Characteristics, Inc. (Farmington, MN, USA) (see [75]). Eggs deposited in an oviposition chamber (agar plate with cheese cloth) were collected weekly, pipetted into soil-filled containers, and held at 26 °C for 1 week. Larvae were reared on roots of germinated corn seed in 16-oz containers, while adults were maintained in a 30cm^3^ BugDorm (MegaView Science, Taiwan) at 26 °C, 70% relative humidity with an L14:D10 photoperiod and fed an artificial diet (Western Corn Rootworm w/o Pollen Substitute, Frontier Insect Diet, Newark, DE, USA). Injected individuals were reared in small containers with corn seedlings to allow downstream observation.

### Transcriptome sequencing, assembly, and annotation

Total RNA was extracted from mixed-staged *D. v. virgifera* embryos (*n* = 500 from an overnight egg lay aged up to 14 days), mixed-stage larvae (first-instar larvae (*n* = 20); second-instar larvae (*n* = 10); and third-instar larvae (*n* = 2), as well as an adult male, and an adult female (*n* = 1 each) using the RNeasy Mini Kit (Qiagen, Hilden, Germany) and treated with DNase I (Qiagen) according to the manufacturer’s instructions. The isolated total RNA was submitted to the Genomic Sciences Laboratory (North Carolina State University, NC, USA) for quality assessment, poly(A) selection, fragmentation, selection of ~ 650 bp fragment sizes, Illumina TruSeq® library preparation, and 300 bp paired-end sequencing on an Illumina MiSeq sequencer (Illumina, San Diego, CA, USA).

Raw FASTQ reads for each library were assessed using FastQC [76]. Reads were initially imported into SeqMan NGen ® (DNASTAR, Madison, WI, USA), where onboard scripts were used to quality trim and de novo assemble reads into contigs using default settings. Additionally, raw reads from individual libraries were trimmed of Illumina adapter sequence contamination, bases having Phred quality score < 20 (*q* < 20), and sequence reads < 35 bp using Trimmomatic 0.32 [77]. Resulting trimmed read pairs from each library were concatenated into single R1- and R2-specific FASTQ files using a custom PERL script, and then assembled into contigs using SOAPdenovo-Trans v 1.0.3 [78] (asm-flags = 0; max_rd_len = 301; map_len = 75; avg_ins = 700; kmer (−K = 127)). Trimmed reads were also assembled with Trinity [79] using default parameters, except for adjustment for library insert length (−-group_pairs_distance = 700) and minimum read overlap (−-path_reinforcement_distance = 75). The complexity of SOAPdenovo-Trans and Trinity assemblies were reduced by clustering allelic variants using CD-HIT-EST [80] with default parameters, except for change of sequence identity (−c 0.95), word length (−n 10), and length of throw-away sequence (−l 11). The relative completeness of each clustered *D. v. virgifera* transcriptome assembly was evaluated by comparison with the universal single-copy orthologs from Arthropoda obtained from OrthoDB v 9 [81] using BUSCO v 3 [82] (*E*-value cutoff 0.001). Full- and partial-length open reading frames and corresponding derived amino-acid sequences were predicted from the resulting SOAPdenovo-Trans clusters with TransDecoder v3.0.0 [83] using a minimum length of 100 amino acids.

The transcript sequences assembled by SeqMan NGen® (DNASTAR, Madison, WI) were imported into Blast2GO v4.0 [84, 85] and annotations acquired via BLASTx [86] comparison to the non-redundant (nr) arthropod-specific protein database at the National Center of Biotechnology Information (NCBI). The combined graphs were created at level 2 for Biological Process (P), Cellular Component (C), and Molecular Function (F) categories from Blast2GO.

### Bioinformatic analysis of the *D. v. virgifera* ABC transporter family

A searchable database was created from the combined DNASTAR *D. v. virgifera* transcript assembly, and subsequently searched with the set of deduced *T. castaneum* ABC transporter amino-acid sequences [30, 32] as queries using the tBLASTn algorithm in BlastStation software (TM Software Inc., Arcadia, CA, USA). Homologous sequences were selected based on sequence identity and *E*-value (< 10^− 6^). Putative *D. v. virgifera* ABC sequences were then used as BLASTx queries of the non-redundant NCBI protein database using the web blast interface (https://blast.ncbi.nlm.nih.gov/Blast.cgi) to confirm their identity as insect ABC genes; those that appeared to not be of non-insect origin, or were otherwise not ABC genes, were discarded. The number and positions of transmembrane domains were assessed via query of the NCBI Conserved Domain Database [87]. Finally, each *D. v. virgifera* ABC gene was putatively assigned to a subfamily (A-H) based on greatest similarity assigned to orthologs within BLASTx results. This BLAST search procedure was analogously repeated for SOAPdenovo-Trans and Trinity assemblies. The complexity of each ABC gene set was reduced by clustering allelic variants (sequence) across assemblies, and a comprehensive non-redundant set of putative *D. v. virgifera* ABC transporter contigs were generated (Additional file 7). Assembly of origin is denoted in sequence names as follows: DNASTAR (D), Trinity (T), and SOAPdenovo-Trans (S = “scaffold” and C = “contig”) within the FASTA files. The full translation product of each contig can be found in Additional file 8.

Phylogenetic relationships among derived *D. v. virgifera* ABC transporter protein sequences were reconstructed from the conserved NBD. A multiple sequence alignment was performed with MUSCLE using MEGAX [88] (default parameters) and used within a subsequent phylogenetic analysis. The unrooted Maximum Likelihood phylogenetic trees were constructed in the MEGAX program using default parameters in all categories except: LG model of amino-acid substitution with Gamma distributed substitution rates (based on Best Model determination within the MEGA program), Partial Deletion treatment of gaps/missing data, and 1000 bootstrap replicates [89]. ABC transporter subfamilies were assigned to *D. v. virgifera* sequences and clades within this phylogenetic analysis by comparison to similarities from our BLASTx search results and tree topologies among nearest orthologous gene family members in *T. castaneum* [30, 32]*,* and *D. melanogaster.* Multiple sequence alignments were generated as described above, wherein the deduced *D. v. virgifera* amino-acid sequences included full-length sequences when possible, but some were incomplete partial-protein sequences. All phylogenetic reconstruction methods were performed as described above.

### Gene expression across developmental stages

Preliminary analysis to estimate the relative expression levels for eight transcripts (*DvvABCA_50718, DvvABCB_39715, DvvABCE_2830, DvvABCF_2701, DvvABCG_3712, DvvABCG_14042, Dvvw* and *DvvABCH_5118*) across growth stages was made via semi-quantitative PCR in order to ensure dsRNA injections would be performed prior to the time of corresponding peak expression. Total RNA was extracted from each developmental stage [embryo (E), larval (L), pupal (P), and adult male (M) and female (F)], from which cDNA was reverse transcribed using the Superscript™ III First-Strand Synthesis System (Invitrogen, Carlsbad, CA, USA) using an anchored poly(T) primer. These cDNA pools were then used individually as template in eight separate PCR reactions each using *D. v. virgifera* ABC transporter transcript-specific primer pairs (Additional file 9: Table S3). Primers for the *D. v. virgifera ribosomal protein S6*, *DvvRPS6*, were used as an external control. PCR reactions were set up using MyTaq™ DNA polymerase according to manufacturer instructions (Bioline, Memphis, TN, USA), and subsequent amplification reactions were performed in a C1000 Thermal Cycler (Bio-Rad Laboratories Inc., Hercules, CA, USA) with the following cycling conditions: (95 °C for 3 min), 25× (95 °C for 30s, 58 °C for 30s, 72 °C for 10s), (4 min incubation at 72 °C). Amplification products were then visualized and compared using 1.5% agarose gel electrophoresis.

### RNAi knockdown phenotypes

Primers were designed for the generation of dsRNA using Vector NTI Advance (VNTI) software (Invitrogen), for all ABC genes whose orthologs are known to produce obvious RNAi phenotypes in *T. castaneum* [30]. These primer sets targeted regions that encoded transcript-specific TMD domains; this was done in order to potentially reduce unintended off-target effects by avoiding the more conserved NBD domains. Partial cDNAs were amplified for the 8 genes (*DvvABCA_50718, DvvABCB_39715, DvvABCE_2830, DvvABCF_2701, DvvABCG_3712, DvvABCG_14042, Dvvw* and *DvvABCH_5118*), as described above for developmental stage expression. Nested PCR was performed with an initial denaturation of 95 °C for 3 min, 35 cycles at 95 °C for 30s, 58 °C for 30s, and 72 °C for 10s, and then a 4 min incubation at 72 °C on a C1000 Thermal Cycler (Bio-Rad). PCR products were purified using the QIAquick PCR Purification Kit (Qiagen) according to the manufacturer’s instructions, ligated into the pGEM-T vector (Promega, Madison, WI, USA), and the resulting plasmids were used to transform TOP10 competent *E. coli* (Invitrogen). All positive clones were cultured in a selective LB medium containing 100 mg ampicillin L^− 1^. The recombinant plasmid DNAs were isolated using the QIAprep® Spin Miniprep Kit (Qiagen), and the inserts were Sanger sequenced and confirmed by use as BLASTn queries (https://blast.ncbi.nlm.nih.gov/Blast.cgi). Purified plasmids with each cloned ABC transporter were used as template in separate PCR reactions primed with the following primers: T7 as a forward primer (due to location in pGEM), and a pGEM-specific reverse primer that was tailed with T7. This enabled all amplification reactions to be performed using the same set of primers under conditions described above. PCR products were analyzed by 1.5% agarose gel electrophoresis, purified using the QIAquick PCR Purification Kit (Qiagen), and then ~ 1 μg of each was used as template for dsRNA synthesis using the MEGAscript T7 in vitro Transcription Kit (Ambion, Austin, TX, USA). Each of the synthesized dsRNAs were purified using the MEGAclear Kit (Ambion) and concentration determined using a Nanodrop 1000 (Thermo Scientific, Waltham, MA, USA) using the single-stranded RNA setting.

RNAi assays were conducted by injecting dsRNA corresponding to each of the 8 specific *D. v. virgifera* ABC genes individually into the hemocoel of third-instar larvae, pre-pupae and/or newly-eclosed female adults. Before microinjection, experimental insects were anesthetized on ice for 30 min, then injected with ~ 0.2 μl of a gene-specific dsRNA at a concentration of 1-2 μg/μl. Each treatment was replicated three times, with ≥20 individuals in each replicate. Following injection, larvae and pre-pupae were allowed to recover at room temperature for 1 hour, and then moved to germinated corn for further monitoring and phenotypic analysis. Phenotypes were observed daily using a stereomicroscope, and transcript levels assessed at 5 days post-injection by semi-quantitative PCR using RNA isolated from pools of injected individuals (one individual per replicate, for a total of three individuals per PCR reaction).

Treated females were kept in an oviposition chamber (agar plate with cheese cloth) and maintained on an artificial diet. At 2 days post-injection, females were mated to untreated males, and generally started to lay eggs ~ 10 days later. To determine egg viability, eggs were harvested from the oviposition chamber and placed on moistened filter paper in Petri dishes and held at 26 °C, 70% relative humidity with an L14:D10 photoperiod. Females were allowed to lay eggs over a two-week period, and eggs were counted every other day to assess the rate of egg laying. Hatch rate counts were made every other day, beginning 10 days after the first egg lay (22-days post-injection) and continuing for 4 weeks until no further hatching was observed.

## Supplementary information

**Additional file 1:** **Table S1.** Comparisons among *D. v. virgifera* reference transcriptome assemblies based upon total assembly output, number of transcript clusters, predicted open reading frames (ORFs), and benchmarking of single-copy orthologs (BUSCOs; Arthropoda v 9 reference set).

**Additional file 2:** **Figure S1.** Blast2GO annotation results for the combined *D. v. virgifera* transcriptome.

**Additional file 3:** **Figure S2.** Top-Hits species distribution from Blast2GO.

**Additional file 4:** **Table S2.** *D. v. virgifera* ABC naming chart.

**Additional file 5:** **Figure S3.** Multispecies ABC Protein Phylogeny.

**Additional file 6:** **Figure S4.** Effect of *DvvABCG_3712* RNAi on egg hatching.

**Additional file 7.** Nucleotide Sequences of Dvv ATP binding cassette (ABC) transporters

**Additional file 8: ** Amino Acid Sequences of *D. v. virgifera* ABC transporters.

**Additional file 9:** **Table S3.** Primer sequences used for dsRNA synthesis and RT-PCR analysis.

## Data Availability

The datasets generated and/or analyzed during the current study are included in this published article [and its supplementary information files] or are available at NCBI under BioProject PRJNA490283, with SRA database accessions SRX4669438 to SRX4669441 for the raw reads, and TSA database accessions GHNH00000000 and GHRK00000000 for the Trinity and DNASTAR assemblies, respectively.

## Abbreviations

ABC: ATP-binding cassette
Bt: *Bacillus thuringiensis*
GO: Gene ontology
NBD: Nucleotide-binding domain
NCBI: National Center of Biotechnology Information
ORFs: Open reading frames
TMD: Transmembrane domain
