# Supplementary material for: Structural and functional insights into the Diabrotica virgifera virgifera ATP-binding cassette transporter gene family
Source: BMC Genomics. 2019 Nov 27;20:899. doi: 10.1186/s12864-019-6218-8 (PMC6882327; doi:10.1186/s12864-019-6218-8)
Supplement: Supplementary file 1 — Additional file 1: Table S1. Comparisons among D. v. virgifera reference transcriptome assemblies based upon total assembly output, number of transcript clusters, predicted open reading frames (ORFs), and benchmarking of single-copy orthologs (BUSCOs; Arthropoda v 9 reference set). [file 12864_2019_6218_MOESM1_ESM.docx]

**Table S1:** Comparisons among *Diabrotica virgifera virgifera* reference transcriptome assemblies based upon total assembly output, number of transcript clusters, predicted open reading frames (ORFs), and benchmarking of single-copy orthologs (BUSCOs; Arthropoda v 9 reference set).

|  | DNASTAR | SOAPdenovo-trans | Trinity |
| --- | --- | --- | --- |
| Assembly output | Contigs | Contigs | Components |
| contigs/scaffolds |  |  |  |
| Total nucleotides | 48,787,149 | 77,045,998 | 126,689,979 |
| Count | 25,296 | 133,180 | 162,897 |
| Singletons | 25,296 | 133,180 | 162,897 |
| Longest/shortest | 27,253bp/492bp | 15,650bp/128bp | 29,515-bp /201bp |
| N50 | 1,604 | 439bp | 427bp |
|  |  |  |  |
| CD-HIT-EST clusters |  |  |  |
| Total nucleotides | 47,927,778 | 69,085,871 | 105,965,115 |
| Count | 24,405 | 106,139 | 151,844 |
| Longest/shortest | 27,253bp/492bp | 15,650bp/128bp | 29,515bp/201bp |
|  |  |  |  |
| TransDecoder ORFs |  |  |  |
| Total nucleotides | 22,112,229 | 26,984,979 | 41,026,530 |
| Count | 18,305 | 37,533 | 40,087 |
| Longest/shortest | 26,526/297bp | 15,195bp/300bp | 29,484bp/300bp |
|  |  |  |  |
| BUSCOs (count/proportion of 1066) | |  |  |
| Complete | 928/0.871 | 862/0.809 | 1,047/0.982 |
| Single copy | 906/0.850 | 823/0.772 | 934/0.874 |
| Duplicated | 22/0.021 | 39/0.037 | 113/0.106 |
| Fragmented | 10/0.090 | 153/0.144 | 9/0.008 |
| Missing | 128/0.120 | 51/0.047 | 10/0.010 |
