# Supplementary material for: Structural and functional insights into the Diabrotica virgifera virgifera ATP-binding cassette transporter gene family
Source: BMC Genomics. 2019 Nov 27;20:899. doi: 10.1186/s12864-019-6218-8 (PMC6882327; doi:10.1186/s12864-019-6218-8)
Supplement: Supplementary file 2 — Additional file 2: Figure S1. Blast2GO annotation results for the combined D. v. virgifera transcriptome. [file 12864_2019_6218_MOESM2_ESM.docx]

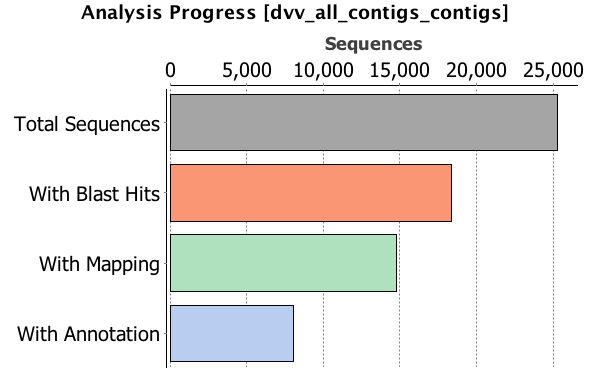
**Figure S1:** **Blast2GO annotation results for the combined *D. v. virgifera* transcriptome.** Among the 25,296 contigs used for the BLASTx search against the NCBI nr protein database, BLASTx analysis revealed over 18,343 contigs shared significant identity with known arthropod proteins.
