# Supplementary material for: Structural and functional insights into the Diabrotica virgifera virgifera ATP-binding cassette transporter gene family
Source: BMC Genomics. 2019 Nov 27;20:899. doi: 10.1186/s12864-019-6218-8 (PMC6882327; doi:10.1186/s12864-019-6218-8)
Supplement: Supplementary file 3 — Additional file 3: Figure S2. Top-Hits species distribution from Blast2GO. [file 12864_2019_6218_MOESM3_ESM.docx]

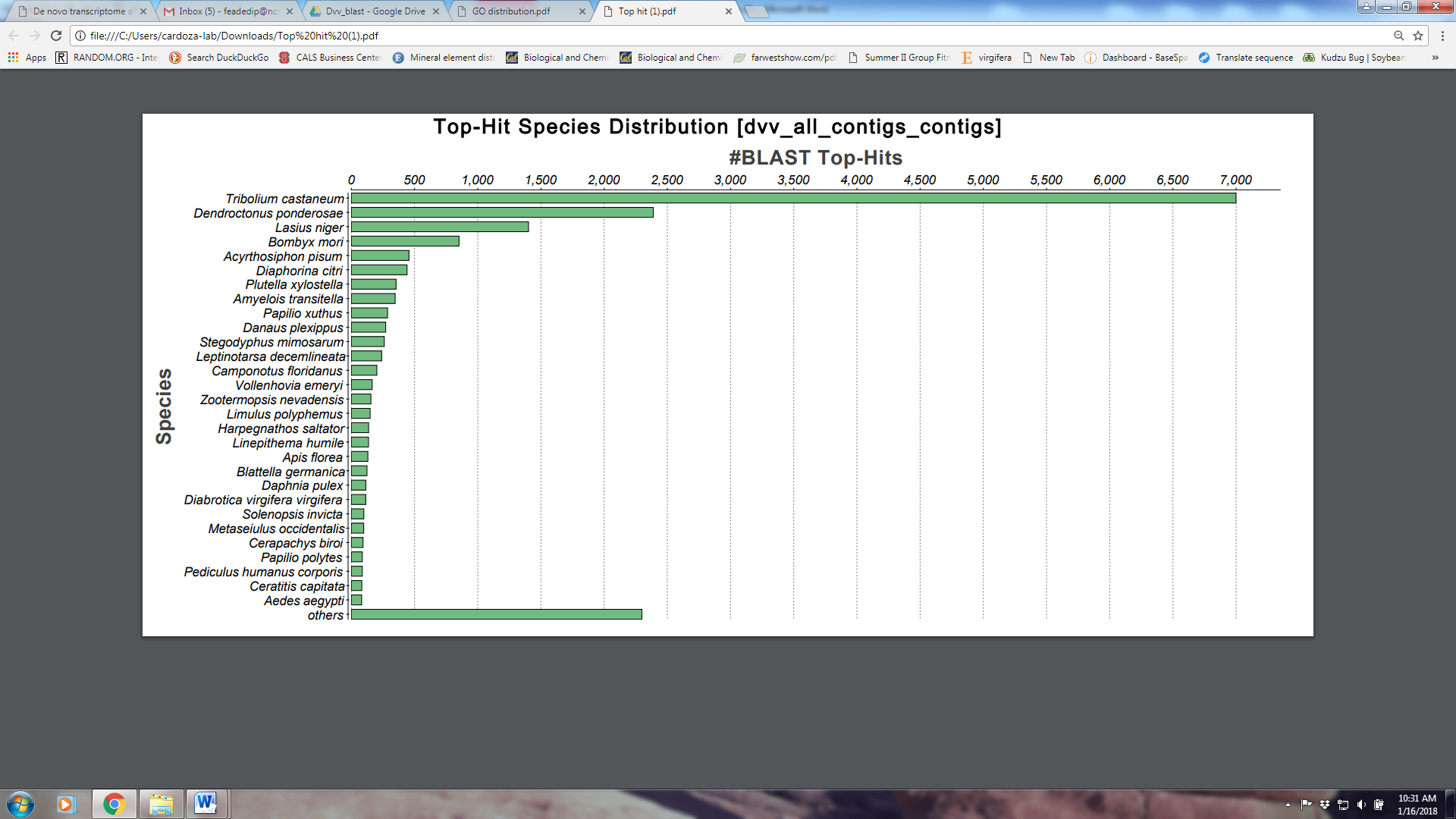
**Figure S2: Top-Hits species distribution from Blast2GO.** The most represented species in the top scoring BLASTx-matches the *D. v. virgifera* contigs when compared to an arthropod-specific database.
