# Supplementary material for: Structural and functional insights into the Diabrotica virgifera virgifera ATP-binding cassette transporter gene family
Source: BMC Genomics. 2019 Nov 27;20:899. doi: 10.1186/s12864-019-6218-8 (PMC6882327; doi:10.1186/s12864-019-6218-8)
Supplement: Supplementary file 4 — Additional file 4: Table S2. D. v. virgifera ABC naming chart. [file 12864_2019_6218_MOESM4_ESM.docx]

**Table S2:** DvvABC naming chart

| **Subfamily** | ***Dvv* gene name** | **Contig numbers** |
| --- | --- | --- |
| A | *DvvABCA_49125* | DvvABC-A T49125_c1_seq2 |
| A | *DvvABCA_50718* | DvvABC-A T50718_c0_seq1 |
| A | *DvvABCA_18330* | DvvABC-A D18330 |
| A | *DvvABCA_266167* | DvvABC-A C266167_7.0 |
| B | *DvvABCB_21313* | DvvABCB_D21313+T40801_c0_seq1 |
| B | *DvvABCB_17742* | DvvABC-B D17742 |
| B | *DvvABCB_19147* | DvvABC-B D19147 |
| B | *DvvABCB_39715* | D vvABC-B T39715_c0_seq2 |
| B | *DvvABCB_9796* | DvvABC-B D9796 |
| B | *DvvABCB_13664** | DvvABC-B D13664+T8676_c0_seq1* |
| B | *DvvABCB_17837* | DvvABC-B T17837_c0_seq2 |
| C | *DvvABCC_41801* | DvvABC-C T41801_c0_seq1 |
| C | *DvvABCC_44708* | DvvABC-C T44708_c0_seq1 |
| C | *DvvABCC_48952** | DvvABC-C T48952_c0_seq2+T48952_c0_seq1* |
| C | *DvvABCC_17573* | DvvABC-C T17573_c0_seq1 |
| C | *DvvABCC_51687* | DvvABC-C T51687_c0_seq1 |
| C | *DvvABCC_21020** | DvvABC-C S21020Locus_43217_0+T47823_c0_seq1* |
| C | *DvvABCC_222633** | DvvABC-C C222633_3.0+T22098_c0_seq1* |
| C | *DvvABCC_18126* | DvvABC-C D18126+D19164 |
| C | *DvvABCC_49513* | DvvABC-C T49513_c0_seq6 |
| C | *DvvABCC_14070* | DvvABC-C D14070 |
| C | *DvvABCC_22628* | DvvABC-C D22628 |
| C | *DvvABCC_20002* | DvvABC-C D20002 |
| C | *DvvABCC_7536* | DvvABC-C D7536 |
| C | *DvvABCC_47333* | DvvABC-C T47333_c0_seq11 |
| C | *DvvABCC_49618** | Dvv ABC-C T49618_c0_seq2+T79857_c0_seq1* |
| C | *DvvABCC_45163* | DvvABC-C T45163_c0_seq1 |
| C | *DvvABCC_43960** | DvvABC-C T43960_c1_seq1+D21270+T49513_c0_seq3* |
| C | *DvvABCC_48940* | DvvABC-C T48940_c0_seq1+D21306 |

(*) Incomplete sequences

**Table S2 cont:** DvvABC naming chart continued

| **Subfamily** | ***Dvv* gene name** | **Contig numbers** |
| --- | --- | --- |
| C | *DvvABCC_217405** | DvvABC-C C217405_3.0+D12550+S19317Locus_19671_1* |
| C | *DvvABCC_10132** | DvvABC-C T10132_c0_seq1+D20603+T57212_c0_seq1* |
| C | *DvvABCC_48300** | DvvABC-C T48300_c0_seq1+D21892* |
| C | *DvvABCC_47673* | DvvABC-C T47673_c0_seq1 |
| C | *DvvABCC_5345* | Dvv ABC-C D5345 |
| C | *DvvABCC_22413* | DvvABC-C D22413+T49258_c0_seq1 |
| C | *DvvABCC_18709** | DvvABC-C D18709* |
| C | *DvvABCC_21941* | DvvABC-C D21941 |
| C | *DvvABCC_15305* | DvvABC-C D15305 |
| C | *DvvABCC_12562* | Dvv ABC-C D12562+D20321 |
| C | *DvvABCC_10642* | DvvABC-C D10642 |
| C | *DvvABCC_41602* | DvvABC-C T41602_c0_seq1 |
| C | *DvvABCC_12703* | DvvABC-C D12703 |
| C | *DvvABCC_14968* | DvvABC-C D14968 |
| D | *DvvBCD_11014* | DvvABC-D D11014 |
| D | *DvvABCD_11628* | DvvABC-D D11628 |
| E | *DvvABCE_2830* | DvvABC-E D2830 |
| F | *DvvABCF_2701* | DvvABC-F D2701 |
| F | *Dvv BCF_802* | DvvABC-F D802 |
| F | *DvvABCF_9935* | DvvABC-F D9935 |
| G | *DvvABCG_9811* | DvvABC-G D9811 |
| G | *DvvABCG_3712* | DvvABC-G D3712 |
| G | *DvvABCG_14042* | DvvABC-G D14042 |
| G | *DvvABCG_10897* | DvvABC-G D10897 |
| G | *DvvABCG_22358* | DvvABC-G D22358 |
| G | *DvvABCG_23081* | DvvABC-G D23081 |
| G | *DvvABCG_13051* | DvvABC-G D13051 |
| G | *DvvABCG_38769* | DvvABC-G T38769_c0_seq1 |

(*) Incomplete sequences

**Table S2 cont:** DvvABC naming chart continued

| **Subfamily** | ***Dvv* name** | **Contig numbers** |
| --- | --- | --- |
| **G** | *DvvABCG_13829* | DvvABC-G D13829 |
| G | *DvvABCG_49457* | DvvABC-G T49457_c0_seq1 |
| G | *DvvABCG_36869* | DvvABC-G T36869_c0_seq1 |
| G | *DvvABCG_79525* | DvvABC-G T79525_c0_seq1 |
| H | *DvvABCH_20789* | DvvABC-H D20789 |
| H | *DvvABCH_5118* | DvvABC-H D5118 |
| H | *DvvABCH_18290* | DvvABC-H D18290 |
| H | *DvvABCH_11818* | DvvABC-H D11818 |

(*) Incomplete sequences.
