# Supplementary material for: Structural and functional insights into the Diabrotica virgifera virgifera ATP-binding cassette transporter gene family
Source: BMC Genomics. 2019 Nov 27;20:899. doi: 10.1186/s12864-019-6218-8 (PMC6882327; doi:10.1186/s12864-019-6218-8)
Supplement: Supplementary file 6 — Additional file 6: Figure S4. Effect of DvvABCG_3712 RNAi on egg hatching. [file 12864_2019_6218_MOESM6_ESM.docx]

**Figure S4: Effect of *DvvABCG_3712* RNAi on egg hatching.** Eggs laid by females treated with *DvvABCG-4C* dsRNA failed to hatch, compared to those laid by buffer-injected control females which hatched successfully. The eggs laid within a period of two weeks were counted every other day.
