# Supplementary material for: Structural and functional insights into the Diabrotica virgifera virgifera ATP-binding cassette transporter gene family
Source: BMC Genomics. 2019 Nov 27;20:899. doi: 10.1186/s12864-019-6218-8 (PMC6882327; doi:10.1186/s12864-019-6218-8)
Supplement: Supplementary file 7 — Additional file 7. Nucleotide Sequences of Dvv ATP binding cassette (ABC) transporters [file 12864_2019_6218_MOESM7_ESM.docx]

**Additional file 7:**

**Nucleotide Sequences of Dvv ATP binding cassette (ABC) transporters**

>Dvv ABC-A T49125_c1_seq2

CAATAAACGCTCATACAAACTTTCAAAACAACCAGTACAGTACCCCTCACTCCAGAAACCCCCAAAAAAACTATATGAATTTCGCACCTTTCCACCAGGATAGCTCCTTAATTAAAAATTATTGAATATAGAAAACAACTTGCAAACTGAAAGATGGAAATCTTAAATAAAAAATTATTTTGAAGTGCGTGGTTTGTCCAAATATAATTTAAAAAAATGCGATGCCACAAAGAAGTTTAAAACAATATTTGGTTTCTCAACTACACAGGTGTCCAGTCTGTGTGCTTACTTGTTTTGTTAAATGTAACTGTTTGCAAGAATAAATATTAAATGTTACCAAAAAAATAAAAAATCACTGTATTCTACTATTTCTTTTTGGTAACATCTTCTTCATCTTCTCTGGCAACTTTCAAAAAGACGTCTTCTAACGAAGCTTCACTGATTGCATAATCTTCAATTAAATGCGAATTGTGTGTTTTGAGTGCCTCCACTTCTTGGAAAATATCGCTCCATTTTTTAGTTTTATCCTTAATGTAAACATGGAGGAGACCTGAATGTTCATCTTTGATTTCACCGCGATCATGATTCGTAAAATAGGATTTAAGATCATCGACACTTTTAAATTTGTTACTTATTACTCCATCTACCTCGTCTACGTCATCATCTACCAGATTTATAGGCTGACCTTGATTAGACACAAGTTTTAATTTTAATGTAAATCCACCATATCTATTCTTTAAGTCCACCAATCTTCCTTCTTCAACAAACGAACCTTTCTTCATTATTTTCAGCTCGTCGCATAAAGCTTCGCATTCAGTCATACTATGAGATGTTAAAATGAATCCAATCTTGTTTCCACTATTGCCTTTCATGAGTTTAATCAGATCCCAACACTTCCTTCTAGTAGCCGGATCTACGCCGTTGGTGGGCTCGTCCAATAATACAAACTTACGGTTACCTATAAGGGATATGGCCAAGCTTAGTTTTCTTTTGTTTCCTCCACTATAATGTCCGCACGGGATATCCGCATATTGTTTTAAGTCAAAAGCTTCCAAAAAGCGATCAATCATTGTTTCATCAGTAACACCTCGTAACTTTGCCACGGTACTTAGTAGTTGTCTGCCAGTAAGGACAAAGTTTAGCGAATCTGACTGAGGACAATAACCTATTGTCTCCAAATACTCGCTTCCTGTGATGTCCAGAGGTTGTTTATTATTGTCTAATTCGATCTTAATTTCTCCATCATCAACCACTTCCTCTCTTGTAAGCATTTTAAAGGTTGTAGTTTTACCAGCCCCATTTACACCTAATATACCCAAACAGTGACCGTGCTTAAGAGTGAAATTTATATTTTTAACTATCTGTTTTCCGGCATATAATTTCATTATTTTCTTGGCTCTTAAAGTATTGTAGCCGTCACCAACATCTTCGTTAACATATTTGTCATTTTTGGGGATTAGTGCTGTATCTTTGTCTTTGAATGATTTCAAAATAAGATTACATTGACGCATAACCATAGCTTTTAATTTCTTCATAAAATATGTGTCTAGAATAATGTTTATCGTTAGATAAAAAACTCCGCATCCTATCATAAACATGTAATGTTCGCTGAATAAGTTGTTGTAGCTCTTATAATTTTGACATTCGATTGAACTTTCAACGCAGCAAGCGTGTGACTCCATCTGATTACACATAACTCTCAAACGTTGGGGAGATATTTTGAAATTGTAAATATTAACTGTGCGTCTAATAAACGAGACCAATGCATCTGATAATGAAACTTGCGGTAATAAGAAGAAACACACATATTTAATTTTATTTCCAATATTTACGTAGTATTCATCTTGCGATTCTAGTAATACGGCTATGGTTAGTGTAAGAATGATGCCCAAGAACATCCCCATGATGACGAATAACGCAAAGGCACCAGATGCTGTCTTCTTTCGGCTGAATATATACGCAAAAGGTATCCCAGCAATACCATAACACAGAAATATGGAAAATAAATAACCGAGTTCGGTACCGTTATGTTGGAATACGGGAGAACATATCCACAGTAAGAAACTTAATATGAACATTAAAAATATGTAAAATGTCATATCGGCAATCCAATTAACAAGCCAATAGAAGAATGGTTTCACGCCAGCCATGTACTGTATTTGTAAAAATCTCGTCGATATTTCAGTATGTGGGAATATGATAAAAATGCCGTGAATAAAAAGACATCCCACCGGTAACAGGATGAGCCACAGAACAGCAACTTTTGCTTCCGAATACTCCTGTGATGAGAGTCGCTCTTGGACCTGCGGTAAAGGCCAATTACTGACAGATATTCCATATTCGGGACCTAATAACGTTTTAGCCAGCGAGTCGGTAATTAGATTTAAAGAAATCGGAGTACTGTGAGTAGCTATACCATTGTATAATGCTACTAATGTAAAATCACGATCATAAACGAAATTAGCTCCAGCAATCATATGTTCTTTATAGTAAGCTATGTTTTCCAGACTTCTTTGCAAAATAGATTTTTCAACGTTATCAACTTTGAATGGTAAACTTCTTTGCGATTCTACAGCTGACATGTAATATTTCATTAACGTATTAATTTTTTCGTTTGATGTGTCCCCGCTGTAGTAAACCGATGTTTGTTTGTAGCTGCTGAGTTTGAGGGGTAGTTCTGGTCCCCTTTCACTATAGTAGTCATTTCCAGAACTTAAAAATACCGTCAGAGAAAAAAGGACTACAGCTACACCCACAGGTACAATGTATGTTGACCACTTTTTTAGAGAGAAATTCACTTTCTTTTTGAGTAAAGTAATAAGAGTCAAAGAGGCATGAGGTTGGATGTCATCTTGTGTGGCCACTGATGAAGTGTCGTCTGATGAATGTTCAATCTCTTGGCGTGTTTTTAGGAATACATCTTCCAAAGTCGTTATCGTTATCGATATATTACTAAGGTGTAACTCGGATCGATTTTTCTCCAAATCTCCAAGAAGCCCCGTCATGTTGGTATTCTGATAGGGTAAAACAAATACCAAGTTGTTTCCATTATTTGACGTCAAATGCGCATCGGGCATAAAATGTTTAACTCTTCTACTAATGGCATCTATATCTGCGTCTTCTTCAATCATTAAATTTAAGTGGTAACCTGTATCGTATTTCTTTTTCAAAAACATTGGAGTACCATAACAGTTTAGACTACCGTCAGACATTATTGCTATCCAGTCTCCTAGTGCATCTGCTTCTTCCATAAAATGTGTGGTAATAAGGATTGTTTTCTCCCCTCGCCATTGCAACAATAGATCCCAAAGCTCTCTTCGAGATTGGGGATCCATTCCCGAAGAAGGTTCATCCAAAATTAATACCTTTGAATCACCGATAAGGGCCATTGCCAAGCATAGCTTTCTTTGCATACCACCTGAGAGTGTCTGAGCCATCGAATGCTCTTTATCGGGCATATTTAATTTTGCCAATAAACTTTTTGCTTCTATGTTGGCTTCTTTCGTAGATTTTCCTTTCAGCTTGGCGAAAAATAAGAGATGTTCTTTAACTGTCAAATCGGTAAACAGAAGATTATGTTGGGGACATAGTCCCAAACTCTTCCTGATTTCGTCCATGTCAGTATTAATATCCAATCCATTTATTGTCACCGAACCAGACGTAGCTGGAATCAGTCCTGTTATTATTGACATGGTGGTTGACTTCCCTGCTCCATTGTGACCAAGTAGTACAGTGATTTGATTTTTGTAAATGTCTAAATTCAGGTTATTGACAGCTTGTTTTTGTTTATACCTTTTATGAAGATGGTTAATTTGAATACCCTTAGCTAAATTGTGACCTGCTTCAACCTTCTCCAAAGGAACTGTTTCAGAATCTACTGGCACTGTGGTGATAGATGGTTTTCTACAGAGCTTCATAAAATTTTGTAAAGGGAATAATATACTCTTTCGGACCCCGTATTTACCAGGATTAACTCCATCCATATAAAATGTGAAAATGGTGTAGATAACAATATCGACTATTAACATCACATAAACGTTTAACATGGTGATATCATCTGATGATCCACTACCAGGCTTGAAGAAATTTGACCAATTGATGCCGACTTCTCTTTCTTCAAATACAGAAATGATGGAATATCCGTAATGAAGGGACATATTTGGTAGTAGGTTTAACAGAACGTTGGATAACCAAGACAATTTGTTTGCTTCGTCTAGTCCACATGCGTATTTTGGTATGAAAAATGATAAAATCCATACGAGAATTCCTGCGACTAATGCAATTGTGGGTCGGCTGAAAAACGAACTGATGGCAAAACAAAATGCTACTGCAGCCATACAATACAATATTAGAAACACGAATAGTATTCCGCCGTTGGCAAATTCTACAAGTGGAGAGTCAGACCCAAACATTGGTACTTTCATAAAAATAGATATCACACTAACGGCGAAAAACATCGGTATCATGGCATAAATAAACCAGCCAAACCATAACATCCAGGATTTCATTCCTACCATTTTCATTAGTTCCTTTGTTCCTGAATGTTTCTCTTCAACAACTCTCTTAAGTACCGCTGGACATAAAAATATAAAACTAAACAATGTGATCAGCGGCAAAAATTCCAAAAACAATGTTGTTATAGCAGAGTCTACTTTATGGGGAGGGTATGGAAACTCTTGTATTTGTATGGAGTAAGTGTCCTCTATATTGCTACTTCTCAAATTTTCCTCCAAGAATGACATGTCGAGGGCTAGTTGAATTTTCAAAAAAATCTTCATCCCGATAAACATACTCACTGCCGGGGCTATAACCGTAGACTCTTTGGTACATGCGATCGGTGACCAAAAACTCCGGCTGGTAGTAGTTTTTGTGATACCTTATCTTGTAGTTAAAATGTTTGGGGTTTTTGTCATCAAATATAATTGCAACTGTTGTATAGTTTGGATTTTTATAGTAGTATCCCAATAATTCATTTTCTGATCCAAACCTTTGTATATGATCACTAGGCATCCCCAACTTGGTTAACATTTTGGTAGTAATGTTGGTATAATATTCATTATAGGGTGTGTATAATATATACGTTCCAGCCACATCTATGTTATAATTTATACCATCTATCCTGCCATACGTGGGATTGGGAACTTCTATTTTGTTCAAGCCTGTGATTTGACTTCTGCCGTAGGCAATCAATAAAAATAGGCATACTGGCAGGAATGATTCTATAATTGTTAGAAACCAATGCCTTTTTCTTATTATGTAGTTTTTCCAAACAACGACTTCCAGTTGTAAAAGACTCATTGTTAAAACCAAAGAAAACACCCTCCTAGAAAACCATAATACAAAAATGGATTTTGTAGGAAATTTTTTTTTGATTTTTTATTTTAATGTTTATAACACATACACTTTCGAACTTTACATGACGCGATTTACAAAATAACTAATGATAATTATTGATAACGATAATGAACATGACAGAAATATAATTTATTTTTTTATG

>Dvv ABC-A T50718_c0_seq1

TGTTTTATTTATATTTCAATTATTATTCTAATTCTTCTTCTTAACATCGTCACCTTCTGGATTTTGGTGTTTTGTAAACGTTAGGAATACCTGTTCTAGACTACTTTGTCCCAAAGAATAGTCTTCAATATTCAGATCACTTCGCTTAGCTCTTTCTAATATACCAAACATAGTGGACCATGCCATGGAAGTGTCGGTTATATAATAATACAATAGTTCTTGATGTTTCTCTCTAAGATGAGCATAAGGAAATTTATCTTTGATATACTTCTCCAGTGACTCTGTATCTGCATGTACCAGACCACCGCTCTCTGGCAATTTCTTTAGTTTTATGGTTAAGGTGTATCCCTCCGCAAATTTGTTTTTAAGATGCTGTGTGGATCCAAGACACTTGAAATTGCCGTTCACCATAATGGCTATTCGAGTGCACAAGGCTTCACATTCTTCCATGCTGTGAGAAGTTAGAACGATGCACTTGCCGTTGTCTCGAATTTTGCATAATGAATCCCACAGGTAACGTTTCGTTGCTGGATCCATACCTGTTGTAGGTTCGTCCAAAAATAGTACTGGCGGATCCCCTATCAACGACAAAACAGTACTTAACTTCCTCTTATTTCCACCGCTCATCTCTTTAACTTTCTTGTCCAAATGACGATGAAAATCGAAGTCTCGAGACAAGAAATTCGCAATTCTTTGGGTTCTTTTAAACTCTATTCCTCTGAGTAGGCAGTACATGATAATCGTTTCCCTTGCTGTCATATCATCCAGCAAAGCGTCGAATTGAGGACAGTAGCCGATGTTCCGTTGTACTTGCTTCAGTTGAGTTTTTACACTCTTTCCTTCGATCCATGCATCACCGTAAGATACAGTTTCATCTCCGCTCATCATTTTAAATGTTGTAGTTTTTCCTGCACCATTAATACCCAACAAACCAAAGCACTCAGACTTTTCAATTCCAATAGACAACCCATTCACAGCTAAAAAGTTGTTGTAGTATTTGGTTAAATCTTTCACGACTAAAATGTTAGTTTGACGTATATCATGATCTGTGGCCATGTGAATCCTTTCTTTTTCTTTGGATACGTCGTCGTCTTCGTCTTGAATGAGAATTGGTTGTTTCGGGAAGAATTTTTGGTTGATGTAGTACATCAGTCTGGCTATGAATTTGTATTCGATGATAAATAGTAATATTGACCACAAGACACCAGAAATCAGTGAGTACGTTACATTTTTTGCGATACCATTATTGTTCCATGAAAAGTAGTTGTCGTTAAGATTGGTACAAATATCAGCGACAGTGGATGGAAATTTTGAAATGCACTCATCTTTAGTAAAATTATTTTGAGCACACGAGTCAAATACTGTTTCACAAAGAGTGTTGTATGAATAGATAGCGTAGCATTTGTTTATTCCTGTAGCTAAAGAATAGTGCGGAAACACAAGAAAAACGTTATCCAAGGTTTCTCCTATACGTTTTAGGTTCGTGCTTTGCGTTTTAAGCACTTCAACTAAAAGAAAAGCTGCATTCCCTATAAATATACCCACGAGAGTCATCCTTGTGTAACCCGTCGATGGAACTGTGAATATTAATGATGCTACATAATAAATTGGCATAAACGACCAACCAAACAAAAGTAATAGGAAAAATAGCCGACCCATATCTCCACTTGTCTTAAACCCATCTTGTTGGAAACAATATATAGTGATCAATAATACTGAACATACCAGTAAGTAGACAAACATATCACAAATTATATTAACAAACCAAAAAATGTAAACCTTCACTCCTGATACAAACTGCAGGTGTTTTGACTTGGATACGTTTTCTCTTATCAAGAATATAACATAAAATGATGAGATAAACGCCATACTGAATCCAATATTAAATGCAAACTGGAATCCCTGAGATTGTCCACCAACAATGTTGTCTATTTGGGTTGCAGCTTTAAAAGGTAATGGTTTGTTAACAAATTCAATATGTCCGTTAGTGTAGTGATTGTAAACGGAAGACAAAGCCATGCCTAAGGCAATACCAGGTGAGTGGTAAGGATCATTGTTAAACCATGCCGTAATAACAGGATGAATTGTCTTGATGATACCAAATTCAAATTCTTGCTCTGTGAAAGTTGATCCTATAATGTAACTTCTACGCACTACGTTTGGATTAGTTGCAGTCAGGTCTAACATTATACGGGTAATATTGTCAATATTTGTTGGAGACATTCCATATCCATTTAAAACACCGTAGAAGTTATTCGTATAATTTTGTGGATCGTCAAATCGCTCAATTAAAGTAATAGGGTCAGTGAATCTTGCTAAATTTAAAGGCATTGCCGGCAAAACAGAGGGAACACTTCCTGAAGGTACACTCATAACTATAATAACGTTTATCATAGGCAAAAATATCTGAATCGCCAACAAAACCCATGATCTAAAGGTTCCTATAATCTTCTTCATAAGCATTGCGACGAACTGATTGACAATCAAATGTAAACCACCAGTGTACGAGGGAGATAAAGATTCTGGACCTAAATCAGCAGAAGTGAGTATAGGATTTTTTTCTTTTTTGTCCTTTTTCTGTTTTTCATTGTAAATTTCTTCTTGTCCATGGTCTGCACCAACTTTCATGAAAACTTCTTCCATAGTGGTTAAAGAAATACCATAGCTCCTGATTCCAAGTTCATTTGATTCTTGTTCTATTTGTTTTAACATTGCTTCGAATACAGGAGCATTATTTTCAGATAACAAGTAAGTTAGTTCACTTCCAACATTGCTATGGATCTCTATCTCTGGTATAAACTTCTTCAGCAGTTCTGTAACTTTATGTGGATTGCAAGTTTTCGCTTTATCCATAATAAGACTGTATCCCGCACCGTACTTCTTCTTCAAGAAGAAGCTGGATCCACAGCATTGTAATTCTCCACCAGCCATGATTGCTATTCTATCTCCTAAAAGATCAGCTTCGTCCATAAAATGTGTACTGAGCAATATTGTTCGACCCTCTTTTTGTTTCTGTAATAATTCCCACAACACTCGTCTCGCTGATGGATCCATTCCAGCAGTAGGTTCGTCCAACATAACAACCTTCGAATTTCCACACAAGGCCATTCCAACACAAAGTTTTCTTTTCATTCCCCCTGATAGAGTACTGGATTTCTCCTTCCTCTTTGGCTCCAGATCAAGCAGCTTGACGTATTTATCTATTTCTGCTGTTATTTTGCTTTTAGACAAACCTTTGAGTTTGCTGAAGAAGTACAAGTGCTCTTCGACGGTGAGCTCGTCGAAAATGATGTTGTGTTGTGGACAAAGGCCCAAGCTATTCCTGACTCCTGCGATGTCTAACCGGATATCATGACCATTGATTTTTGCTGTGCCGTTTGTTGGGCTTATCATTCCTGTTAACATGGACATCGTCGTAGTTTTTCCAGCTCCGTTGTGACCCAACAAAACTGTGATTTGATTTTCAAACATATTTAAGCTTAGATTCCGTACAGCCGTTTTCTTGCCAAAAGCTTTTTTCAGATTGAATATCTGAATCCCAGGTCTAAGGTTTGGTTCGGTTTCAAAGAATTCATTTGCTGAATGATCATTATAGTTATTATAATCTGATACAGCTCCTTTTCCACACCAGTATTTCTTTGTGAATGGGAAGTACCATTTTTCAGCTACTCCATATTCTCCTGGAAATACTGCCTCTATGTAGAGAGCAATTAATAGGTAGATTATTGTGTTTATTATGATCATTATCCATATTAATCCCAGGCTCAAGGTGTCATCTGGGGTGTTTGGTTTAAACAGAGTGCTCCAGGTCACACCATCTCCAGTTCCTTCATACATAAGTACAACTTGAAATCCAAAAGCCATAGCAGTATTTGGCAACAAACTTGTTAATAGCTTAGTAGATAATGCAAGATCAGTGTAGTTATTCTGCATGAAGAGATAAGGAGCATAAGATATGAACCATGCCAAACCTGCCATAGTTGCTGCAGTATTTGCTTTGGAAAAGAAGACGCTCAGAGCAAAGCAAAAAGTGATCATAGAGCAACAATATAGCAGGAGAAATACAAACAAAACGAATGGGTCTGCATAGGTGAATACTGTGAATTCTGTATTCGAGTACCACCTTACTTTGAGAAGTACTACCATAAGTGCTACCGAAATTATAACAAAAATGAAGCACTTGACAAACCAGGCCAACCAATGTAGCCAATTAGGTAGTCCCATAATTTTCATTGATTCCTTTAACTGTTTTTCTTTTTCTGTGGTGATAGATTTGACTGTATTTATACAAGTGTAGACAAAACTAAGCATGACAATCATTCCAAGCATGCTCGTAAGTGCTGTTAGAAGTTGATCACTGAACCAACTTGCGAATGGAAAGCGTTGCATTTTTATGAGAGGAAATGGATTAGACTTGTCCATCAAAAAATTAAATATTTCCTGTGGATCTTCGTGTGTAATACCCTTATCTGCTAGAAGAAGAGCAGTAGTCACAAAATGTTGTACGGCCAGAAATCCTTCAGCAAAATAATTTGGTGCTGATCCAGTAGCATTGTCGGGGGATCTTGGACCAGAGCTTTGAAATACGGGGTACACTAAGTTGGTCCTCCAATTTTGACGTCCAATCGAATCTAATTGATACACTGTTTCTCCTGGAAATCTTATATTAACCTCTACATTGTCTCTATCCCCTCCAAACTCGATACCATAATAGGATGAGTTTCCAGCTAATGATATTCTTTCTAATTCGCCTGAGCTTATTACACGCTGAGTGTTGACTTTTGGTATTAACTTAAAGAAGCCTATTACATCGTCGTAGTATTTATCTTCTGGAGCAGTATATAATATTGTAAGGCTGAAATTGCTATCACCAGTTTGCATACTACTAAATAATGATGGTCCTGCTTGCACAGCATCTGGGTCATTACAAAAAAGCGGTAGGCTAAGGTTGAAGTTTCCCTCGAAAGCACAAAATGGCTTATATATTCTTTCATGTTCATATGTCGGATCCGACAAACTTCTGATGTAGACTAAAAGGAATGAAAAGAGAACAGGAGCGACAATTTCTATTATGGTTTGAATTGGTTTTCTATATTGCAGCAGCCAATTCTTCCACATTAACAACAAAAATTTATCCAATTTTTCTCCCATTATTCCTTTATTACCGGTATTAGTGCAATTTGATCAGATCTATTCACTCACTACACTTCAC

>Dvv ABC-A D18330

CTAAAACCATCAGTTCAAGTTCATCTTTGTGGTGGAACATGTATACAAGACAGTATTAAAGTTTATGTGATTATAAATGACATAATAGTTCCTATTATAATTTATGTGAAATCGGTAATCGGTAAATATAACAATGAGTGCGGAGTCTAATTCAAATAGAAAAAGAAATCATAGCTACACTGTACTGCAAGATGGATCTGACATTGGATTTCAAGAACCAAGAGGTGCCATGGCGGAGAACCTGGACAAGTTTATTCTTCTTATGTGGAAGAATTGGTTGCTTCAGTATAGAAAACCCGTACAAACTGCGGTAGAAATATTGGCGCCGGTTATCTTTTCCATCTTACTTGTAGTAATAAGAAGTCTATCGGATCCAGTTCGTCACGAAACTGTAGTGTATCCGCCCTTTTGTACCATTCCTATAGCTCTAAGAGAGAACAAAACTGGGATCACGATTTGCCCTTCTTATGATGGAGTACCTTATATGGACAGAAATCAATCAGATGGCGGAAACAGTTCTAACAATCCATTTGAGAAATTTGCACTTGTCTACTCACCGTCCAACCCTCCAATAGACCAAGTGATGAATTATTTTCGACTCGCTTTTAAAAATGTCGTGGCCTTGGAGAGCTCTCAGGCTCTGGAGAAATATTTCATAACAAATTCGTCCAACATTACATTCGCTGGTATACAGTTCGATGATTCTTATAAGACCTTAAAAAGTTTAGACGACGTTAAGCACCTACAAGTATCAATAAGATTTCCAGGAGAAACCAGGTTAAAGTTAGATCCTTTTAATTACAATAACTGGAGAACGAATCTCATTTTTCCAATATATCAGCAGCCAGGACCTCGACTTTATAACTTGATTACAGGAGCAGCTCCAAGTTATTACAGGGAAGGTTTTTTGGGCCTACAATATTACCTAACACTTAGTGTTTTACTGGCGAAGAGTAATGTAACTTATGCTACAGATGATTATCTGGATCTTATATCCTGGGTAATAGAAAACCAGTTTCCTTTAGTCAACATGCGAAGGTTCCCGAATGCACCATGGTACGAAGATATTCTGCTGACAGCTTTGAAGTCTCTGATTGGTATTATAATAATGTTAAGTTTTGTCTACACTTGTATTAATACTGTCAAGTCAATAACCACGGAAAAGGAAAAGCAACTTAAAGAATCCATGAAGATAATGGGTCTCCCAAACTGGTTGCACTGGACAGCATGGTTTGTGAAATGCTTCATGTTTCTTTTAATATCATCAGTATTGATGGTAATATTTTTAAAAGTAAGATGGTACACCAATACGAATTTCACTGTATTTACCAAAGCTGATCCGTTTGTTCTACTTTTATTTTTAATGTTTTACAACTGCGCCACTATTACTTTTTGCTTCGCACTTAGCGTCCTGTTTAACAAAGCTAATACTGCAGCTACTATAGCAGGTATGGTCTGGTTTTTATCATACTCACCATATTTATTTATGGCGAATGTATATGACACCTTAACGTTAACTTCGAAATTAGTAGCAAGTATTGGATCAAATACTGCCATGGCATTTGGTTTTCAAGTAATTCTGATGTACGAGGGAACTGGCGAAGGAATTCAGTGGAACAACATTTTTACACCAAACACCCCTGATGATTCCTTAACTTTAGGGTTAATTCTAATTATGTTAACGGTTGACTCAATTATGTACTTATTAATTGCATTATACATTGAGGCTCTTTTCCCCGGAGAATTCGGAGTTCCACAACCTTGGTATTTTCCATTCACTGCACAATATTGGTGTGGACACCCTATATACAGAGGTGTCGAAGATTTTGATAATGGTGCTATAAAAGGAGAATTTTTCGAAACTGAACCAGAAAACCTAAAACCAGGAATTCAAATCCGAAATCTCAAAAAAGTTTTTAACCAAAAAGTAGCTGTTAGAAATCTCTCTCTAAACATGTACGAAGACCAAATAACAGTACTTCTAGGACACAACGGTGCTGGTAAAACTACAACGATGTCGATGCTAACAGGAATGATCACACCAAACGGAGGTACTGCAAAGATTAGCGGGTATGATATAAGGACCGATATGGAAGGCGTAAGGAAAAGCTTAGGACTTTGTCCGCAGCACAATATTATCTTCGATGAGCTCACTGTGGCTGAGCATATTTACTTCTTTAGTAAGCTCAAAGGAATGAGAAAGGGTGAAATTAAGTCTGAGATAGCAAAATATGTAGATCTTCTCGAATTACAAGAAAAGAGAAACTCCAAAGCATCGACTCTCTCAGGCGGTATGAAAAGGAAACTCTGCGTGGGTGTAGCCCTTTGTGGTAATTCTAAAGTTGTTATGCTTGATGAACCAACTGCTGGTATGGATCCTGCTGCAAGAAGAGCTCTCTGGAATTTGCTAGAAACACAGAAAGATGGACGTACTATACTTCTTACAACTCATTTTATGGACGAAGCTGACATCCTTGGTGATCGTATTGCAATTATGGCTGGTGGACAGCTGCAATGTTGTGGATCAAGTTTTTTCCTCAAGAAAAAATATGGTGCTGGTTACAGTCTTATTATGGACAAGTCACAAGAATGTGACCCTAGACGAGTTACACAGCTGTTGAAAAAGTATATACCAGACATTGAGATAAATAGTAACGTAGGTTCAGAATTGACGTATCTGCTGGTAGATGATCATGTCCATGTATTTGAACCTATGCTTAGAGAACTTGAAACTGAATCCGAGATGTTGGGAATTAGAAGCTATGGTATTTCGCTGACGACACTAGAAGAAGTATTTATGAAAGTTGGAGCAGACCATGGTCAAGAGGAAATGTACAATCATGAACATGGCAACATTGTTCAGAATGGGACTGCACAAAATGGATTTACCAATGGGATAAACGGGACACACAAAACTAACAATGGTACTCACACCATGGTTCCGACATATTCAAACGGATTCTCTCTATTACTGAACCAGATTATAGCCATGTTATTGAAAAAGTTCGTTTCAACTGTCCGCTCGTGGATCTTACTGGGCATACAAGTAATGATGCCCACTCTGTTTCTTATCATTGCCTTTGTGGTAGCCCGCAAAAACAAAATGACAGGAAATCTTCCTGCCATGCCTCTAAGCTTAAGTAAATTTGAGAACCCTGTGACTTTAGTAGAAAATGGGACATCGGATTATTTGCCTTACTACATGAAAGTTTTAGAGGATTATGGATACCCTGCAACCATCGTAGATAATATTACTTCAGTATTATTGGATAAGACAGAACATCACCCAATTCTCGTTACTAGAAGATACCAAGCAGCAGCAACGTTCGGCGAATCTGGAATACCTGATCTACCATTTAACCTCCCGAATTTGACAGCTTGGTTCAATAACAACCCCTTTCACTCACCAGCGGTATCTTTGTCTTTGATGCTAAATAGCATTTACCGAAAACTAGGCGGTTGTGATGACTGTACTATAGAGTTTACCAACAGTCCTCTACCTTACAGCGCCGCGACGCAAGCGTCGCAGTTGTTGACAGTACAAAACATTGGATTCCAGTTGTCGTTTAATATAGGATTTAGTATGTCATTTGTTGCTTCCTTCTATGTTTTATTTGTTATTAGGGAGAATCGTTGTAAGTCTAAGCATCTGCAGTTTGTGTCAGGAGTGAAAGTATACGTCTTTTGGTTAACTGCAGCCTTCTGTGATATGTTGACGTATCTGTTTACAGTGTTTGTACTGATGATCACTATGGTTATGTTCCAAGAAGATGGGTTTAAGTCAGGGAGTGATATAAGTCGAATGTTTTTCATCCTGTTTTACTTCGGATGGGCATTTTTACCAATGTTTTACCTAAGTTCGTATTTCTTTCAAGTTCCTTCTACGGGTTACACTAGAATGACGCTTGTTAGTATCTTTGGAGGAAACGCTGCTTTCCTTGTGGTACAAGTCCTTCAAAGTCCAGGATTAGACCTGCAATATATAGGAAATGCTTTGCATTGGCTGTTCCTTATATTTCCGCATTACTCGTTGGCAACTGGAATAAATGAAAGTTTCAAAGTGTATGCTTACAATAACATCTGCGCAAACCTTCTTAAAACGTGCGAGGAACAGCACATTCCGAAGAAAACTTGTATATCGATAATGCGTAATGAAAGAATTCGTGAAATTTGTGAAGACGCTGATCTGAATTACTTTAAATGGAAGGCACCCGGAATTGCCAGGAACATGGTTTATTCATTTTTAACAGGCATTATTTTATTCGCGCTTCTTTTGGCCATTGAGTATAAGATTTTCTCTAGAGTACACTATTACTTAACTCAAAAGCATTTTACCAAAAAACCTATACCTGTCGAAGACGAAGATTCTGACGTTTCGAAGGAACGAGAAAGAATACATGTTGCTACAGAGATTGACATTAAACAAAATTATACGTTGGCGGTCAAAGATTTGACTAAGTACTATAAGAACTTTTTGGCTGTGAACGGGCTGTGCGTTGGAATAAGAAAGTTCGAATGCTTTGGATTGCTTGGAATCAACGGTGCTGGAAAAACTAGTACCTTCATGATGATGACTGGTGATACTAGTATTTCATATGGTGACGCCTGGGTAAATGGTAAAAGTATTAAACAACATTTGGAAGAAGTTCAAAAAATAATCGGATATTGTCCCCAATTTGATGCTTTACTGGACGACATGACAGCAGAAGAGTCGATTATAATGTTTGCTATGCTAAGAGGTTTACCTTTCAAAGATACTTTTAAATTAGCTGATTATTTGTCTAAAGAATTCGATTTTACCAGACATTTGAAAAAGAAAGTGAAAGAACTAAGCGGTGGCAACAAGAGGAAATTAAGTACAGCGATTGCGTTGATTGGGGATCCTCCTTTGTTGTATCTAGATGAACCAACCACAGGAATGGACCCTGCCACCAAACGTCACCTCTGGAATGCCTTATGCAAAATCCGAGACAGAGGTAAATGTATAGTTCTCACATCGCACAGCATGGAAGAATGCGAAGCTCTCTGCACCCGAATAGCCATAATGGTAAACGGCAACTTCAAATGTCTCGGGTCCACGCAGCATCTCAAACACAAATTCGCAGAAGGGTACACTTTGACGATCAAATTAAAGAAAATCGCGGAGAGTTCCAGCGAAGGCCTCTCAGAAACTGAACCTATAGAGAAGTTCATTTGCCAAAGGTTCCCAGGAGCCCAGCTTCGGGAGAGACACCAGGAGTTATTAAATTATTATATAACTAATAAGTCGGTGCCTTGGTCAAAGATGTTTGGCATCTTGGAGAAGGGAAAGAGAAGTGATCTCAATATAGAAGATTATTCATTAGGACAATGTAGTTTAGAACAGGTGTTCCTGTTATTTACTAAACATGAAAATAGTACATGATTTAGTTCCCTTAAGAGATGCGTCAGTGCCTTTGCTCTGTGTTATTTTCTCATTGTGATAATATATTATTTTTAGTTATTAATAAGATTCCTCATTGGGGCACTACCGTTTAAAGAAACCAATAAAGGATGATGTGTCGGTTTTTAATCAAAATAATGTGTTTCAATAGAATATGATAGTAAGGTAATATAATTAGCCCAATAAATGACCGTTTTCGAGTGTAATTTCCAGGGGCRACTCCGAATTGCATGAARGTTTGGATTTGGATTCTACTTRCCCTCCACTTCAGRGTTGAATTTGTGCCGTTGGTTGCTTTTACTTGGGGGTGACATTTACCCCTTCTCGGGGGTAAAAAACGCGTG

>Dvv ABC-A C266167_7.0

CCAATCAGTAGATTATACAACTCATATAATCAGAAGATCTAAGTCAGCATGCAAAATGGCCTACGGCGGCAAATCACTTTTTTTCTCGCAACTGAAAGCAATGCTCAAAAGGAATTTATTACTAAAAAAGAGGCAAAAATTAAAAACGATAGGTGAAATCTTGTTCCCCATCTACTTTCTAGCCCTTCTGGTTGTTATCGAGTTGGTTCTACCTGATCCAAATTTACCAGAAATCAGTACTCCAAGAGGAGAAGAATATTTATTTAAATCTTTTGACAACGGTGAATCTCATAAAATAGCATATGCACCAAATACAACAACAAATATCAACTTTGTCAACAATGTAACCGAAACATGGAAAAAGATGTCCAATTCCAAATCGAGTCTAGAATGGGTCCCTTATAGTGCAGAGGATAAGGTCCAGGAAGCTTATGATCAAGATCATAAATTTGCACCGATGGCAGTGATCTTTAAGAGTGATGTTCCTTATGTGCATACCTCATTAAGTTACCAGATTCGTACTAATCCCTACTATATCAGTACCCCTAGTTCTAATGATATTGGCTGGGATAAACAGTCTTGTAGGGGTGGTACAAACCCAAATTCAAACATGGAAGACGGCAGTACATGTCCAGCTAATACTTACTACTTTTCTGGATTTTTGGCTCTTCAAACCCTCTTAGACTATACGCGAATACAACTTGATAATCCAACTCAAGATAATATACCGTATATATCTTTGGAAATATTCCCAAAAGCTGCAACTACTGTTGGTGATTTTGCAAACATGATTCGCATTATAATTCCAATTTATATGGTACTAGCTCTTTCTCAATTCGTTACGTATCTAATAATTTTAATAGTTGGAGAAAAGGAGAAAAAGATTAAGGAAGGAATGAAACTTATGGGATTGATGGACAAAGTGTTCTGGTTATCATGGTTGATTATATACGCATGTTTTGTAATTCTTTTGGCTACAATATGCGTTTTGCTGTTATTTGCCTTCAAGGTATTTGTTCATGTAAACCTTCTACTAATCTTCATCTTAATGGTCCTCTATGGTATTTCCCTCATTATGTTTGCATTTATGTTAACACCATTTTTCGACAAAGCGAGGACGGCTGGAGTACTAAGTTCCTTTATATTGGTACTAATAATTGTTTTATATTTCGTCGAGCAAATTGTCTCCGGTAAACATCCTCAATATTTATGGGCACTGTCCCTTTTAAGTCCGGCTGGATTTGCATCAGCTCTTGATAAAGTAGCAATATCAGATATTCAAGGAAAAGGATTAGACTTCAGTAATATGTGGGATAACCATGGTTCGGGGGTTCCTTTTGGTGGCAGTTTGGTTATGATAATGGTCGATATAGTATTGTATGCCATAATAGGCTGGTATCTTGATAATGTCATTCCAAGCTCATATGGTGTAAAAAGATCGCCAATATTTTTCCTGATGCCATCTTATTGGAGATCCAGTCATAATCAGATCCCTCCCAACCAACTTAATACAGAAAGTTCTGTAGATGTGGAGCCGGTGCCAAGGGAAATGCAAGACAAAGAAGCAATCAGAATTATAAACTTGAATAAAAGTTTTACGCAATGCCGTAAACCAACCGTTACTGCGCTCGATGGTATCAATTTGTCGATTTACAAAGGCCAAATCACTGCAATTCTGGGTCATAATGGTGCCGGAAAGACTACGCTCTTTAATATTTTGACTGGATTGTCCTCGCCTACTAGTGGGAGTGCTTTGGTTTTTGGATATGATGTCAGTAACCCCAATGAAATGGACAAAATAAGAAGAATGACAGGAGTTTGCCCGCAGCATGATATCCTGTTCGACGATCTGACACCAAGAGAACACTTGGAATTCTTTGCAGCCATAAAAGGAATATCGAACAGACAGTCCGCAATCGAGAAGATAATAAGAGAAATAGATTTACTTGACAAAATTGACACAGCCAGTAGAAGCTTAAGCGGGGGACAAAAACGGAAACTCTCTATTGGTATTGCTCTCATTGGTGATCCAAAAATCATTATACTTGATGAACCAACGGCTGGCGTTGATCCCTATTCAAGAAGACATTTATGGAATGTTTTGCAAAATGTAAGACGCGACAAAGTGATTTTATTGACTACACATTTTATGGACGAAGCTGATATTTTAGCTGATAGAAAAGCTGTTGTTTCCAAAGGAAAAATAAGATGTTGCGGAAGCTCATTATTTTTGAAAAACAAATTTGGAATCGGCTACCATTTGACATTTGTCCTGGAGGATAAGTCAAATGAAAATGCTATAAACCATTTGGTTCTGCAATTCGTCAAAATGGCAAGGAAAGATAGACGACACGGAAAAGAATTGAGTTTTATATTGCCACATAATGCTGTTGAAAATTTTGCTTCACTTTTTGCGGCCATAGAACACGAGATCAGCATTAAATCCGATTTAGGTATTTCCAGTTATGGCGTTTCAATGACTACTTTGGAAGAAGTTTTTCTTGGTTTGCAAAAAGAAGAGGAATATGGCGACGCAACGGTATACCAGGATTTGCCCCAGAGCTCAGAAGGGATTAGCAACGAAAACAGAGCTCTTACCGAAGGTATTCGTTCATTGGAAGCATTTCGCTGTACTCCTAGTACTTCGCAAAATTTAAGAACGTTGATAGGCCTACGACTTTTGAGACTACGACGTGAGAAAAGGAAGCTGTGGATGGTAATCATCTTGCCAATTATTTTTACAGGTCTAGGACTGTACTTAAATAAGGTTATTGACACTGGAAAAAAATACGAACCACCTAAAACATATCCAATGCCATTAGACTTGAGCCATTACCTAGGCTATAATATTAGTATCTACAATGGGAGTCCGGCAGATTTAACTACATTTAAGAATCAGTTAGAGAAAAATGGAGCAGAGCTAGATACTTATGATGGAGAATTTACCTCATTGTTAGACATTGCTCCACATTATGCATCTTTTAACATTCACGATTTTAGTTCTTCTTCTCAAAATATATCCGTACTTTACAATGGCAGCTATTCCAATAACTTACCAATGTTTATTAATCTTATCAGCAATACTTTTTACAGTATGGCCGCAACAACTGGAAAAATCGCAGTTACCACATACCCTTTTGAACTACCTCCAACTAATGTGAGTCCTACTCCTGCTAGTCCAGGGAACTTCATTTTGGGTATGATTTTCCTGTTTGCGCCAATCGTACTAGCAGTTGATATGGTGTATGAAAGAGAGATAAAAGCCAGAAATCAGTTAAGAGTTAATGGACTTCCTTTCACCGTTTACTTTACAAGCTTTTTCCTGGTTCAAATCTTATTAATGGCAACCATAACTATATTGTTAATAATTCTTATTGTTGTTATGAAACCACCGACTTTTTCAAATTCCTCAATAACAGTTTTAGCAATATGGGTTATTCTCTATTGCCCCGCTTCAGTTTTGTTTTGTTCCTGCGTTAGCTACATGTTTGATAAGTCGGAGTCTGCGCAATCGGTTATGCCAAACGTATCTACTCTTCTAGGATTAATACCATATATATCTGTGATGTATGCAAAAGAAAGTGTTGCAACTATTTTGCATTACGTATTTGCATTTACTGATATGATGTATGTACCTTATGGTATGCTTTATTATATACAAAAGATTAATTTGGAATGTATATCTGATCCCCATTGCGAGGGAACTACATTCTCCAGCTTCATGATACCGGAAATTATAGTACTATTTGTTACGCTGATAATTCAAATACCTCTCTTGTTCCTGATTATGCTTATTCTGGATGTTAAGAAAAATGGAGGACAAGTTTTGGATATATTTAGATCAAAGAAAATTTCCGAAAATATTGTTGAAGAGTCCAGAGATGTCGGAGTACATGAGGACAGAGATGTTAAAAATGAGAGACAGCGAGTAAACAATTTAATTAGGGATCCTCAAAATAACCGCTCTGTTATAACAGTTGAGAATCTACATAAGGTTTATCAAAAAGGTGTTAAATCTGGTATTTGCTCACGTTCTGCGGAAAGTCTGAAGGTCGCAATAAAGTCTATATCATTGGCAGTAGATTCGGCAGAAGTATTCGGACTTCTTGGACACAACGGTGCTGGAAAAACTACGGCAATGAAAATAATTACAGCTGAAGAGGCACCAACACGAGGACGGGTACAAATTGTTGGAAGAAACATAACCTCCAGCGCTAATACAGCATTCCAGTATTTGGGCTACTGTCCACAACATGATGCCCAATGGAAAAATATAACTGTGAAAGAACATCTAGAATTGTATTCTCACATAAGGGGTATTCCGAAGAATGAAATTAAAAGAACTGTTGACTTGTACTTAACGGGTCTTCAAATTCACGAACATAAAGATAAACAGGCTGAAAAATGTTCGGGAGGTACCAGAAGGAAGCTCAGTTATGCTATGGCTATGATTGGCAATCCGAAAATAGTTCTTCTCGATGAGCCTAGTACTGGAATGGACCCGCAAAGTAAAAGATTTTTATGGGATACTGTTTTAGCTAGTTTTAGGGGTTCAAGGGGAGCTATTCTTACAACACACTCCATGGAAGAAGCTGATGCGTTATGTTCGAGAATTGGCATTATGGTAAATGGAGAACTTAGATGTCTAGGATCAAGTCAACATCTTAAAAATCTATATGGAGCTGGATACAATTTAGAAGTTAAATTGGCAACACAGCAGGGTACAGATTTACGCCAAAAATTAAAAGAATTAGAAAACTATGTGTTCCATCTTTTTCCCAATGCAGTATTACAGGAAACTTTTGGCGACAGGCTCATATTCAGCGTGCCACAACAAAGTGTTCCTTCCCTGGCCAATTGTTTTAGGAGTTTAGAGGACGGTAAGCGTCGATTAAATATTGAAGAATACAGCTTTAGCCAGACAACATTGGAACAAGTTTTCCTAAAATTCGCCCAAGAAGGTGAACATGAAGATTAATTATGTGATAATGGTATATTACATTACTATTACTATTTATTTGAAGACAT

>Dvv ABC-B D21313+T40801_c0_seq1

TGTGTTTTTAATATTAGTTCAACTAAACTAAATACTAATTTTTAATAGAAATATACAAATTTTTTTTTAAGAAATACTCTCTACCGATTGTAATTTATAAAATTTATAATATAAACCCTGTTTTTCCAGAAGCTCTTTATGAGTTCCCATTTCTACTACATTTCCTTCTTTTAGTACACAAATAACATCGGCATCTTGAATTGTCGTCAAGCGATGAGCTATGGTTATACACGTTCGACTTTTTCTAGCATTGTCCAAAGCTTCTTGTACTATCTTTTCACTTTCATTATCTAATGCTGATGTTGCCTCATCCAATAAAAGTATTTTTGGATTTCGCAGCAAAGCTCGTGCTATGGCAATACGTTGTTTTTGTCCTCCAGAGAGCTGAGTTCCTTTACTACCTACTTTTGTTTCGTAACCCTTTGGAAGATTTAAAATAAATTCATGAATATTTGCAGATTTAGCAGCATCAATAATAGCATCCATTTCCACTTGTTTGTAATTTGCACCATATGCTATATTCTCAGCTATAGTTTTGTCGAATAGATTTGGCTCTTGTGAGACTATTCCCAACTGAGATCTTAACGACTTCAAATCAACATATCGAATATTTTTATCACTAATTTCCACTTTTCCATAACTAGGATCGTAGAATCTTTCCAGCAGTTGAATAATAGTTGATTTTCCGCAGCCACTTGATCCTACCAAAGCAACTGTCTTTCCTTTTGGGATTAAGAGGTTTAATGCTTTTAGAATTGGTATTGAAGGTCTCGTAGGGTATGAAAAGTAAACTTCAGAATATTCTACATTCTCATCTTCCCATAAAAAGTTTAAGGCGTCTGGCATGTTTTGTACGACTGGTTGCCTCTCTAAAAGCGTTATTATTCTGCATGCCGCAACAAGACCTTTTTGGAAATTTGGAGAAAGAGACAATGCGTTTCCAACTGACCATGTACCGACTATCATTACTTCACAAACTATAAAAATTGTTCCATAGGGACAATCACCGGAAATGATTAGGTTAATGCCATATCTTATTCCCGCTACATAGGCAAACAGCATTAGACTTCTAGCTAAACCTACTATTATGCCACGATAATGTGACTTTTTTCTTGCCATTTTCTGATACGGTAATAACTCTTTGATATACTGTTCCATAAATACTTTTTCACAACCTAGAGAAACCACTGTTCTTATATTACCGATAGCTTCTACAGCCAACTTTGCAGATGCTTCTAACATTTTTTGGTTTTTCGTGGCATCCTCTTGTAATACCTTCTGCTCAAAATATACAGAAAAGAATATTACGGGACACAAAGAAAAAAGCACAAAGCTTGTCCTCCACTCAAAATAAAACGAGAACGTACACGTAAGTATGAATGTTGAAATAAAGTTTATTGTTGTTCCTATCTGTGGCCCAGCTGCCCCTTGGACAGCTACAGCATCTCCAGCCAGACGTGCACACAGTGCCCCAACTCCATTGCTTTTTTGGTCATACCAGCCAATTTCCTGTCTTAACATTGCTTCAAATGTTTTCGACCTAATTTTTAGAGTTAAATGTTCTCCAGCAACAGAAAAACTGGTTGTCTGCCAAAACATTGCAACTCCAGTGACTACACCTAGTATTAGGAAATATAAACAATACATATTACTCTCACTCCGTACTTCTCCATCATTATCATTTGCAAAAATTCCCATTATTCCTCCAAAAACTAATCCATAAATTGGTAAAGAAGCTCCCGTTATGAGAGAAGATAAACAGCCAATAAGAATATAAAACCATTCTGAGGCGTTTAATTTTAAAACCTTCCATAAAATGTTCTTCGCAACTGGTTGATTAATCTGTGTATCTTCAAGTTCTTCGTCTTCTTTTTCATTTATAGACTTGTTAGCTTCACTGAATTTTCTGCTCCGTCGTCTATTACCTTTTTCTTCTAAAGTTTCAGTTAGTCCTTGAGAAATTACTAGATTGTGATAAGCTCCTTTCATATCCATTAGTTCAGTATGTGTGCCCATTTCTAAAACCTTTCCTTCATTGATTACCACGATTTTACTTGCTTGTCGTATAGTTGATAGCCGATGTGCAACTATTATTATTGTACAGGTTCCTCGTATTGAATCTAAAGCCTCTTGTATTTCTGCCTCACTTGTCGTATCGAGAGCTGAAGTCGCTTCATCTAGAAGTAGAAACTCGGGAGTTCGTACTAGAGCTCTAGCTATGGCTATCTTTTGTTTCTGGCCACCAGATAACTGAGTTCCTCGTTCTCCTAGAACCGTTTGGTAACCCCGCGGCAAAGTCTGTATAAATGTGTGGATGTCAGCTTTCTTCGCTGCATTCTCTATTTCTTGTTGAGTAGCATTTAGTTTTCCATATCTTATGTTTTCGGCGATGGTAGTGGAAAATAAATCTGGCTCTTGACTTACAACGGAAATCTTTTGTTTAAGCCAGTCTAAATTTAGCTGCTTAATATCTATACCATTTATCTTTATTTGACCCATATCTGGATCGTAAAACCTCTGAAGTAATTGAATACAGGTAGATTTACCACATCCAGATTTTCCAACTAATGCAACTGTCTCACCTTTACTTATTGTAAGATTTATACCTCTAAGAATCTTTACATCTGATCTTGAAGGATAACTAAAATGCACATCTTCAAATGTTACTTCCAAAGCGGCATCATTTATTACAATTCCACTGTTTGCAAATTTTCTGATAACAGGTTTGCTATCTAATACGAAAAATATTTTTTGTGCTGCCCCTTTGGCTGTTCCAAAAGTCTCCAGTAGTGGAGCTCCTAATCCAAAATTCCAAGTAGCTATTAACGTTGAAAAGAACACAGATACCATATTTGCCGGTGTATAAACTTGTTCGTGTTCTGGAAGATGTCTGTCATTCAGTATTAATGTTACTCCATACCAAAACGACAAAGCGTAGCACGCGTATACGAAAAACCATAAGCATCCATTACTTACAGCAGTAAAAAAGCTACGTTTTAAGTTATTCTTTTTGGCATCAACGAGGTGTTTATTATATCTTTCAATTTCTTTGTCTTGTCCATCAAAAGCAACAACAGTTTTGATTGCGGTGAAGACCTCTTCAGCGATGGTACCAGCTTCTCCGTATGATTCCATTTCTTGCGCTGAAAATTTTGTAGATATCCATGATATTATTGTCATAATTGTAGTCGATAAAGGAAGGGATACCATGCACACCAATGCTAATTTCCATCCTTTAACTAGACCCAAAACACATCCTGCTACAAAGGTTGACTCAAAAAATAGAAAAGTTCCAATTTTCTCACCAATTCCCTCTTCTAATTTTGAAATGTTTTGTGTGAATGTACTTGCAAAGTCTCCAGTACGATTCTGGTCAAACCACGCAATATCTTGATTAAGCGTTTTCTCAAGAAAAGCTTTTCTGATTTTGAAAATCTGTCTAGATGCGCTGTAAATAAAAAGGACCGTTGATATATAGGTAGTTAAAATAACGCCTATCCCAACGCCTACCGACTTCATGCCAAACAGCCAAACGGCATTTATTAATTCATCTGCTAACAAAGTCTTTTCTGGCTCACTAAGTGATTCGTTGTAGTTTGAAGCGTATGTTACTATTGCACCGGTAACATCTCCAAATAATGTCATAGAATATGGTTGAACTATTCCACTTAAGGCAGCACAAATAGCTCCAATTGACATAAAAATAATATCCAAAGTAGTACAGTACTGAAACAAACGAAGATAACTTATTTCATTAGTTTTGTTCTTTTCACATTTTAAATCATTCTCATTTTTTTTGTTTCCCATTTTGCATTCGGTTTGCACTAACTACCAACTTTCCTTCTATTTTGTGTATAACCATAAAATTGTTATTGCAGTTTCTTAACTATGTAGAATAAAGTCTGTCATGAGGCATTAGTTAGTATTAAAATGCATCCATAAAAACTAATAGCCAGACAGAATGATTTATCTAATTGTCTATATCAAAAACGAATACAACTGATTCATTTTTTAAATACGAATACGAACGCAAGTCGGTCCAATTAAAATTTGTTCAGCAAATAAAAATCTAATTCTAGCTCAATGT

>Dvv ABC-B D17742

ACTGACTTGAAAATTTGATTTTTACGTTTTTTGAGTATATAATTTGTAGTACAGTCCCTCCTTTTGCAGAAGTTCTTGATGGCTTCCAGATTCAGCAATTACACCATTTGCTACTACGCATATCAAGTCTGCATCTTGTATGGTTGTTAATCTGTGGGCAATCGTAACACATGTCCTTCCTTTTTTAGCTTGATCTAGAGCTTCTTGAACAACCTTCTCACTTTCAGTGTCTAGTGCAGATGTGGCTTCATCAAGGAGTAAAACTTTTGGATTTCTTACTAACGCTCTGGCAATAGCAATTCTTTGTTTTTGTCCACCAGACAACTGAACAGCCTTTTCTCCTAGTTTTGTTTCATATCCCTTCGGTAATCCAGTTATAAAATTATGAATATTGGCATTTTTAGCAGCTTTAATTATCTCATCCATAGAAACCTCCCTTGAATTATCTCCATAAGCAATATTTTCAGCAATAGACTTATTGAACAGATTTGGTTCTTGTGATACGATACCCATATGCGACCTCAACGAATCTAGCGATATATCTTTTAAATCTACACCATCCATTTTTACTTCACCAGATCTAGGATCGTAAAATCTCTCGATGAGTTGAATTATCGTAGATTTTCCGCAACCACTTTCTCCCACTAGAGCTACAGTTTTTCCATTTAGAATGTCCAAATTAAGTCCTTTTAAAATTAGTACGTTGGGTCGTGTTGGATACGTAAATTCGATTTCTGAAAAACTGAAGTTGCCACTTGCCTTAACCATTTGTCGTGAAGAAGGTAAATCACGAATAAGTGGAATTCTTGAAAGGAAGCTTTTAACTTTTTTTGCTGCTGCAACACCCTTAGCAAAGTTTGGAGAAAATGCCAGTGAGTTGGCAATTGAAACTGTCCCCATAATTTGTGCTTGAGATACCTTAAACACTCTATCGTATGGCAATCCATCCCTTATCAAAAATCCGCCGTAGTACATTGCGGTAGCATAAGCAAAGAACAACAAACTTCTGGAAAGACCAAACACGATTGCTCTCCAGTGAACTGTTTTTAACGAAGATTTATAATGTGGCATGAGCTCAGATTCATATAGCTGCTGGAACTTTTCTTCTAAGCCTAAGGAGGCAACTGTTCGTATATTCCCAACGGCTTCTACTGCAGTCCTTGTTGATTTTTGTAGTGCGGAATCTCTTGAATCGTTTCCACCTCTGGTGTTTCTTCTTTCAAAGAACATAGCGATCAAAATAACAGGTGTGAAAGCTGCTGTAACTAAACCCAATTTCCATTCATAATACATGGAAAGACCAATTGCTAAGCAAAATGTGGCTATTGATTGAAGAATGGTTCCAACCCGAATTCCCGTTGCCCCTTGAATATTAGAAGCATCAGAAGACAATTTGGCACAAAGCGCTCCAACCCCATTTGTTTTCTTGTCAAAGAAGCCTATTTCCTGGTATATCATTGCCTTGAATAATCGACTCCTAACTCTTTCTGTCATTTTTTCACCAGCAATTCCTAACAGGTACATCTGAGTAAATACTGATACCATAGTTATGAGTCCTCCCAGCACAAAATATAAGCAATATTTGTTGGTTTCAGATCTTACAAAGTCAGGATCACCATTTGCTAATGTCCCCAAAATGCTACCAAACAAGACTGCAAAAACGGGCATTCCACAACCAACAACAATTGATCCAAGACCAGCAAATAAAATGTAAGGCCATTCTGGAGCATTCATCTTCAATATTTCAAAAACGGAAACGTCTTTATTTTCATTAAAATCGTCTTCATTATCTTCTTTTGTAGCCTCTATGTTATGGTCAGAGCCTGTGCTACTTTCTGCCAAGCTAATAGCTCTTACCACTTTCTTCTTTTCAGCGACTTCAAACTGTTCCGATGATTTGACTTGGGCTGTTACTAATCTATAATACTCTTGTTTAAGTTCCATTAGTTCTTCGTGGGTTCCTTCTTCTACCACGACTCCTTGAGAAAGTACGATAATTTTGTTTGCACCTCGAATTGTTGATAGCCTGTGTGCCACAATTATAGTTGTACATTCAACACTTGCCGCATCTAGAGCTGCTTGTACTTTAGCTTCACTGGTATTATCCAGTGCTGAAGTAGCCTCGTCCAAAAGTAATAAAGTAGGATTTCTTACCAAAGCTCTAGCTATAGCTATCCTTTGTTTTTGTCCACCTGAGAGTTGAGCCCCTTTCTCTCCAACCAATGTATTATATCCGTTAGGAAGAGCTTTAATAAAGTTATGCGCATTAGCTTTTTTAGCTGCTTGAATGACATCTTCCTCTGTTACTCCATCTTTACCGTACCTAATATTTTCTAAAATGGATGTTCCAAAGAGAATTGGTTCTTGACCTACAACTCCTATTTGGCTTCTTAACCAAGTTAAATCGTATTCTTTAATATTTTTTCCGTCTATGGTGACCTCTCCAGAGTCCGCATCGTATAACCGTTGAAGTAACTGAATAACCGTAGATTTTCCACAGCCCGAACTACCTACAAGTGCCACAGTGTCTCCAGATTTAATGTTCAAACTTAATCCTTTTAATATCGGTACATCTTTCCTAGATGGATAACGGAAAGCCACATCTTTAAATTGTATATTTCCTATGAGTACGTTTAGCGTTTTTCCATTTCCTTTTGATAAATTAATTGTAGGTTTGTGATCGATTACACTAAAAATTTTTGACGCCGCCGCTTTTGAGATTCCAAATGCCTCTATATATGGAGAGGCAATACCAAAGTTCATAGATCCTGTCATGACACTAAAGAAAACAGTCACCATATTACTAGGAGTATATATTTGATTCGTAGCTGTCCTGTCCCTTAAAACTAATTTTACACCATACCAGAATGCCAGAGCATAACTGGCATAGATCATGAACCACAATAAACCGAAACCTAATGCTGAAAACATCGATCTTTTTATATTATTGTTTTTGGCTTCAACTAAATTTTTATTATATCTGTCTACTTCTTTCTTCTGACCTCCAAAAGCCGTTATGGTTCTAATTAAAGACAATGCTTCTTCGGCTATYGATCCAGCAGCGCCATATGCTTCCAGTTCATTTTTTGCTAATTTTGTAGTGAGAAGTCCTACAATTCCGATTGTTATTAATGTAGCGGGTAGAGAGGTTAAACATATTAAAGCAAGTTCCCATCCTTTCACAAGAGCTATGATAAGTGAAGTTAAAAAAACAACTTGAAAACTCCAGAATACCGGTACTTTTTCACCTATGCCATCTTCAAATTTGAACAAATCATCTGACATCCTACTGGCAAAATCTCCAGTCTGATTAATATCGTACCAACCTACATCTTGGTTTAAAATGGATTTCAAATATGCTGACCTTATTTTGAATATCTGTCTCATAGCAGAATAGTTAAAAGTGACTGTTGCTAGGTAACTGAACACAAACATTCCTATTCCGATCAGAGAATTCATTAGGGCAAAATATCGGATACCATCAAAAAAATTGTCTTCTGCTATTTTCTTCTGATCGGCAGGTAAATTTATCTGTATCGACGTAGCATATGCAATGATATCTCCTGTGAGGCTACCAAAGAGAATCGTATTTAGGGGTTGTAAGACTCCTGTTCCAGTTGCCGATATAAGTCCTACTACCATTAAAAATTTATCATAAGTACTTGCATACCTAAACATCTGAGGAAAAGATACATTCTTAATTTTTTCCTTTGGTTCTTCACTATTAACAAATTGGGCATCAATACCAATTTTCTCTTTATCCTTTRTACTATGTTTTTTTTCTTCTGTCATTGTAATAATTTTAGTRTAATGTACGCAATATCAAAGGTATTTTAGTTGTGT

>Dvv ABC-B D19147

CCTCATTTCTGGCGGTCAATGTATTACATTCACATCCTATTGTTTAGTTAACAATGATAAAATATGTGATAGTCATTGCAAACCAATGTGTAAACGTTGATGTAAATAATATATACATAATGAGATAATTATTATTTATCGATAGGATTACTGCCAAAACCAGACAAGAATGAATGTATATTCAGTGATAGTCAGTAGTCATAATCTATCGAAGTCGCTCAAGCTTTTGAAATTGGTTAATAGTAGGAGATACTTTAGCAACATGTCTCAGAAAATAAGTCAAAATAAAACATATATTTCAGTAGAAATTCCGGAAACAACCAAAGGTGCCTCAAGAGGATTTTTCAGTAGAATTTTTAAAAGAAACAAAGAAGTAAAAGAAAAACAAGAACCTAGATTGAAAAAATCCGAACTCAAGAGATTGTTTTCTCTTGCCAAACCTGAAAAATGGAAGTTAACTACAGCAATAGGTTTCCTTGTAGTGTCGAGTACAGTAACAATGTCAATCCCTTATAGTTTAGGAAAAATATTAGATATTATATATGTGGGTAGTGGAGACTCAGATGCAGCGAGAGCACGGTTAAACCAAGTGTGTGGTATATTGCTAGGTGTGTTTGTGTTAGGGGCCATTTGTAATTATGCCAGAGTTTACTTAATGTCTACAGCAGGATATAGAATGACAAATGCTTTACGAAGACAAGTTTTTGGTGCAATATTAAGACAAGAACAAGGTTGGTTCGACAAAAGACCTACAGGTGAACTTGTGAACAGACTGTCAGCAGACACACAAATAGTAGGATCTGCTTTATCTCAGAATATATCTGATGGTTTAAGGTCTCTAGTCATGGTATTTGCTGGTACAGGTATGATGTTGTACATGTCTCCACAGTTGGCATTGGTTGGTTTAGCAATAGTACCTCCAGTAGCAGTTGTTGCTGTGCTTTATGGTAGATTTGTGAGAAAGATTTCAAGGAAAGTCCAAGATTCGTTAGCAGATTCTACAAAGGTGGCAGAGGAAAGAATTGCGAATATAAGAACAGTGAAATCATTTGCACAAGAACCTAGAGAAATAACTAGTTATAATATTTCAATTGAAAATGTTCTTAAGTATTGTTACAAAGAAGCAAAAGCAAGAGCTTTATTTTATGGCATGACAGGGTTTAGTGGTCATATTATAATAATATCAGTTTTGTATTATGGTGGGGTGATGGTGTCTTCAAATACCATAACAGTAGGTAATCTCTCATCATTCCTACTATATGCCGCATACATTGGTATTTCTGTTGGTGGATTATCCAGTTTTTATTCAGAACTAAACAAATCTTTAGGGGCAGCAACCAGAATTTGGGAAATAATTGACAGAGAACCCACAATTCCTACCCAAGGAGGTTTAATTCCAATGACAGACGTTGAGGGTCATATTGAGTTCAAAAACGTTAAGTTCTGTTATCCTTCAAGAAGTGACATTGAAATTTTCAAAAGTTTAGTTTTGGACATCCAACCAGGCAAAACGCTCGCCGTTGTTGGTCCTAGTGGTTCGGGCAAGAGTACTTTAGCGGCGCTTTTATTAAGACTATATGATCCTATAGATGGGGCAGTATACATTGATAACCAGAATATTAAGGAGTTAGATCCTGCATGGATTAAGAAACACATCGGTACCGTTAGTCAAGAACCCATACTTTTTTCTTGTTCGATAAAGGAAAATATACTGTATGGAGCAGATGATCCTCAGAAAATCAATGATGACGAATTTATAAGAATTTGTAAAGAAGCCAATGTTTACGAATTTGTTCAAAACCTACCTGAAGGTTTTGAAACTGTTGTAGGAGAGAGAGGGGTTATGCTTAGTGGAGGCCAAAAACAAAGAGTAGCAATAGCTAGAGCTCTTATCAAAAATCCAAAGATACTACTGTTGGATGAAGCCACTAGTGCGCTGGATGCCCAGTCTGAGCATTATGTACAGGAAGCTTTAGATAGAGTTATGAAAGGCAGAACCGTTTTAACCATAGCTCATAGATTATCTACTATTCAAAATGCCGATATTATTGCCGTTCTACAAAATGGACAAATTGTAGAACAAGGAAAGTACGAGCAGTTACTACAAAGTAATGGACCTTTTAGAGAGTTGGTGCAACATCAAACGTTTTCGCAATTAATGGATGACTGATTTTTAATCACGG

>Dvv ABC-B T39715_c0_seq2

GTTAACATTCAAAATATGTTTGGTGCGAAATTTTATACACCCCATAATTTATTATTATTTTCTCTAAAACAACTGCAACACATTTAACACAATTTAAAATCAAATATTAATTGCTAATTGCATAGCCAAGATCAGATTTAATAACGAGTTTCATTTTTCATTTAATAAATTTGGATCACGTTATTCTCAGAGAATTCGTTTTATGAATTTATATGACCTAAATTTGAACTTATATATATTTATCTATTGAAACGGGTGTAATTTCAAAATTTTTATACTATGAAACAAAATGACGTTTGATGTCTTATATATTATGTGCAGTCCGACAGGCACCAACATCAGATACGTGAACCTGCGTAAAAAACGAGACATACGTGACGTTTCCTCCGATTTCACATCACGTGTTTTTATGTCAAACATGTTTTACTTAAATTGTAAGAGTTTGGATTGACAACTCTTAAATTATATATATACCCTACTAAAAGAAAATAAATTTCTTTCGTCATACTGCTGATTTTTTCTTTTCTGGTTCAGTGTGCGATTGATTTTGCGTTTCCCACAATTTCCAATATAGTCCGGAATGACTTTCCAGTAGTGACTTGTGTGTTCCTCTGTCAGCGACTTGACCATTTTCCAGTACTATGATTTCGTCTGCGTCCATGATGGTGCTAAGGCGGTGAGCTATGCAGATACTGGTACGTCCCTTAGTTGCATTTCTCAGTGCCATTAGTATATTATGCTCAGTTATGGAGTCTAAAGAACTAGTGGCTTCGTCAAACACCAGAATAGGGGAGTTTTTCAAAATTGCTCTTGCTATAGCTACTCTCTGCTTCTCGCCGCCACTTAACTTCAATCCTCTTTCACCTACTTGCGTGTTGTATCCTTGGGGCCAGGTCACAATGGAACTGTGAATTTCTGCGAGTTTTGCAGCGTTCATTATTTCTTCTTCTGTAGCTTGAAGATCACCGTAATGGAGGTTATGCCTAATTGTATCATGGAATAAAACGCTATCTTGAGGCACAATGGCGATTGCACGGCGCAAACTGTCCAGGTCTACATCTCTTATATCTTGATTTCCTATGAGGATTCGCCCTTTCGATGGTTCATAAAATCTATACAGCAGTCTAACAAGAGTCGATTTTCCTGATCCGGATCCACCTACAATAGCTACTTTCTTGCCAGGTTCTATTGTTAAATTTAAATCTTTGAAAATTGTTTTTCCGGGTCCATACTCAAAAGACACGTTTTCGAATTTTATCGGTGAAGATTTTGAATCGACATGAAGGTATGGGGCTTCAGGTTTACTCTTTACGGCACTATCCATTGTCATCAGAGTGAACATAGTTTGCATATCTATTAGTGCCTGTCTCACTTCTCTATATACACTTCCCAGAAATCCTAAAGGAATTGATAATTGAAACAGCAGTCCATTTACCATCACTAAATCTCCAACTGTCATGTTCCCTTTAACAATTTCATTAGCTGCTAAAATCATAATCCCACTAAGAGCGGCGCTGAAGATGGCATTTTGTCCAAAATTTAACAGTGCTAAACTAGAAGCTGTCTTCAAACTGGCAGATTCATATTTTTGTAATGCAGAATCGTATCGTTCAGCTTCATACTTTTCATTGTTGAAATACTTAACAGTTTCATAATTGATTAAAGAATCTATGGCTTTATTTCCAGCTTCATTTTCAGCTTTATTCATGTAAACCCTGAACTTTGTCCTCCACTGCGTCACTGACAATGTGTACATAGCATACACACCAACGCATCCCAAAGAAATTCCTGCAAACATAGCACCACATTTAATTCCAAGAATAGATGAAACCAAGGCCAATTCAAACATTGTTGGAACTACGTTGAAAACCATAGCTGACAATACAAAATTTATTCCTCTTGAACCTCTATCGATTGTTTTGGAAAGGGCACCTGTTTGTCTTTGTAAGTGAAATGTTAGGTCTAGATTATGTAAGTGAATGAACACATTTTTCGCAATTTTCCTAATAGAATGCTGAGCAACTTTAGCAAAAACAGCATTCCTTAATTCGTTAAAACCTGCAGCTCCAGCTCTTGCAATACCATATCCTAATAATAAACTAGTTGCTACAGTGGCTACCGTTTGTGGAGCAGTTTCCATATTTAAGGCATTTCCAACATTTAAATAATCCACAGAATATTTAAAAATAAATGGGACACACACATTCATGACCTTGGCTCCAATAAGTAAAGACACTGCCAATTTTACCCTGTCTCTTATGGATTTATCATTTTCTGGCCATATATATTGTAACATAGCTCCTACCATTTGCCGACCAGTGATAGGAGTCTGTTTTTGCAAGGATATAGTTTCTCTGGTCAGAGTGGAGGCCCCTGGATGAAAACATCCTCGAATAGGTTGTGCTTTGGGAGCACTACTAGGTATTTTACTTAAAATGCCTGCTAAAATGCCACCACTACCTTTGCTACTGTTTGATTTTTTCTCAGTCTTTGTAGAATACTCTTTGGTATAATAAAATTGGCTAAGGTATACATTTTTTGTATTTTGTCCATAAACTTTAAATATGCATTGTTTTTGGTAAACTACTGTTTTTAAATGGCGATTTATAGCTTTAAAAGGAATATTACTTAAATTTAACACGGTAGCCATTGTACTGATTTATTGAACTACACATAAATTTAATGAACTACTGGAAAGAAATATTAAATCATATCTTTTACCTTTAAATAAAT

>Dvv ABC-B D9796

AACAACACCAAACAACAGTACAATAACCTACAATTTTTATCCTATTGTGTACTATGACATTTTGGTATGACTAAACAATAAATGTAAATATACAACATAATTTTGAAYAGACTCCAACATTCACATTCAACACATTTCCATACATAAATGACGAATGAATGAAGATTATTTTTTAGTTTGACAATAATAATATGCTGAACATATCATACTGCCCACCAAATATTTCAGTATGGGATGTATGGGTGGATCATGGAATCCCACAGTGTTTCATGAACACTGTGACTTCTTCTGTATTAGCAATATATATACTAATAGCTGGTTCAATACAACTACATATTTACAGACGATGGGGTGTTGAATCTGGAACCAGGTTTTTGCCTAGAGGAAGATTATATTATTTACAAACCTTTTTAATATTGTTTATACCAGTGTTAGAAATAGTTAGATTTATTTTAACCGCAACTGTTTATGATGATAAACATATTTATGGATATATGATTGTTTCTCTAGTATTAACGACATTTGCTTTTCCGTTTTCTTTATGGGTTTTAAAAGTGGAGTTATATAACATTCTTCCATCAGTTCAAACAAGAGGCCATGGGATAGTTTTATTGTTATTTTGGACATTGGCCTTTATATCAGAAAATCTAGCGTTTATCAATTTGACCCAGGAGAACTGGTGGTTTAAACTGAAGGATTTGACCGATCAGCTGGAAATGGCATTATTTATTTTACGATATATTGCTTGCCTACTTATCTTTTTCCTGGGCCTCAAAGCTCCCGGAATAGTAAACGAAGTTGATTATTATATGTTAGGTGGTCATCAAAGGAATGTTATTAATCCGAACAATGAAAATGCCTCTACATGGAAGAATTTTTGGAAAAAGGTGAAGATATTAGCGCCTTTCTTATGGCCAAAGAAAAATTTCACATTACAGTTTAAAGTACTGATTTGCTTCATATTATTATTAGGAGGAAGGGCGGTTAATTTGTTTGTGCCTATCTATCAAAAATTAATTGTTGATAGCATGGAAGAAACTGTGGCAAAGATGCTTTTCAGATGGGACTGGGTTCTCATATATGTTGGTTTAAAATTTCTTCAAGGAGGTGGCACTGGTGGCATGGGTCTATTAAATAATGTGAGATCGTTCTTATGGATTAGAGTCCAACAGTACACAACAAGGGAAGTTGAAGTAGAATTATTTAGGCACTTACATAGTCTTTCGTTGAAGTGGCATCTAGGAAGAAAAACGGGGGAAGTGTTGAGAGTGATGGACAGGGGGACAGATAGCATCAATAACTTATTGAATTACATTATATTTTCCATTTTTCCAACTATAGTGGATATAATAGTAGCAATTGTCTTCTTCGTTTCTGCCTTTAATATATGGTTTGGACTGATCGTTTTTACTACAATGATACTTTACATAGTTCTAACGATCGTTATTACCGAATGGAGAACAAAATTTCAGCGTCGTATGAATTTAGCAGATAATGAAACTCGAAGCAGAAGTGTAGATTCTCTTCTCAATTTTGAAACAGTTAAATATTACGGAGCTGAGAATTACGAAGTGGATGCTTTTAGGGAAGCTGTTCTTAAATTCCAAGATGAAGAATTTAAGTCAAGTATCACTCTTAATATCTTAAACACAGTACAAAATGTTATAATATGCGGAGGGCTATTAGCGGGAAGTTTGCTCTGTGTTTATATGGTAGTAGAACCTAAAACTCTAAAGGCAGGCGATTATGTATTATTTGCTACATATATAGTACAACTTTACGTGCCGCTTAATTGGTTTGGTACCTACTACAGGGCCATTCAAAAGAATTTTGTAGATATGGAAAACATGTTTGACCTTCTCAGAGAAGAACAAGAAATTATTGACGCTCCAGGAGCTACTAATCTATCTGTTCCAAGGGGCCATGTTGAGTTTAAAAACGTCAGTTTTGGATATCTTCCTGAGAAGTTGATATTAAAAAATATAACGTTCAGTGTTCCTTCTGGAAAAACTGTTGCACTAGTAGGACCATCAGGAAGTGGCAAGAGTACTATTATAAGACTCCTCTTCAGGTTTTATGATGTCGATACAGGAGTTATTGTTATAGATGGGCAAAATATTAAGACTGTTACTCAAGAATCACTAAGAAGAGCTATCGGCGTAGTTCCACAGGATACAGTATTATTTAATAATACTGTAGAATACAATATACAATATGGTAGACTAACAGCAACAGTGACTGATGTGATAGAAGCCGCTAGAGGTGCTGATATACACGAAAAAATTCTTACCTTTCCAGAAGCTTATGAAACCAAGGTTGGAGAGAGAGGACTAAGACTAAGTGGTGGTGAGAAGCAGAGAGTGGCTATAGCTAGAACGCTATTGAAGGCTCCAAATATAATCTTACTTGATGAAGCTACCAGTGCCTTAGATACTCAAACGGAAAGAAATATACAAGAATCTCTGAATAGAATGTGTGCAAACAAAACCACGATAATAGTAGCACATAGACTCTCCACCATTATTCATGCAGATGAGATATTAGTTTTACAAGAAGGAGAAATAGTGGAAAGAGGAAAACACGACCACCTGATAGGCCAAGAAGGAATATACGCTAACATGTGGCGACAGCAATTGGAGAATAAAGATAAAGAGTCTTTGGAAAATAGCTCGGAGAGTGCTAAATCTAAATAGCCATAGCGCAGCTCGAACATAGCTAAATTGTGGTACTGAATTGAAATATTCTATTTAATTATAAGTAAAATAGTTAAAATATGGTATTAGAAAACAATATTGTTTTTTATTTAATAAAAAAATAAA

>Dvv ABC-B D13664+T8676_c0_seq1*

CTTCTTTTAGGCGGCCGTTTTTTAGGTTCAGAACTTTCTGGTTTATTTTCTGCAGGCTCTTTGTCTTTATTTTGTAGCTGTTGTTCCCACATACTTGCATATATTCCGCCTTGTGTTATTAAATCTTCGTGCTTTCCTCGCTCCACGATGCTTCCTTCTTGTAAAACTAATATCTCATCTGCGTGGATAATGGTAGATAACCTGTGTGCTACTATGATTGTAGTTTTATTTGTACACATTCTATTCAGAGATTCCTGTATATTTCTTTCAGTTTGTGTATCTAAGGCACTTGTAGCTTCGTCTAATAAGATTATATTTGGTGCTTTTAATAATGTTCTTGCTATGGCTACTCTCTGTTTTTCACCGCCACTAAGTCTAAGTCCTCTCTCTCCAACATTTGTTTCATATGATTCTGGGAACGTGAGAATTTTATCGTGTATGTCAGCGCCTCTAGCGGCTTCGATTACATCGGCATCGGACGCTGTTAGTCGTCCGTACTGTATATTGTATTTTATAGTATTATTAAATAGTACTGTGTCCTGTGGTACCACACCGATAGACCTTCTTAATGACTCTTGAGTAACAGTATTAATATGCTGTCCGTCTATTACTATTAGTCCTGACTCTACATCATAGAATCTGAACAGAAGTCTTATAATTGTACTCTTACCACTTCCTGATGGACCCACCAATGCTACCGTTTTGCCAGATGGGACAGAAAACGAAATATTTCTTAAAACCACTTTTTCAGGGAGATATCTGAAGGTAACGTCTTTAAATTCAACACCACCTCTTGAAACAACTATATCATTAGCGTCTGGAGGATCAACAACTTCCTGTTCTTGTCTAAGAAGGTCGAACATATTTTCCATGTTCACAAAATTCCTTTGAATCATACTGTAGTAATTACCAAACATGTTAAGAGGTATGTAAAGTTGAACTATATAGGTTGCAAATAATACGTAATCTCCGGGTTGTAGAGTGTGAGGTTCCACTATCATGTATACACACAATAAACTTCCTGCCAACAGTCCACTGCATACTATAATGTTCTGTACAGTACTTAAAATATTTGATGTAACGTTTGACTTAAACTCTTCTACTTGAAATTTGAGAACGGCTTCTCTAAACGCTTGTACCTCAAATTTTTCTGTACCGTAATATTTAACAGTTTCAAAATTAAGCAAAGAGTCAACACTTCTCGTTCTAGTTTCATTTTCTGCCGTGATCATGCGACGTCGATACTTTGTTCTCCATTCAGTAATTATAATTGTAAGTACAATGTATAATACCATCGTAGTAAAAACGATTAGCCCGAACCATACGTTGAAGGCTGACACAAAGTAAATGACAGCTATCGTTATATCTACTATAGTCGGCAAAATGGAGAATATGAGAAAGTTTAATAAGTTGTTAATACTGTCTGTTCCTCTATCCATTACTCTCAAAACTTCACCTGTTTTTCGACCAAGATGCCATTTCAATGAAAGACTATGCAAGTGTCTAAATAGTTCCACTTCAACTTCTCTTGTTGTATATTGCTGTACTTTGAGCCACAAGAATGATCTCAAATTATTTAACAATCCCATTCCTCCTGTTCCTCCTCCTTGTAGAAACTTTAAGCCAACATATACAACAACCCAGTCCCATCTAAAAACCATTTTTTCTACGGTACCCTGCATACTATCAAcGATTAACTTTTGGTAGATGGGAACAAACAAATTTACCACTCTCCCGCCACCCAATAACAAAAAACAAACTAGCACTTTTAACTGCAGCACGAAGTCCTTCTTTGGCCAGAGGAATGGCGATAAGATCTTTAACTTTTTCCAAAAGTTGCTCCATGTCGATGTGTTTTCTTTGTTCTTAAATTTCAAATTAACCGCAATGTTGTCAAACTTGACGTTGTTCTTCTGCTTCTTTTCTTGATCATCTTCGACTTGTGTAGCTGTCATGTTAAGTTTTGTAAATGC

>Dvv ABC-B T17837_c0_seq2

GTTTTATTTATATTATACATACTTATTTACAATATTAACAAATAGATTATCCAGCTGGACTGTTTTGTTGTTGGTATGCTAATGACCAATAGTAGCCTCCCAGTTTCTTCAGCGATTCATGAGTACCCATCTCTACAATTTTTCCTTTATTTAATACAACAATCAAATCTGCATTCTGGATTGTAGACAATCTATGAGCTATCACTATCACCGTTCTACCTGTCCGTGCTCGTTCCAATGCTTGCTGAACTATTTTTTCTGATTCTGTATCCAAAGCACTTGTAGCTTCATCCAAAAGTAAAACAACTGGATTTTTAAGAAGAGCACGGGCAATGGCAATTCTCTGTTTCTGGCCCCCGGATAAAGTCACTCCTCTTTCACCAACAGGTGTGTTATAGCCCTTCGGAAAACTGGTTATAAATTCATCCGCATTCGCCAGTAGTGCAGCTTCTTTTACCTCATCATCTGAAGCATCAGGTTTGCCGTAGCGAATATTTTCCATTATTGTAGTACCAAACAATACTGGTTCCTGGCTTATTAAACCCAGTACTCTACCTCTTAACCACGAAGGATCCAATGATCTTATATCGTGTCCATCCAATGTTATCGAACCATCTTTTACATCATAAAATCTTTCTAAAAGTGCTACCACTGTCGATTTTCCATTACCAGACGCCCCTACAATAGCAACTGTTTTTCCTGATGGTACGCTAAGATTAAAACCTTGCAGAATTATCTGCTGACTTCTAGTTGGATAAGCAAAACAAACGTTCTTAAATTCAATATCCCCCTTCACTGATTCATATGGTAATACTTTTCCTCCTGTAAGTGCCATTTTAGGAGTTTTATTAATGTATTCGAAAACTCTCGAACCAGCTGCTACGCCCCTTACTACAGAGCCAAAAAGTAATGATATTTGAGCCAAAGAGCGTTGTATGGTTTGAGATGCTACTAAATATGCCATAACTTCCCCAGCAGATAACTGATTGGTTGATAGTAAGTAACCACCCATGTACAAAGTAGATAACACCATACCATTTATAAACATATTGGTTCCAGCTTGAAATAAACCAATGCCTAATCCCAAATCCTCATTTAAAACCATTGCTCTATCAGCTTCTGTGTTAAATAATTCCTTTTCTTGGTCCTCCATGGCAAACGCTCGAACTGTTCTGATATTGCTCACCGCCTCGTCCGCTACGGCAGTTGTTTTTTCTACCTGAGCTTGAGCTCTTCTTGAAACTGATCTTAGTAGAGAGCCGAAAACTGTTCCTACGGCTATAACAGATGGTATGCAAAGTAGAGAAATGAATGTCATTTGAGGTGATAGCATAATTAAAGATACTGAACAACCGACAATCTGCGTGGCCGCTCGCAAACCTCCAGAAACGATTTGTTTGAAGCTACTTTTAAAGTCTTGAACATCTGCTGTTAATCTGTTAATAATTTCACCTGTTCTTTGCTGATCAAAAAATGCTATATCCTGTTTTAATATAGATTCAAATAAATCTGTCCTCATTTTATATGCCATACGTTCTCCTAAATTCGAAAGCATAAAGATGTAGAAAAATGTACATACACTCTGAGCAAGATACATGGATATTAACTTTATAACAGGTCTCTTCATTTCATTAAGAAATAATTCACTATCCCTGCTTTCACTAAATTTTGCTAGTACATTCACCACACCACCTATAACTTGAGGTATTTGTATATTTAATAAAGCCACAGCCAATGCCCCCACAATTGCTGCTAAGAAGTACCAAATATGTGGTTTCAAGTACTTCCATAGCCTTGCCCAATCAAATTTAACATTTTTGTCTGATTTATTTTCATAACCAGCCATTCGAGTCTTCTTTGCTTCGCATAATACCCCATTGTGTGATATGTACAATTTTATAATTAGACCACCAGTAAGTGTAACCCCAAATGTTGCAACTTTTGATGGTATAAATGATTTTGGAACTGGTTTTTGGGACACTGTTCTGGAGATATAATTTTTAATATGACCCTGGCAGAAATATTTCTTAAATATTGCACTATTGTGGAATAATAATTTGTTATGTAAATTTTGTTGAATTAATCTCCACATTATTGTTTATATATTTCTTTTAAAATTTGTTGGCTAGAAGAAAACCAAAATTTATTTTAAATAAAAATTAAAAACTATAATAATTAGCTATGAATGTATTTTACATTTTCATTAGAATTTTTTGAATATTAAAATTTGTGGTA

>Dvv ABC-C D14968

CTGTCGTTATGATCTCATAAAAGTTCTTAATTTAAAATTGGCTAATAATCACAGGAACATTTTTTTGAAGACCTATCTATCTTTAATTTGTTTATAAAAACTTTCTTCTGCTATTCCTTGTAAGTTCTCTGCCATTGTGGTACCAGTTTGTCGAACTAATCCATAGAATACCCCTTTCTTCTTAAGCAGCTTGTGTGGATGATCGAATTCAACAGCTTCTCCAGCGTCCATAACTAAAACCTTATCGGAATCCATGATTGTGTGCAATCTGTGTGCTATCGTTAACACAGTACATTCAGAAAATTTCAGTCTTATAGTGGTTTGAATGATGGAATCAGTCATTGGGTCTACGTTGGCAGTTGCTTCGTCGAGTACCAGAATCTTGTTTCTTCTAATTATCGCTCTAGCTAAGCAGACCAGCTGCCTTTGGCCAACACTGAAGTTTGAACCGCCTTCGGCCATTTTGCTGTCCAGACCTGCTGGTAATTCATCAACGGCGTGTTTTAGTTCTACTTCCTCCAAAGCATCCCACAGAACCTCATCTTTGTGTTCATCGAACGGATCTAAATTTTTCCTTAGCGTTCCTGAAAACAATACTGGTTCTTGGGGTATGATTGATATCTTAGATCTAAGCAATCTCAATTCTACGCTCTTACTATCTATACCATCTATAAATATGTGTCCCTCATTTTGAGCCAACCTAAATACAGCTTGTATCAAAGAAGATTTACCAGCACCGGTCCGACCAACTATACCAATCTTCTGTTTAGGTTTGACTTCAAAGTTGAGATTTTTCAACACGTATGGACTATCATCAGAATATCTCAAGGACATGTTCTCAAACTTCATATGGCCCATGCTTGGCCAATTAGGGGGTGGCTCCTTTTTAATCTCGTCTTTTTCCTTTGGAAGGTCTGCGTATTCTTGTACTCTTTCCACTGATGTCATTTGATTTTCGAGTTCACTCCATTGACGCATACCCCATTGGAACATTCCAGAAAGTGTTATTGCTTGAGTCAATGATAAGCCGACATTCCCACCAAAAGTTTCTTTCTGTATAAACAATATGCTTACGACCACCATTCCTATAAAAATTACACAGTGTAGATCTAGCCAAAACCCAAATCCACGATTAGCAGTCAAAAACATATAGTACGCTGCAGTATAAGCATCTTGAAAGTGATCGAATTCTTTAGTAAGAATCTCTTCAGCTCTAAAAGCTCTTATAGTTGTTAGACCTTGCAAAGATGCTGATAAATGCGTAAATATTGGACTACGAGTTACAGCTTCTACTCTTTTAATGTCTCTGCTTGATGCTAAAAATGCTTGCCTTATAAAGTAGAAAATAACTAATATTACGCCGGTCGGTAGGAGAACCCATGGATTTACTGATGCAATAACTAAAGTTGTACCAGTAACAATCAGACCAATCCCAATCGTATCTACCAAGCAGTTAGGCAAAGACTCATCCAAAGCTCCAATATCTTTTGAAAATCTATTCAAAATTCTTCCAGATGGATTAGTATTAAAGAACAACATCGGAGAAAAAATAATTTTCCCAAACATAGAATTGTGTAGATTCGTAGAAGCTCTCAAGCAAAATCTGAAGAACGTTATCGATCTTGTCACTGCTAGTATGATGGTAAGTATTACTAAGCAACTGTATATTAATAAGGTGTTGTCTTCCGTTAACACTGTACTCCAAAATGGAGGTTTATTGTCATTTGTAGAATAGGTTGCGTTTGCTAAAGATTCATTAAGAGTTTTATTAGTAAAGGCTCCATTGTTTTTGTTGTCTACTCGCCATTGTTCTACGTTGACCCAAGTTGTTAAAAATATATCAGTCAAACTTCCGAATACTTGAGCAGCTACGAAAGTACATAACAGCACCACAGATTTTAACCAGTAACCACCAGCATTAATATAATTTGCATAAACCCTTTTCGATACTGTACCAGTAGCCCTTTCTTCCCTTTTTTGCTCTATAGCTTCATGTTCGGCTTCACTAGCTTCGGACTCTACCGACTCCGCCTTAGAAATTCTACGTGCGCCATCTTTTTTCTCTTCATCTTTTGAAGAAGCTAGAAGTTTAGTAAAGGCACTGTCTGAATTCTTTAAAGCCTGGTATGTTCCTGAGGCTCTGATTTTTCCGTCTTCGAATAAATAGATGCAGTTAGCAGTACGAAGGTACTGCAGCTGATGCGTCACTAGAATTACACATTTATGTTTCAAATAGCCAGTTATGCAATTGGTGTATAATTGTTTGCCTACGTGAGTATCTACAGCAGAAAGGGGATCGTCTAGCAAATAAATGTCAGATTCTTTATAGATAGCACGTGCTAAGTTAATCCTTGCTCTTTGTCCTCCACTCAACATAACTCCTCTTTCCCCTACTAGGGTTCTGTCTGCGTGAGGAAATAACGCAAGATCTTTCTCCAGAGCGCAGACTCTTAAAACTTCCTCATACTTAATTTCATCGTACTTCTGACCAAAGATAATGTTCTGTCTTATGCTACCACCAAATATCCAAGGCTCTTGAGACGCATAAGATATAGAACCGTTAACTTCAACACTACCCTCTATAGGATCTAATTCTTTTAAAATGACATGTAACAATGTAGATTTTCCTCCACCCACTGTTCCTACCACAGCTATTAACTGATTTGATTTGGCTTCGAATGTTATCTCTTCCAAAGTATTTTCTGGTAAAGATTTGATCCATTTAACAGAGGCGTTTTTAATTTTAACGCCTACTTCTTTCGCGTCCGTTTTATTTGATATCTTTTCAGTGACACCATTTAAACCGTTCTTTTCTGAGTCAGTTCTTCTGTCTAATTCGTCGTACATCAAAAACTTTTGTATCCTAGATATCGACACCTTGGTTTCTGCAAATTGCGTAATGGCTTGAGGAAATTGTTGGGTGACAGATACTCTTAGGAATGCGTAAAACGATGTTACTGTAAATGCGTAAGAAGCTGTCAAGGTGTTGCCTGTGAGGACGTATGTCATCACACAGAGGAAGACAGCTGATCGACTCATTGTAAGGTTGAACGACATCATAATGGCTCGGATAGTAGATGTGTGTCCTATTTCGGTAATTTCTCTCTTTCTAACCATTTCCACTAATTTTGCGAACGGCTTTTCCCACGTGTACATTTTTATCACTTGTATTCCGTTAATAATTTCATTCATCAGTCTTACTCTCTCATCTGTTCGTATGGCAGTTTTGAGCCTATATTGTGACGTTAATTTGGCCATATACATTTGAAACGGTATAAAGCTTAGTAGAAAAACGCATCCAATTAATCCGGTTGGTCCAACATAAAAATATAGCAAAATCATAACAACAACTGTTTCACAAGGTGCTAACCATACGTTATGAATGTGTTGACCTGAAAAGTCAAACCTTCCTACATCATTAGATAACAGATTTACCATTTGTCCAATTGTCGTCTCTGCTAATGATGTTTTACTTAATTTTAATGCTTTCCTATAGATCAACGCACATGATGCAACTCTAACCTTCATTCCTAAGGACATGACCAACATCTGGTAGTTATGGACGGACGTGACTTGGATGAGACTTGCTACGATTATTAAAAATGCGTTGATGTAGATTTCTGTTTGGTTGGTTGTGGCACCTGGTTGGAAATATGACACTAGTCTGGAGATCAACATTGGTTGTGCAATTTTTATAAAATCTGAAAACATATTAAAAAAGGCATAATAAAATAATTCTAGCCTAAATACTACAAAAAGGGCCCATAAAAAAGACGGCTTCGATTTTTTAAGTTCGTTTTTCCATGCTACTACTAATTGATCTGCTAAATATCTGGATTGTTGGGAATGTCTAGTTTGGTACATATCATTTTCGTCTAAATCTTTCCTTAATCCTTTAGAGAGAAATCCTGGTAACCAGCAAAAAAATATGTGTGAAAATAAATTAGTTCTTTTCAATGGATGCGGTTTCTTTTGTATTCGATTTTTTTTAACTTCTTCCATTATCTCTTTTGTACTTTAACATAAAATAAGTAGGTAAACAGCTGACCACTCTTTCCTGACACAACTCTTTCCACAGATCACTTACAAGTTCAATGTCATTTGTAAAATTATTCCGAAACACAGTTTTTGATAACTTTTAGGGCAAATTATATCACGACAGTGTTTTGCGTTCGTTTGTATCTTTG

>Dvv ABC-C D12703

CTGTACTAATGTGTGCAGATCTTTACTTTTGGAGGTTCATATAACTCTCTTCGGCAATTTTACATAAATTCTCAGCCATCGATTTTCCAGTTTGTTTAACCAAACTACAGAATATTCCCTCTGTATTTTGCAACAAAATGTGCGGATGATTGAATTCTACCGCTGAACCTGCATCCATAACTAATACCTTATCAGAGTCCATGACTGTATGCAATCTGTGTGCTATTGTTAGAACGGTACAATTGGCAAATTTGTTTCGTATAGTAGACTGTATTAAAGCGTCTGTTTTTGGATCAACATTGGCTGTAGCTTCATCCAAAACAAGGATCTTATTTTTTCTGACTATAGCTCTAGCCAAGCACACGAGTTGACGTTGACCCAAACTAAGGTTTGATCCTGCTTCACCGATTTTGCTTTCTAAGCCCATTTCCATTTCGGAGACAACATCCTTTAGAGCTACTTCTTCTAGAGCTCGCCATAGGTCTTTATCATCGTATTCATCCATAGGATCTAAGTTTGATCGAACTGTACCAGAAAATAATACAGGTGACTGGGGTATAATAGCTATCTTTGAACGCATACTGATTAAGGGGATATCACTAGTACAGATTCCATCAATCAATATCTTTCCTTCAATATCTGTTAATCTGAAAAGAGCAGAAATCAACGTTGATTTTCCCGCTCCTGTTCTTCCTACAATTCCAACCTTCTCCTTAGGTTTAATCTTAAAAGTAAGATTGTTCAAAACATACGGTAGGTTTGTGGCATACCGTAAATAAACAGATTGGAACTCAAGCATGCCATATTCTGGCCATTTTTTGGGCGGTTCCTTTTGTTTCTCAAGAGATTCTTGTTTTATATTAATGTAATCAAGGACTCTTTCCACTGACGTCATCGAGTTGTCCAATTCGCTCCATTGGCGCATGCCCCATTGGAACATGCCTGTTAAACTTATGGCTTGGGTGACTGCTAGACCTACGTTGCCGCCATAAGCACTGCCGAAAATGAAGAAACTGAAAGTAACCAATGCAATATAGCCAACGCAAATAAGATCTAACCAGAAGCCGAAGGCTCGATTGCAGCACACAAATAGATAATACGCTGAGCTGTGCAAATCCTGATGTTTGTCGAATTCATTTCTTAAGATTTCTTGAGCTCCAAAAGCTCGTACTGTAGTAAGTCCTTGCAGAGATGCGTGTATGTGAGAAAAGACTGGACTTCGTGTTGCTGCTTCCATACGTTTCAAATTTCTACTTGAAGAAACGTAAACAACTTTGAATAAATAGAACAGACAACCTATTGCTAGAGTTGATATCAGAATCCAAGGATTGACAGTTCCAATTACAATGTTAACAGCTAGAACATTTAGAGCAATCTGAATTGTGTCGACTAAAACGTTGGGCATTGTTTCATCTATACATCCCATATCTTTCGAAAATCTGTTGAGTATTCTGCCAGAAGGGTTTGTGTTGAAGAAGAACATAGTACCATTACAAATCTTATGCAACATAGTATTGTGCAGTTTTACAGATGCGTTCATACACATTTTATAGAAACATAAAGACCTCATAACTGTTACAGTGATCACAGATAAAATAATACAGCTGTATATTATAATGCATCTATCACTATTGAAGAAATCAGGCTGTATAGGTGTCAGATGTCCTAGAGAATATTTCCAAACATAATTGTAGTTTTCTTCCATATAAACGTCATCTGAGTATCCTAGAAGTAATGTTTTATTTCCTTCTTCTTCTTGAAAGGTTTCTTGTTCTCTATTTACCCAGAAATTTACAAAATAGTCCGATCCGCTAGCCAAGAACTGAGTCAGCACAAACAGCATGAGCACCCAAAATCCATAAAACCAACCACCTGCATATTTCAAGTACCCAGAATAGGCTCTAGATGTCAATTTAACGACTACAGTTTCCTCAGTAATTTCTGTGGGCTTCTCATCGGACATATTTTCAAAATATTCTATTTCTGACGATTCTTCATCTATCGTATCACTTTCCTCTTCTTCAACGTTGCTGACGAGTAGTCGCGCGAAGTCGGTGTTGGATTCCGATATTTCTTTGAATGTTCCACTTACGACGATCTTTCCACGTTCTAGTAGGTATATTTTGTGTACGTTTTTTAAATATTGCAGTTGATGGGTAACAAGAACAGTGCATTTTCCTCGCAAATAACTGCAAATACAGTCCTCAAATATTTGTTTACCTACGTGAGTATCGACGGCTGAAAGCGGATCATCTAATATATAAATATCTGCATCCTTATAGACAGCCCTTGCCAAATTGACTCTAGCCCTTTGGCCTCCACTAAGAGCAACTCCACGATCACCAACCATACTCTTATCTCCATGAGCTAATAATGACAGATCTCGTTCCAAAGCACAGACTTTGATAACTCGCTCATACTTTTTGGGATCCCATGGTTCACCAAATAAAATATTTTGTTTGATGGTACTGGCAAACAACCATGGCTCTTGTGAAGCATAGGAGATTTTACCAACAGTATCTTTGTTGCCTTGGCTGAGTGGTAGCTCTTTCATGATGGTTTGGAGAAGAGATGTTTTACCAGATCCCACTGCTCCTACTATAGCTACCAGTTGTTGAGGACCAACGTTAAAATTTATATTCATCAAAGTGTTTTCTTCTTGTGTATCAATCCATTTAGCTGAGACGTTTTCTAAATACACACCTACAGATTTCTTTTTTTTAGAACTATGTATAAGCGATGTACCAGATGTTATCTTCTTAGCTTGGTATGGTTCTCGTATAGGCTCATAATAAACCTCATCAAACTGAAGAAACTCTTCTATTCTAGCTATAGATACCAAGGCTTCCGCAGTTTGAATTATAGCTTGAGGAAAGTCATTACTGACTGCTGATTTTAAAATGTTATAGAACGGAGAGATAACGTAAACGTACCCAGCATTAAGTCTTTTTCCAGTGAAAGTATAAGTTATAATACATAAAAATACTGCTGCTCTTGTTATAAATTTGCTGAAAGACATATGAAGAGCTCGAATGTAGGAAGTAAGGCGAATTTGTTTTATTTCTGAATTCCTTATATGTTCGATCAGTTTGGAAAAAGATTTTTCCCAAGTATACATCTTGATCACTTGTATCCCACAAATGACTTCACTCATGAGTAAAACCCTTTCATCTGTTTTACCAGCTGTTTTCTGCCTGAATTGCGTTGTTAGCTTTCCCATCACCATTTGAAAGGGAATAAATAGAAGCATAATYAAGAATCCAGCTAGCCCGGCATATCCAGCTACCTCAAAAATCAAATACATCACAACTATGCTTTCCAACGGACCAACCCAAAGAAAATGCCAATATCTAAAAAGATTGTCAAACCTGTTAACATCATTAGACAGCAGGTTTATCATCTGACCGATCGTTGTGTTCACTAAAGCTTTTTTGCTTAATTTCAAACTTTTTCTGTAAATCAACGAACAACAAGCTATTCTGATTTTCATTCCAAGGTGTTGCAGTGAAAGATGGAATGAATGACAGAAAATTACGTAAAAAAGTGAACACAAAACTATAAACAGGGAGTATATGTATGCTTCTGTGAGAGTTACTGACGTCTGGTTTGGTTCGTAATATTCTATAAGCTTCTTTAAGAGTAAAGGCTGTGATAACTTTATACAAAATTCTAAAACAAAAGTACAACAGGCGTGAATGAGGATATCCTTTTTGAATAGTTTCCAAATCGGACGTATGAGGCATGGGTGTGAGTGTTCTAGTTTTTCTAACTTCCAAAACGCTTCGAGCTGATCTCCGAGGTAATGAGACTCATGTTCTTCAAGGGGCTTGTATAAGTCGTCTTCATTTAGTACTTTGTTCCATCCCTTGTAAAATACTTTTAAACCCCAACAAAAAAATAAATTTGACAAAAAGTTTGAGCTTTGTTGTGGATTTTTACCCTTATTCTTGAGTTCATCACAATTGCCATCATCCATAATTCATATACCAATTGTTTTTAATTTTATATTTGCCACGGGTAATTTAAATTTAAATAAAAAAACACGTTAAAACTGTAAAACCAAACACAAAATGCTTCACTCTGTCCTAAAGTTGTTCGTTGAGCCGTGAACCATAAAATGGCAAACTTTTTAAAACCGATTAAATTGGGTTTTGACAAACTCTTAATTACTATCAGTTTTTATAGTATTATTTTTCTTTTTCGATAATTATTTCAGAATTGTTCGTTACAGTTTGTCAGTTAAGTATCTAAAGTTATCGCTAGTATTCGRTATAACGCTACGCTTGGTCGKCTGTAACAGACGATCAGTTGTGTTGTGTGGATTCAGACAACTGTTT

>Dvv ABC-C T41602_c0_seq1

TTATAAAAAAATATTAGATTACAACGTAACTAAACACTAAAAAACTATTAAATTAATTAAGTGTACATATATGACGTTTATTTAAATATACAACTCTCTATATGTTTCACAGTTCTAATAATTCATTATAATTTTTTCAAGTCGATTCTTTTTCTTTAGAAAACAGTATTGTTATCTCTAAGCATCTTGCTTTGTTCTGCTATTGCGAATAAATTCTTTGCTGTTGCTTTGCCTGTTTGCATAACTAGAGAATAGAAAACACCATTGATATTTTGTAAAAGAGTAAAAGGATGGTCAAACTCTACTGCTTGTCCAGCATCCATCACTAGAACTTTGTCCGAATCCATAATAGTATTCAATCTGTGAGCGATTGTTAATACCGTACAATTAGCAAACTTCTTTCTGATTGTTTTTTGAATTAAAGCGTCTGTGTAGGGATCAACGTTTGCAGTTGCTTCATCCAGGACTAAAATTTTGTTGTTGCCAACTATTGCTCTTGCTAGACAGACCAATTGTCTCTGTCCCACACTGAAATTAGTACCTCCTTCAGCCATTTTATTAGCTAAACCTGCTGGCAGTTCTTCTACTGCTTGCTTTAATTCCACCTCTTCCAATGCGTTCCATAGGGCCTCATCAGTATACTCGTCGAAGGGATCTAAGTTGTTTCTTAAAGTTCCAGAAAATAGTACTGGTTCTTGAGGTATAATTGAAATTTTTGATCTAAGAACCTTTAAGGATATTTTCTTGGTATCAAAATCGTCCACCAAAATCCTGCCCTCAATATGGGTTAATCGGAAGAGAGCTTGAATTAAAGATGACTTTCCTGCTCCAGTTCGTCCTACAATACCAATTTTTTCTGAAGGTTTGATAGTAAAACTGAGATTTTTAAGTACAGGAGGATCATCAGTTGTGTAATAAAGCGACATATTATCAAATTGTATTTCTCCAGCATGTGGCCAAGTTTTCGGTGGTTCGATGCTATGCTCATCGTCTTCCTGTTTTAAGTCTGCGTATTCCTGAACTCTTTCGACGGATGTCATTTGGTTTTCAAGTTCACTCCATTGCCGCATGCCCCATTGGAACATGCCAGTGAGTCCTAATGTTTGAGTGAGTGCTAGACCCATATTGCCACCATATTGTTCACTCTTTACAAAAACTAACGCCACTATGACCAAACCAACGTATATAACGCATATGAAATCTAACCAAAAACCAAAAGATCTATTCGCCGCAAGATACATAAAGAATGCAGCACTGTGTAAGTTTTGATATCTATCGAATTCTTCTTTAAGAATATCTTGTGCCCGAAATGCTCTTATGGTCGTTAATCCCTGCAAGGAAGCTGTAAGGTGAGTATAAATTGGACTTCGAGTAACTGATTCGACTCGTTTTACATCACGACTTGTTTCTAAGAAAATTACTCTCAAAAAGTAGAAGAAAACTGCTATGGCCACCGTGGGTATCATAATCCAATATGTAAGACTTCCTATTACAGTAGTGATAGCGGCCACAGCCAGAGCTATTTGTACTGTATCCAGTAAGGTCGTTGGTAAGACCTCATCAACTGAACCGATATCTTTAGAAAATCTATTAAGTATCCTTCCAGACGGGTTGGTATTAAAAAATCTCATTGGGCTATATACTATATTCATGAACATCTTATTGTGCAGTTTGGTTGATGCACCTATACACCACAAGTAAAAGCTTAGTGATCTAATGTTAACGATAATGACGACTGATGCTACTAGAGCAGTATAAAAATAAACAGTATTCTTCGAAGTGAACACAGGGGTCAGCCACCAATTTTCGAATGAGTGATAAACTACTGCGTCTGGATCTGTTGCATTTATTATAACTTTTGTTGAGTTCCATTGTTGGATATTTACCCAGAAAGTTACAAAATAATCCGATAAACTATCCAAAGCCTGGCCAAAGAAAAACATGAAGATCAACAGAAACACTTTAATTAGATTTCCTCCCGCTCTGGCATAACTCATGTAAACGTGGTCAGATATATTACCACTTCCTGTACTTTCGCGTTGTAAGACCTGGACAGCACCTTCTTTCTCGGGCTTTTCAGATTTAGTCGCAACTCGCTGAGATTTTCTTCTGGTTTCTTCTTCTTCCTCTTCAATGTCAGCTAACAATTTACTGTATTCTGTATTCGACTCTCTTATATCAGAGTAAGATCCTGATACTGCTATCTTACCGTTTGTCATTAAGTAGATTTTATCAATGTTCTTTAAATGTTGTAATTGATGCGTTACTAATACGACACACTTATTACCGAGATATCCACAAATACATTCTTCAAATAGATGTTTGCCTACATGAGTGTCAACAGCTGAGAGGGGGTCATCTAATAAATATATATCAGCATCTTTATAGACTGCTCGAGCTAAGTTTATTCTTGCTCGTTGTCCGCCACTAAGAGTAACTCCCCTTTCTCCTGCTAAAGTTCTATCCCCGTGCGGGAATAATGTGAAATCTCTTTCTAAAGCACATACTTTAACTACTTCATCGTATTTTTCCTGGTCGAACTCTTCTCCAAAAAGAATGTTTTGCCTTATGCTGCCACCAAACAACCATGGTTCTTGGGAAGCATAAGACACTGTACCAGTTATATCTACGGAGCCACTTTGAAGTTCTAATTCTTTCATGATTATGTGCAATAACGTTGATTTCCCACTGCCTACAGATCCAACAACAGCTACAACGTCACCCGCTGTAACGTCCATGTTAATATTTTCCAAATTATTGTCTGGTTGTGATTTTAGCCACTTTGCCGATGCCTTTTTGATATGAATACTCCCATTTGACGTTTGTAAAACTTTATCAGTAATATATTTATGTTGTTTTTTGACTTCCTTCAGTTCTTGTCCAGGTTCAAAATATTTGTCAGCCTCTAGTTCGTCAAACAGTAAAAAGCTTTGGATTCTCTTCATTGATATAAACATTTCGGAACCTTGAGATACTGCCTGGGGGAACCACATGGTTATAGAACCTAGAAGTCGATAGTAGGAAGCGACTGTGTAGGCGTATGAAGCAGTTAGAGTATTTCCTGAGATTACATAAGTAATTATGCTGATCGCAACAGCACCTTTATTTAACACTGTAGTCATAGACATGAGAAGTCCACGAATTAAAGAGGTTTTTCGGATATACTTCATTTCCATTTTTCTTACATATTCTACTAGTTTTGCAAAGGAATACTCCCAAGTGTACATTTTAATGACCTGGATACCAGAAATAATTTCGTTCATTAGTCTTACTCTTTCATCTGTACGTGTAGCAGTTTTTAACCTAAATTGTGATGTTTTTTTACCCAACCATGATTGGAGTGGTATGGACAGTAACAAGAACACGGTTCCTAGTAGTGCTGTCCATCCAACAACAAGATACAATAGCACCATCACGACGGTTGTCTGTATGGGTGCCAAATAAAAATGATGAAGGTGATGTGTAGCTTGATCGAATCTTCCTACATCATTCGATAATAAATTTACCATTTGACCTATTGTAGTCTCCGCTAAAGCAGATTTGCTGAGTTTTAAAGACTTTCTATAGATTAGTGAACAAGATGCTATTCTCATCTTCATTCCTATTTGCATTACGGCCAAATTGAATCTGTGTACCATTATTACATTAAGAAATGAAGTAACTATGATTAAACCGGCGTACAAATAAATGTCGTTCATGCTCTCTTCTGTTGGTATTTCTTCATACACCTTTAGCAGTTTTGAAATCAGAAACGGTTGAGCCATTCTTATACCTTCAGTTGTAAAAGCCAATATGTTTAATAAGAATATTTCGCAGAAAAATGTTCCTATGAGTACTTTCCAGTAAGCTGGGTTTTTGTTATTTTTAATGTGTTTGCTCCATCTTCGCTCCAATCTTGCACCCAGCGATCCTGAATCATGTTCAGATCTATGTTTATACATATCATCTTCAGTTAGATCTCTTTTATACCCTTTGGCAAAGTAAGGAATAAGCCAGCAGAAAAATAGTCTGGATATTGCATTTGTTCTTTCTATTGGATTCGATTTTTTCGGAGGTTGATCATTTCTTATTTCCTCCATTTTTATTGTCTACCGTATATTGTTGCAATCATCCAAATATTTAACGTGTGTGCACTATTCAAATTTATCATGAATGCACACCGGTTTTGGCAATTACAATGCAAT

>Dvv ABC-C D10642

TTGGCAAGTAACAAAAATTATAACTGGTCTAAATACTGTTCTTGAAAAGTATGAAAAGATCACAGCAATCTTAAAATTAATTCCATGAGATAAAATTCTTAAACTCTTTTCTTCTCGTAACTTTCTTTGGCTATGTTTTCTAAATTCTTTGCAGTAGAAGGCCCAGTAGCGTCAACCAAATTGTGAAGCACACCATGTACGTTTTGTAGAAGAAGATATGGATGATTAAATTCCTCCACTCTGCCGGAATTCATAACCAATATTTTGTCAGAATCCATTACCGTATGTAGCCTGTGGGCAATTGTCAGCACCGTACAATCCGCAAACTTATCCCTTATGGTTTTCTGAATCAAAGAATCGGTGTGTGGATCCACATTCGCGGTAGCTTCGTCCATGACCAAAATCTTATTATTTCTAATAAGAGCTCTCGCCAGACAAACCAACTGCCTCTGGCCAACACTGAAGTTGTTTCCTCCTTCTGTAACTTCTGTATTGAGGCCTGCTGGCAGTTCGGATATTACATCTTTCAGTTCAACTTGATCTAAGGCGTTCCATAATACGTCATCACTGTAGTTTTCGAAAGGATCCAAGTTTTCACGCATTTTACCTGAGAAAAGCACTGGTTCTTGTGGTATAATCGAAATATTAGTCCTAACCAAATCCAACGGTATTTGCGTCGTATCTACACCATCGACCACAACAGTTCCTTCAAGTGGATAGAGTTGAAAGAGAGCCGTTATGGTTGACGATTTACCAGCCCCAGTCCTTCCAACTATGCCAATTTTCTCCTTGGGCTGTACTGTAAAGTTCAGTCCCTTAAGTACAGGCGGATCCTGTGGACTGTATCGCAAACTGACGTCTCTGAATTCAACAAGACCCTTCTCCGGCCACGGCTGAGGAATATTTTTAGGTTCTTTCCGTTCAGGTTCTGTTTCCAAACGGGTGTATTCCAAAATTCTTTCTACTGACGTCATGTTGTTTTCTAGTTCGGTCCATTGTCGCATTCCCCACTGGAGTGAGCCCATTAGACCCAGATACTGAGTGATGACTAAACCTAAATCACCTCCATGGATTGTGTCTCGGAACAACAAGAGCGAAAATACTGCCACAGCTATGAAGACAATACAGATTATATCTAACCAAAGTCCAAAACATTTACTGGAAGCCAAATATAAGAACCATGCTGCACTGTGGCTATCCTGGTAGTTGTCGAATTCTTCCGTTAACATTTTTTCAGCGTGGAAAGCCCTAACTGTGCTCAATCCATTAACTGAAGCTGTGAGGTGACTCAACATTGGACTTTTTGTAATTGCTTCAACTCTTTTAACACTTCTACTGGTTTCAGAATACACTATTTTCAATAAATAGAAAATTATCATTAGCACCACTGCAGGTACAGTCAATAAGGGCTCTACTATTGCAGATAGTGTAATAGCTCCAAGAAGAAGTAATGCAATCTCAATAACATCAATGATAACAGAAGGCAAATATTCATCCACAGTTCCTAGATCCTTCGAAAATCTGTTTAGGATCCTTCCAGATGGATTTGTGTTGTAAAATCTCATTGTCGCTTTGATTAGTTTGTCAAATATATATTTATGTAGATTCTTAGAAGCTATTACGAAAAAAATCATAAAGTACACTGCCTTGACTACCGATATGATTATGTTACCAACGATAAGGGCAGTGTAAGACCACATGATCAGATCCCTGTTTATAGTTTCATTGGGGGCGGTTAAATTCATTCTTTGCTTCTCGCTAAAATCTTGTTCTAAGTTCACCCAATAAGTAACATAGTATTCTCCGGCATTAGCAACTACTTGACAAATAATAAACAGGAAGCCCAGTGCTAACATCGAACAAATCCCACCGCCTGCTTTTAAGTACAAATAATAAGTAGCTGCCTTAATGGTACCCTTCTCCTGCATTTCCTTCTCCAGAACTTGGTCCTCTTCATCTTCCATTGGCTCATCGTAAATTGACGCTTTTGATTTAATTGACTTCATCCGTTTGTCTTCTTCGGCTTCCTCGTTAAATTCTTCCATAACTTTGGCGAAATCTAGACCGCTCTTCTTTAGTTCTGTGTAGCTTCCAGTCATCTCCATCTTACCATCTTTCATGATAATGATCTTATCGGCGTTTCTTAAGTACTGTAGCTGATGTGTAACCAGAACACATATTTTGTTTGACAAGAACTGTTTGATGCATCTATCGTAGAGATGTTTTCCTACGTTAGCATCAACAGCCGATAGAGGATCGTCTAGAAGATATATGTCGGCTTTTTTGTACACGCATCTAGCCAAATTCACTCTAGCTTTTTGCCCTCCACTTAGAGCTTTTCCTTTCTCTCCAACCAAAGTTTTATCTCCGTGGGGGAATAAGGCAAAATCTGATTTTAAGGCACATACTTCTACAACCAGTTTGTATCTTTCTTCGTCATATTCCTCACCGAACAAGATGTTTTGACGTACGCTCGCAGCAAAAAGCCATGGCTCCTGGGAGGCATAAGACACTTTCCCATCTATTTCTAGCTTTCCTGATTTTACTGGTAGTTCTTTTAGTATCAAGTTTATGATGCTGCTCTTGCCGCTACCAACTGGTCCAATGATCGCGAGCATTTTATTAGGAGGGACATTGAATGTTATGTCACTTAGGTTATTTTCAGGAGACTGTGCTAACCACTTGGCAGAAACTCCAGATAGCATCAATTTAGGCTCTTTGACTTTTTTAATTAATTCAATAGCTGTACCGTTTCCGTTAATACCGTTCTTCATGCCATCTAGTCCGTTCAGTTTAGATTGATAGTTACCATTCATCTTTTCATAACCATCTTCAGAGTCTTGTTCTCGTTCGTCAAATATCATTAGCTTATGTATCCTATCTATAGTAACGTGCATCTCGGCCAATGCTGTTATACTTAGAGAAAATATGATGGTAATGATTGTTCTCATCTGGTTGTAGATGGCGGTAATAGCAAAAACTTTATCGGCTGATACATAATTACCGAGAAGGACGTAGCCTACGATGCTCACAAATATAGCAGTTCTGGACACGAATACTTCGAATGAATAGAGTATTCCTAATAGGCAGGAGTGTGATCTGATTGCACGCATTTCGGATCTCCGTGCCAAATCAATAACATGAGCGAACGGTTTTTCCCAGCAATACATTTTAATCACCTGAATACCGGAGATAATCTCGTTCATTAGTTTGACCCTTTCGTCCGTCCTCAAAGCTGTTTTCAGACGCATCACAGACATTTTTTTGGCTAACCAAATTTGAAGCGGGACGAACGCCACTAGGAACGCCATACCAAAAAAGGCACTCACTCCAATCTCTCTATACAATAGCCACGTCCCAAGAGCGACTTGGATTGGCCCTATCCAGGCGTAATGAGTCAAACCAAATAACTGGTCAAATTTGCTTACGTCATTGGATAATAAATTTACTAATTGACCTACAGTAGTGTTTCCTAAAGCTTCTCGGCTAAACCTCAAAGTTTTCCTATATATCAAAGACGAGCAAGCCACCCGTATCTTCATGGCGATGTGCTGTAAGCCCATATAGTTTGGTTGTTGCATCACAGCATCTAATAAAAGTGTAATCACAAGTGCAGCTGCATAAATTAACGCCTCATCTTTCGTTATTTTGGTCTGTCCTGCTTCCAAATATGCAACTAGCGTTCGTATACAGTAAGGCATTACAACTATTAACAAGAGTTCGTCGAACAATCTGATTAACCCTAAGACCGTATATCTAAGTCCAAATATCCTAAATAAAGCCCTATGTAGTGCAGTCTTTTTGTGTATCCTATATTCTTCTTTCCATGCTTTTTCTAATTTTGATCCTAGTCTACTAGATGTGTGCTCCTTCAACGGAGAAAATAGTTCTTCTTCTGTTAATTTGTACTTGTACGTTTTTTTGAAGATAGGGAACATGTAGAAAAAGAAGAGAAAAGAAATCGGATTGGTATTTTCCGCAGGGTTGCGGGGCTTAGTGGTCTTGTTCCCCGTGTCCATTGTTGCTTTAAACACGGTATATCACAGTAAAATTCGTAAGTACACTTCGTATGTGTTTCGAGAGTCCGCGAACTGCAGAGCGGACAATCGCACACATCTGTCCCAAT

>Dvv ABC-C D12562+D20321

TTCAGTGCGTCACAGTTTTTCGATTTCATTCTAACGCATTAAATTGTATGTGACAGAAAAAAACGAAAAAATCTCGAAATGGAGAACCTTTAGTTTCAAATAACTCAAATGTGGTGCAATTTTTTGGGAAAACTTAACAGACATGTTTAGAAGTTCACTAAAAAACCTTTCAAATGAGCTCGGATAAAAGCTTTTTTTGCATAAGAACTGACTGACTTATGACGAAAATAAAGTCGATCCCTGCTTTTTTTCGGAATGTAACAATTAAACACTCGTCATTACAATCCTAATAGAAATGAATAGCTTCCCTCTTCAAATTAACTTTATTTAGGTATAGTTGATACGATCCACGTGATTTGACCGGTTTAGAATGCTTAGTTTAGAAAAAATTGTTGATTCGATCGAAAATGACATTATTATAATTTAAAAAAAAGTGCATTTTTCTTAAATAAATCTAAAAGTATTCATAATATGCAACAATAATTTAAATATTCATGGTAGATTTTGTTTACTGAAAATTTTGATTTTTCTTTTTGAATCATGAATACTTCCGACCTCCATATCTATCGACTTCCATAGTTATTTTGATTTTCATTTCCTCGATGGGATTGTTTTTACCCCCAGGACAAAATCTACTAGAGGGTAATTTTTGAAAAAGAGGGTCAACCGAGCTTACATCCAAATTTTCATTAAAATCGGTGTTGATGCTTAAAATTCCACGGTTTTACAGTTTTTTAGCGCTGATTACTGCTCTAATAAATAAAAAAAGTTTCCTATAGTTTGTCCTAAAATATAAGTAGCAGCCCCTCATAAGTTTTTAAATTTACATATCTATAGGTTTGTTACACTAAACTAAGCACCATATTGTGATGTCATAAGCGTTTAAACACTGGTCAATTATTCAAGAATTCCTACCTATCTTATATACATACAACTAATAGGAACAATGTATATGTTACATTAATACAAAAATACTTTCACTGGAAATTTTAAATTCTCTATTATGTATATTTAAAGAGAATTAATTGGAGATAATTAATTAATTATTAATTAATTTCAATACTAAAATGTATGCTTAGTCTCTATTTAGTTTTCTGGCTTTCTTCTGCTATTGACATTAGAGTTTGAGCCGTCCCCTTGCCCGTCTTCATCACCAATGAATAGAAAGTTCCTTGTTTATTCTGCAATAGCGAATACGGATGATCAAACTCCACCATTTTCCCAGCATCCATCACCAAAACTTTATCTGAATCCATTATAGTATGTAATCTATGCGCAATCGTCAACACTGTACAGTTTTCGAATTTCTTCCGGATGGTCATTTGGATCAATCCATCTGTGTGGGGATCTACGTTCGCTGTGGCTTCATCTAATACAAGAATTTTGTTGTTTCGGATTATAGCTCTTGCTAGACATAGCAACTGTCTTTGGCCTACACTGAAATTGGAACCTCCTTCCGCCATTTTATTGTCCAGACCAGCTGGCAACTCTTGGACGGCGTGTTTCAGTTCAACTTCTTCCAGGGCGCTCCACAGAACCTCGTCCTTGTATTCGTCAAAAGGATCCAAATTTTTCCGCAACGTTCCAGAAAACAGCACGGGCTCCTGCGGTATAATAGAAATATTTGACCTAAGTTTTTTCAAAGAGATAGTCTTCGTATCAACACCATCGATAAGAATACTGCCTTCAATGTGGGTTAGTCGGAACAAAGCTTGAATTAAAGAAGACTTTCCAGCTCCAGTTCGTCCTACTATACCAACTTTTTCTTTAGGTTTGACCACAAAAGTCAGGTTTTTCAACACAAATGGATCATCAGGAGAATATTTCAACGACATGTTGCTGAACTCTACTTCTCCTTTTTCGGGCCAGGTTTCTCGTGGTTTGTTCGAGGGTTGGTCTTTTTCTTGCTTTAGATCTGCGTATTCTTGTACTCTTTCCACAGACGTCATCTGATTTTCAAACTCGCTCCACTGTCGCATTCCCCATTGGAACATGCCCATTAAAGCCATAGCCTGTGAGAGTGCTAGACCTATATTACCTCCAAATTGCTCACTTTTAATCAATAATAAAGCTGCCAGTACCAATCCAATATAAACCACACAAACAAAATCAAGCCAAAATCCAAACGTTCTGTTAGCACCTGCGAAGATGAAATAAGCCGAAGTATTCAAATTTTGATAATTGTCAAATTCCTTTTTGAGTATTTCTTGGGCTTTAAAAGCTCTAATTGTTGTCAGACCTTGTAATGATGCAGAAAGATGTGTGAAAATTGGACTTCTGGTAACAGCTTCTATTCGCTTAACATCTCTACTGGTTTGGACGAAAACTATCCTCATTAGGTAGAACAATACAGCAAGCAATACAGTTGGTATAACAATCCATATGGTTAGACTGCCAATTACCAAACAGATAGAGAAAACATATAAGCCAATCTGAACAGTATCCATAAGAGTCATTGGTAAATTTTCATCCATAACACCAATATCTTTCGAGAACCTGTTTAATATTCTTCCAGAAGGATTGATGTTGAAGAACCTCATAGGACTGTAAATAATATTCTGAAACATCTGGTTATGTAGTGTTGTCGACGATGTTAAGGATAAATGATAAAAATAAAGTGACCGTCCCAAGATCATGATGATCAAAGATATTACTACGAGTAAGTAGTAGATGGCAGTATATTCGCTGGTGAACAAGGGCGTTAACCACCAACCTTCGTACGGGTTTTCAGGTTGAACGGTGGTTCTCAGCGCTTTCGGTAAAGATGAGTTGGATGTTATATTTAAATTTGTTGTTATATTTGAATTGGATGTAACTGTTTCAGTCAGGTTTTGGGAATTCACTTGTTGAATGTTGACCCAGTAAGTAACGTAGTACTCAGAGAAACTCTCCATAAACTGACTGGTAATAAAAATCATACTGATCAGAAATATATGGAAAAGGTGCCCCGAAGCTTTAATATAGTTCATATACACACGACCAGAAATTTTGCCTGTACTTTGAGCCTCCTTTGCCAATATTTGAATTTCGTTATCTTCGAACATTTCTTCCTCCCTAGAAAGCGTTCTTGTTCTCGACATTTTCCTAGTTTCCTCCTCGTCCTTTATATCAGTAAGAAGTTTACTGTATTCTGTATTGGAATTTTTTAAATCCTGATAGGTACCAGACAATTTAACCTTTCCATCTCTCATTAAGTATATATGTTTAAGTTTTTTTAAGTACTGTAACTGATGAGTTACTATAACTACACATTTGCTACTAAGATACCCGGTAACACAGTCTTCGAATAGTTGTTTTCCAACATGGGCGTCAACTGCTGATAAAGGATCATCTAGTAGATATATATCAGCGTCTTTATACACAGCTCTAGCTAAATTAATCCTAGCTCTTTGTCCCCCACTAAGTGACACCCCTCTTTCCCCAGCTAAAGTCCTGTCACCATGAGGAAATAAAGTAAAATCTCTTTGTAACGCGCATACTTTAACTACTTCGTCGTATTTACTTTGATCGAACTTTTGTCCAAAAATAATATTTTGTCTGATACTACCTCCAAATAACCAAGGTTCTTGTGATGCGTAAGAAACAACCCCGTGGACGTCCACAGACCCTGACTGTAATTCCAGTTCCTTTAAAATAATGTGTAACAAAGTAGTTTTACCGCTACCTACAGGACCCACAACAGCCACAACATTGCCCGGAGTTACGTCCATGTTAATTTTCTCTAAATTATTTTCAGGAGAAGATTTTAACCATTTCGCTGAAGCGTTTTTTAGGTGTATACCTGGTTCTTTGTTTCTAATGGTGAGTTCCAACGATTTAATTCCGTTCTCTTTGTGTGATTTTTCGTCTAATGCTATATAGTTCTCATCCTCGACTTCGTCGTATAATAAAAATGTTTGGATTCTCCGTGAGGAGACATATAACTCCGCAGCTTGGGAAATGGCCGTGGGTAAGAAATTGGTAACTGTATACAAAAGCCTGTAGTATGATGTTACGGTATAGGCATAGCTAGCAGTTAAGGCGTTTCCAGTAAATACGTATACTAGTATGCTAGCTGCTATAGCACTCCTGTGAAGCATAATGATACAAGACATTAGCACCGATCTGATCACTGAGGTATATCGGATGTACTTCATCTCCTTCTTTCTAGAAAATTCTACTAATTTAGCAAATGGTTGTTCCCAAGTGTACATTTTGATTACTTGTATTCCCGCTATAATTTCGTTCATCAGTCTTACTCTCTCGTCAGTTCTGTTGGCTGTTTGAAGTCTAAATTGTGATGTCTTCTTACCCAACCAAGATTGAAATGGAATAAACAATAATAATACTATAGCTCCTGCTAACGCAGCTCCTCCCACGTTTACGTACAATAAGTACATAACAATTAATATTTCTATCGGGCCTATGTATATGTTATGGAAAAAGTAAACAGCCAAGTCAAATCGGGACACATCGTTTGAAAGCAAGTTGACCATTTGCCCTATTGTAGTTTCAGCCAAAGCAGACTTGCTTAATTTTAGAGCTTTTCTGTATATGAGGGAACATGACGCCACACGCATTTGCATACCTAACTGCATAAGTCGTAAATTAAAGTTGTGCCCCATGTTGGCTGCTACAAAGGAAGTTAGGACGATTAAAGAAGCATACAGGTAGATTTCACTTCTTTCTTCATCTAGCTGCTTGGACTCATACACTTTTAAAAGTTCTGCAATGAGGAATGGCTGAGATATCCTTACGACCTCTGCAATGACTACGAGACAATTGATACCTAATAGATCTAAAACAAAACTAGAGGCACATGCTTTTATCAATGACGGCTTCTTCTTTCGTAGTTGCTTATTCCATTTTACTTGCAGCTGGTCACCTAACTTGCCCGATTCGTGGCAATTTCGATGTTCATACATATCATCTTCGGTGAGATCTTTCTTGTAACCTTTCACAAAAAATGGAAGAAGCCAAAAAAAAAATATTGATGAAAATATATTAGCAGTGTCTAAAGGATTTTTCTTCTTTGCTTTGGCTTTGCTGTGTATTTCTTCCATATTTAAGTTGTGTTGTTATGTACATGTAGTCCTCTTTATAGATATTACCGAACTGATTCACTTCACTTTAAGGGTTAAATTACTTGTCACACTATTTTTTATAATTAGAGGGATTCCTATATTCTTAATACATTTTTTTAACAATTTTGTACATTCTAAC

>Dvv ABC-C D15305

CACATAACCWTATGCCCGATGTGTGTAATTTCACATTTCTTTGTTATATTTTCTTTTGGAATGATGTACTAAAGGGCCGGTTGTTCGAACGCCAATCAACAATGATCACTATCAAATAATTATTTACTGTCAATGTCAACTTTGTTTGGGTTGTTAAAAAGTCTGTGGAGTCTATAATCAATTAATATAACAATARTTATTAACATAATTAATAATAAATCTCATAATTRTAATTAATTATGTTTTYAGCAACCCAAACAAAGTTGACATTCGACAGTTTTGGTGACAGCAATTAAATATTTGACAATAATCATTGCTGACTAGCGTTCGAACAACCGGCCCTAAGGTATTAACGAATTCATTAAACTCAATATATCGGCACATAGAACCTAATAAAAAAATTTGTAAAATGGTAATCTTGAACATAAGATATGCGCCTTAGTTAGCAGTTATCGCATTGAGTAAAATATAAGACTAGAGTTTAAAATTAATTAACCTTAATATAATTAACGTTAAAATAAATCAATGTAACATACTTAATGGCAACAAAATTTTTAACAATCTAATTGTTTTTGTACTCTTTGATTTAGTAATTTTTACTCTGGTAATGCATTTAGTTTTTGATAACTATCTGAAGCAATTTTTCTTAGCTGTTCAGCTGTTGTTCTACCAGTTTCAGCAACCATACTGTAAAAAACTCCGTTTTTATTTTGAAGGAGTAAGTGAGGGTGATCAAATTCAGATATTTGTCCAGCGTCCATAACTAATACTTTATCAGAGTCCATGATTGTGTTCAGTCTATGTGCGACGGTTATCACAGTGCAATCTGAGAACTTTCTTCTGATAGTTTTCTGGATTAAAGCGTCAGTCTGTGGATCTACATTAGCCGTAGCTTCATCCAACATAAGAACTTTGTTATTTTTAAGAATAGCTCTTGCCAAGCAAATCAACTGTCTCTGTCCAACACTGTAATTGGAACCTCTATCCATGACTCTATTTTCCAATCGGTTAATAACATTAGCAGGATCTTTTAGTTCTACGTCTTGTATAGCTTTATACAAAACTTCGTCTGAATATTCTTCGAATGGGTCCAAGTTGTATCTTAAAGTTCCTGAAAATAGCACTGGGTCTTGGGGAATAATCGAGATTTTTAAACGGAGATCATTGAGGCTAATGTCTTTGGTGTCGATGTCATCGATTTTAATTGAACCTTCAATTGGGGCTAGTCTAAAGAGTGCCTGAATTAAGGAAGATTTTCCTGCACCGGTTCTGCCAACAACTCCAACCTTTTCCTTTGGTTGAATTTTAAGATCAAGATGTTTCAAAACCAATGGTCCACCCTCAAAGTATTTTAAACAAGTATCTTTAAAAGTGATTTCTCCTTTCTGAGGCCATTCTTTTGCTGGTTTTTGAGGTACGACCGGTTGTGGTTCTTTTTCCAAATTTTGGTACTCCAATACCCTTTCCACGCTCATAAGTTGATTGGTTACCTCGGCAGATTGACGCATACCCCATTGTAGTAAACTTGTTAATGCTGTTGCTTGAGTGATGGCCAGACCTACTTGACCTCCCTTCAGATTAAAGGCCTCCCCAAATAATAGTAAACTAAAAGTAAGTGCTGCCAAAAAAAGAGAACAGAAACAGTCCAAATAAAATCCAAATGCATTACTGGAAACTATAAACATGTACCATGCACCTGAATGATAATCTTGGTGATGATCAAACTCGTCCATAAGAGTACTTTGGGCTCCAAAAGCTCTTATGGTTGTCAAACCTTCAATGGTAGCTCTCAAATGGGTAAACACGGGACTTCTCATTATTCCTTCTAACCTCTTAATATTCTTCGAAGATTTTAGGTAGACATGCCTCATAAAACCAAAAACTACACTCAAGGCGCCAATCAGAATTAAGAAGTAAGGATTGACTACGACTATCAGTAAGAGAGATCCACACATAGACAAAAGCATCTGTGAAGAATCCAATATTGCTTTCGGTAGTAATTCGTCAACACATCCCATATCTTTAGAAAACCTGTTGAGAATTCTTCCTCCTGGATTGGTATCGAAGAATCTCATTGTAGCAGTGACAATATTATCAAAAATTGTCGCATGCAGTTTTTTGGAGCTCCACATAGCAAGTTTATAAAAGAAGAAAGATCTTGTCATAGCAAGAACAAAAAGAGCTCCTAAAAGGAACCCATATATATAAAGGCAATTATTTGTTGTCAGTTCAATGGATGGAATAATGAATGTAGGTGTAGTAGAATTAGAAGATTCGCTGGAGCTCCTGAATTCCTCTACGTTGACCCAATAGCTTACCCAGTAGTCTACCCCACTGGCAGCTAATTGAGTTCCTAGATACAATATAACGCATATTAAAACAGCAAATGAATTAGAACCTGCCAACATATATTTAAACAACAGAGAACCGCCCACTTTTCCTTTTGATGATTGTTCCTGAAGGTCTTTGACATCAAATTCGAGTTGCTTTTCAGCTTCTTCTTCTTCAAAGTCGATATCATTCGATATGAGAGTATCCGCAATGCTTAATTCACTGACTATACTTGCCAGTGAATCTTTTCGACTTCTTATTGAGAGTTGCCTATTATATTTAGATCTATCTTGTTTCTTTTCTTCTACATTTTCTGGCTCAGCAGTTAAAAGTTTTGCATAAACATTGTCACTGTTTGCCAAAAAATTGAAGGTTCCTTCATCTTCTATGCGTCCATTATTTAAAATAATAATGTTATCCGCATCTTTCAAATAGTGTACTTGATGGGTAACTAAAATTCTCGTTCTGTTGGCCAAATATCCATTAATACATTTTTCATATAGATGTTTGGAAACGTGTATGTCCACAGCTGACAACGGATCATCCAAGAGATATACATCGGCTTCTCTATACACAGCTCTTGCTAAATTTATTCTAGCTTTTTGGCCGCCACTCAAAGATGATCCCCTATCACCTACTAGTGTTAAATCTCCATTTGGGAACTGCTCAAAATCTTTCTCTAAAGAGCAAGCTTCTATAACTTCATGGTATCTCTTTTTGTCGTATTCTTCACCAAAAAGAATGTTTTGACGAACAGTGGACGCAAAAACCCACGGCTCCTGAGAAGCATAGGATATTTGACCTCGAACTCTCATACTGCCTTCGGTTATGTCCAGTTCCCCCAGTAATGTTTGCAATAATGAACTCTTCCCGCTACCAACAGGTCCAATAATTCCAACCATCTGTCCATCAGAAATATTTATATTTATATTCTCAAGAGCATTGTCACTGAAGCTTGCATTCCATTTTACAGTCAAATTTTGCAAACTTATAATTTCTTTAAAATCGTCTACACTGTTTACATTGCCATTCATAGCGAAATTTGTTCTATTAGGATCGTATTCTTCGTTTATGAGAAAATCTCTAATTCTTCGAATAGCTACCATAACCTCTGCCATTTCAGATACTCCTCTACTAAACATGGTGGACATAGCCATTGAAAGAACGTTGAAATAACCCATTACAACAAAAACCTTCGACGCTGTGATTTCTTCGTTCGACAAAATAACTGTCAGCAAAGTACAGAACAATGCCAATCTGGTGGTGAAAAGATTCAATGCCATAAACGTAGCTCTCACATAAGCAGATTTTGTAATAATTTTTATTTCATTTCGTCTGGCAATTCGGATTATTTTCCTGAACGGTATTTCCCATGCGTACATTTTGATAACTTGAATTCCTGAGATGATTTCGTCCATTAGTCTGACTCTCTCGTCTGTTTTGAAAGCAGTCTGTTTTCTGTATATTGCAGATAATTTTCCGGTGTAGGTTTGAAAGGGAACTATCACAAATACTGCCACAACTCCCACTACTCCTGCATACCCAGTTCTCTGGTACAGTATACCCATTACTATAAGTGACAATACAGGTGCCATCCATAATTGGTGAATGAATAAACTAACAATATCAAATCTACTGACATCGTTGGATAATAGGTTGACCAGTTTTCCGGATGCTGTTTCTCCCAAGGCTGTTTTGCTCAATTTTAATGACTTTCTGTATATCACAGCACAACAAGATGCTCTTATCCTCATGCCACTGTGAAAAACCTCTAGCATATATTGATTTGACATCAAAATGTTCAAGATGTTTATGGCCACTAGGATACCTGCATACGTAAGAGCCTCATTTTCGGAAACTTGTGATTCTGGTTTAAAATAATCCAATAGGTTCCCAAGTATTAGAGGTTGAATGAGTCTTAGCACTAAATCTGTCGTGATAGTATATATTCCTAGCTTTGCGTACTCCAACCAAAATGACATAACAATGGCTTTCAATAAACTTGGTTTGGAATTTGATTTCTTAGATTTTTCTAGTTGCTTGTCCCAATTTCTTTGTAAATTATCTCCCAAGACTTTGCTTCTGTCACTCTTTAACGGATTATATAAATCGTCAACTTCCAGCGTCTTCGATAAGCCCTTCTTAAATACATCATACGTATATCCAAAAAATAGAGCCGATATAATATTCGCAGTCTCCCGTGGATTGGGATTATATTTCTCTTTGGTCATTTCCATCTTTGCGATTTCTTAACTATTCTAATTTTCTAAGTAAGTATTCGTAAGTAGTCACAGGACAATTTTTTGTTTTTAATTTAACATGAATTGGTTATCTCGGGAATTGATACAACCGCTATTTAAGATATGTTTATCTATCATTAGTGTACATGTTC

>Dvv ABC-C D21941

CGGACTTTTCCTTTTGACCACTTCTTCTTTTTGGCTTTGCCACCTCCAGACCCCTCTTTCTTCTTCTGGGTCTTTTGGGGTTGCTTCGCCGATGACTTAGAGTCCTTCTTGGGAGGCATSTTGCTCATCCAGAAATAACTGTCTATAATGTTCCTCCGCTATCTTTTTAAGCGCCTTCTCAGTCGCTGTCCCAGTTTCTTTTAACATTCTACTGAAATATCCTTCATAATCTTTTAACAGTATGTGCGGATGCTCGAATTCTACAGCTTGACCGGCGTCCATTACCAAAACTTTATCGGAATCCATTATTGTATTTAGTCTATGTGCTATGGTTATCACAGTACAATATTTAAAGTTCTTTCGAATTGTCTTCTGAATTAAGGCATCGGTACTGGGATCAACATTGGCCGTGGCTTCATCTAAAACAAGTATTTTGTTGTTTCTTACAATCGCTCTAGCTAGACACATTAATTGCCTTTGACCGGTACTGAAATTTGAACCACCCTCCCGTATTTCCATTTCAAGGTCAGTAATCGAATGTTTCAACTCTACCTTTTCTAATGCATCCCATATCTGCTCATCACTAACAGTGTTGAATGGATCCAGATTATATCTAACAGTCGCTGAGAACAGAACTGGTTCTTGTGGTATTATAGAAATTGTTGATCTCAATTTACTAAGCTCTATATTGACAGTATCCACGCCATCAACTTTTACTGATCCTTCTAATGGTGAAAGTCTGAACAATGCGGATACAAGTGAAGATTTTCCAGCACCAGTCCTGCCAACTACACCAATTTTTTCTCCAGCTCTAAACACAAGATTTAAATTCTTTAGAACGGGAGCACTTTCTAATGTGTACCTCAGGAAAACGTCTTTAAACTCTACTTCTCCCGCTTGTGGCCAATCTTTATCTAGAGATTTATATCTAGGTATTAAATTATTCACTGACAGTTGTTTCTTTCCTTCTGTTTCTTTTTCCAAAGTTGTATATTCAAGTACTCTCTCTACACTGGTCATATTGTTAGCTACTTCTGCAGTTTGGCGGACGCCCATCTGTAACATTCCTGTTAATATCAAGCACTGGGATAAGACAAGGCCAGCGTTTCCTGCAGAACTGGGATCTGGGTTATCAAAAATAAGGAACTGGTACGTAACTATAGCTATAAAAACTGTACTTATGCAATCAAGATAAAAGCCAAATACTTCACAGGAAGCAATATAGAGGTACCAAGCTCCTGAACTATTGTCCATAAGTCCGTCAAATTCTTGGATAACCATATCTTCGCACTTAGATGCTCTTATGGTTGTCATTCCGTCTAGGGTGGCGATAACATGGGAATATACTGGAGCTCTACTGACTCCTTCCAGGCGCTTCACGTCTTGAGTCGTTTTTAAAAAGAACAATCTGAGATAATAGAACAAAATTCCTATGACTACTGCTGGTATTATCATCCACAATATTTTAATAAACACCAGTACCAGGATACCTACAAGGACCAACAAAACTTGTGTACCATCAAGCATCGCTTTTGGTAAAAGCTCGTCAATGATTCCCATGTCATTYGAAAATCTGTTCAGGATTCTTCCTGAAGGATTCGTGTCAAAAAATCTCATAGGTGCTTCCAAAATCTTACTGAACATACTGTTGTGCAAGTTTCTTGAGGCATTCATAATAATTCTGTAGAAATTTAAAGATCTGATTGGAGTCAGAAAAACAGCTAGACATATTGTGACACTGTATATAATTATATAATAATTTGAGTCGTGGGTAAACCGTTCTTCAAGTGGAATATTTTCACCCACTACCGAATAGTTGTACTTGCTGTAATTTCCAGACGAGGTATTATTTGCAACAACTATTTCCAAGGGTGGTGTTGTTGTACTATTAGCTGCCACTGTATTTACCCAATAAGTCACCCACAGGTCTCCTGCATTAGATGCCGCTTGGGCCAAAACGAATAAAATTACCATAAAACAAAATCCAAAACAACTGGTACCGAAACGGAAGTATTTGCCATAAGTTGAAAAGGGAATTGATCCTTCGCAGGTTTCTTCATTATTTTCCTTGGGCCCTTCTTCATTTTGAGATAACACCTGTTTTATTTCTTCCATATTAGACTGTTTTCTAGTATTTAAAGATACATCCTTTTTCTCATCTTCAATCTGTTCTTCACTTCTATTTTCTTGCAATGACTTTAGGTAATCCTCGGACAGTTCGTCAAAGGTTCCCATTTTCTTGATTTTTCCCTTTTCGAAAACTATAATAACATCAGCATCTTTTAAAAACTGCAACTGATGTGTCACCAAAATCCTAGTTTTACTGCCCAAAAATCCTTTAATGCATTTTTCAAAAAGATGTTTACCAACTCGTGTATCCACCGCAGATAAAGGATCGTCTAGTAGGTAAATATCAGCGCTAGTGTACACAGCCCTCGCTAAGTTTAATCTTGCTCTTTGACCACCACTCAGGGAAACCCCCCGTTCTTCTACAATGGTTTTATCGCCAAAAGGAAATTGCTGAAAGTCGGTTTCTAGAGCACATACTTGAACGACTTCGTCATATCTGTTTTTAATGTAAGGTTTTCCAAACAATATATTTTCTCTTACGCTAGATACAAATAACCAAGGTTCTTGGGAAGCGTAAGACAAGTCTCCTGACACCTCTAATTGGCCACTTTTGATTGGAAGCTCACCGAGTAACATTTGCAGTAAAGAAGATTTGCCACTACCCACCGTCCCCACCACGCAACATAGTTTTCCTGCARTTAACTTTAGATTTAAGTTATTCAGAGTGTCATCCTTCAAATCCTCATTCCAAGTAGCACAAGCTTCTCTGGCTTGTATGGTTCCTATTTGCTGGACCACAGTATTTTGCACCTGAACATTATTTTCTTCCAAAAGTAAAAATTGTTCGAGTCTTCTTAAAGAAACCTTAACTTCGGCATATCCAGCCAATGCATAAGGAAACATGATGCAACTGTACAATTGTAAGGAATTGTATAATTGGGCGCTAAGGAAAACTTTGTCAGCCGATATTTGATGTCCTAGCAGAACAAACGTTATGACTGTCAGGTATAATGCAATTCTCTCAGTAACTACTATAAATGCAAGTGACAATCCCTTAATAGCTGACGTTTTAGATATTAAACGTACTTCTTCAGTCCTTGCGGTTTCCACAACCTTTGCGAAATAATTTTCCCATGCATACATTTTAATTACTTTAATACCTGCCACTAACTCATTCATCAATTTTACTCTAAAATCAGTCTTTTCTGCTATTTTTGAACGAAGTGTCCCTTGAAGTCTTGATAAATAACCTTGCATAACAATYCCTTCGAAAAGCATAAATGCTGTTCCCGCCAGAGCAGCTATTCCAACACTTCGGTACATAATGTAGAAGCAAATAGCAGCTTGGAATGGCATTACCCAAATAAAATTTAAATACATTGAGGCAATATCGAATCTTTGTAAATCGTTGGAAAGAAGATTTACCAATTTACCTCCTGGGGTTTTGTTTAAAGATGCTTGGCTCAATTTGAGTAACTTTCTATAAACTAACGCTGAACATGCTGCTCTTGCTCTCATTCCAATTCTTTGTGATCCCAAAGTTGAATGGTGATAAGAGAGAGCAAATATAAAGGCTAAAAACACAGTGGCTGTTCCTAATATCCATCCTGAATACTCACCGAACATGTCGTACTTTTGGGTTTTGTCGAAATATTTTAATAGTTCTGCAAGAACCAGAGGTTGAAGTGTCTTTATTAAAACAACTTGCAGAAATACCATTACTCCTTGTAATGAATATGGAAAGGCGTATGTTTTAAAAAGTGCCAATTTCAAACTAGGTTTATCCTTTTTTCCGGCTTTGTGTAACATTAATTCATTTTCCCAATGTCTTTGAAGCGTATTGATTGTAGCGTGACATGTGTCTGAGTTGAGCGTGTTGTAGATATCCGACGGTTGTAATTTTTTCTTAAAACCAGTTTTAAAAAGTGGAAATATCCAGCAGAAAAATATTTTTGATAAAAAGTTTGCCGATATTTGAGGTGATGGATTGTTGTGTTTTTTGGTAGAATCCATTATTAAAGGAAACAATGTCTACCTAAGAATTCTTCTGAATTTAAATTTGAAGCACTATCTAGAACAGCGTGTAAGCACTTTTCGCAAGTTTTATGGCTACG

>Dvv ABC-C D18709*

ATTTAACCTGTGTGCGACGGTTATCACAGTGCAATCTGAGAACTTCTTTCTGATAGTTTTCTGGATTAAAGCATCAGTTTGTGGATCCACATTGGCTGTGGCTTCATCTAACATCAGAACTTTATTATTTTTCAGAATTGCTCTTGCTAAGCAAATTAACTGTCTTTGCCCAACACTGTAATTGGAACCTCTATCCATGACTCTATTTTCTAATCGGTTAATAACATTAGCAGGATCTCTTAACTCTACATCTTCTATAGCTTTATATAAAACTTCGTCTGTATATTCTTCGAATGGGTCCAAGTTGTACCTTAAAGTTCCTGAAAATAGGACTGGGTCTTGTGGAATAATAGAGATTTTCAAACGGAGATCATTGAGGGTAATATCTTTGGTATCGATCTCATCGATTTTGATAGTTCCTTCAATTGGAGCCAGTCTAAATAGTGCTTGAATTAAAGAAGATTTTCCTGCACCTGTTCTGCCAACAACTCCAACCTTCTCCTTGGGTTGAATGCTTAGGTTAAGATGTTTCAAAACCAATGGTCCCCCTTCAAAATACCTTAAACATGTATCTTTAAAAGTAATTTCTCCTTTCTGTGGCCATTCTTTCGCAGGTTTTTTAGGACCGACTGGTTGTGGTTCTTTGTCTAAACTTTTGTATTCTAATATTCTCTCTACACTCATAAGTTGATTGGTTACTTCGGCAGATTGGCGCATACCCCATTGTAAAAAGTTTGCCAGTGCTGTTGCTTGAGTAATCGCCAAACCTACTTCACCTCCTCTTAAATTAAAAGTTTCTCCGAATAACAATAAACAAAAAGTAAGTACTGCTAAAAATATTGTACAGAAACAATCCAAGTAAAATCCAAATGCAGTACTGGAAACTATAAACATATACCATGCACTTGAATGATAGTCTTGATGATGATCAAATTCGTCCATAAGAGTGCTTTGCGCTCCAAAGGCTCTTATAGTTGTCAGACCTTCAATTGTAGCTCTTAAATGTGTAAAAACTGGGCTTCTCATTATTCCTTCTAACCTTTTAATATTCTTAGAGGATTTTAAATATATATGTCTCATTACACCAAACACTGCACTTAAAACTCCTATCAAAATTAAGAAGTAAGGGTTAACTACAATAATCAGTACAAGAGATCCACACATAGATAGAAGAATCTGTGAAGAATCCAATATTGCTTTAGGCAATAATTCATCGACAGCTCCCATATCCTTAGAAAACCTGTTAAGAATTCTTCCTCCAGGATTTGTGTCGAAGAATCTCATTGTAGCATCAATAACATTATCGAAAATTGTGCCATGAAGTTTTTTCGAGCTCAACATAGCCATTTTATAAAAGAAGAAAGACCTTGTCATAGCAAGGACAAAAAGAGCTCCTAAGATAACTGAATATATATAAAGGCAATTATCTGTTGTTAATTCGATGGATGGGATGATGAACCTAGGTGCAGTAGAATTCAAAGATTCAGACGAGTTTCTGAATTCCTCTACATTCACCCAGTAACTTACCCAGTAATCCGTCCCACTGGCAGCTAACTGAGTACCGAGATATAGTAGAACGCATATGAAAACAGCAAATACATTAGAACCTGCTAACATGTATTTAAATAACAAAGAACCGCCCACTTTTCCCTTCGATGATTGTTCCTGAAGGTCTTTGACATCAAACTCTGGTTCTTTTTCAGCTTCCTCTTCGTCAAAGTCGACATCGTTTGATAAAAGAGTATCCGCAATACTCAATTCACTTATTATACTTGACATTGAATCTTTTCGACTTCTTTGTGATAGTTGCCTAGAATATTTCTGTCTCTCTGGTTTCTTCTCTTCCTTATTTTCTGGTTCAGCTGTTAAGAGTTTTGCGTACACATTGTCACTATTTGCCAAAAAGTTGAAAGTTCCTTCATCTTCGATGCGACCATTATTTAAAATAATGATGTTGTCAGCATCCTTCAGATAGTGTACTTGATGGGTAACTAAAATTCTCGTCCTGCTTGCCAAATAGCCATTAATACATTTTTCATATAAATGTTTGGAAACATGTATATCTACAGCGGATAACGGATCATCCAAGAGATAGACATCTGCTTCTCTATACACTGCTCTTGCTAAATTAATCCTAGCCTTCTGACCACCACTCAAAGATGATCCTCTATCACCTACGAGAGTTAAATCTCCATTTGGAAACTGTTCAAAATCTTTTTCTAAAGAACAAGCTTGTATAACTTCCTGGTATCTCTTTTTATCATATTCTTCACCAAAAAGAATGTTTTGACGAACAGTGGCTGCAAATACCCACGGTTCCTGGGAAGCATAGGAGATTTGACCTTGTACTCTCATTTCGCCTTGGGTGATGTCCAGTTCACCTAGTAAGGTTTGCAATAAAGAACTCTTTCCACTACCTACAGGTCCAATAATTCCTATCAACTGTCCATCTTGAACATTTAAATTTATATTCTCGAGAGCATTGTCGCTAAAACTTAAATTCCATTTTACAGTTAAATTTTTCAAACTGATAATTTCCTTAAAATCGTCTACACTTTTGACATTGCCGTTCATACCAGAAGTTGATCTATTGGGATCGTACTCTTCGTTTAGAAGGAAATCTCTGATTCTCCGAACAGCTACCAAACATTCTGCTATTTCTGATACTCCTCTAGTAAACATGGTGGACATAGCCATTGAAATAACGTTGAAGTAAGACATTACAACGAAAACCATTGAGGCTGTGATTGGTTCGTTCGACAAAATAACAGTAAGCAAAGTACAGAATAACGCCAATCTAGTTGTGAACAGGTTCAATGCCATAAATGAAGCTCTCACATACGCAGATTTTGTAATGATTTTGATTTCATTTCGTCTAGCAATCCGTATGACCTTCCTGAACGGTATTTCCCATGCGTACATTTTGATTACTTGAATTCCTGAGATGATTTCGTCCATTAGCCTGACTCTCTCGTCTGTTTTCATAGCTGTCTGTTTTCTGTATTTTGCAGATAGCTTTCCAGTGTATGTTTGAATAGGAACTATCACAAAAACTGCCACAACCCCAACTATTCCAGCATATCCAGTTCTCTGGTATAATAAAACCATTACTATAAGTGACAATACAGGTGCAATCCATATTTGATGAATGAGTAAACTGACGAGATCAAATCGACTGACATCGTTGGATAGGAGGTTGACCAGTTTCCCTGAAGCTGTTTCTCCCAAGGCTGTTTTGCTCAATTTTAAGGACTTTCTATATATAACAGCACAACAAGCTGCTCTTATCCTCATGCCACTGTGGAAACCCTCTACCATATACTGATTTCCGATCAAAAAGTTCAATACGTTTATAGCCACTATGATTCCTGCATACATGAGTGCCTCATTTTTTGAAACTTGTGCTTCAGGTTTAAAATGGTCCAACAGCTTTCCAAGCATTAATGGTTGGATGAGTCTAAGGACTAAATCTGTTGTTATGTCGAATATTCCTAACTTCAAATACTCTAACCAATATGTCATAAGAATGGCTTTCAGTAAACTTGGTTTTGAGTTTGTTTTCTTGGCTTTTTCTAGTTGCTTGTCCCAGTTGCTTTGTAAATGATCTCCCAATATTTTGCTTCGATCGATCTTTAATGGATTATATAAATCGTCAACTTCTAGAGTTTTCGATAAGCCCTTTTTAAACACATCATATGTATATCCAAAAAATAGAGTCGATAAAATATTCGCTTTCTCCCGTGGATTGGGATTATATTTCTCTTTGGTCATTTCCATCTTTGCGGCCTGGCAATTCCTCTAGTTGCTACTCAAGTATATCCTGTAACTACCCTCGTTTTCAATTTCGCATGATATTTTCCACTGGACTGCATAAAGATCGCCAAGATTTATGTGTATGCTTCTATGTATTATATCACTACAACCAAATCAAGTGAGGTAGGTACCTTGCGTTTTTACAACCTCATCATCAGCGTCACAATGTGTATCTGTATAATTATTATTACGGCAAGGTTTTTCTTGATTGGTTTGTTCTGTATTTACATCGACCTGTTGGTTATCTCCGGCCTTGATGTAACCGCTATGTA

>Dvv ABC-C D22413+T49258_c0_seq1

CTAATGACTAATGAATTTTTAATAGAAGGTCAATACCGATAAATCCGGAGGTTCCAAAGTTTCAATGAACAGAGGCAATGGATTCCACAAAGGAAACCTACAATCCCAATCCAAGGGACAGTTCCAATATATTCTCAATATTATTTTTTGGGTTCACGTATCCCATATTCAAGAAGGGCATGAAGAAAGACTTTGACGTGGAAGATTTGTATAACCCATTGAAAAATGACAGAAGTACTCTTTTGGGAGATAAGTTAGAAAGGAATTGGGACAAACAAAAACTTAAACCAAAAGGCAAACCCAGTCTCCTCCGAGCAGTAGCAATTACATATTGGTACGAATATCTACGATTAGGGTTCCTCACATTGATAGGAGATGTTTTTATTCGAGTAAGCCAACCTTACGTTTTGGGCTTGTTGCTCACCTATTACAACCCACATTCCAAAACCACCAAGGAACAAGCTCTAGGGTACGCTGGGATGATAGTTACTTTTAATACTCTAACGTCGTTTATAAAGAATCAGTATATGATGAATTCTATGCACGCTGGTATGCGAGTAAGGACTTCGGTGTGTGCTCTTATTTATAGAAAGGCTATCAAATTAAGCACTACTGCCCTCGGCAAAAGTTCAGTAGGGAAGATTGTTAATCTTCTTTCGAACGATGTAAGCAGATTCGATTCAGCCTCGATGTTACTGCACCAAATGTGGGTTGGACCAGTTTCTGCAATAATAATTATGTATATCATTTTTCAAGATATTGGATGGTCCGGAGCAACTGGAGTTGTTACCATTGTTACAATTATGCCCATGCAAGCGTATATTGGCAAATTATCAGCAAAATATAGAAGAATAATCGCTGGTAAAACAGATGAAAGAATAAGACTGATGAACGAGGTCATAGGGGGCATTCAGGTGATTAAGATGTATGCGTGGGAAATACCGTTTACAAAATTAATCAGCCTTGCACGAAAAGCTGAAATTAATGTTATTAGAAGAAGCGCGTACATAAGAGGTGTATTTATGGCTTTCAATTTATTCAACAATAGATTTGCTTTGTTTGCAACTTTGGCTACGATGGTATTTACTAACAAAGCTATCACGGCAGCAAGAGTGTACGTTTTTATGTCATATTACCAAATCCTAGCTAACACACTAGCTGGAGTATTCGTCAATGGTCTTACACAGATTGCTGAACTTCTAGTTTCCATTAACAGAATCGAAGAGTTTCTCGAAAATGAAGAACACAAAGAATTACCTGCCCTAAAAAGATCTGTAATCTCGAACGTCAATGAAGAGATGGTAGCTTGTAAGAATATGGTTGCGTCTTGGAATGAAAACTCACTTGATCCTGTTTTGAAAAACCTAAATTTTAAACTTGGAAAAAATACTCTACTTGGTGTTATTGGTCCTGTTGGcAGTGGGAAAAGTTCATTGCTACAGTCTATTTTGGGTGAACTTGATATAGTTGACGGAAGCCTAGCCGTCCACGGCACACTCTCCTACGCCTCCCAAGAACCTTGGATCTTTTCCGGCAGTATTAGGCAAAACATTCTTTTTGGTTCCGACTTTGACAAAGCCCGCTATGATGAAGTATTAAAAGTATGCGAACTCAGAAAAGATTTTGACCAATTCCCCGACCGAGATTTCACTCTGATAGGAGAGAAAGGCGCCAATCTCAGTGGTGGTCAAAAAGCTAGGATTAATCTAGCTCGTGCTATTTACAAAGATGCTGATATATATTTACTGGATGATCCACTTTCTGCCGTTGACAGTATTGTTTCCAAAGTATTATATGAAGACTGCATTAATGGATTTTTAGCTAAGAAAGCAAGAATATTAGTAACCCACCAAGTTTATTATTTAAAAACTGCTGATCACATTCTCGTTCTCAATAATGGAGGCATCGAAATTGAAGGCACCTATTCAGAACTACTAAAAAGCGACAACCCTCTCACTGTCCACTTAACCGAAGAAATCGAAGAAATTTTAAAACGTCAAAAGTCCCAAGATTCTGCTATAGAAGAACAGGAAGCTAAGACTATGAGCACGATGGATCTTTCCAAAGTGAATGGAAAAATGAAAGCCATTGTCAAAGAAATGCAAGAAGAAACATCTAAAGGAAAAGTTAAAGGTTCCTTATTCCTCGAATTCTTTAAGTCAGGCATGAGCATCTGCGAAATAATATTTTTAGTTACTCTTCTTACTATAGCTCAAGCCAGCGCCACTGTTATAGATTGGTTCATTAGTTTCTGGACGAATATCGAAGAGTACCAGATGACATTGAACTCTACCGCAGCTAATGTATTAAACTCTACCGTAGTTGATGCATTAAACTCTACCGCAGTTGATGCGATAAACTCTACCGCAGTTGATGTGTTAAATTCTACCACTGTGAATGCATTAAACTCTACTGTAGTTGATGAAAGTGTGCCCTTTTACATGAATTGGAGTAGCCAAACTTGTTTGATAATTTATGGTGCAATTTTAGTCTTTTGCTTGGTTACAACGTTATCCAGGTCACTTAGTTTTTACAGATTTATTCTAAATTGTTCTGAGAATTTACACGGCATGTTGTTCACTGGAGTAACTAATACATATATGAGATTTTTTGATAAAAATCCAAGTGGAAGAATCTTGAATCGGTTTTCAAAGGATATTGGTTCTGTAGATGAAATCTTGCCAAGGATGTTATTCGAAGCTTCTAGGGTCACCCTCAAAATGCTTGGACACTTGGTATTAGTCCTTTACGTAAACCCGGCATCAGTTATCGTTGTAATAATATTGGGAATTTTGTTTTCCTTTATTAGGATAGTCTATCTTCGATCATCCAACAATATTAAGCGGCTCGAGGGAAGAATGAAAAGTCCAGTCTTTAGTCATCTGACAGCTACATTGGAAGGACTCACCACCATAAGAGCCTTTAAAGCTCAAAACATCCTTAGAGCAGAATTCGACAAGCACCAAGATAGCCACACCAGTGCTTGGTTCATGTTTATTAGTACCAGTTCTGCGTTTGGATTTTCATTGGATGTCATCTGTTTGATGTTCATTGGATCGTTAACCTTCAGTTTGATTGGGCTGGGTGAATATTTTAATTTAACCGGTGGTGACGTGGGTCTAGCTATTAATCAAGCTTCGTCGCTTACTCAAAACATACAATTTCTGGTTCGTTTCTCTGCTGATATCTCCAATCAACTAATGTCAGTGGAAAGAATTTTAGAATATAAAGAATTGATACCAGAACAGCAACCGGAAAAACTACTGTTACCTCCAAAAAGTTGGCCGGATAAAGGGATTGTTAATATAGAACATTTAAATATGAAATATATAGATGATGGTCCCACTATTCTAAAAGATGTCAGTGTTAAGATTAATCCAAAAGAAAAGATTGGTATTGTGGGCAGAACTGGAGCAGGAAAATCTTCATTAATATCAGCCATATTCCGATTGACTCCTCTAGAAGGTAAAATCTACATTGACGATATCAATACCAAAGACATAACACTAAAACAGCTAAGATCTAAAGTTTCAATTATACCCCAAGATCCCATCTTGTTCTCCGGCACCTTGAGATATAATCTAGATCCCTTTGATGAATACAGCGATGAAACTTTGTACAGAGCCTTGAACGAAGTCGAATTAAAGGATCCGAGCAACATTATTAATCGTTTGGAAAACAGGGTTATGGACAGAGGATCAAACTATAGTGTTGGGCAGAGGCAGCTAATATGTTTGGCGAGAGCTATTATAAGAAATAATAAGATTTTGGTCTTGGATGAAGCTACTGCAAACGTGGATCCACAGACTGATGCTCTAATCCAAAGGACTATTAGAGAAAAGTTCGCAGATTGTACAGTTTTAACAGTGGCACATCGGTTGAACACAATCATCGACAATGACCGAATATTGGTGCTTGAAGCAGGAGAAATTGTCGAATTTGACCATCCGTACTTATTATTACAGAACAAATTTGGAGTATTCAGAAAGATGGTGGAAGAAACTGGAACAGCTATGCTCAGACAGTTTTTGGAAACTTCCAGTCAGAATTATCAAAAATTAATTGATTAAGTGATCTGTTAAGTTCCTGTATTGTACTTGTATTATTTTTT

>Dvv ABC-C D5345

CAGAGGATCAGTATAATAACTTAAAATATAAAATGATGAAAATAACGTGAAAATAATATTTTTTTTAAATTTCGATAGTTTATATAATGATAAAGAATAATACATAAGTTTAAAAAATACTGCCTTTGTAATATTACCCAATTATACATTATATTTTATGTATATCAACATTTAATAGTCAATAACCCAGAACTTTTTAGCATATTATAAAATAGGCTATGTTCATCTTGTGCCAAAGTACTAACTGCGCCATTTTCAACAATTTCTCCATTTTGTACCACTAGGACTCTATCACAATTTAGAATAGTTTTTAGACTATGGGCCACAACTATAACTGTACTTCGAAAACTATAACAGTCCAAAATTTGTTTCTCCAGAATGTTGTTGAACAAAGACTCAGATCCCTGATCCATCTCTGAAGTTGCTTCATCCAAAAGAATGATTTTGTTTTGATGCAACAAGCATCTGGCAAGACAGATTAATTGCCTTTGTCCAGCACTTAAATTTTTTCCTCCTTCGCATATTTGAAAATCTAGAGAATCTATTATTGTTGTTAACTCCAGCTTATGGATGACCTTCCAAATGTCTTCGTCTGGGTATACACCGAAAACTTTAGGTGCCATATTGCTTTTTATTGTTCCCGAAAAAAGCACAGGATCTTGAGAAATTATGGATATTTTTGAACGAAGTAATGTAAGCGGAAGACTTTGGATATCTTCATTACCAATGGTGATTTTCCCTTCGTAATCAAAGAGCCGAAGTAATAAAGTTATCAATGAAGACTTTCCTGAACCTGTTCTTCCAATTATTCCAACTTTTTCTCCTTCATTAATTGTAATATTAATGTTTTTTAAAATATACTGTTGACTATTATCGTAACGAAAATTTACATTTTTGTATTGGATTATATTGTTTGAAGTCCAATCTTTAAATATGTCCCTAAGGTCATTGTCTTCTGTTTTTATACCAACGTATTCCAAAATCCGTTCTACCGATGTCATTTTATTCTCAATATCTGCCCAAAGCCTAATGGCGTATTGGGCAATGCCATTCAACATAAACGCTTGTGTTATTGCTAAACCAACATCCCCAACACCAGTTTCTTTTCCAACAATGATGAACGCTGCAATAATAGCACCTATAAATGTAGTACAACAAACATCCAAGTAGTACCCAAAAGCCCTCTGGCAGCATTTTGAAGTATAGTTTGCAGATGTATATAAATCTTGATGTCTATCAAATTCTTTGCTAATGGTTTCTTCGGCTTTAAATGCTCTAATAGTTGAAAGTCCTTCGAGAGATGAGTTTAAATGGCCAAGCATTGGACTTCGAACTGCTGTATCCAGTCTCTGCAGATTTCGACCAGTTGGCAAGTAAAGTTTCCTTAAAAAATACAAAAGTATGAAAACTACAGCAGTTGGTATAATGAACCCAACGTTTACTAATGTTATTAGAATTATTACTCCGCATAAACTCAAAACAATTTCTAAAAATTCATATATTGCAAAAGGAATTTGTTCATCTACAATGCTAAGATCCTTCGAAAATCGGTTAATAATGTTTCCAATTAGGTGACTATCAAAAAATTTCATAGTTGCATTGATTACAGTGTTACTTAAGCATCGATGTAGTTTTATAGAAGCCTTTCTTGTAAAATTAAATAACAAAAAGGCTGAGAATATGGAAAGAATCGTGGTTGACAGTGTAAACGCTGAATATATCTTCAAAGAAAAATCCCACACTGTATTTTCTTCATTGATATTCGTTGTGTTGTGATGAATGTGATTTACCCAGTTGCTTAAAATCTTTTCAGTATAACTTTTTGAAAATTGTGAACCAATAAAAAATACCAATACAATTGCAATGACCAAAATTCCTCCACCATAATTTCCATATTTTCTATAGGTATAAAAATCAACTTTTCCAGACTTTTTATTTTCTTGGTACATATTATTTCTTCCAATATTTAATTTCTTCAACAGCGAAGATTCTTCAGTGGTTTCATCATTGTTAACCTCATCAGCGTTGTTATTAACTTCGATCAGATTCTTTAACTGTTGTTCTTTTTTTAAAATGGTTTTGATATCCTGGTCATCCTCTTCGGATAGGTTTTGTGATACAAATGTTTTACCTTCGTTCAATACAATTATTTTGTCATTTCCGTTAGTATATTGAAGGTTTTGAGTAACAAAGATACATATTTTATCTGAGAGGAACTCTCTGATACACTTTTGAAATACGTGGCGTTGAACGTTGACATCCAAAGAGGATAAACAATCATCTATTAAGTAAATATCAGCATTTCGATACAAAGCTCTTGCTAAATTTATTCTAGACTTTTGTCCTTTACTGAGGTTGGCACCTTTGTCACCTATTTTTAGGTTGTTTGCTAGGGTAAAATGCTCAATATCAAAACCCAAAGCACATATATCCAAAATTCGGTTATAACGTTCTTCGTCGAAAGGCTCATCAAAAACAATATTTTGTTTAAAAGTTGCTGGGAAAATCCATGGTTCCTGCGCAGCATAAGAAATAGTTCCATATTTAATTATATGATCACTTTTTGTATCAAATTCACCCAAGATGCTTTTTAAAAGAACCGTTTTGCCACTACCAGTCGAACCACTAATAATGTATAGACCAGAATCAAATCTTAAAGTAATATCTTTTAGTAGCACTTTATCATCTACTTCAATTGATAAATTGTTCAGTGAAACATATGATTTCTTTTGATCAACCTCAATTTCGTCATTAACAATTACTTCTTCAGCAGTCATCATCTGCTGAATTCTTTTCATAGCTGCAGACATGTCAGCTGTAACAGTAACACCGATGGGTATAGCGACATTGAGGGCATGTCTTATTCTTAAAAACAAAGTTGTGATGTAGTATACCAGCTCTGCTGAAATAGTGTTGCCCAAAAGGATGAAAGTGGTTAAAAGTAGATAGTAGGCTATCTTTGATGTTAGACTTCCAATTAGCAGAACGGTGGTTTTGGCAAGAAATATTTTCAATATCGCTTTCATTTCCTTCTTCCTCGTTTGGTTAATTTTGTGTTCAAATTCAAACTCCCATACATTCATTTTTATTGTACTGATTCCAGACAACATTTCTTTGGTAGTTTGCAATCGTTCATCAGTCTTCTTACTAGATTCCAGTCTTAATTTTGATATCAACTTTCCCAATAAAACTTGAGCAGGAATAACTAACAGGAAGAATCCAATTCCAATTAAAGAACCAACGCCCATTTTGTTAAATATTAAATAAAACACAATAATCGCTTGCACTATTCCTACCCACACGTCATTGAAATAGAAAATGAAGTTTTCTATCGCTGCTACATCTTTCGTCATCAAAGTTACAATTTTTCCTGAGCTCACTTCTAACAGCGATTTCTGGGTCAGTCGTAAAGATTTTCTAAATATGGCCGAACAGAATGCAGTTCGTACCTTGATTCCCAATTCAGATAGAACCAGAAGGTAGTTGTGTAGGTAAGTCACACTGAGAAAATTCAGGAGTACAAGTAGGATGGCGCAGTATATGGCTGAGCTTCTGGATAATTCGTCTTGACCAGGTTGAAAGTACTTTATAACTTTGGATAACGTCAAAGGGATGGTTATTATAACTATAGATCTAACAATCACTTGTATCATTCCTAGTAAAAAATATTTTAAACCATAGCATTTACATAATATTCGAAAAAGAGACAAGTATCCATGATTTTTTACGTCATCTTTAATACATTTTTCGACCTCATTTCCGGCTTCTTCTGCTTTGCATTTCTTGAGTACTTCATAAAGTTCATCCTCTTCGAAGGTTCCTCTTTTGTGTGTTTTCCAAAATATTGGCAAGGTGTATAAAAATATGATTCTAGATAAGAAGTTGGAATTCTGTATGGGATGTTCCTGTCTGTTATCACTTAGTTTATTCTTGTTCATGATTATCCCTGACCATCCTCACATCGCCACAAGCTTGCAAATATTTGTCAACTGTACGTTATTACGCAATAACAACTTACAATACGTCTATG

>Dvv ABC-C T47673_c0_seq1

CTCAGAGCAACAGTGGCCTAACCCACAAATTGAACATTTTTAGATTAATATATAAATAAAAGCAGCAACATTATGATTCTGAAATTGTTTAGCCTCCCCGGTCTCCAGATTTGACGCCACTTGATTTTTCTTGTGACCTACGGTAACCTTTCTGGTTACCTACAGGAAAAGGACTATTTTTAAAGGCTGTTTACAGATATTGCTTGAAACCGATCATACCTAACATTTGCTGTAGGTACCTCAAATTAAGTGATATATAATTTCCAACGTCAAATATATCGTCATAACATAAAAATACATTTGTAATCATTATTAATGGTATACCTATACCTAGGTAGATATACCAAGAATTATTTTCGAAATCTCATAACGTGTCAAAATATTAAAACTAATCCCTACTCAGGGAGTGATAGAGTCCTTTGATAACTATCTTTGGCAATTTTCCTAAGTTGATCACCAAGACTCTTGCCTGCTTCAGCTACCATTTTATAAAAGACACCATTAGAATTCTGAAGTAGTAGATGTGGATGATCGAATTCTACCATAGTGCCCGATGCCATCACAAGTACTTTATCCGAATCCATAATAGTGTTCAGTCTATGAGCAACTGTAAGTACCGTACAATCTGCAAACTTTTTTCTTATAGTTTTCTGGATTAAAGCATCAGTCTGTGGATCCACATTGGCAGTCGCTTCATCCAACACTAAAATTTTGTTATTTCTAATAATAGCTCTGGCCAAACAGATCAACTGCCTTTGACCAACGCTGTAATTGGAACCTCTGTCCATCACTTTATTTTCTAATCTATTTATTATATTGGCAGGATCTTTTAGTTCGACATCCTCGATGGCTTTGTATAAAAGTTCATCGGGGTATTCTTCGAAAGGATCCAGATTATATCTTAAGTTTCCCGAAAATAGGACAGGATCTTGGGGAATGATTGATATTTTTGAGCGGAGATCTTCCAAAAGTAAGTCTTTTGTGTCTATTCCGTCTATGTTAATTTCTCCATCGATTTTGGCTAGTCTAAATAGAGCTGCTATCAGAGACGATTTTCCAGCGCCTGTTCTACCGACTATTCCAACCTTTTCATTGGACTGAATAGATAAGTTTAGATTATTCAATGTCAAGGGTCCATTTTCTATATATTTTAATCCCATATTTCTAAATGAAACGTTTCCCTTTTCTGGCCATTTCGGCAATGGCTTTTTAGGTACCACTGGCTGTATTTCGGCAGGTAAGGACTTGTATTCCAATACTCTTTCTACACTCATTAGCTGGTTAGTAATCTCTGCAGACTGACGAATACCCCATTGAACCATACCTGTCAGTGCTGTTGCTTGTGTTATAGCAAGACCGACTTCACCACCTTTTAGTGATAACGCTTCTCCAAAATTAAGTAAATAAAATGTAATAATGGCTATAAATATGAAACATATGATGTCCAAGTAGAATCCAAACGCAGAACTTGCAGCAATATACATGAACCAGGCACTAGTATGGGAATCTTGATGCTTGTCGAATTCGTTCATTAGAATGGGTTGTGCACCAAATGACCTAATCGTTGTTAATCCCTGGAGAGTAGCATTTAAGTGGGTAAATACAGGACTTCTCATTATTCCTTCCAGTCTTTTGATATTTTTCGATGTTTTCAGGTACACATGTCTCATTGATAAGAAGAAAATTCCCATGAAGAAAACTAATATAAGGAAATATGGGTTTATTATTGCAACTAATATCAACGATCCAACCATCATTAAAATAATCTGCGAAGCATCTAAGATAGCTTTGGGCAACCATTCATCGATGGCTCCTATATCTTTCGAGAACCTATTTAAAATTCTTCCACTGGGATTCGTATCGAAAAATCTCATAGTAGCTGCGATGACACTATGGAACATGACGTCATGTAATTTTTGAGAACTCCACATGGCTAGCTTGTAAAATAGCATCGATCTAGCGAATGCTATCACGAATAGAGCTATTAATCCTCCAGCGTATATGTAAATACATGTTTCGGTTGACCATTCTACGGTAGACCTAATACTGACTTCTTCTGTTTTGTTTGCACTTGTTCTAGATAACGTTTGGTTTCTAAATTCTTCTATATTTACCCACATGCTGACAAAATAGTCCACACCACTGGCTGCCACTTGCGAAAGGATATACAACAGAAGAACGAAAGCCACGAAAAATATATTACCTCCGTGGATTAAATAACTCCAGAACAGTGATTCTTTCACTTTACCCTTCGAGGATTCTTCCTGTAAGTCTTTCAGTCTTATTTCTGGTTCTTCTTCATCATCACTTCCAGTAGCTTCTTTTATAATAGCATCGGTGAGACTAAGCTCACTAGCGGCGCTGACTAACGACGTTCGCGACCTCCTGATTGATATTTTTCTGCTAATTTTAGCTGTGTCCGTCTGTTTCTGTTTTTCTTCATCTGTAAGTTCAACTTCGGAAGTCAGCAATTTGGCATATAAATTATCACTAACTGAAAGCTCTTCAAAACTGCCTTCATTTTCTATAGTTCCGTTATTCAAAATAACAATATGATCAGCATCTTTTAAGTGATGAACTTGGTGGGTAACCAATACTCTAGTTTTATGAGCTAAATACCCATTTATACATTCATCATAAAGATGCTTTGAAACATGAATATCCACAGCAGATAAAGGATCATCTAACAAATATATATCAGCCTCCCGGTACAACGCCCTTGCCAAATTGATCCTAGCCTTTTGCCCTCCGCTCAAAGACGCCCCCCGTTCTCCTACGATGGTGAGGTCTCCGTTTTCAAATTGTTTAAAATCCTTTTCTAGGGCACACGCTTTGACCACTTGATTGTATCGGTGTTTGTCGTATTTGCCACCAAAAACTATGTTCTGTCGGATGGTAGCTGCAAATACCCATGCTTCTTGGGAGGCGTAAGAGACGGTGCCGTTAACTTCAATACTGCCATAGATCACCTCGAGCTCTCCTAAAATCGTCTGTAACAGCGAACTTTTTCCGCTTCCGACTGTTCCAATGATACCAATCAACTTCCCCTCATTAACAGCCAAATTTATTTTTTTTAGAGCGTCATCTTTGGTGGCTAAATTCCATTTGACGGTCAAGTCTTTTAAGTTAATAAGAGTTTTGTTGAAGAATATTTTGTCGTTATTGTTTTGGTATTTTACCACTGGACGGAATTCCTCATTTAACATGAATTCCTGGACTCTTTTGATTGCTACTATTAGTTCTGCAATTTCCGCTATACCTCTAACAAACATGGCAGACATCGTTTGGGAAAGTATGTTGAAGTAGGACATGAAGACGAATACCTTGGTAGCTGTGATCTGCTGGTCAGTCAATACCATTGTCAATAATGTAGCGAATAAAGCAGCTCTGGTAGTAAATAAGTTGAAGGTCATAAACAAAGCTCTTACATACGACGACTTTGTTACAATTTTCAGTTCAGCTTTCCTCGCTAATTTGATCAAATGTTTGAAAGGCTTTTCCCATGCGTACATCTTGATTACTTGGACACCAGATATGACTTCATCCATCAGTCGGACTCTCTCGTCCGTTCTTAGAGCAGTTTGTTTTCTATAAATTGCAGAAAGTCGACCAGTGTATGATTGCAAAGGTACGACTAGGAACACCGTCAGAATACCCAGGATTGCTGCATAGCCAGCTTCTCTGTACATGAAGAACAAAATAAATATGGCCGAAGTGGGTGCTACCCACATGTGGTGGATAAAAATTGATACTATGTCAAACCTGCTGACGTCATTTGATAATAAATTAACTATTTTACCAGAAGCAGTGTCTCCTAATGCTGTGTGACTTAATCGAAGTGCCTTTCTATATATCAGGGAACAAACTGCTGCTCTAACTTTCATACCATAATGAAAGGCATTCATGATATACTGATTGATTAATAAAGCGCTTATTCCATTTAGAACTACTACAGCACCTGCGTACCAAAGTGCTTCTTCCTTAGTAGTCTCTGTTCCTGGTTTGAAGTACGACAACATGTTACCCAACATGATGGGCTGTGATAACCTAACTAAGAGATCCATTAGAGCTAATATACAGCCTAGATATAAGTATTCTGGCCAAAATGTGGACACTAGTATTTTCAGCAAGCTCGGTTTTTTTCGGGTTTTGTTGCATTTTAATAAATGTGAATTCCATTTTTTTTCTAATCTATCTCCTAATACTGTACTTCTATCACTTCTTATTGGATTATACAGATCATCCACGTCCAAAACTTTGCGATATCCTTTCCGGAACATCTCGATTGTGTAAGAAAAAAAGAGGACGGAAAATACGTTGGCTCCATCCCTGGGATTGGGATTGTAATTTTCTTTTGCTATATCCATGATTATTCACAGAGTGTTTACTTAAAACCATTGAACTAAACACAAATTTTATTAAGGTTAACTTAGGCACTAAACTAAAACTCGCGTGAACGGAACACAAAACACTTATCAAAAATTATATAATTAACATTAATTAAGAGGGCTCAAAATGTATCACGTTATCAAGACGATTTTAAAAACTTTCAATTACCGACACACTTCTTTCTTCCTAAAGATTAACCATAACTGACACACAGTGACTTAAACGATAATAAAAAGATTGTTTGGGCTGTTTCACTTTATGGCGGCATTAAGAGTCGGATGGTCTCTTTTATGCAGATGATTGTAATTGGAGGCGGCTGTTAAAAGTTATTATCTGGGTTGAATTCCTGGTAGTTAATTTGAACGGCCTGAGGATATTCCCCGTGAGTATTATCTTCCAACTGGACCAATTTGGGTTTTTTGTGCTGCCGTTGTTGCGTATGAGTAATTTGTTATAGCAGCAGAAGTACAATTGTGATTGAAGTCTGCTAATATTCAATTGTACAGCAAACAAAATTTAATCTCCCACGGAAAAACAATTAAAACTATTTCTCACTTGTTTTATTTTCGCGGTAACTGTTTGAAATTTACGTCAACTCACGCCGTGGTTTGTTTGTTCGCTTAAAATAAACGTCCTCTCCGTTA

>Dvv ABC-C T48300_c0_seq1+D21892*

AAATTTTATTTAAAATCTAAATAATTTAGTAATCCTGCTTGCTTAACCATTTTGTAAAACATTCCATTCCTATTCTTAAGTAATTCTTGGGGTTGATCAAATTCTTTCATTTCTCCTCTATCTAATAGCATTACTTTATCACATACTAACACAGAATGTAATCTGTGTGCTATCGTTAACACCGTACAATCAGCAAAATTATCACTAATAATCTTGTGTAACATCCTATCAGTCTCATGGTCCATGTTCGCTGTGGCCTCATCCATTACTACTAACTTATTCTTACGTAAGATAGCTCTAGCTAAACAGAGCAGTTGCTTTTGCCCAGAACTGAAATTCATGACGCTGCTATTAATTGGTTGTTCTAAACTGGCAATAGATGTATTAATTCCAACTCTTTCCAGTGTATGCCAGAGATCTTTATCTTCAAATTCATTCAGAGGGTCTAGATTTGATCTGATAGTTCCTGAAAATAAAATCGGATCTTGTGGTATCACAACAATATTTTTTCTCAAATAGTCCAAAGCCAGTGTCTTAATATCAACTCCATCGATAATTATCTTGCCTTTGACTTCGTACAACCTAAAAAGAGTAGCAAGAATTGACGATTTCCCGGCTCCAGTACGACCAACCACACCAATTTTTTTCTCCAGGTTTAATTTCAAAATTTAAGTTTTTCAACACAAAGCTGTCACTATCACCGTATGCCAAATATACGTTTTGGTAAGAAATACTTCCTGTACTGGGCCAATTTTGAACTGTGGTGCCGTCCTTCTTTTCTGTTTTGATATCTGTATATTCTATAACTCTTTCAAGTGACGTCATGTGGTTTTCCAATTCGGTAAACACTCTAATACCCCAGGTTAGTACTATAGATAATTGCGAAATCTGCGTTAAAGATAAACCAATATCACCAGCAGTAATATCTCTTTTAAAGAAAAGATAATCCATTATGATGAAAATCAAGAAGAAATTACTGAGCATATCGACGAAAAATCCAAAAGCTCTCGAGCTCATGTACCAAGTAAAATTGGCAGACGTGTATAAATCTAGATGTCTGTCATATTCCTCGATGAGGATAGATTGAACATTGGATGCTCTCACGGTAGTTAAGCCTTCCAGTGTGGCGTTCAGATGGCCTAACATGGGACTGCGAGATGATGCCTCCAATCTCTTCATACTTCGTCCTGCTGGCAAATAACAACTCCTTACCCAAAACAACATGATAGTCACAACAATGACACATACTGCAAATGCAGGGTTGATGAAAATCGTCAGGGTTAATATTCCACCCATACTGAACAGCATCCTGCAAAACTGATCCAGGTTATATGCAATACTTTCATCAATATTCATCATATCTTGGGAAAACCTAGTCAAGATATTTCCGATCAAGTGGGTATCGAAAAATCGCATAACCGAATTGTTAATCTGATACACCAAGGCCTTGTGGATATTGATCGAAGCCGTTTTGCAGAAACTTAAAAGTAGCCATGTCGTAAGTAATTCCAATGATGAAGAGGCTAGTAAACATAAATAATAGAACCTAAAAGTGGTATGTTCCTCGGCTTGAGCAGTCTCCAGTGTTATATTCATGTAGCTGAATTGTTCTGTTGAGATGTTTGCAATACTATTCTGTATAGTCATGACTGCTTGTTTTTTGTCGACCCATTTTGTTAAAAGTCTATCCGCTGAAGTAGTAGTAAATTGAGTGAACCCTCTCATAACTATGTTCAAAAGAATCAAGAATACTCCTCCTCCATAAATCATGAACTTTTTATACACGTACCAATCAACAGAACCTTCCTTCTTCTGTTCTTTATAAATTTTAGTTCTTGCTGTGCTTTGTTCTGCTTCTATAAGTCCCGTTTTTTCACTCGATTCTTCTATAGTTGGCTGGTTGGAATCATGAATGACTTCTTTTTCTATACTATCATCCTGATCTGCTATCATATAACTTTGTTGAATAATTCTGGCAGTAGGTTTACCCATGTCTTTAATTCTGCCGTTTTCCATTATGATCACGTTATCAGCTTCTTTTATCTGAGAAGCTGTTTGGCACACTAAAACACATATTTTTCCTTTTAAAAATTTCTGGATGCATTCGTGAAAAATGTGATCTTGAACTGAAGCATCCAGTGCGGTCAAACTGTCATCTAAAAGGTATATATCGCTGTTTCTGTATACTGCTCTTGCCAGGTTTATTCTGGCTTGTTGACCCTTGCTCAGGTTTTGACCATTGTCAGTTAATATAGTCTCGTCTCCGTTTTCAAAGAGGTTAAAATCAAATAGAAGAGCACAGGCCTTGACTACTTCTTGATACCGTTGTGCATTGTATGGTTCTCCAAATAAAATATTCTGCCTAATCGATGAAGGAAACAGCCACGGGTCTTGGGAAGCATAGGAAATCCTTCCCTGTGTTTCAACGAAACCTTTCGTCAACGGCAAATCCTTCAGAATAACTTTTAAAATGGAGCTCTTACCAGATCCCACAGCACCAGTAATCATAGTCAAACCAGAAGTTATCGTAAAGGAAACATTGTCCAAAACTGGTTCTCCTTTGAGTTGCACTGAGATTTCATCGAGCTCTACACGCGGTTTTTCTATGAGTTGCTCACTGTGTTGATCTTTATTTAATTCTTCAGCATTTAGTGCTTTAGATATTCTCTTAAATGATGCTACGAACTCGCTGCCTAAGCCTAAACCAAATGGAACTGCTCCTCCCAACCATCCTCGAAGGTATCTGAAGTTAGACAGGACGAAGAAAATCACTGTTGTGTCGGTTGATACACCTGTCCAAATTGTGGCCAAGATAACCATGAAGAAACCGAGGTTTGTAAATATTATTCCGTTAAGGATTAGAACTCTTTTGAGGTAGAATGCTAATGTAAGCTTTTTCATTTCGACTTTCCTGGCCTGTGAAACTTTTCTGGAAAAGAAGTTTTCCCAAGTATACATTTTGATTATTTTTATCGTCGTAAGAATTTCTTGAGTAAGCTGCAACCTTTCATCAGTTTTTTTACATCCTGCCAACCTACATTTTGCAATGAAAGTAGTTATAAGTATTTGTAAAGGGATAGATGACAACAAAACTCCAATCCCCACGAACGAGGCAACTCCAATTTTAGAGTAAAGCATGTAACATATAATAACTGTCTGTGTTATAGCAATCCACAAATCATTGATGGTAAAAATTACTGCGACAAACGACTGTACGTCTCTTGTTATCAGTGTTACGATGTTTCCCAAAGAGGTTTCTGCTACAGCGACAGGTGATAACCTCAGAGCTTTCCTGTATAGCAAAGATGCAAATGCAGTCCTCATTTCCATTCCAAGCGTTTGGACCCACAACAAATACTGATGATTATAAACAGTGTCCAAACAGATATATATCAGGTAGGAATATCCATAAAAGTATCCATCTTCTCGAGTATATGTCGTTTGTCCTGGCGAGAAGTATCCAACCAAATGACTTATGAAGTAAGGTGTCATGATACTTCTTGCTGTAGAAACTACAAAATCAATAAATCCAAGCAGAAGATATTTCAAACCATAACAATTCCATAGTACTCTAACCAACGAAGGATGTTTCTCCAATTTTTTCTCATTCATCCATTCTTGTTCCAAATAATCACCACAAGTTTTTGACTTGCAGTTTGTGATGACTTCAAAAATGTCTTCCTCAGTGAGGTCAGCTTTACCTGCTTTTCGAAACAATCCTCCAACATATGCAAAAGTTAAAAAAGAGAAGATGTTAGCTGTCTCTCTAGGATTAGGTTTTCTCCTCTCTCTTTGACAATGATCCATTGTTCACAGATACTTAACATTGACTGACTAGACT

>Dvv ABC-C T10132_c0_seq1+D20603+T57212_c0_seq1*

GTGGGTTTGTTAAGATAAAAATCATTTTAATATTTCTTCCTGATGTTATTAGGTAAGATAAACTTATGTACATGCTTTAATAATGTGGCTTAAACAGAATACATTGCTGTCTTCTTGTACTATGTTATGTTCTTTCAATTAGGACTGGAATTATTACTGGAAATACACAGGTTACATAATTTAAAAGAAATTATAATTTCACATAAATATAAATTACATTTTTTTACAAACACCAATTAAAAACATGAAGGAAATATTCAGCTACTTGCATTCGCGGCTTGACTTAGCATATAAGCTTTATTGGCAATTTCTATTAGGTTCTGAGCTGTATTTGTGCCGGTCTGCATCAGTAAGGAATAGAAAACACCATTTGTGTTTTGTAAAAGAATATAAGGACTGTCATATTCTAGGATTTCTCCAGCGTCCATAACCAATACTTTATCTGAATCCATAATCGTATGCAACCTATGTGCCACAGTTATCACTGTGCAGTCTGCAAATTTCTTCCTTATAGTTTTTTGAATGAGTCCATCTGTATGAAGATCAACATTAGCTGTAGCTTCATCTAAAATTAATATTTTGTTTTGGCGAACTATGGCTCTAGCTAAACAAAATAACTGACGTTGTCCTACACTAAAATTTATTCCACCTTCTGCCATTTTATTATCTAGGCCTTCCGGAAGGTCACTTACAACATATTTAAGCTCTGTATCTTCCAGAGCCTTCCATAATATCTCATCAGGATATTCGTTAAATGGATCCAAGTTATACCTAACTGTACCAGAAAACAGTACTGGTTCTTGAGGTATAATGGATATCTTTGACCTCATTTTACGTAATGGGATCAATTTTGAATCAATACCATCAATAATAATGCTACCTTCCGTAAAAGTCAACCGAAACATTGCTTGAATAATAGAGGATTTTCCAGCTCCAGTTCTTCCGACGATGCCAATCTTTTCCTTCGGGTTAACAATAAAACTAAGATTCTTTAGAACCAAAGGTAACGTCTTGGAGTACCCGAGAGATAATTTGTTAAAAACAATTTGCCCTTGTTCTGGCCAAGTTTTAGGCGGATCTACACCTTCGTCAATTTCTTTCGTTAGATCTGCATATTCTTGGACCCTCTCTACTGAAGTCATTTGGTTTTCTAATTCACTCCACTGCCTTATACCATACTGGAACATTCCCGTGAGACTCATGGATTGAGTTAAAGCCAACCCAACATTTCCACCAAATTTTTCTTCTTTTACAACCAAAAGAGCAACTAAAACTAGCCCAATATAAACCACACAGAAAAAGTCTAACCAGAAGGCAAAAGTTCTAGTGGCCCCTAGATACATAAAAAATGCTGCGCTATGAATATTTTGCAGATTGTCAAATTCCACAGCCAATATATCTTGTGCTTTAAATGCTCTTATCGTAGTTAGACCTTGAAGAGATGCCGTCAGATGGGAATATACAGGACTACGGTTGATCGATTCCACTCTCTTGACATCTCGGCTGGTCTCCAAGAATATAAACCTCATAAAATAAAATATTACTAATACTATTATAGTCGGTATTAGCATCCAAGGACTTAAAGTTCCAATTATGAGACATATGGATAATACTGATAAACCGATCTGAAGTGTGTCCAAAATAGTAGTTGGTAACACCTCATCTACAATTCCAATATCTTTGGAAAATCTATTCAAAATTCTCCCAGACGGATTATTATTAAAGAAATGCATAGGACTGTATATGATGTTGTCAAACATTTTATTATGCATAACTATAGAAGAAGACAGACCCCATCTGTAGAAAAATATTGAACGTGTGTAAGTGACTATAAGCAATAATAAAACAAAAGTCGCATACACATATACAGTAATATTTCCGGTAAAGAAAGAGGTCAACCAGTGATCCTGTGTCAAGTCGTATGGATTAGCAATTACCGTGGAAGATTGGTTGGATTTGTCCAAAACTGTTGTTGAATTATATGTTTGTATCACCCACTGCTCAATATTAACCCATATTGTTACAAAATATTCTGAAGAAGTGTCGAGAACCTGACTCAGAATAAAAGCTAAGAGTAGTATTACAACTTTACATAAGTTTCCACCAGCTCTAATATAATTCATATAAACGTAAGTAGTAACTTTCCCTACACCCTTCTCTTCTTTTATTAAAGCAGGAGCTTCTTTTTCTCCCTGGTTTTCGATATATAGTTTATTTTTAACGTGTTTCTGATTCTTTTCATCTTCTTCCTCTTTTTCTAGATTTTCTAGGAGTTTGGTAAATATACTATTCGATTTTATAATATCTTCGTATGAACCTGATGCCCTTACCATGCCATTCTCCAGCAAAATAATATTTGCTACAGTTTTTAAATATTGCAATTGATGTGTGACTAGAACCACACATTTGTCAGCTAAAAATCTACAAATGCATTCTTCAAACAAATGTTTTCCAACATGAGTATCTACCGCGGACAGCGGATCATCAAGAAGATATATATCTGCTTCTTTATATATTGCTCTAGCTAAATTTATTCTAGATCTTTGCCCTCCACTAAGAGTAATTCCTCTCTCACCTATTCGCGTTTTATCACCATATGGTAAAAGAGTTAAATCTCTTTGTAAAGCACATACCATGAGTACTTCTTCGTACCTCTGTTTGTCGTAGGGTTGTCCAAAGAGAATATTTTGCCTAATGGTGCCACCAAACAACCACGGTTCTTGTGATGCAAATGATATTTTTCCATTCACTTCAATGTAACCCTCTGAAGGTTCTAATTCTTGAAGAATTACTTGAAATAGTGATGATTTTCCACTACCTACTGCTCCTACAACGGCCACCAAACTTTTCGAAGATACGTCCATTGAAAT

>Dvv ABC-C C217405_3.0+D12550+S19317Locus_19671_1*

TCATCTTTATACTCATCGAAAGGATCCAAATTATTTCTCAATGTTCCAGAGAATAGGACTGGCTCTTGAGGAATAATGGAAATTTTAGATCTGAGCTTCTTCAAAGCCACAGATTTTGTATCAATATCATCAATAAGTATGCTACCATCGATATGAGTCAACCGAAAAACAGCTTGAATTAACGAAGACTTTCCTGCACCTGTTCTTCCCACTATACCCACTTTCTCAGAAGGTTGGATAACAAAACTGAGATTCTTAAGAACTGGGGGATCGTCTGGTGAATACTGAAGGGACATATTTTTAAATTCTACTTTTCCTTGATCTGGCCACGTCTTATCTGGTTCTACAGTGTGGTTGTCTTCTTCGTGTTTTAAGTCAGCATATTCTTGTACCCTTTCCACTGATGTCATTTGGTTTTCGAGTTCAGACCATTGTCGCATGCCCCATTGGAACATTCTCGTCAGACCCATGATTTGAGTAAGAGCAAGACCCATGTTTCCTCCAAATGTTTCGCTTTCAACAAAAATCAGCGACAATATAACTAAGGCAATGTATATTACACATATAAAGTCCAACCAGAATCCAAAGGTTCTATTTGCTCCTAGATACATGAAAAATGCAGCGCTGTGAGTATTTTGGTATTTATCAAATTCAGTTTTGAGTATATCTTCAGCTTTGAATGCTCTGATAGTAGTCAGTCCTTGCAGCGATGCTGTTAAATGAGTGTAGATGGGACTTCGCGTGACGGATTCCACCCTCTTAACGTCTCGACTGGTTTCTAAAAATATGACCCTCATTACATAAAACAATATACCTATGCAGATAGTGGGTATCATGATCCAGTATGTCAAACTCCCTATCACGAAACAGATTGCAGCTACCTCGAGACCAATTTGAACAGTATCCATGAAAGTCATTGGTAAAACTTCATCTAAGGCACCGATATCTTTGGAAAACCTATTAAGTATTCTCCCAGAAGGATTTATAGTGAAGAACCTCATGGGACTATAAACTATGTTCTCGAACATGGTATTGTGCATTTTTGTTGAGGCAGTTATAGACCATGCGTAGAATGAAGACGATCTAATGTTCATAATAACGACCATAAGAATGTTTAGAGCGCTAAAGTAGATTACAGTGTTTTCGCTTATAAACAATGGGGTTAGCCACCAGTCTGCGAATCCTGTATGATCAACTGGTTGAGGAGAAGGACTGGTAATGTCTAACGGTGCAGTCATATTTTCTAAAGACGCAGGTGCTGTTGGGGATGCAATATTTTGCAAAGCTTTCCATTGTTGAATATTGACCCAGAAAGTAACGAAATATTGGCACATACTCAACAAAGCTTGGCCCATTACAAACAGAATTATTAAACAGAGAGCCTTAAACAGATGCCCACCTGCTTGAGCGTATTTTAGATACACACGTCCAGAAATATTACCAGTACTCTTAGCTTCTCTTTGTAACACCTGCACTTCTTCTTCTTCTTCATCTTTCTCTTGTTTTGCTTGAACAATTCTGGATTTCCTTCTTATTTCTTCCTCTTCCTCTTCTATATTTGCCAAAACTTTACTGTAGTCTGTATTGGAGTTTCTTATGTCATCATATGATCCAGAAACAGCTACTTGACCATTCTTTAATAAATATATATTCGGTATTTTCTTTAAATATTGTAGCTGATGCGTCACCAAGACGACGCATTTGCTTCCAAGGTATCCACAGATACATTCTTCAAATATCTGTTTACCAACATGAGTATCAACGGCGGATAGGGGATCATCTAGTAAGTAAATATCAGCTTCTTTGTAAACAGCTCTAGCTAAGTTGATTCTAGCTCTTTGTCCTCCACTTAAAGTAACTCCTCGTTCACCCGCCAAGGTTCTATCTCCATGAGGAAATAGAGTAAAATCTCTTTGTAAGGCACACACTCGTACTACTTCGTTGTATTTAACTTGGTCGTATTTTTCTCCAAATAGAATATTTTGCCTTACGCTACCTCCAAACAGCCAAGGTTCTTGAGATGCATAAGAAATTTTACCACTTACAGTAACAGATCCACGTTGGAGCTCGAGCTCCTTCATTATTATGTGCAGTAAGGTTGTCTTTCCACTTCCTACAGATCCAACCACAGCTGCTAGATTACCAGGTGTTACATCCATGTCAATGTCTTCTAAATTGTTCTCGGGTTGCGACTTCAACCATTTAGCAGACGCGTTTCTAATATGGATGCTTCCCTTATCGGAACTAGGTAATTCTTTCATAGCAAGTTTGCCGTTTTTGTTACCGTTTAGAGAGTTTTCAGGAATGAAATKAGCGTCAGTCTTGAGCTCATCATACTGCAAGAATTTCTGGATTCTCTTCATCGATATGTACATCTCGGAGCCTTGAGAAACGGCCTGAGGGAAGAAGTAAGTCAATGTGCCCATCAGACGGTAATATGAAGTAACTGTGAAGGCATAGGAAGCTGTTAGAGGATTTCCTGTCAAAACAAATGTTACCAAGCATATGGCAACAGCTACGTCATTTAAAGTCATGGCCAAGGACATCAGAATTGCACGAATGACGGATGTTTTCCTGATGAACTTTACTTCATTCCCTCTAACTAACTCAACTAACTTGGCAAATGGGTATTCCCAAGTGTACATTTTGATTACTTGAATTCCAGATATGATTTCATTCATTAGCCTTACCCTTTCATCGGTACGAGTTGCAGTTTTCAATCTGAACTGCGAAGTTTTCTTGCCCAACCATGATTGTAAGGGTATAGAGAGTAACAAAAATATAGTTCCAAGAAGTGCTGTCCATCCAGCAAAGAGATATAAAAAAACCATCACAATAAGTGCCTGTATTGGAGCGATATAGAAGTAATGAAGATGATGTGCAGCTTGATCAAATCTTCCTACATCATTAGAAAGTAAATTTACCATCTGCCCAATGGTAGTTTCTGCTAGAGCAGATTTGCTAAGTCTTAAAGCTTTCCTATATATCAAAGAACACGAAGCTATTCTCATCTTCATACCAACTTGCATCATTGCAAAATTGAATTTATGGAGCAGGATTACGCTGACCAATGAAGTGGCTATTATCAATCCTGAGTACAAATAAACATCATTGATATTTTCTTTGGGATCCTTTTCATAAATCGTTAGTAGTTTTGATATTATAAATGGTTGGGTCATTCTTATCGCCTCTATTAATACAACAAGAACATTGAGAACAAATATTTCCCAAATAAATGTTCCTATTAACACCTTATAATATGCGGGATTCTTGTTCTTAGCTATGTGTTTGATCCACCTTTCCTCTAGCCTATCTCCAAGCTTACTGGAATCATGTTCACTTCGGTGTCTGTACATATCATCTTCGGTTAGTTCTCGTTTGTAGCCTTTAACGAAGTAAGGAAGTAACCAACAAAATAATAAATTGGAGAAATAACTTGATCGATCTATTGGATTAAGTTTTTGTTCTGCAGTCTGGCCTTTTGATTCCTCCATTTTGTAATTTTAGCTCTAAATTCAGTGCCTACAAGCCAGGAAATATCAGGAACATCAGGACATAAAGTAGTTTTAAGTAAAGCACTTGAACAAAACAAAACGATTTTTTATCACATAAAATAATTGATACCTAATTGAAGTGAGAAGTGTATGTTTGTGATATGA

>Dvv ABC-C T48940_c0_seq1+D21306

ATTTATTAGGAACTGGCTTTATCACAAATTTTTGCAAATAAGGATTTTTCATTTTCTAATAGCGTCTTCGGCTCATCGAACTCAACAATTTGTCCATTTTCCATCACAATTACCATATCACAGCTCAGAATAAAGTTTAACCTATGAGCTATCGTTAAGATAGTACAATTCTTGAATATTTTCTCGACCAACGAATGTATCAAGTTATCATTTTCCGTGTCCATGTTCGCGGTAATCTCGTCCAAGACTATAATCCTGCAGTTATTCAAAGCCGCTCTGGCCATGCAGATTAGCTGTTTTTGTCCTGCACTAAAGTTGGCAACAGTAGAGTCCAAAGTTTGTATGTTTTCAATCGAGAGTTGGTTAAGCAGAGACCAAATCTTATCGTCTGCGTGTTCTTTATAGGGATCTATGTTTTCTCGAAGTGTGCCACTGAATAATATAGGGTCTTGCGGTATTATGGCGATGTTTTTCCTCAAGCAACTCAAGGATATAGTTTTGATATCCACGCCATCAATTTCAATATTTCCTCCGTATTCATATAGCCTAAACAACGTTGAAATAATTGAAGATTTTCCTGCCCCAGTTCTTCCTACAATACCAATGTTGTGTTTGGGTTTTATTGTAAAATTAATATTTTTCAAGACTGGAATCTTGTTATCACCGTATTTTAATTCAACTTGCTTGTAAATGACTCCACCTTCTGTTGGCCAGTCCTTTGGTGTTTTGCCTGAGTCATCTTCTTGTTTCACTTTTGTGTACTCCAAACAACGTTCCACTGACGTCATATTAGTTTCTATTTCTATCCACTCGTTGATACCCCATAAAATCATATCTGAGAGTCTTATTATATTTGTAAGCGCAAGCCCTACGTTTCCAGCGGAAGTATCGTCTGGGAAAAACAGGAATTTCGCCATAATGATGGTGGAAAACGTAGCACTGAGTGCTCCCATATAAAATATCAAAGCAGTATGGCTCAATCTCATAGTGAAAACTGCCGACGAATACAAATCCTGATGTCGATAGAATTCATTTGTCAGCCTTTTTTCAGCATCGAATGCTCGGATAGTTGACAAACCATCGAGCGTTGCATTAATGTGGCCCACCAAAGGGCTTCTCGTTGCAGCTTCTAGTCTTTTTAAATTACGACCCGTGTTGATATATAGAATTCTTAACGTCACAACGAGGCTGATAAAAATTATTGAAGGGAAGAGGAATACCCAATTAACTGTTGCTATGAGTACCAGGATACCTAAGCAGTAAAATGCGGCCCTAAATAGATGAAGAAACATCATAGGTAACCTCTCGTCTAAAATATTCAAGTCATACGAAAACCTATTGACCACATTGCCTAAATAATTTGTATCAAAAAATGTCATTACTGCTGCTGTGATTCGAGAAACCATCAGTTTATGTACATTGTAGGAGGCATTCCTGCAAAATCTTAACAACGAGTATAGAGTCACTAAATTGTACAGAGAAGAAAATACAGTGGTAAACGTGGTGAATAACAGAAGATCTTGCTTTTTGTAGGGTCCTCCAAGAATGGGGAGCATTGTGCTGCCATCGTTAGCTTGTACGTCAACCCACTTTGCCTTTAATTTCTCTGAAGTAAAATGAGCTACTTGGGAGCCAGTATACAGTAAAATTATGAACACGACAAAGCAAATTCCTCCTCCGAATCTCATGTAAGACTTATATACATCCGCCCCAACCTTTCCCGTGTTATTTTTCTCGGCGAATAAATCGTCATTTGTATATTGATCGTCTTCGTGACTAGATTTAGCGGCCTCTGATTGTTTCTTAGCTTCAGACACCTGAACGGGTTCTTTTTGTTCCTCACCGATGTCTGGGGTGGGAGCCGGAATTATGTTAGTGTCACCTACCAGCTTTCCATGACTTAGAGTAAAAATGTTCTCAGCGTTTTCGATATGTTTTGGATTCTGAGAAATCAAAATACAAATCTTCTCTTTTAAGAATTTAAGAATACATTGTTCAAATATCACATCTTGGACGTTGGCATCTAGAGCTGTAAGCGGATCATCCAGCAAATAAATGTCACTCTCTTTGTATATACACCTGGCCAAATTAACTCTAGCTTGTTGTCCTTTACTAAGATTAAGTCCAGCTTCAGCTACAATAGTCTCGTCTCCGTTTTGCATAAATGCCAGATCGTAATCTAAACCACAAATTTTGAGAACTTCTTCGTAGCGTGCTTTGTCATATTTTTCACCGAAAAGAATGTTGTTTTTAATCGATGATGGAAATAGCCATGGTTCTTGAGCTGCAAACGATATGGTACCGCCTAATTTGCAGTATCCTTCTGACAAATTGTGGAGTCCAATGATAACTTTAAATATCGAACTCTTTCCTGACCCAACAACTCCTGTGATCACGTGCAGTCCAGGTTTCGAGATTTTTAGAGAAACATCATCAAGAACCTTCTGATTTTTTATTTCTACCGAAACATTGTTAATATCAATCAAAGCATCTTTGTAAATCTTTTCTATTTTCGAAATTTCTTGGCATTGGAAGACTTTGTTGATTCGTTTCAAAGATGCAATCAACTCTGCTGTTCTACTCATGTTTATGGGGATCATTATCCCCATGGCCAATGTGAGTTCTCCGAAGAGGGAATCTATGTAGAAGATCAACTCGGTGTTGGTGTCGTAACCTAGCGCAGTGTAAGTAAGGATCAAAGCCAGGAAAACTATTCCTGACAAAAAAATTCCGATGATGACGATAAGGAAGTGAACAAAAAACATCTTTAGAAGAGCTGACAATTCTTTGCTTCTTTCTTTAGATATTTTTCCAATAAAAAACCTTTCCCAAGTATACATTTTGATGACACGAATTGCTGTAAGTGCTTCCTGCGTTATCTGTAGCCTTTCATCAGAGTGCTTTCCTACTGTTAGCCGCAGCTTTGTTACTAATTTACAGAGAAATCCTTGTACGGGAAGAGCTACGGCAAACATTCCAACGGCCACGAATGCAGTTACCCCAATCCTGCTATATAATAAATAGAACACGGTCGTCGTCTGTACGAAAAATATTACAAATTCTTTAAACACCCAAAGATTCGTCTCAATACTGTAGATATCTTTTGTCATGACTGTTATTAGATTTCCAGAGCTGGTCTCTAAGATCGAGTTGGAGGAAAGCTTCAGCAACTTTTCATACAGGAGGGACTGCAGCGATGTCTTGAGTCTTAATAAATATTTCAGTTCGAATACGTTGATGTTAGCGGTGTAAAATACCGAGAAGAATTTGATAAATATTACTAAACTCGCGCAGTAGAATGCTTGTGTTTCTGATATTGTCTGCTGTTTTTTGTCGAAATATGCAATTAATTTGCTCATTCCATACGGTCGTACTATACTGCGGATTTCGACCCATGTTACATGAGCTAAACAAAATAACAGGTAGGCTAATCCGAATTTGCTCAACAATAGTTTAAATAACGACGGATTTTTATTCCACTGCTTCACTGCTCGCTCTCCGTTAAATTTTGATTTACATTTTTTTGGTAATCCATATAAATCACTCTCGTTCAAATCATGTTTCCAACCTTTTTTTATTAGCTCCAAAACATATCCAAATGTCAAATATGAAAATATATTAACTTTTGATAAAGGATTGAATTTCTCCTTTGGTTTTTCCATAATCTTATTTACCATGACTGGTAGCCATTAAATCTTTCACTTAAAGGATAACGCTTACAACACAAACCCAAAAAAATTTATACCATTCAGTTTGGAATCTATGTTATTCACAAACATTAGATACAAGTGAATCATTATTAGTTGTTTT

>Dvv ABC-C T43960_c1_seq1+D21270+T49513_c0_seq3*

AAAACTGAGATTCTTAAGAACTGGAGGATCATTTGGTGAATACTGAAGAGACAAATTTTTAAATTCTATTTTGCCTTTTTCTGGCCATGTTTCATCTATTTCTACAGTATAATTATCATCTTCGTGTTTTAAGTCAGCGTATTCTTGGACCCTTTCCACTGAGGTCATTTGGTTTTCTAGTTCAGACCATTGTCGCATCCCCCACTGAAACATTCCCGTCAGAGCTAAGGTTTGCGTGAGGGCAAGACCCATATTTCCTCCAAATGTTTCACTTTCAATGAAAATCAATGACAATAGTACTAAGGCAGTGTATATTACACATATAAAGTCCAGCCAGAAACCAAAGGTCCTAGTGGATGCAAGATACATGAAAAATGCTGCGCTATGATTATTTTGATAACTATCAAACTCATTTTTGAGTATTCCTTCAGCTTTGAATGCTCTTATAGTAGTCAATCCTTGAAGTGTAGCTGTTAGATGAGTATAGATAGGACTTCGGTTTACTGATTCCACTCTCTTAACATCTCGACTAGTTTCTAAAAATACGACCTTCATTACATAGAACAGTATCCCTATAAGGATAGTAGGTATCATTATCCAGTAGGTTAAACTACCTAGTACGAAACATATCGCAACAACCTCAAGGCCAATTCCAAGAGTATCCATGATAGTCATTGGTAAAACTTCATCTAAAGCACCGATGTCTTTGGAAAACCTATTCAGAATTCTTCCAGAAGGATTTATATTGAAAAACCTCATTGGGCTGTAAACTATGTTCTCAAACATCATATTGTGCATTTTTGTTGATGCTGTTATAGCCCACGCATAAAACGAAGACGACCTGATATTCATAATAATTACCATCACCATGTTTAAACCAATGAAGTAGAATGAAGTGTTTTCACTCACAAAAACTGGGGTTAGCCACCAATCAGCAAAACCTGTATTGTCGGTCGATTGAGGAGGAGTGGTCAAATTTAACGGTGCGGTAACATTTTCTACTACAACCCCAGTGTCTGTTGAGTTTAGTATGTTTTGTGAAGATTTCCATTGTTGAATATTGACCCAGAAAGTAACAAAATATTGACATACGCTTAACAAAGCTTGGCTAAGTATAAAGAGGAATAATATAGAAAAAGCCTTAAAGATATGTCCACCAGCTTGAGCATACTTTCGATAAACACGTCCAGAAATAATTCCTGTACTTTTACCTTCTCTTTGTAATACAGGACCTGCTTCTCCTTCAGCTTCTTCTTCTTTTGTTTGAACAACCTTAGACTTCCTTCTTATCTCTTCTTCCTCCTCTTCTATATCATGCAACAGTTCACTATAATGTGTATTAGAATCTTTTATATTATTATAGGACCCGGAGACAGCTATTTGTCCATTTTTTAATAAATATATATTCTGAATACTTTTTAAATACTGTAGTTGATGAGTGACCAGTACGACGCACTTGCTTCCAAGGTATTCACAAATACATTCTTCAAATATATGTTTACCAACGTGTGTATCCACAGCGGATAAAGGATCATCTAGTAAGTAGATATCAGCGTCTTTGTAAACTGCCCTAGCTAAGTTTATTCTAGCTCTTTGTCCTCCACTTAAGGTAACACCTCGTTCACCCGCTAAGGTTCGATCACCATAAGGGAATAGAGTAAAGTCCCTTTCTAGTGCACATACACGAACTACTTCGTTGTATTTGAGTTGGTCATATGTTTCTCCAAATAGGATATTTTGCCTAATACTACCTCCAAATAACCAAGGCTCTTGAGAAGCATACGAAATTTTTCCAGTGACATTAACAGATCCACTTTCTAATTCGAGCTCTTTCATAATTATGTGCAACAAAGTTGTCTTTCCGCTTCCTACGGATCCAACAACAGCAGCTACACTACCAGCTGCCACATCCATGTCAATGTTCTCTAAATTGTTCTCGTATTGTGACTTCAACCATTTAGCAGACGCCTTTTTAATATGAATGCTTCCCTTATCAGATCCAGTTAACTCTTGTAAGTCTTGTTTACCGTTTTGTTTACCATTCAGTGAATTTTTAAGATTTTCAATGTTAAGTTCATCATATTGCAAAAATTTCTCGATTCTCTTCATGGATATCCACATTTCGGAGCCTTGAGAAACGGCCATGGGTAAGAAGTAAGTAAGGCTGCCCATGAGACGATAATATGAAGTAACTGTAAACGCATATGAAGCTGTTAGGGGATTTCCTGTCAATACATATGTCACCAGGCATATGGCAACAGCTATATCATTTAAAGTCATGGCTATGGAAATTAGAATTCCACGGATTACGGATGTTTTCCTAATAAATTTTATTTCATTCCCCCTCACATACTCCACTAACTTTGCAAATGGGTATTCCCAAGTATACATTTTGATTACTTGAATTCCAGATATGATTTCATTCATAAGTCTAACCCTTTCATCGGTTCGAGTAGCAGTTTTCAGCCTAAACTGCGAAGTCTTCTTGCCCAACCATGACTGTAGCGGTATGGACAGTAGCAAAAAGAATGTTCCAAGAAGTCCTGTCCATCCCACAACTATATATAAAAATACCATCACAATAAGCGCTTGTATAGGAGCGATATATAAGTGATGTAGGTGAATGGGAGCTGTGTCAAATCTGCCTACATCATTAGAAAGTAAATTTACCATCTGTCCAATGGTAGTTTCGGCTAAAGCAGATTTGCTGAGTCTTAGAGCTTTCCTATATATTAGAGAACACGATGCTATTCTCATCTTCATACCAATTTGAAACAGTCCTAAACAAAATCTATGTACAAGGACCACACTGATAAATGAAGTCGCAACTATTAAGCCTGAGTACAGGTAAACTTCGTTAATATTTTCTTTGGGATCTTTTTCATAAACGATTAGTAACTTTGATATTATAAACGGTTGAGCCATTCTTACTGCTTCACATAATATAACAAGAATGTCCTGACCGAATATTTCCCAAAAAAACGTTCCCATTAACACTTTCCAAAATGCAGGATTCTTATTTTTAGCTACGTGTTTGATCCACCTTTCCTCCAGCCTTTGTCCTAGCTTGCTAGAATCGTGCTCACTTCGATGTCTATACATATCATCTTCGGTTAGTTCTCGTTTGTAACCTTTGACAAAGTAAGGAAGTAAGTAACAGAAAAATAAACGAGAAATAAAATTTGACCTATCTTTTGGATTCAGCTTTTTCTCTGGAACCCCACCAACTTTAACTTCCTCCATTTTATAATTTTAGCTGCAAGTCAGACAAATCAGCAAACAGTGTTTTTTTAATTGAAGCACCTAAACTAAACTCGAGTTAAATCACTTATTATAATATACAAATAATTGACGTCTGAAGTATCTGTGTGTATGTGTGGGATGGAGAGTACTTCGAGATACTAAAAACGGTGGATATTTTGTTATAACGGTGGTTATCAAATGCA

>Dvv ABC-C T45163_c0_seq1

ATATAAAATTTATTAAACTAGATAATTATCTATATAATAATAACATTAAAAATCAAGCTTCTTGCACTAGCCTATAAAAATAAGTGCTAGAATCAGCCATCAGTACGTCGGGTTTGTCGAATTCCACCACTTCTCCTTGATGCATCACCAACACCCGATCCGAGTCGAAGATGGTCTGTATCCTATGAGCGATGGTTATGACTGTACTACTACGGAAAGCGGTACGTATAGTGTTTTGAATAAGTCTATCAGTCTCTTCGTCAACGTTTGCTGTTGCTTCGTCAATGCACAGTATTTTAGCATTGTGAAGAACAGCCCTGGCTAAACAAACCAATTGTTTTTGACCGACGGAAAAATTCGAACCACTTCCGTCAATCTTGTAATCTAGTCCTCCCAAGGAATTAATAACATCAGTCAAGTTTACTCTTCCCAGAGCGTTCCATATTTCGTCGTCCTTGAACTCTTCCAGAGGATCCAGGTTTTCTTTTAAAGTCCCGCTGAACAGGAAAGGATCTTGCGGTATGCAGAACATCCTAGATCTTAATGTTGCAAGCGATATTTTCTTGATATCCACTGCGTCGATAGTGATCACTCCTGCAGTTATATCTACTAAATTAAACAATACAGAAATGATGGAGCTCTTTCCAGCGCCAGTTCTACCAACAACCCCAAGTTTCTCGTACGGCCTGGTATCGAACGATAGATTCATCAGTGATGGTGGCAAATGTTTCCTATAATGTAAGGTGACATGTTCAAAAGAGATAACACCTTGACTGGGCCAAGCGAATGGAGGATCCATAACAAAATATTTACTTTCCATTGGAATGGATTCTACGTATTGGTTGATGCGTTCGACGGCCACCATTTCCCTCTCTGTCTCCGTGAACGAGTTGACGACGCCGCTGAGTGATCCAGTGATTCCCAGAGCGTACGAAATGGCCAATCCGATAAAACCTGGATCCGCGATATCATACTGATGCTGAATGACGGCAATAAAACTAACACCTGTAATTATGATCACTCCTATAAACTGTAATCTAAGACCAAGCCATCGAGCTGCCGCTTGACTGGCGAATTGAGCTTTCAGATTAGCTTCTAGATGTTGATTGTTGTCGTGTTTGAATTTGTGGATCACTCTCATCGCTCTAATTGTAGTCAACCCTTGCAAAGTTTCATTAAAATGGTTATATATGGGAGAAAGCGTTACACTAGATATCCTTTTTAGTTCCCTTGACGTTATCCTGTACTGATTTAATAAATATGTATATATGGGTACTAACGGAATGAGGAAGACACATATCCAAGGCAAACCGTACATCGTTATAAAAAGAGAACCTAGTAATCCGAATAACTGCGCAAGAAATATATTAAGAATAAACGGCAACGAATCATCAACAGTGTAGGTATCGCTAGAAAACCTATTAATGATTCTTCCTATTGGTGTTACATCGAAAAATGTACATCTAGCTCGTAACACGGTCTTCAATAACACCTTATGAAACTTTGTAGCTGCCAGAATTCCACCATAAGCAAATATAAACGATCTTATTAGCGTGAATAGCGTATTGACACACGCCAACTCGACGTATACTTTTAAAAACTCTGACATGTTATCATCGTCTTCTACGTAATCGATATAGTCAGTCGCCGTCGCTAAGTATACTGTTAAGTTGGTAGAATTGGTAACGGGGCTGGTTACACCATTGGCTAACCACCAGTCAGTGAAATTCCGTGACACTTGCATTAAAGTCATCGAAATCAGTATTGAAATGCTGACAAAGTGGCTGATGCCTTTCCAGTAAGAAATTATCACAGTAAAATCTAGATTACCCCGTTCGCTGACTTCTTTGAACAGTTCGATATCCTTATCCTTTTCTTGGGCGTTGTCCACGCACAAACTATTCTCTAGAGTGCACTCAGACGATGTATATTCAGATTGAATACTATCCTCCAGTTCTAAATCTACGGGTAAAGTGTCGTCTATGTTGCGGAGTACATCGACAGGTTTGCCAATTGACTTGATAGCTCCGTTTTCTAACAACAAAATCCTATCCGCGTGCAGCAGATATTTTACGTGGTGGGTGCATAGTAGTCTAGTTTTTCCTTGGAGAAGTCCAATGATGCAATGATTGAAAATGTGTTTGCCTACTTTGACGTCAACTGCTGATAGAATATCGTCCAAAAGATATACCTGCTTCTCTTGGTAGATCGCTCTAGCTAGGGCTATTCTAGCCTTTTGACCCCCGGAAAGAGTACCACCGCTATCTCCTACTTCTGTTTTGTCTCCTGATGGTAAGATCTGCATATCTTCGATCAGGCCACAAGCAAATATGATATCTTTGTATTTCTTCTCTTCGTACGGTTTTCCGAACAAAATGTTGTCCCTAACTGTTCCTCTCTGTAACCATGGTTGTTGTGTTACAAGTCCAAATCCTGTTTCTACTTGACGTACAGCAAGACTGCCGGATTCTACAGCGAGTTCACCTAATATTGAGGAAAGCAGGGTAGATTTGCCACAACCAACTGTACCCATAATGCCAATAAATTCACCTTTCTTTATCTTCAAAGTTATATCCCTGAGCTTGAAAATAATGGTTTCTCTTCTTTGGACACTGCTTTCGCCTTGTCCCCTTTTGGATAACGTTCTTTTTCCTTTCCCTTTTCCTTTAGGCACTTGTACAACGGGAGTGGCTTTCTCCCAGCTGAAACTGGCATTGGAGATGGATATTTCTGTATTTTGGTCTGTATTATCTTCTAGTTCTTTGTGGTCGTAGATTTCATTTAGGTTCATATATGGAAGATCTAGTAACTTCTGAATCCTCTTCAACGACACCCAGGCTTCAGTCAAACCATTCAGTACCCAAGGAAAAGCGTTAAGAGGGCTCACCAGCATGTTCAGAAGGGCTATGCTTGTAAACACAGTGGCAGCTGTCAATTTGTTTCCCATCAGAACGTAAGTAACGAACGTCAAAATGGAGATGACCACTGGAGTAGTAGCCCAGAAGTATACGCAGAGGGCGTCCAGGTATTTACGACCTTTCAGGTACTTCAGTTCGGCGTCCCTTTGCCTAGTAATAAGTCTTATAAAGTGTTGTTCCCAAACGTACAATTTGATTGCCTTAATGCCCCTGAGTATTTCGGAAGTCAACTTCACTCTGCCGTCCTTTTGTTCCATCATCTTGGTACTAAGATCGCCGATTTTGTTCGCTATGCATTTATTTATGGGAATGAGGATTATGCTAAACATAACACCAGCTAAAAACGCCAAGCCCACTTGGTTGTACAGAAGGTAAAGGGATACAAATAGCTGGAATGGTATACTCCATACTGCGTGGAAACTTGGACAAGAATTAACAATCCGGTCTGTATCTGTAGACATGTAGTTCATTATCTCCCCCACAGATAATTTAGACATTAAAACCGTAGATCTCACTGATAAAGTTTTCCTGTATATAGTATTGATTATAGCTCCCCTCATTTTGAGACCGATCACAGACATCTTGAAATTAAAGTGGGAATCACAGAGAGCAGACATAGAAGTGCTAACCACCAACAGCACAGCATACAGATAGCCCCATTTGATATCTATAGATCTCTTTTCTATAAAGTCTACTAATTTGTTCAACAAAATTGGTCCAGCAAAATTACTACAATCCGCTACCAACCTCAAGATACCAACGCTGTAGAATTGCCAGGCGTAGCATCTATGAAGCGCCGCTAACAAACTAGAACTTTTGCGTGGCTGAATAACTATTACTTCTCGGGAAGAATCCGTATCGTGTTCAATCCTCAGGTAATTTTCTAGTCTATTACTATTGTTGTCGCTACTTATTGAGTCCGGTAGATCGTACAAATCGTCAGATGTAACTATTTTATCTTCAACTCCTTTGTTTACTAGAGACTTTACCCAGGAAAACGATAAACGGGACAACCAGTTAGTGTCTTCCATAGCAACCCCCAGATCGTTTGGATCTCCCTCTTCACTGAATCTGCCGTAGGCATTGGCGTTTATCAAGGGCTGGCGCTCTGTGATCTCTGTATACCTCTCAGGAAAATTTAATGTGGTGGTGCTACCGCCTGGTATCAAACTAAGGGCATAAATGATCTGCAAGACGGTGTAATACAAAGTAATACCTAGCTCCATTTTGGTATTCTCTGTTGGTCTTTGCGAATTTTCCAAGTAGGCACTTCTCAACGATACGATGGTCATCGCGAAAACCATACACCAGACGATACCCATTGCAGTAGGACCTCTTTGACTTTTACCTAACCTCGATCTTAGTCCTACAGTGTAGAGGAAATGGGAACACCAAGAAATGGCTTGGACTGCGCAAACCAGAAAGGAGGCGTTTTCTCTGTTCGGAGTTTTCGTCATAATAGTATTGTATGCGTGCATTATTGGATAGAACATCAGTAGGAGACTTACTACATTTCTGGTTATGATACAGTATCTTTGAAGCTTTCCTCGAGAAACAAAACCAGTTTGACGCCCAAAGTAATATGAAGATAGTACTGCAAAAAGGGAAAGTACAGGGATCTGGAAACATAGTTGCTGGAAGCACTGGCCAAAATCGTGTCTTTGCTGTGACCATATTTGGAACCCTTCGGGACCACAGAGGCTGGTCCAGTTCCAATTACGGCCTATGACATCACTTGTCATTTTAAATCATCTGGCACCATCTGCAGGTAGCTGGAAGTCCCAAGCAATGGCTTTTCTTTGTTTCTTAATTTCAGTTATAACTACTATGTGTCACATTATTACCTCAATACTTATTATTATTAGTTAATAATTAATTAGTAAAAGTAAAAGTAAAACAAAACAGCTGAGCTGATTGATGT

>Dvv ABC-C T49618_c0_seq2+T79857_c0_seq1*

GCCCTCTCGGCGGCCCTGCTAAATAGCACAACACAGAGAAGATTGAAGAACTTATGGTCCAGGAGTGAATGCTAATAGGCTGAAGAAGATGATACTAAAATTTTACTTAAAAACATTTTGATCTCGATAATAAATTAAAAATAAAAGTATATAGTAAAAAACAAGAAACTAACATTTAGCTATGTTCAATATTAAGTGTCTTGGATATTGAAGTGTTAAAAACTGTTAATTTGGAGGAAATTTCTACAACTGATTAATTTATTTAAGTGAAACGCACAAACAATCAGTGCCCCTATCAAACTACTACCTAAAACTGTCTGGATGAAGATGACTCATAGCTTCGTAACTATATTTAGCTAACTGATGAAGAGTTTCAGCCATAGTTTTTCCTGTCTGCTGAACCATCCCATAAAAGATACCATCTTTATCTTGTAATAGTACGTGAGGGTGGTCAAACTCCTTCAAAGTTCCAGCGTCCATTACTAGCACCTTATCTGAATCCATTACAGTATTCAGTCTGTGAGCTATAGTTAGAACCGTGCATTCTGCGAATTTGGTTCTTATGGTTTGCTGGATCAGAGCATCTGTTTGTGGGTCGACGTTAGCAGTGGCTTCGTCGAGGACTAAAAGTTTGTTCTTTCTGAGGATGGCTCTTGCTAGACAGACAAGCTGGCGTTGGCCTACGCTGAAGTTGGTGCCACCTTCGGACATTTTGGAGTTGAGACCGGCTACCAAGTTTTCGACGGCTTCTTTTAGTTCTACTTCTTCTAAAACGCTCCAAAGTTCAGCATCTGAAAATTCATCAAATGGATCTAAATTCTTCCTCATAGTTCCCGAAAACAGAACAGGCTCTTGTGGGATAATAGAGATCTTAGTTCTCAAATCATGTAATCCAATCTGTGAAATGTTAACTTCATCTATCAAAATATCGCCGATAGTTTCAGTTAATTGGAAGAGAGCATTAATAAGTGAAGATTTCCCTGCACCTGTCCTTCCGACGATACCGATTTTTTCTTTTGGTTTCACCACGAAGCTCAAATCCTTCAAAACGAAGGGATCCTGTGGGAAGTACTTTAGGTAAAGGTCCCTAAATTCCAACTTGCCCTTCTCTGGCCAAGAAGGTTTTGGTTTCTTGTCAGGCTGAGATTCCAACGCGCCTTCTTTTTCTATATCATTGTACTGGAGTACTCTTTCTACGGATGTCATTTGGTTCTCCAATTCGGTGGACTGCCTCATTCCCCATTGGAACATTCCAGTCAGTCCAATAGCTTGGGTAATAGCTAAGCCAACGTTTCCTCCGAATTTTTCGTTACCGATAACTAGAAAACTGAATGTAACAAGAGTAACGTAAACCATACACAGTAAGTCCAACCAGTACCCGAAAGCTCTGGACGTTGATATAAAAGCATACCAAGCTGAACTATGCAAATCTTGATGGTTATCAAATTCCTTTTTCAAAATTTCTTCAGCTTGAAAAGCCCTGATTGTTGTTAATCCCAAAAGAGAGGCATTTAAATGGGAAAATACAGGACTTCTGGTGGTTCCTTCAATTCTCTTTATGTTTCTACTAGTTCTTAAGTAAAAAATTCTGAAGAAAAAGAAGATTATTCCAATAGCTACAGTCGGTATCATTAAGAAAGGAGTTACTACTGCTACAACTACTATAATACCCAAAAGAGCTAAACCGATCTGTAGGCAATCTATTAAAGCTGTCGGCAAAAGTTCGTCAATGGATCCCATGTCTTTGGAAAACCGGTTTAATATTCTTCCGGAAGCATTAGTATTGAAGAACCTCATGGTGGCTCTGGTAATGCTGACGAACATATTGTCATGGAGTCTGGTAGAGGCGCGCATACACACAGCAAAGAATGTGAACGATCTGACTAACGTTATAATTACCAACAACACAATTAGACCCGAGTAAATGTATATCGAGGTTTCTCTTGAAAAGTGCCAAAAGTTATTGGTGATGGTCTCATTTGATGAAAGTCCTGTTGATTGTGTTGAGTTTGCGGCACTGTCTTGTTGTTCTAAATTTACCCAATAAGATAAGTAGTAATCTGAGGCACTGGCAAACATTTGTGATAACAGGAATAAAAAGAAGAATATAAAAATAACACAACAATTTCCTCCCGCTCTGAAATAGGCTCTGTATACATATCCACCAATCGTACCGGTGCCTTTTTGTTCCTGGACTTCTTTGGGTTCTTCCACTTCTACTGAAGCAACACTTTTTATAGAGAGCTGACGCATGACTTCAGATTCTTTAGTTGTTTCCTCTTCCTCCGTAGATTCCCCGAGTGATTTTGTAAAATCTAATCCACTCGCTTGTAATTCTCTAAAAGACCCTGTTTTAGGAATGCCATCTTCCAAATACAATATATTGTCCAATTCCTGTAAGTACTGTATTTGATGAGTAACTAATATCACAGTTTTATTTTTTAAGTAACCCGTAATGCACTGTTCAAATATTTCTTTCCCGACGTGAGTGTCCACAGCGGATAAAGGATCGTCTAGCAAATAAACGTCGGCTTGCTTGTAGACGGCTCTTGCCAGATTGATTCTTGCTCTTTGACCACcacTGAGGGATACACCTCTATCTCCAACAATAGTCTTGTCTCCATAAGGCAAAAGTCTAAAATCCCTTTCTAAAGCACACTTTTTAACCACAGTCTTGTATCTTAATTTGTCCATGGGCTGTCCAAATAATATATTTTGTCTAACGCTGCCTGCAAATAACCACGGCTCCTGCGATGCATAACTAACTGTACCATTGACAGTAACAGATCCATAAACTAGTTTCAACTCTTGCAATATCACGTGAAGTAAAGAACTTTTTCCGCTTCCAACTGGACCTATTACTCCTAATAGTTGACTGGGGTAAACCTTTATAGTAATACCTGTTAAAGTATTATCCACAGACATTTCACTCCACTTGGCAGTAGCATTGTTTAAAAAGACTCCCAGCTCCCCTTTCCTCTCAGATTTCTCAACTCCATTCCCAGATTTGGATAAATCCAAACTTTTACTACTGCTCATAGAACATCGTCCAAACTTTTCTCTGACCATATCTATCTCTTCAAATTGCATGAATTTGTTCAGTCTTTGGATAGATATTCTAACTTCTGCGACCTGCGCGATACCTTGAGGGAAGAACACCGTCAGAGATTGTCGAAGGACGCTGTAGAATGATGTAATGACGAAGACTTTTTCGGCGGAGATGTCATATCCGGAGAGGACGTATGCTAAGATGCTGACGAATAGTGATATTCTTGTACTGTACATTATAAATGAAAGAAATATGCCTCGCATAAAGGCAGTAGCTCGGAGTGACTTGATTTCATGCCTTCTGGCTGTAGCTACCAACTGGGCAAATGGTGTCTCCCATGCATACATTTTAATTACT

>Dvv ABC-C T47333_c0_seq11

CAAAGATAAAATGGAACAACTATAATAAAGATATTGTTTGCACATTAATATTCTTATTTTCATATTTCTTACATTGAAACAGGGGCAATCTCAAATCTCGCTTTTGAGTCGATTGTTTTGCACTCAAGTGTAGTTTAAACTTTTGTAATACTTCCAATCGTTACGGAAGGAATAACTTTACAATACACTGTTCAACTTCTGTCTATTGTATCGTCATTTTCAACGATAAGTAAAACAAAAAATTTTAACTATCTATTCCCCCTTTTTCCCCTTTATTCCGCCGTACTTCTTATTATAATCTTCCTCTGCCACTGCCTTCAACATTTCTTCCATAGCTGGTCCCGTCTCTCTAAGCATTCTACTAAAATATCCGTCTGGGTTTTGTAGAAGTTTAAAAGGATGGTCAAATTCTACTGCTTCTCCGGCATCCATTACCAGCACGCGGTCTGAATCCATAACAGTATTAAGTCGATGGGCGATGGTAAGTACAGTACAGTCTCTAAACCTCTCACGGATTGTTTTCTGGATTAGGGCATCAGTGCCTGGGTCTACATTGGCAGTAGCTTCATCCATAACAAGAACCTTATTGTTTCGTACTATAGCTCTTGCAAGACAAAGGAGTTGTCTTTGACCTGCACTAAAGTTTGAACCTCCTTCGCTGACAGTTTGATTTAAATCAGTAATGGCATGTTTTAGTTCAACGCTTTCTATTGCCTTCCAAAGTATTTCATCGCTGTATTTTTCAAATGGATCTAAATTGTATCTAACTGATTCCGAAAATAAAACGGGCTCTTGAGGAATTATAGATACATTCAGTCTTAAATCTTTTAAGCCTATTTCTGCCGTATTTAAATTATCTATGGAAATCGTTCCCTCGATTGGGGCTAACCTGAACAAAGATGCGATCAATGTTGATTTTCCTGCTCCGGTTCTCCCTACTATACCAACCTTTTCTCCAGAATTGACTTCAATATTCAAATTCTTTAAAGCAGGAGGTACATCAAGGGAATATCTAAGGAAAGTATTTTTAAAAATAATTTTTCCTTTCTCTGGCCAATCTCTAGGAGGTTTGGTTAAAGATTCGAATGGTCCTTCCTTCTCTAACTTTGTGTATTGCAACACCCTTTCTACACTTATCATATTGCTAATTGCCTCCGCAGTTTGTCGCACTCCATATTGTAACATTCCGGTTAAAATTAAGCTTTGGGATAAAACCAAACCTACATTTCCTCCCGCAGTGCTATCGACACTGAAAATTAAGAATTGATAGGTAAGTAGTCCCAAAAACAGCGTACTGATAATGTCCAAATAAAGTCCAAAGGCTGTACTACTAACAAGAAAACAGACATATGTACCAGTATGCTGATCTTGCAAAACGTCAAACTCCTTTATGACCATTTCTTCAGAATTTGAAGCTCTTATAGTTGATATTCCTTGTAACGTAGCAGAAACATGTGAAAAAACTGGGGCTTTTGCTACTCCTTCCCATCGTTTAATGGATTGTGCACTAGCTAAATAAATATTACGGCAATAATAGAATAAGAATCCAATCAGTATAGTAGGAATAATCATCCAAGGGGTTACTATAAATACCAAAACTAAAACGCCTACTAAGACCGTGAATATCTGAATTGTGTCAATACCAGTCTTTGGCAACATTTCATCTATTGCTCCCATATCTTTTGAAAATCTGTTCAATATACGACCTGAAGGATTTGTATCAAAGAATCTCATAGGAGCTTGAAGAATATTACTGAACATTTTATTATGTAATGTTCTTGAAGATGACATACATACTATGAAGTACAAGAAAGACCTCAGGGTTAATAAGATCATACTGGAAAACATCAAAATGGTATAAATGATTATGTATCTGTTCTGATCATAAACTTGCTCAGCTTCTATTCTTACTGTAGTAGTGTCGATCACAGAGGATATGGTTAGCATGGATTCCATGACAGAAATATTCGTGGGTAACGTCGGTAGTGTTAATAAAACCGGATCTGCAGAAGTCGTTAAGGGTAGCAGAGTAGTTAGTGTGTTTGTCAAATTATTTCGATTTGTTTCACTAGTATTTTCAACATTTGCGGCCAACATATCAGCGTAGTATTTTCCTTCGACATTGGTCCAGTGTGTCAGCCACAAGTCAGTTATACTGGTAAAAGCTTGCCCAGCAATGAATGCTACCGCTGTGAATGTTAAAAAGAAAACACCTGCACCAGATCGGAAATATTCGACAAATATTGAGAATGGGACAGTGCCTTGTTCAACTTCCTCTTCTCCTCGTTCGTTGGATTTATCTTCTGGCTCCTCGGAAACCGATAATGAAGTATTAGATTGATAATGCTCTGAACGAAGTGTTTTCTCACTGATGGTGCTTTGTGATCTTTCTACTTTTGATCTCTCTTTTTTAACTTCATTTTCATCTTCTTCTGGTTCAGGTTCTTGTTGTAAGACATTAAGCTCATCCTGAGATAATGCACTGAAATTTGCTGCTTTTTCGATTCTGCCATTGTTTAATATAACAATAATATCAGCTTTCTTCATATACTGAAGCTGATGAGTCACTAAAATTCTCGTCTTATTTTTTAAATATTTCACCATACATTCATCAAATAGATGTCTTCCTACTTTTGTATCCACTGCAGAGAGTGGATCGTCAAATAAATAAATATCAGCATCTGAATATACTGCTCTTGCTAAGTTAACTCTGGCTTTTTGGCCACCACTTAATGCTGCACCGCGTTCACCTACTAAAGTTTTATCGCCATGCGGTAACTCCTTCAGGTCTCTTTCTAGTGAACACACACGAATGACTTCTTGGTATCTCTTTTTATCGTAAGGTCTACCGAACAAAATATTCTCCCGTACATTAGAAACAAATAACCATGGTTCTTGACTAGCATATGCAACATTTCCATAAACATTCACCTTTCCATTCGTAGGGGACAACTCTTTGAGCAAAAGGTGTAATGTACTACTTTTCCCAGATCCTACATTTCCAACAACGCAACACAGAGTTCCTGGCGTTAAGTGTAAGTTCAAATCTACTAGTGTTGGTGTTATTGGATTCTCTGACCAGCTTGCTGTGACATTAAACAACTCTATTTCGCCTGGTTTCCCTGCTAATGTATCATTTTCTCCAAGTTTTTTCTTCGGCGTGGTCTCTTCCAGAAGAAGGAATTCTTCTATTCTTTTTATCGATACTTTAGCTTCAGCATATGTAGATAAGGCTCTTGGGAAGAACATCGCAAAAATCATTTGAACAGTATTTACAAGTTGAGCCATTGAAAATACAATATCCCCAGATATTCTTCCTTCCATTAGTACAAACGTTATTACCACAATAAATAATGCAAAACGTTCTGTAAAAACAGACATTGCCGATAGCATACCATGAATGTAAGATGATTTTGTGATGCTATCGATTTCCAACCTCCTAGCTAGTTCTACAACTTTCTCAAAAGGTTTTTCCCATGCATACATTTTAATTACTTGAATTCCCGTCGTTATTTCACTCATGAGTTTTATTCTTGTGTCTGTCCTAACAGCAATTTTTCCCCGTAATTGTCCTTGCAGTCTTGATAGGTAACTTTGAAGAGGTAGTGCTTCCACTATAATTGCACCCATTCCTGTAAGGGCAGCCACAAGATTGCATGTTCTGTACATAATGTAAAATGATATCGCACTGTTAATGGGCATTACCCAAATGTAATGTAGGAATACTGCAGCGTAATCAAACCTCTGCAAGTCGTTGGATAAAAGATTGACTAGTTGCCCTGCTGCCGTTTTACCAAGGGAAGTGTGGTTTAATTTTAACAGTTTTCTATACAATAAGGAACCCACTGCAATTCTCACTCTCATACCCACTCGTTGAGTATTTAAAAATGTATTATGAAGAAGGATAACATTTAAAAATGCCATAATGACAACTCCAGACGCTAAGAGCCACCCAAATTCATTAGGCTTGATTCCATGCTTTTTGTCGAAAAAATTAATATATTCTGCGAGTACTATGGGTTGTAACATCCTAATAACTATAAACTGCAAAAATATCATCACACCAGATGCTGTATACATTTTTAAATATGTTTTCACAATTGCTGACTTTAAACTGGGTTTCTTTTTATTTTGGCTTTGATCACATTTTTTAATTTGTTCTTCCCAATTCTTTTGAAGTTGATTTCCCAAAGATTCAGACATATCAGGTTTTGTTGCATTGTATATGTCTTTAAGTTCAACATCGTTTTTATATCCATATTTAAAGAACGGAAGAAACCACAAATTCAGTCTGGTAACAGGTTTCCTTACTCTAGTGGAACGCCTTAAAATGGGTTCCTCAGTTTCAGTTTGAGCCCCAGGTACACTATTCGAACTTTCCATATTGTTTGAAATTTCGGATTCATCACCCAGATAGCATGCAATATTCCTGGGACATTCTCTCAATGTCCTGATGTAAATTTATTTCCAGAGCATGTATCCACTCTCGACCAACCAGTGAGTAGCCATCACCATCCACCACATACAACTTTAGAGTGTGCTTTAATTCCTGGTGCTCAACCTCCACTGAAACTAAACCTAAAACATTCAAATTATTTTTGCAGTATGTAGTTAATTTAACATCCGATTTTTGTAATTGTAATGCAGGAAAATATTTTTCAAATATATTTTTATTTATGATTGTAACAGCAGCGCCAGAATCAATTTCAAAATTTAAAATATTACCATTCACTTTTAAATTTATAAAAAATTTATCTTTGCAATTTCCAGCATTTACTTCTAAGACCTCTTGAGCAGAGTTTACTGAATTTACTTGCCTGTGTGAATTTTGTTGTGCCTTTAAACAAACTTTTTGAATGTGTCCGACGTTTTTACAAAAGTTACAGATTAAATGCTTTTTATTACATTTATCGGCTAAATGCGAGGTGTCTCCGCATCTATAACAAGCCCTATTACGGTTATTTGACATTGTAAAAGTATTATTAGGAGAGCTACTATGATTATTTACTGTATTATTATCATTAAATTTACTAGTGTTTGTATTTTTATGAAAACTTTTTGCTTTCGCATTTAAAACATTTATACTAGCCTGATTGTAATTATTGTTGTGAGAAAATTGATTACTATCCTTTTCTGAAGTTTCCATGCTTGCTGCAATTTCTACGGCTCGGTCCAAGTCCAATCCCTTTGTTTCTAATAGTCTGGCTTGAATCCTCTTTGACTGTAAACCAAAAACAAACTGATTTCGTATAGCACTTTTTAAATATGTGGAAAAGTTGCAGTTTATGGCAAGTTTCTGTAGAGAATGTAGGTATTCTTGTATACTTTCGCCTTCTGCTTGTCTTTTTGATTGAAACCTAAATATTTCAGCAATTTCAAGTGGTGCAGGGTTGTAAAAATTATCCATTAACTTAACTGTATCCTCATATGACTTGTCTTCAGGGACCTCTGGAGCTAGTTTATCGCACAGTGTATCGTATGCTTCCGACCCCATGTAATGTAGTAAATAGGGCAATTTCATTTCCTCTGGAATTTTAAAAACTTTGTATGCTCCTTCCAGCCGTTTTACCCACCTGGACCATTTTGTAGCTGTCTGGTTAAAAGGTTCAACGGAAAACTGGAATGTAGAAACTTGTGTAGACATTACTGGAGCTGTAGCAGGTGCAACTATCGCAGTTGTAGTCGAGGAACTCGTGTTTGTACCGGCTGTAGTTGGAACGTTGTAGTTGATGTAGTGTCTGTAATATTTGTATCCATTATCCTCGTCGCCAAATGTCAAGTCCTTTGTACTTAAAGGACGTAAGTATTTGTAATGAAATAATACTCGATGTAAAATACTTTATTTTAAAGAATTATGCACATGGTTTCCATTTTCTATCGCAGTATTAAGTATGTGACAGCTGTCGTTCAGATGACAGTAGTACCAACCTACATTCATGGTTAAGGGAGTTTTACACGAACATAACACCTCCTCCCTTGACCATGAATTACAAAAGTTAACTAGAAGTAAAAGTACTT

>Dvv ABC-C D7536

TTTGTTTTTGATTTACAACAATTTATCAATATAGTTTATTTCTTTGTTCTGCAGCTGCAATGTATCAAAAGATCACAAATTTGTTTTCACTTTAATAAGATATTACAAACGTATAACATTTTCATATATTTATATAAAATAGACATCTTAATTTAAGGAACTAATCTTTGTTATCTTCAATTGCTAAATTTTGTGGAACTTCACTGGTTAACGRTCCCTTCTTCTTAATGTAGTCATTCTTTGCTACATTACTTAATAGTTCCGCCATGGCGGGTCCAGTTTCCTGGACCATCTTGCTAAAGTATCCTTCCGGATTTTGTAGAAGCTGATAGGGATGGTCAAACTCCATTGCCTGTCCGGCATCCATGACTAAGACTCTGTCTGAATCCATAATGGTGTTTAATCTGTGGGCAATTGTAAGTACTGTACAGTCTTGGAATCTCTCACGAATAGTTTTTTGGATGAGGCCGTCAGTCTGTGGATCTACGTTAGCGGTAGCTTCATCCATAACGAGAACTTTGTTATTTCGTATAATAGCTCTAGCGAGACATATAAGTTGTCTTTGGCCAGCACTGAAGTTAGATCCTCCTTCACTGACAACTTGATTTAAGTCACTAATAGCACCTTTGAGTTCAACGTCTTCTAACGCTTTCCATAAAACCTCATCACCATGTTTCTCAAAAGGATCTAAATTATACCTGAGTGAAGCGGAGAACAAAATTGGTTCTTGGGGTATGATTGAGATATTTAATCTTAGATCATTCAAACCTATTTCGGCAGTATCAACATCATCTATTGATATAGTACCCTCTATCGGTGCTAATCTGAAAAGTGACGCAATGAGGGTTGACTTTCCTGCACCAGTTCGTCCAACAATTCCAATCTTCTCGCCAGAGTTTACTTCTATATTTAAATCTTTCAATACAGGTGGAAGTTCTGGAGCATATTTTAAGTACGTATTCTTAAATATAATTCTTCCTTTTTCGGGCCAATTCCTTGGTGGCTTCTTGCCAGGTAGCGTTTCGAAGGGCCCTTCCTTATCCAGTTTAGTATATTGCAAAACTCTTTCTACGCTAATCATATTACTCGCCACTTCTGCACTTTGTCGCACTCCATATTGTACCATGCCCGTTAATATGAGACTTTGAGATATGACCAAGCCCACGTTTGCACTTAAAGTATTTTCTGTTCTAAAAAGAAGAAATTGGTAAGTCACAATCGCCAAGAAAAAGCAGCTCATGACATCCAAATAAAATCCAAAAGCGGTACTGCTGACTATAAATAAGTACCACGTGCTGGTGTGCTGATCTTGTAGGATATCAAATTCTGTAGTTATCATTTTTTCAGCGTTTGAGGCTCTAATTGTTGTTATACCGTACAGTGAAGCCGATATGTGGGAAAACACCGGAGCTCTTGACACTCCTTCCAGTCTTTTTAACGATTGTGCGCTTGTTAAGTAAACTACACGGAAGAAGTAGTAAAGAGGAGCAAGTATTACTGCTGGAATGATCATCCAAGGAGTCACTATGAACACCATAGCTAAGATACCAATCATAACCATAAAAATTTGAATAGCGTCAATTTGACAGCGTGGTAAAAGTTCGTCTACTGCACCCATATCTTTTGAGAACCTGTTTAAAATTCGCCCTGATGGATTTGTATCGAAAAATCTCATCGGTGCTTGAAGAACATTGTTAAACATTTTATTATGCAAAACTTTGGAAGCAGTCATACATATCTGGTAATAAAGGAATGATCTCCACGTAAGAAATACAACAGAAGCTAATATAATTCCAGTGTACACATAAATATAATATTCTGTAGACGGAATGTCATTCAGTTCTTCTAAAGTAGCATTTCTGATGHGGTCTGTTAAATATCGTTTCTGACTTAAACTTAGAAGTGAACTATCTGCAGATGGTGTTGAATCTGTTAATAACGTAGCAGAAGTTGACATCTCATCAGATAATCCAGTAGACATTAGTAATGGTCTTTCTAAGGTTTTATTGGAAGTTGCATTGTGTAGGGAATCACTAAGATGGTATCTTTTTGCCTCGTTATTAGTCCAATGTGTAAGCCAAAGATCTGATGCATTCGTAATCATTTGAGCGACGAGGAATATAAAAATTGTAAAGAATAAGAATAAAACTCCACCGCCTGATTTCCAATATTCCACATAAGTAGCATTTGAAATGGCACCTTTTTCGATAAGTTCGTCTTCTTCGTTCGGATCATCCGAGAATACAGAACTAGCCAAAGAACTCATAGACTGGAAATGAGGTACTGTCGAATTTTTACGAATCTTATCGCCAGTCTCTGGTACCTTTTCTTTCTCTTTATCATCGGCCTCAGGTTCTTGTTGAAGAGCATTCAAGTCATTTTCCGACAATTCGTTAAATTTGGCTATTTTCTCAATTTGACCATTGTTTATAATAATTATCAAATCAGCTTTCTTCATAAACTGGAGCTGATGTGTGACCAAAATTCTAGTTTTTCCATATAAATATTTCGATATACATTCATCAAAAAGATGTCTTGCTACCTTTGTGTCGACCGCCGATAGCGGATCGTCGAATAAGTAAATATCTGCTTCAGTGTATACTGCTCTGGCAAGATTAATTCTTGCTCGTTGTCCTCCACTTAACGATGTTCCTCTTTCACCTACTAAGCTTCTATCACCAAAGGGAAATTGTTTAAAATCTCTTTCTAGAGAGCACACCTTAACTACGTCATGGTACCTGTTTTTCAAGAATGGTTTTCCAAAAAGTATATTTTCTTTCACGTTTGAAACAAAAAGCCAAGGTTCTTGGGAAGCATAGGAAATTTTCCCGTTAATCTTCATTTGCCCCCTACTTGCCGGTAATTCCCTCAGAAGTAACTGCAGAAGACTACTTTTTCCACAGCCTACGTTACCAACGACGCAACATAAAGTTCCCGGTTGAATGTGTAAATTGAGATCCATCAGAGTGGGTGCAATTGGTTTCGATGCCCAACTGGCTGAAATATTAGTCAATTGAATTTCTCCTAATTCGTCTGGGTTGGAGATTTTTTCCGGCTCCGGTATTTTTTCGTTTTCATCTAATAAAAGAAAGTCTTCTAACCTTGTAATAGAAACTTTGGCTTCAGAATAAAATGACAAAGCTCTTGGGAAGAATATCGCCATGATCAATTGAACAGTGTTGAATAACTGAGCTAAAGAAAAAACTACATCTCCCGTTAATCTTTGTCCAACTAGTACAAAAGTAATGACTGTGATGTATAAAATCATACGTTCTGTGAATATGCCCATTGCTGATAGGACGCCATAGCAATAAGAGGTAGTTGTTATAATATCTATTTCGTATTTTCTAGAGAGCTCTACGACTTTTTCGAATGGTTTTTCCCATGCATACATTTTGATGACTTGTATTCCAGAAGTAATTTCGCTCATCAGTTTTACTCTTTTGTCTGTCTTTAGAGCAATTTTGTATCTTAGTTTTCCTTGCCATTTCGAGAAACTACCTTGTAAAGGAAGAGACTCCAGAGTAATGAAAACCATTCCAGTAACGGCAGCGATGATGCCAACATATCGGTATAAAATATAAAACGATATCCCTGCAGTAAGTGGCATAATCCAGGCATAATGTATAAATTGGGCGGCTAAGTCAAATCTTTGGACGTCATTAGATAAGAGGTTAACCAGTTGCCCGGATGCTGTTTGACCCAGGGAATTGTGGCTTAACTTCAAGAGTTTTCTATAAATTAACGAACACACTGCTATACGCACTCGCATTCCTACCCTTTGGGTATCTAAAGTACAACTATGCGTTATGATAACATTAAGAAAGGCCATTCCGATTACACCAGTTGCCAGCCACCAACCAATGTAAGCTTCTTGATTACTGTCAAAATAGTTTATGAATTCGGCTAATACTATCGGCTGAAGCATTCTAATAACTACAAATTGAAAACCTAATGCTATGCCAGATCTGGAATAAGATTTCCAAAATGTCTTTAATATGGCATTTTTTAGGCTAGGCTTCTTTTGCTTTTTGTCTTCATGATTTCTAATTTCATCTTCCCAATTTTTTTGCAACGCATCCCCAAGAGCGGCTGATAAATCACCTTGTGTGGTATTATATACATCTTTTATTGTGATGTCGTGTTTGTATCCAAATTTAAAAAACGGCAGAAACCACCAGTAAAATAGTTTTGAAATAAGATTAGCTTTTTGTTCCGGTGATACTTTAACGTATTTTTTGGTTGAATCCATCTCGAACTTAAGTACTGTTAGTTAAGTAAGGAAATATCACAAATGTTTTTGACCAAAAACAAAGTACATATTTGCACTCGGCCCTTTGATTTTCCGACTACACGCCTATGAAGAGAACTAGGCCTACTACAATAACAGTTACTGTAAATGCACTTCTTCACTTTTGGTACACTTTTCAACAAAGATTTATTTCATAAATTATCAAACATTTTTATAACCTATAGTATAGCACAGTCGGTGTTTTAATCAGCTAGTTATCACACTCGCACTTATACTTATCACTTATCCACACTTTATAATCTACAACCTAATACAAACAGCTCCGCTCCAACTACTATCGACTTAACATGTTCGTCT

>Dvv ABC-C D20002

TTATGATATGATTGTGTAGATATTAGTGACAATATGGATATAGGGTACAAATTGAAGGCAGAAAATCCCAAACAAAATGCAAATATATTTTCAAAGATATTTTTCGGGTGGATGATACCACTAATAAGGAGAGGAACTAAACAGAATTTAGAAATAAATGACTTATACAAAACTTTAAAAAAAGATCAATCAAAAAGACTAACAGATGCTTTGGAAAAAAATTGGCAAAAACAGGTTGATAAAGCGAAGAAGAAGGGAGGAAAACCCAGTGTACTTATGGCTATATCAAGGACGTTCGCTTTTGAATTTATGATGTACGGAATACTATGGGCCATTCAAAATGTAGTTCTAATGTCTCTAAAACCAATATTGATTGCACAATTGATCGAGTTATTCACTGACGATACTTCTACCAGATTTAGAGAAATGTATTTCTTCAGTACATCATTAATTTTAGTGTCACTTTTAATTGTATTCTTCTTCCACCACACGAATTTTGGTCTGCAAGCAATTGGAATGAGGATAAGAGTTGCGACTTCATCCTTAATCTACAGAAAGATTACTCGGTTAAATCAAAAATCTTTGGGCGAGACTGCTACAGGACAAATTGTCAATCTACTATCAAACGATGTCCAGCGATTTGATATGGTGGTGGTACCGTTACACGCGTTGTGGGTGATGCCGCTTCAGGTGGCCATACTTATGTTTATAATCTGGAATCAAGTTGGCATTTCGTCTTTAGCAGGTGTTATTTCGATGGCAATCATTGCTTTGCCCGTACAAGGTTATATGGCAAAGCTCATGGGTATGCTTCGACAAAAGGTTTCCGGAAAAACCGACACCAGAGTGAAACTAATGAATGAAGTCATTGGTGGTATACAAGTGATAAAGATGTATGCCTGGGAGAAACCATTCGAAAAAGTCATAAAACAAGCTAGATCATCGGAGATTGGTGATATAACAAAGGCATCCTACTTGAGAGGTGTATTTTCTAGCTTCATCGTATTTTTGGATAGAGTGGCTCTATTTTTCACTGTTATGACCTACGTTTTACTAGGTAACGTTATTTCCGCTGATATAGTTTTCTCATTGGCCCAAACCTTCAATATTCTACAAACAGCCATGGCCATTTGGTACCCAGTTGCAATTAGTGTTGGAGCGGAGGCTCTAGTTTCAGCTAAGAGAATTCAAAACTTTTTAATTATGGAGGAAAGAGAAGAAGCGTCTATCGAAAAAATAGATAAGCCCGGTATAATACTATCTAACGTGTTTGCTTCTTGGACAACTAAGGGACGTACTTTACAAGATATTTCATTCCAAGTTCCACCAGGTACTTTGTGCGCTGTAGTAGGACCAGTAGGTGCTGGAAAAAGCTCGTTGTTACAGCTCCTTTTGGGAGAGCTGCCACTAAAACATGGAAGAGTTCAACTGGGAGGAGAAGTTTCCTACAGTTCGCAAGAACCTTGGCTTTTCCAATCAACTGTACGGAACAACATTCTCTTCGGAAGTCCCTACGAAAAACGCTGGTATGAAAAAGTGGTTAAAGTGTGTGCATTGGAACGCGATATGGAACAGTTTCCACAAGGAGATAAAACTATTGTTGGAGAAAAGGGTGTTTCACTTAGTGGAGGACAAAGGGCTAGAATAAATTTAGCACGAGCAATTTACAGACAAGCTGATGTTTATTTAATGGACGACCCGTTGTCTGCTGTTGACACACACGTTGGACGACATTTGTTTGATCAGTGTATTCTTCACCATTTGAGAGGTAAAACTAGAATCCTAGTAACGCATCAGCTACAATATCTGAAGAAAGCTGGTTTAATCGTTGTACTTAATGACGGCAAGATTGAGGCTCAAGGCACCTTTGAAGAGCTAATGGATAGTAAAATGGACTTTACAAAACTCTTAGTTGCTGCCGATGAAACTGGTGAGAAACATGACAAACAAGAGGATGCTGATCCAGAACCTGTAGATTTTACAAGAAAGTTTTCTAGTACTAGAAGATTTTCGGTATTGTCCGACGCCAGTGGCGAAATATCGTTGAGTATGCGCTCAGTAGATATGACTTCGGATAAAAATGGTGAAGAGGAAGAAAGTGCTGGTGACGGAAAACCATTTAAAGACTATTTGTTTGCCACTAAGAATATTTGCTTCGTTATTTTTGTATGCGTGCTGATGATAATGGCGCAGGCTTTTGTTGTAGGTGTAGATTTATGGTTAACATTCTGGACCTCTCAAGAAGCAATACGGCACGAAAATGGAACAATAGTTGAATCAACATCTCCAACTGTAGAAATAATTCCATTACATGGAGACAACTTCAGTTATTCCTATAATTATAATTACAGTTACATACCAGAGAATAACACAAACTCATTCAATAACACGAAATCATTCAATATCAATGATATATTTGATACTGTTAACGTCGATGGCCAGTTGAAAAAAATAATCAAAACGAATTGGGCGTTGTACTTCTACAGTGGTCTAATAGGACTGGCTATCGTTTTTACGCTAACTAGGTCACTGCTGTTCTTTAAGGGTTGCATGATGGCTTCAGTTAACTTACACAGCAGTATGTTCCATATGTTACTTAAGGCTCCGATGAGGTTTTTTGATACGAATCCCAGCGGAAGGATCCTCAATAGATTTTCCAAAGACATGGGTGCTATAGATGAACTTTTACCTATGGGTTTCTTAGATACGATGCAGATCATGTTAGCATTGTGTGGAATTCTGGTAAATATTACTGTTTCTAACGCCTACATAGTTATAGCCATTGCCATTCTTGCAGCAGTTTTCCTAAAATTCCGATCATGGTATATATCCTCAGCTAGAGTGCTTAAGCACTTAGAGGGAATAACTAAGTCTCCTGTTTTTTCTCATATTAATGCCACACTAAATGGAATCATCACAATACGAGCTTCAAATGCACAAGATGTCCTAATAGAAGAATTTGACGAAAATCAGGACGCAAATACCTCAGCATGGTACTTGACAATTGCCTGTATGAATTCCTTTGGATTATGGTTGGACTTCTTGGCTATTATATTCTTAGCAATTGTTACATTCTGTTTTGTGATTTTAAGAAAATTCACCGATGTCAATGGCAGTTTAGTAGGTCTAGCTGTTTCCCAATGTTCAGCTCTTACTGGCATGTTACAATTTGGAATGAGACAAACTGCAGAAATTATTAACCAGTTAACCAGTGTCGAAAGAGTTATGCAATATACTAAACTTGATACAGAAGGTCCATTTGATACACCAGAAGAAAATCGTCCGAGAGGAGTATGGCCAAAACGGGGTCAAATTGAATTTAGGAATCTATCTTTAAAATATGTTGAGAACGATCCACCAGTACTCAGAAATCTTAACTTTATGATAACGCCAGGACAAAAGATTGGTATAGTTGGCCGAACAGGGGCAGGAAAATCCTCTCTTATCTCAGCTCTCTTCCGTCTGGCTCCACTTGAAGGTGCCATTTATATAGATGGAGTAAATACCAAAAATCTAGGATTAACTGATTTAAGAAGAAAAGTTTCCATCATTCCCCAGGAACCAGTACTGTTTTCCGCTTCTTTGAGATACAATTTGGATCCCTTTAACGAGTTTGATGATGACAAAATTTGGGATGCTTTAGAACAAGTGGAACTCAGGGACAGCGTAGACTCCTTGGACTTCCATGTAGCTGAAGGCGGTGGTAACTTCAGTTTAGGCCAAAGACAATTAGTATGTTTAGCAAGAGCTGTTTTGAAGAACAATAAAGTTTTAGTTTTAGATGAAGCTACAGCAAATGTTGATCCTAGGACTGACGCACTAATCCAAGCGACAATTCGAAAAAGATTCAAAGACTGCACGGTTCTAACGATTGCCCATAGGCTGAACACCATCATGGACTCCGATAAAGTGCTGGTAATGAGTTTCGGAAACATGATCGAATTCGACCATCCTCATAAACTCCTTCAAATTCCTGACGGACACTTCCACAAGATGTTACTAGAAACTGGACCAGTGATGTCGGCGCAACTCAAAGATGTGGCCATGAGGGCATACCAACAAGAGTGAAGACAAGCATAGTATAGGAATCTTTATTTATTTCTTTAAAATACCTTTATTTTCTAATAACACGTCACACTACATAAACGTTTTTTTCGGATAAATGTTAAATATAAAAAAGTATCTTAAAAAGGATTTTACTTAATATGAAGATAACGTTGAAAGCGTTGAAATTTTTCTTTTATAGTAATTATGTTTTATAAATACGGTTTACTACATATTTTATAGTTTTATTCTATTTTCTATTTCCTCGGTAAATTAAGCTTTAATAACTCTAATTGCGTCATATAAGTACTCTCTCTCTCCTACAGCGTTACAGCCCCAGTCGGGCCATGTATCCGCCTCCAAGTATCTCTGTCCCTAGCTGCTCTCCTCCAGTTATACACCTTTAAGATCTCTTTAGCATCGGTTCTTCGTCTATCCACCTCCTCCTTTGTCTTCATACTGGTTGACGTCCCACCATAGTTCCGCTAAGCGTTCTATTAGGTGTTCCGTCATTGTAGGTTATGTTATGAGGTGACGTGCTTAGCGCAATCTTTGTATTTTTGCATAATTTGCTATAGAGTGTCATATAAGTACTCTAAGATAATTACGTAGACTACATTTGGTAATGTAATTGGCATAATAACTAAACTACTTACTACCTTGACATTAAATTTTCGTCTTCTTCAAGTACCGTCTCCGCGACAGAAGTCGGCAACCATCATACCTATTCGAATTTTAAAGATGGCTTCTCTAAAATGTTTATTTAATGTATGATTCTCTCACCTTAGATCCTTTCCTGCATAATCAGTTGAAGCACGTACCTCTCCACATGTAATATGTTCGTGATATTACAATTTTCTTATTTTGATTGTATTTAACATTTTTTCATTTATTTTTCGTCATTTCGTCGTTGTAATCTGCCGAGCGTTGTAGTTTCTCATCCGTTTGTGTTAGAGTAATCCTTGTCGATCCCGAAATGCACCAGGGAGGTTTTGCTTTCTAGGTCATCCAATTTTTTAACTTTCTTGTGTGGACCAAATAGCTAATCCCTTTTTTGAAGCTCCTCGATCTGTTTGTATTTTCTTCAAATATTTTTTTTCTCTTGCCTGCTTTCTCCTCTTCCTGATTTCGTGGATAATTTTCCTGTTTCCCTAATTTTTATCTTTGTTCTTACAAAAATACTGGATTTGATGTAGGAGGTATAGTTGAAATACACTAAATGAGGGTAATTAAAACRRAACGTTTTCGGATTRGAAAATCCAACATCACTATTTCTGAAAAGC

>Dvv ABC-C D22628

GGCGGAATTGTAATTTTCAGTGAAAGTCACTAACGATACAGGTTTAATCTTCTTATATGTGATACGCCGAGTATAGTGAAATATGTGCATTTTCGTTTCGTTTTTAGGGTCCATCTATAGGATGTTTAGGATATTCTAATGGAATGTACGGAATATTCATAAAAAATAAATTGTTAGGCACTTGGGATTGTTGAAGTACCTAAAGTAGAAAATGGATTCGACTAAGAAGCATGTAAAAGTGTCACCGGAAGAGAGTGCTAGTCCGTTTTCAGTACTTTTTTATTGGTGGATTCTGCCTTTTTTGAAGTATGGATATCAAAATGATATTGGGATGAAAGATGTATATAATACCACACAAGCAGATCAATCAGGACCATTAGGGGATGAATTACAAGAAAATTGGGAGCGAGAAATTTTAAGTTATATTGACAAGCAGAAAAACAAACCTAGTTTAAAAAATGCAATATTTAGGACATTTTGGAAATCTTTTATCCTTTCCGGTGCTGCAATATTTGTACAATTTATAATTATTAAGACACTCCAGCCAGTAGTTTTGGCCAAATACATTAATTTCTTTGATACAAACAACAAACCCTACCTTGGTTGGATATGGGGATGTGGAGTGGTTTTGTTGGCATTGGCCAATGTGGTTCTATACCATAGCACAATGTTAGCAACCCAAAGGATTGGTATGAGAATTCGTACTGCGGTATCTTCGTTGACTTACAGAAAGCTTTTAAAATTAAACCACAAATCTCTAGGAGAAACTGCGGCAGGGCAACTGGTTAATCTTATGTCCAATGATGTTCAAAGATTCGATGTGTGTGCCGCTTCCATACATTTTATTTGGATCATGCCTATATACGCGGTACTTACATTCTATATCTTGTATATCTATGTGGGAATCATTGCTGCTGTGACTGGAATGGCGTTTATTAGTTTGGAATCAATTCCATTACAAGGAACAATATCCAGATGGCAGGGTGTATTGAGGTATAAAATTGCCCTAAGAACTGATAAGAGAATTAAACTGATGAGTGAACTAACATCTGGAATACAAGTAATCAAAATGTACGCATGGGAGAAACCCTTTGAAAAAATTGTAGAAATGTCAAGAAAATACGAAATAGACGTGATAGCCAAGACCTCATATTTGTATGGAATTTTATCAGCTACTAGTGTATTTACTGAACGACTGATCCTTTACGTAACTTTAATACCATTTGTGTTACTTGGACATAGATTGACAGGAGGCATTGCATTTTCCCTTGCTAACCTTTTTAATAACATACAACTTGTCATGGCGATTAATTTTCCAAGAGCACTATCATCTTATAATGAAGCAAACGTGTCTATAGCAAGGTTAGAAAAATTCCTTCTATTAGAAGAAGTTGAAGAAGAAACAGTAGTAAACGAAAAGCACGGTGATCATGTGGGATGTATTAATTTAAACAATGTTACTGCCAGTTGGTCCCCTAAATCGATCGTACCAACATTGATTGATATTGATCTACATTTAAGATGTGGAACCTTATGTTGTGTGGTGGGTAATGTAGGAAGTGGAAAAAGTAGTCTCTTGCAGTTATTTCTAAGAGAATTACCAATAACGTCAGGTTACATGAATATTGCAGGAAAGATCTCATATGCTTCTCAAGAGCCATGGTTGTTTGTATCAAACGTGAAAGATAATATTTTATTTGGAAAATCATTTAACAAAAAGAGGTATCAAGATGTTATAAAAGTGTGTTCGCTAGAAAGAGATTTAAAACAGTTACCGTACGGCGATAAAACATTGGTGGGAGAAAGAGGCAAATCGTTGAGTGGAGGACAAAAAGCAAGAATTAATCTAGCAAGAGCTGTCTACACAGAAGCTGATATTTATTTGTTTGACGACCCCCTTTCTGCCGTCGATACAAAAGTTGGAAAGCATCTATTTGATGAATGTATAACGAAATATTTAAATGGAAAAACAAGAATTTTGGTAACACACCAACTTCAATACATGAAGAAATCAGATCTGATCGTCATAATAAATAATGGCAAAATAGACAAAGTGGCAAAATTTCATGACCTAACTGAGCACGAGTTGAATCTCCTCCAACAAACCCCAGAAATTGATGATAAAGATAAAGAGAAAATGCCGACTATATTAGAGACGAAAATTCCAAAAGTTTCATCGACTGCAACACTTCAATCAGCATCTTCATTAGCAAGTTCTATACCAACTGAAGAACCAGGAGAAACAGGCGAATTAATAGAAAAAGGAGATTTATCGACTTCTCTTTATTGGGAATATTTCCGATCTGGGACTGGCATTGGTTTCCTGCTATTTACAGGTTTTATGTTTATTTTTTCACAAATCATAACAAATGCTTCAGATTTGTGGTTATCTCATTGGACTAATGTTGAAGCTAGAAGATATGTCTCAACTTTAAACTTATCTACTGAATTCATTTCTACAACGATTTCTTCTATAAACAACCATACCGTGACAGATATGCTCAAGTCTGTGGCTTTTACAAACACAAATCCTCAGGAAATTATCACAACTGCCGATCCTTCAGTTAGTGCTTCAAAGTTGTCTTCAAATATGTCATCACTAGAGTTGGAAAATTTTAAAAGTGAAGGATACTATATATGGGTATATTCAGCTTTGATCATAGCTGTAGTAATATTACAGATATGGAGATGCTTTTTGTACTATCAAGTGTGCATGAGCTCATCTAAAGCATTACACAATAAAATGTTTCATAATATACTCCAAGCACCGATGAGATTTTTTGATACCAATCCGTCAGGTAGAATACTAAATCGTTTCTCCAAAGATATGGGAGCTGTTGACGAATTACTACCAAGCTGCCAAATTGATGCGATTCAAATTTTGATGGTTTTTGTTGGTATATTGGTAATGGTTGTTATAGTAAATCCTTGGATGATCCTTACAACCATAATTATCGGTCCGGTTTTGTTTATTCTTCGAAAAATGTACTTAAAAACCGCTCAATCAGTGAAGAGACTTGAAGGAATCTCAAAAGCGCCTGTTTTCTCGCATATATCAGCATCACTGTTTGGAATTACCACTATTCGGGCATCCAACGCTGAGAAAATGGTCACTACAGAATTTGATATTTTGCAAGATCAACACTCCAGTACATGGTTTTTGTTTTTGGTCAGCGGAAGAGTATTTGGATTCTATCTGGATGTTATTTGCTGCATATTTTTGGCTATTGTTACAATTCAGTTTTTACTTTTTAGAGACGAGAATACGTTGAGTGGAAACGTAGGTTTAGCCATTTCCCACAGCTACATCTTAACTGGTATGGTACAAATGGGTATACGCCAGAGCGTGGAAGTAGCCAGTCATATGATAAGCGTCGAACGAATTTTACAGTATACCAAATTAGAAAAGGATGGTGTATTTGAATCACTGCCTGCTAAAAAACCTCCCCGAGACTGGCCTAATAAGGGGAAGATTATTTTTAAGAATACTTTCTTAAGATACGCGCTCAACATGACACCCTCCCTAAGAGATTTAAGTATAGACATTAAGTCTGGAGAAAAGGTTGGCATTGTAGGTAGAACTGGTGCCGGAAAATCCACCCTAATTGCTTCATTGTTCCGCTTAGCACCGGTCGATGGTGAAATTATTATCGATGATATAGAAACTGGAGGAATAGGACTACATGATTTGAGGACAAATATTTCTATTATTCCTCAAGATCCCGTTTTGTTTTCAGCTTCGGTCCGTTACAATTTAGATCCCTTCGAAAAGCATAGCGATGAAATTCTATGGAAAGCGTTGGAAAATGTGGAACTTAAAGGGGTTGTTACAGATCTAAATCAACCTGTTAGTGAAGGAGGTTCCAATTTTAGCGCAGGACAAAGACAACTCATATGTCTTGCAAGAGCCATAGTACGAAATAATAAAATTCTTGTCATGGATGAAGCTACAGCAAACGTGGATCCGCAAACTGATGCTCTGATCCAAAAAACAATTCGTGAGAGATTTAGAGATTGTACAGTACTAACTGTTGCACATCGATTGAACACTATTATGGATTCTGATCGAGTTCTTGTCATGGATGCCGGCCAAGCTGTAGAATTCGATCACGCCTACCGACTATTACAGAATTCAGATGGGTATTTTGCTAAGTTTCTGAAAGATGCTGGACCACCAATGGCAAATAAGTTAAGAGATATTGCTAAGGAGGATTATTATAAGAAACACACGTCGCCAGAAGTTATTGTCGATGACGATTAAGTCGAATTTATTAAAATCTTTTTTTATTGATACAAGC

>Dvv ABC-C D14070

CCTAGAAATAAAGAGATAAATATGCAACTGCTATAACAATAATTTATAAATACTTAATCAATCAAATACTACTATCGTTATTCAATCTTGTTGTTATAAGCAGCCTTGGCGATACTCTTTAACTGCTGAGTCATTCCAGCCCCAGTCTCCAAGACCATTCGATGGAAGTGTCCATCTGGTATTTCTAGCAGATTGTGGGGATGGTCGAATTCTATCATGCTTCCAAAGCTCATTACCAGTACCTTGTCTGAGTCCATTATGGTATTTAACCGGTGAGCGATGGTAAGCACAGTGCAATCTTTAAACCGTTTTCTGATTGTTGCTTGTATCAATGCATCCGTCCTAGGATCAACATTAGCCGTAGCTTCATCCAATACCAATATTTTGTTGTTTTTTAGAACTGCCCTGGCTAAACATACTAACTGTCTTTGACCAAGACTAAAGTTACCACCTCCTTCTGCAACTTGGAAGTCTAACGAATCAATACTTTCCCTTAAATCTACCTGTTCCAAAGCTTTCCAAATTTTCTCATCATCGAATTCATTAAATGGATCTAGATTATATCTTAATGTTGCTGAAAATAATACTGGTTCCTGAGGTATTATTGAGATTTTCTTCCTTAAATCAGTTAGTCCCAAATTTTTAGTGTCCACTCCATCTACGAAAATAGAGCCTTTAGTCGGAGCAAGACGAAAGAGAGCTGCAATAAGCGATGATTTTCCCGCACCTGTTCTGCCTACTATGCCAACCTTTTGCCCAGGTTTTATAACGAAGTTGAGATTGTTTAAAACTGGAGGATCATTCACCACATATATTAAAGATAAGTTTCTGAATTCTAGAAGACCTTCCTTGGGCCAGACACCCTTAGGTATTTTTTCTTTTGGTGTTTCAAATGGACCTTCCGTTTCTATTTTTGTATATTGCAGAGTCCGTTCTACACTGGTAAGTTGATTGATAAAGTCTGCTAACTGTCTCACTCCATACTGTGACAATCCAATTAGTACTGAGCCTTGGGAAACCGCTAGACCTACTAGACTACCATCTACATTGGTAAATTTAGTTAAAATGATAAAGCAGCCAATCACACATGCCATGAATATGATGGATACAATATCCAGCCACAATCCAAAGGAGCTCATGCATCCTATTGTTAGGTACCATGCGGACGTGTGGGCATCTTGATTTTCATCGAATTCATCTTTTAATATATTTTGTGCGTTAGCAGCTCTTATTGTTGTTATTCCATTTAGAGTTGAATTTACATAAGAAAATACTGGAGATTTTGTTATTCCTTCTATGTGTTTAAGAACTTTGGCAGTTGCTACATACCAATCTTTAAACTTTAAAAATATGTACCCCATTGGAACCAGTAGTATAAGTATATAATAATTTGAAACAGTGATGCTAACTAAAATTCCAGCCATTACCACAAAAATTTGACAGCTGTCTAACAAAACTTTTGGCAGCACTTCATCTATAGCACCCATGTCTTTAGAAAATCGGTTAAGAATCCTTCCGCTAGGATTTGTATCAAAAAATCTCATCGGAGCTTTAAGTAACGTGTGGAATATGCTTTTGTGCAAATTAGCAGATGACAACATGCAACATTTGTAGAAAAGAAAAGATCTGATTATCGTTAAAATAATAGCTAGTGCCATTAGAGCACTGTAAATATATATAGCGTAGTCTGTTTTAATGATCTTTTGTATTTGTCCATGAAGATCAATTTTGTCAAATATGTCATCAAAACTGATCTGCTTTAAAATTTGTGAGCCATTATTCTCAATATTGTAAACCAACCGATGGGTTGCATCGATTTTAGTTGTCTGATTTATTGGATATAATTCTACTTCATTAATATCCGCTGATTGCAATGAAATAACTGTACCGTTTGAATGACGTATTTGCTCTTGAGAAGTCCAGTAAGTTACCCATAGGTCAACCAAAACATTAATACCCTGAGCAATAACGAGCAGAATGGTTGTAAAAGTTACAAAACAAAGACTTTTGGTAGACAGAATGTACTGTTTAAAAGTTGTGCCAATATTAGTATTTCCTGACTCTTCTTCAATTACTTCAGGATCATCATACATACTTTCCGAAAAATCAGACAATGCCGATACTGCGGATGATTTTCTTCTACTACGCGCCGAAGCACGTCCACTTTTCCCTAATTTTCCTTCCGAGTCTGCGTCTTCTTCTTTGTCGTCTGTTTCATCAGCGGCAACTAATAGTTTGGTGAAATCTAGATTATATTCCATCAGTTCATTAAAGGTACCCTGGGCTTCAATTTTACCATCATTAACAACAACAATTAAATTTGCTTTCTTTAAGTACTGAATTTGATGGGTTACTAGGATCCTCGTCTTTCCTCTTAAGTGATTTACAATACAGTTTTCAAATAAATGTTTTCCGACATGAGTGTCCACAGCCGACAAAGGATCATCCAATAGATAAACATCAGCATTCCTGTAGATCGCTCTTGCCAAATTTATCCTTGCTCTTTGGCCTCCACTAAGAGATACACCCTTCTCTCCAACGATGGTTCTATCTTTATGGGGAAATTGTTCGAAATCTCTTTCGAGTGCACAAACTTTGACGACCTTATCGTACCAGGTTTTTTCGTATGGCTTTCCAAAGAGAATATTTTTTCGTACTGATGATTGGAACAGCCAAGGTTCTTGTGAACTGTATGATATTTCTCCTCCTATCGAAACTTTACCGGATTTAGTAGGGAGTTCTCCAAGAAGAAGCTGTAACAACGAACTTTTTCCAGCTCCAACAGGTCCCACAACAGCACATAACGTACCTGATGGTATCTGAAGTGAAATATCTTGCAAGGTACTTGTAGTAGGTGTCCATGAAGCGTTCACTTTGGAAAGTATAACCCCCGATTTTCCTAAATCGTCTATTTTAGATTCTTCTTTCTCTTCCATAACAAGAAACTCCTGCACTCTTTTGACTGAAACCAAAGCTTCTGCACCCTGGCTAACGGCCCAAGGCCAATATATAGCCATAGCCATTTGTAAGATGTTAAATGTTTGAGCCAAAGAGAAAACTATATCAGCGGTTATTAAGTTACCTAACAATATATAACAAACAATAGTAAAAAACAGAGCCATTCTATCCAAGAACACCATGCAACTGGAATATACCCCTCTAAGATAGGAAGCAGCTGTAATATCTTTGACTTCAGTGCCTCTAGCCAGTTTAATCACAGCCTCGAATGGTTTCTCCCAAGCATACATCTTTATAACCTGTATTCCTGATACCACTTCGCTCATGACCTTCACCCTTTTGTCTGTTTTCTTGGAGATTCTTTCTCGAAATTTACCTTGCAGTCTTGCCAGGTAACCTTGCACTGGTAAACTAACGACTGCCATAGCAACGACTCCTGCTACAGATGAAATTCCCACTTGTTGCCACATAATAAACAAGAGCACTGCTACCTGAATTGGCATAACCCATAGAGCATGTAGTGGTACTACGACTAAATCGAAACGCATAACATCGTTTGAAAGCAAATTTGCTATTTGTCCTGCAGCGGTTTGGCCTAACGCCTTCTGATTCAGCTTCGTGATCTTTCGATATACTAATGATGATATGGCCACCCTTATTTTCATTCCCAGTGCTTGCAGTCCAAACGCAGTGTGGTGATAAATAAATACAATTAAAACGTTTACTCCAATAAAAGCGGCACTATAAATGTAAATATCTGTGACTACATCATCGGAGTAGTTAATAAACAGTGCAACCAACTGGGCAATTAGAAGTGGTTTCAAAGACGTTAGTACCACATTTTGAATACACCAAAGAATTCCATACATTATCAGCTCAAGACGAAATGTTTTCCATAGCGCCCACATTAAACTTGGTTTTTTACTTTTTTCTTTGGCCAGCAAAACCTGTCTATCCCAGTGCTTTTCCAAGGCATCTCCCAGTCTTTTTGATTTGTCGGCATCTAAAGCTTCGTACAGATCTACCACTTCCAGAGTTTGTTTATTTCCTTTTTTAATTATGGGTACGAGCCATGCAAACAATAACTTCGACAAGAAGTTGGCCCGTCTTTTGGGATTCTCTGTCTTAAGTTTAAACACCACATCCATGATTACCAAAGAAATCGAAACAAACTCATTACACTAACTTATCACTATTTATTGGAGACTATGTAATACTCGGTTACGACCGT

>Dvv ABC-C T49513_c0_seq6

CTAGTTTAATCACATTCATTCTTCAATCAGCAAATTCTTATCAAACGTATTTTTCGCAACTGTTTTCAATTGCTCACACGTACCAGGACCAGTGTCCTCCACCATCTTAGAGAAGATACCGTTGGAATTCTGAAGTAAAAGATGTGGGTGGTCAAATTCTGCTGCTGTTCCCTGATCCATAACCAATACTTTGTCAGAATCCATGATGGTGTTCAGTCTATGAGCTACGGTTAGGACAGTACAATTTGCAAATTTGGTTCGTATGGTCTTCTGAATGAGAGCGTCAGTCTGAGGGTCGACGTTTGCTGTAGCTTCATCCAACATGAGGATCCTATTATTCCTTATGATAGCACGAGCTAAACAAATCAGTTGTCTTTGCCCGACACTGTAATTGGAACCTCTATCCATAACTCTATTTTCTAATCTGTTAATAATGTTATTGGGATCTTTCAGCTCCACTTCGTCTAATACTTTGTACAGTTGTTCGTCGGAGTATTCTTCAAATGGATCTAAGTTGTACCGAAGCGTACCCGAAAATAAAACAGGGTCTTGAGGAATGATGGAAATTTTCGATCGGAGGTCCCTCAAGTAAATGTCTCTCGTATCGATATCGTCAATTGCAATTTTGCCCTCTACGTTAGCTAGTCTAAATAGAGCAGCGATTAAAGATGATTTGCCGGCACCCGTTCTACCAACTATTCCAACTTTTTCCGTTGCCTTAACATTTAGAGACAAATCTTTTATAATCGTAGGACCACCTTCGAAGTATTGGAGCTTAACATTTTGAAAATCAATCTTTCCTGAACTGGGCCAATTCTTGGGAAGTGGCTTTGGATTTTTCGGTTGAGGTTCAGGTGGAAGTTGTTCGTATTCCAGAACTCTTTCGACCGACATCATCTGGTTAGATATGTCAGCTGTCATTCGCATAGCCATTTGAATCATTCCGGTTAAGGTGCTAGCTTGAGACACTGCCAGACCCACAGTTCCTCCCGTTAGTCCCATAGCTTTATCGAACGTAAGTATGCTGAATATTAGTACTGCTATAAATATATTTGAACACATACTTAAACTGATTCCAAAAGCTGCACCGCACGTGAAATTCATGAATCCGGCACTGATATGGTAATTTTGAATTTTATCGAATTCTGCCTGCAGAATTTTCTCTGTTCTTAGTGCCCTGATTGTTGGTAATCCTTGTAAAGTTGCACTTAGGTGAGTAAACACTGGACTTTTCAGTATTCCTTCCACTCTCTTTAAATTTTTCGAGGTTTTTAAATAAATATTTCTTAAGAAAAATGCAGCTATTGCCAAAATTAAGGCCATTGCAATTGCGTATGGATTGGCAACTCCTACTAGAATGAGAGATCCAAATATTTGCAAGATCATCTGACCTCCATCCAAAAGTACTCTTGGTATACTCTCATCAACACTTCCAATATCCTTAGCAAATCTGTTGAGCACTCTGCCGCTAGGATTAGTATCGAAAAATCTCATTTTAGCATAAATGACACCTTTGAACAAAATTTGGTGAAGATTGCGTGAACAAGACATAACCAAAGAATAAAACGAAAATGATCTTCCTAAAGTCATCAAAATTGAAATGGATATAAGTATGCCATAGATGTAAAGACATTCCTCAGTAGTCCAAGCAGGAAATTGGATCCAAGCTTGTGGTTCGAGTTTTACAGTGGTATTTAATGTATTTAATGCTGCATCACTTAAAGTAGTAGAAGGAATAGTTGAAGGAAAATCAGCTAGGACGACAGTTGAATTTATATTATTGGAAGCAATTGTGGTAGAAATATCCTCTAGTAAGGTAGATGAACTATTCGATTCTAATAAGATACTAGCACTGGTTCTTTCATCTTCAATATTTGTCCAGATACTTAAGAAATAATCTACCATGCTACTGGAAACTTGTACTAATACGTAAAGAGATAAAATGAGAAAGACGTTGAAACAATTGGCTCCAGTCCTAAAGTATCTTAAAAGTAAGGATCCTAAAACTTTGCCCTTCGATGATTCTTCTTGCATATCTCGCATCTCCAATTTTTTGGTGTTCTCTTCATCTTCACTATCTTCGTCACTATCTTCCATTAAACCTTCTACGATTGTCTTTTCACTTATGATACTTGTCATCGACGATTTTCTACTCTTTCTTGATTGTCTAGAAACTTGTCTGGCAAATTCAATTTGCTTAACTTTTTCCTCTTCTGTTATTTCTGGTTCTTTCGTTAAAAGTTGAGCGTATAAATTGTCACTATTAGCAAGATCATTAAATGAACCTTCGCCTTCGATTCTTCCATTGTTTAGAATAATTATGTGACTAGCATTTTTCAAGTAATGTACTTGATGTGTAACTAAAACTCTTGTCTTATTTTTTAAAAATCCATTAATACATTCATCGTAAAGTACTTTAGACACGTGTATATCCACAGCTGACAAGGGATCGTCCAACAAATATATATCTTTTTCTCTATACACAGCTCTAGCTAAATTGATTCTCGCTTTTTGGCCACCGCTCAAAGAAGCTCCTTTATCTCCAACAACAGTCATATCCCGATCTTTGAACTGCTCCAAATCTCTTTCCAGAGCACAAACACGAATGACTTCCTTGTATCGTTTTCTATCATAGTCAGCTCCAAACAAAATATTTTGCCGAATGGTAGAGGAAAATATCCAAGGTTCTTGTGAAGCGTAGGAGTAAGTTCCGTTGGCATTTATGGATCCTTTGTCTATATCCAGCTCACCTAATAACGTCTGAAGAAGTGAGCTTTTACCGGCACCAACTGGTCCAATAACACCTAGTAGCTTTCCCTTGTTGAGCTCTAAAGTAATATCATTAAGTACACAATCGCTCAATGAGGCATCCCATTTAGCATAGATGTTTTTGAGAGATAACATTGATTTTGAGTTTTCATTCGGATCTGAGCTTATCTTATTTTGATCGTACTTGTATTCATCATTTTCAAGGAATTTTTGAAGACGGGCGATTGACGTGAACATCTCAGCGATTTCTGCAATTCCTCTTGTCAAAATACCAGTTAAAGTCATAGATAGCATGTTGAAGTATGAAATAAGTACAAATACCTTCGCTGCCGTTATTTCTCCACCAGTTAATGTAATAGTAACAAGCGTAGCAAATAGAGCCAATTTGGTCGTAACCATTCCCAAAGTCATAAAAAGAGCCCTGATGTATGAATTTTTTTTAATAATCTTAATTTCTGCCTTTCTTGCCAATCCAATTACTTTTTGGAAAGGAATTTCCCAAGCATACATTTTGATAACTTGAATACCAAGAATGACTTCGTCCATCAGCCTGATTCTTTCATCTGTTCTCAGAGCAGTCATCTTCCTGAATTTTGTTGCCAGTTTAGCGGATGTTGCTTGTAAAGGAGACATTATAAGTATGACTAATATTCCAGCTATTCCTGGCAGTTGTGCTCTTTGATATAAAAAGTAAATAACGGGTATTGTGGAAACTGGTCCAATCCACAGCTGATGGATCATCATTGATGCGAAGTCAAACCTTGCGACGTCGTTGGAGAGGAGATTTACAACCTTTCCTGCAGGTGTGTCTCCCAGAGCAGTTCTACTGAGTCGAGTTGCTTTTCGGTATATTAAAGCACACACTGAAGATCGAACTTTCATCCCCGCATGGGCTGCATTAGTCATGTACTGATTCATTAAAATAGAAGAGGTCAAAGACATTATCACCATACCAGATCCATAAAAGTATGCTTCCAATTTTGATTTTTTAGATCCAGGAGTGTAATAGTCCAAAACTCCACCTAACATAGATGGTTGAAACGATCGTATACCTATATCAGATACTGCAGCTATAATTCCTATGAGGAGATATTCTAACCAGAAGGTTCTGACGACGGCTTTAAATAGACTAGGTTTTTCTTTTTTGTTTTGTTTTACCAGTTCTAATTCTCTATTCCAATTTTTTTCTAATCTATCTCCTAAATATTCGCTTCTATCACTTCTTAGTGTATTATATACATCCTCTACGCCTATCTGTTTTTTCATACCAGTTCTAAATGTTGGTATTGTAAACCCAAAAAACATAATTGATAAGATCCCAGCAGTTTCCCTGGGATTGGGATTGTTTTGCACCTCCGTCGAATCCATCGCTGATTACAAAATATATTTTATTTCACACAAAATTCTGTAGGTATAATAAATTTAATGTGTTAATACCTAAAATTCTTCTTTTTCATATTCAGTGGAGGACGAGCTTTGGTATGATATGATAAGCGTGTAACCACTAACAATACGACGACATAAATATACACACAGTTTATATATAAAACTTATATTTGATTTCATTTTATCTTCCTTTATCTGCATTCTTCGGTATGCAATGTATTGAAATAATGAAAATCATTAGACAGAGATAATTGTGATAAGATAATCATTCTCGTAGATGAATAGATCATTTTTTGTAAACTAAAAATTTGTATTTTTAAGCATTGCT

>Dvv ABC-C D18126+D19164

GCGTTGTGGAAATTTCTTATTTATCTTGAGGTGGTGAACGGTGCGCAAGGTTTCTTCGTAGAATGGATATAGGCTTTAAGTTAAAACATGAAAATCCAAAAAAGCAAGCAGGTCTAGTTAAAAAACTATTCTTTGGATGGCTAGTAAAACTAGTTAAACAAGGAACAAAGAAACAACTGGAAATATCAGATTTGTATGAGCCATTAGATAAGGATAATTCCAAAACTTTAGGCGATTGTTTAGAGAGACACTGGAAGAACGAAATTTTAAAAAGCCAAATTAAGAAGACGAGCCCAAGTCTTTTAAAAGCCATAGTAAAAGCGTTTTATTTCGAATTTTTATTATATGGAATAGCCTGGTTTGTTTTAAATGTTTTGCTAAGATGTTCCCAACCGATAATCTTATTTCATTTCATAGCTCTATTTTCTGGAGAAAACAGAGAGGAAAATCAAGGAGATATGTATATTTACGGCGGTCTCCTGATATTAGTTTCAGTCCTTTCCATATTTTTTATGCACCATTTACAAATTGGGTTAGCATCAATTGGCATGAGAGTCAGAGTTGCTTGTTCCTCACTGGTGTACCGAAAAATTACCAAACTCAGTCACCAAACCCTTGGACAAACTGCAGTGGGACAGGTGGTTAACTTAATGTCCAATGACGTTCATCGTTTTGATTTAGTGCTGTTACCATTGCACGCATTTTGGGCAATACCTTTTCAGTTTGTCATTTTATCTTATTTTATATGGCAACAAGTCCAAATAGCTTCTTTAGCTGGTTTGGTTTCTATGGTGATTATAAGCTTGCCACTACAAGGTTATCTGGGGAAATTAATGGGCACCCTTCGAGCCAATATAGCTAAGAAAACAGACAATAGAGTCAAACTGATGAGTGAAATCATATCAGGCATACAGGTTATAAAAATGTATGCTTGGGAAAAGCCGTTTGAGAAAGTAATAGAAATAGCAAGGAAAAATGAGATAAGATGCGTTACACTGACTTCTTATCTGCGAGGAATATTCGCCAGTTGTATGGTCTTTCTGGAGAGAATGTCTTTGTGCTTCACCTTAATTTGTTACGTTTTGTTGGGTAATAACATAACAGCTGAGAAAGTATTTTCTTTAGCTCAGGCTTTCAACATTCTGCAATTATCCATGGCGATTTGGTACCCTTTAGCAGTTAGTCATGGAGCGGAGGCGTTAATATCAATTAAACGACTTAAAGCGTTTCTTACGTTAGAAGAAAAGGAGGTTAGTCGTATCAAAGGACTGTCAACACCGGGCGTTGTTATGTCAAATGTAAGTAGTTCGTGGTGCGATGCTGGCGAAACGTTGCAAGATATTTCATTAAATATTCCACCAGGATTTTTGTGTGTAGTGATTGGACCACTTGGTGCTGGAAAAAGTTCTTTATTACAGTTGCTGTTAGGAGAACTAGCTATCAAAACTGGAACGGTTCTAATGGGAGGAGAAATATCATATTGTTCCCAAGAACCTTGGCTTTTCCAGTCAACAATACGCAATAACATTCTCTTCGGCAGACCTTTCGACAAACAGCTGTATGAAAAAGTCGTCAAAGTATGCGCATTAGAACGAGACTTCCAACAATTTCCAGAGCGGGATGAAACTGTCGTTGGAGAAAGAGGTGTTTCACTGAGCGGAGGTCAAAGAGCAAGAATTAATCTTGCTAGAGCTATTTATAGGCAAGCTGATGTTTATTTGTTAGATGATCCGTTATCAGCTGTAGATACACATGTTGGAAAACATTTGTTTAATCAATGTATTGTGAAGCACTTACGAGGAAAAACAAGAATATTGGTGACACATCAATTACAGTATTTGAAGAAAGCCAACCTCATAGTTGTTCTTAATGAAGGTAAAATTGAAACAGTAGGAACATTTGAACAGTTATCGAGAAGCAAGTTGGATTTTGCCAAAATAATAGTGGACAGTGTAGAACCAGGCGACAAACACGAAGAGACAACCGAATCATCGGATCTTACAAATACTGTGTCAAATCCAAGAAAAGCTAGTGTAACATCCACCAAAAGTGATTTATCAGAAAGCTTGGAATATTTTGAAGAAAATAACTTCAGCGACGAAGTAATTGAACATGATCCTCAGCATACGGCGTGGAAAGAGTATTTTCAAGCAACCAGACGTACTGGACTTTTAATAGTAGTTTTCACCATGTTGGTCCTTGCTCAAACAATTTGTTCTGGAACTGATTTATGGGTGGCTTTTTGGACAAATCAAGAAGTAATCAGGCATAGTACTTCAAGACCTTTAGAAAGTGATGTATCTTCAGATGAACCTATTCTGTATGAAGCTACCCCGTTTGATAGCAGCCTGAATTTAACTCACAACTACGAATACAAAGTAGAAGAACCAGTTGTTACAAGCGATGGCATTTTCGATTATGTTTATATAAACAACCACGTGTACCATTTGGTTAAAACAGGCTACGCCATTGGTTTTTATGGATTTTTGATAGTAGGAGTTATAATTTTAACCTTATTCAGATCGATGATGTTCGTTAAAGTTTGTATGATAGCTTCCGTTAATATACACTCGAAGATGTTTAGTACGTTATTAAGAGCACCCATGAGGTTTTTTGACACCAATGCAAGTGGGAGGATTCTGAATAGATTCTCCAAAGATATGGGATCCATAGATGAAATATTACCCAGAGTACTACTAGAATCAGTGCAGATTTTTTTGGTTCTGATTGGTATATTGGTGAATGTATCGATTTCAAGTCCATACGCCATTATAGCTATGTTACTTTTGGGAATTTGCTTCCTAAAATTAAGATCTTGTTACCTATCCATAGCGATGTCGTTGAAACATATAGAAGGAAAAGTCAAATCGCCGATGTTTTCTCACGTCAACTCTTCATTGCATGGAATGGCAACCATTAGAGCTTCAAATACAGAAGATATACTTATTAAGGAGTTTGACGAGCACCAAAATGTTCACACGTCGGCCTGGTATTTAACAATAGTATGCATTTCAGCTTTTGGTCTTTGGATGGATATTGTATGCATCATATTTTTGACTTGTGTCATTTTAACCTTCATCTTTTTGCAAAACTTTTTTAAAGTAAATAGCAGCTTGGTAGGCTTAGCAATTTCTCAAAGTATGACTCTAACTGGCATGTTACAATATGGCATGAAGCAAACAGCCGAGGTGATCAATCAACTAACAGCAGTGGAGAGAGTGTTGCAGTATACTCATATTAACACTGAAGGACCTTTTGAATCACCGATAGAAACTCGTCCTGTCGAACCTTGGCCTAAATTTGGACGCGTGGACTATAACCATGTTTATTTAAAGTATTCTGAAGATGATCCTCCAGTATTGAGAAACGTTCAGTTTACAATTTTACCTGGACAAAAGGTTGGTATTGTTGGACGCACCGGCGCTGGAAAATCGTCTTTGGTTGCAGCTCTGTTCCGTTTGGTAAATTTCCAAGGTACCATATCAATAGACGGAATAGATACAAAACGTATGGGACTTACATATCTAAGGAAGAAAATATCAATAATTCCACAGGAACCAGTATTGTTCTCTGCTACTCTCAGGAATAATCTAGATCCTTTCGAAGAATTCACTGACGAACAAATATGGAAGGCACTGGAACAGGTTGAATTAAAGGACATATCACATTCATTAGATTTGATGATATGTGAAGGAGGAAGTAATTTTAGTTTGGGCCAGAGACAATTAATATGTTTGGCTAGGGCAGTTTTAAGAAACAATAGAATTTTGGTGTTAGATGAGGCAACAGCTAATGTGGACCACAGGACTGATTCTCTAATCCAAACAACTATTCGTAACAAATTTAAAGACTATACTGTAATAACTATCGCTCATAGACTAAATACTATTATGGATTATGACAAAGTTATCGTAATGAGTTATGGAAGAGTGGTAGAATTTGGTCATCCGCATCAATTGTTACAACTACCAGATGGACACTTCCACAGAATGGTTCTGGAAACGGGACCTGTGATGTCTTTGCAACTTAAAGATGTGGCCATGGTGGCTTATTCAAACCTCCTGAAACAGGAAGCAGCTTCTATCCGTTGAG

>Dvv ABC-C C222633_3.0+T22098_c0_seq1*

CTCCTCTGTCCAACACCATCACTTTATCACAGCCCAGCACAGAATGCAATCTGTGCGCTATGGTTAATATTGTGCAGTCAAAAAAGTTTTCCTTAATAATCTTATGTAAAAGTTTATCAGTTTCGTGATCCATGTTGGCAGTGGCTTCATCCATGACTACAATCTTGTTCTTCCGAAGCAAAGCTCTGGCCAAGCAGAGAAGCTGCTTTTGGCCAGAACTGAAAGTTAGTAAGTTACTGTTCGTTGGTTGTTCTAAGTTTGTGATTGAATCGTTGATTCCAACTGTTTGTAAAGCATTCCAGAGGTCTTTATCTTCAAATTCATTCAGAGGATCCAAATTTGATCGAATAGTTCCGGAAAATATCGTGGGATCTTGGGGGATAACAACAATATGTTTTCTTAAGAAATCTAGAGACAAAGTTTTAATATCAACTCCATCAATAAGGATCTTGCCTTGAACCTCATAAAGTCTAAAAAGCGTAGATATTATTGAAGATTTTCCTGCACCTGTCCTTCCTACTATACCAATCTTCTCTGTAGGTTTAATTTCAAAATTTAGGTCTTTTAAAACATAATGAGTATTGCCTTCATAAGACAGGTACACGTTTTGATATGATATGCTTCCAAGTGAAGGCCAGTTGTTGATTTCAGCTCCTTCTTTATTCTCTGTTTTGATGTTGGTGTACTCCAGGATCCTTTCCAAAGACGTCATGTAATTTTCTAGCTCAGTAAACTGCCGTACGCACCACGTTATGCTAATGGAAAGGGCGGAGACTTGAGTGATAGTGAGACCAATGTCACCAGCTGTATTATCTTTGTTAAAGAATAAAAATTTGATCACTACAAAGACGATGAATAATGAACATATCAAGTCAATAAAAAATCCAAAAGCTCTAGCAGAACACATGGATGTAAAACTGGCTGAAGTATATAAATCTAGGTGTCTATTATATTCTTCTATTAAAATTCCTTGCACTTTTGAAGCTCTCACCGTGGTGACTCCTTCTAAGGTTGCGTTGAGGTGTCCAATCATAGGACTTCGACTTGCTGCCTCCAGACGTTTTAAGCTCCTTCCTGCAGGTAAGTACATTTTTCTCATTAGAAAAGCCAACGCAAAAATCAAAGCGATAATGAAAGCACCAGAAGGGTTGACTAATGTAACCAAGAAAACTATTCCACCGACGTTAAATATCGTCCTGCAGCATTGATCCAAAACAGCAGAAAGATACTCATCGACACTCAGCATATCTTGTGATAACCGGTTCAATATATTTCCTATCAAGTGCGTATCGAAAAACATCATCACTGAGTTGATGATCTTCTTGATCATCGCCTTGTGTAAATTAACAGAGGCCCTCCTGCAAAACTCCAACAATGCGTAAGTAGTGAGCAGTTCCATTCCTGTACCCAACATAACAAATACCGAGTACAACCTAAACGTGTCTTGTTCGATGGATTTGGCCAATGACAATGAGGGAAATAAGGTATCATTAGAAAGCGGATTGGTGGAAATGTTTTTCTCGATGTTTAGCACAATTTGCTTTTTGTCCACCCACTTGGTTAGTAGCCTCTCTGCATAACTTACTGCAAACTGAGTGGATCCTCTCATAGAAATATTAAAAAGAACGAATAAGAATCCTCCTCCGTATATAAAGTACTGTTTGTAAACTCTCCAATCCACAGATCCTTGTTTCTTTACTTCACTGTAGATCTTTTTCGTCGACTTTTCGTCTTTTTCAAGCAATTTTATATCATCTGTCACTTCGTTATCGAAATCTGCAGAGTTATCTTCTTTAATTTGTTGTTTTTCTTGTTTTTGTAAGTCCACACCATGATCTACAACTTCTTTTGCTTCTCTTATAATCTGCTCGCTTGGCTTACCTACGTCTTTTACTGTACCATCCTCCAATATTATCACATAATCAGCTTGTTGTATATGACTAGGATTTTGACTAACCAAAACACACGATTTGTTTTTTAAAAACTTCTTAATGCATTCGTTGAATATATAAACTTGAACTGATGCATCTAATGCAGTGAGACAGTCATCCAAAAGATAGATATCACTAGACCTGTAGATAGCTCTCGCTAAATTGATTCTTGCTTGCTGACCTTTGCTTAGGTTGAGTCCTCTATCGGTTAATATAGTTTCGTCTCCGTGTTCAAAAAGACTTAGGTCAAATGTTAGGGCACAGGCTGTTATAACTTCATTATACCTTTTGGCTTGATAAGGTTCTCCAAAAAGTATATTCTGCCTTATCGTCGAAGGAAACAACCACGGATCTTGTGACGCATATGAAATCCTACCCTGTGTGAGTACTGATCCGCTTTCTAATGGTATATCCTTTAAAATAGTTTTAAATAATGTACTTTTACCACATCCAATAGGACCTGTAATCACAGTTAGACCAGGAGATATTTGAAATGAAACACCTTTTAAAATTTCATGGCCTCTGACTTTAACAGTAGCTTCCTTCAATTTAATAAAAGGTGGAGATTTAGGGTCTTCTTGTGTAATTTCTTCGACAAGTTCCTCAGATTTCAAGGCCTTGACTAATCTCTTGTACGAAGATACAAACTCACTGACGTTTCCCATACCCATAGGCATCACAATAGCTAAAAAATGTCTGAGATCTCTGAAGTTAGATAGAATATAGAATATTATGGTGGTGTCTGTAGATACGCCTGCCCATATGCAAGCCATGATTAGCATGTAAAAGCCAAAATTTAAAAACATTACTCCTGTGATTATAATAACTCTTCTGCAGTAGAATCCCAGAACAAGTTTTCTCATTTCCTCTTTTCTTGCTTTAGTAACTTTTTGGGTAAAAAACAGTTCCCAGGTGTACATTTTTATTATTCTTATCGTAGACAATATTTCCTGGCTAAGTTGTAGCCTTTCGTCAGACTTTTGGCACGTTTGCAGCCTAAACTTCGTTACCCACGTACTGATATAAACTTGAAAAGGAAAAACTGATATTAAAATTCCAATGCCTACGAATGATACGACTCCAACTTTATTGAACAGCAAATAGCAAATGACCAAAACTTGCGTTATAGATACCCAAGCATCGTTTACACTAAAAATTACTGTCACAAATGCTTGGACGTCCCTGGTGATCAGCGTAACTATATTTCCCAGATTTGTGTCGGTTGTACCAGCTGATGAAAGTTTTAGAGCTTTCCTATATAAAAGGGATGAAAACGCAGTTCTCATCTCAATGGCAAACATCTGAATCCATATCATATAATTGTGGTTGTAAAATAAGGCCACAAAGTGGATGACCAAAATGCCGCATCCATAAAAATAGGCGTCATTCCTCGTTAAAACGGTTTGGCCGGGAGCAAAGTAGGCAACGAAATGGGCGATCACATATGGGCGTATAATACTATTAATAGTATTAAAAACGAAATCAATGAAACCTCTAAACAAATATCTTTTGCCGTAACATCTCCACATAACCCGAGAAACCGATGGCTTCTTTGAGCGATTTTTTTCACTTAACCATTCCCGTTCTAATAGATCACCACATTTTTTGGCTCGGTGTTGATTTAATACTTCATAGATATCGTCTTCATGGAGGTCGAGCTTAAAAGCTTTTTTGAATAAACTTCCTACATATGCGAAAGTAAGTAGAGAAAATATATTAGCGGTCTCCCTTGGATTGGTTTTTTTCTGTTCTTTTCTGCAGTGATCCATTTTGAAGTTGCACAAACAATGTAACAATACGAACAATACGAAAT

>Dvv ABC-C S21020Locus_43217_0+T47823_c0_seq1*

CTTAAATGCGCAGCCATATTTTTACCGGTATTCCTAACTAAGCCATAAAATATAGTCTTTTTATTTTGTAATAACGTATATGGATGATCGAACTCAACAGCTTCTCCGGCGTTCATCACTAACACTTTGTTAGAGTCCATAACCGTGTTTAGTCTGTGAGCGATCGTCAGCACTGTGCATTCGGCGAACTTTTCCCGGATAGTTTTTTGGATAATAGCGTCCGTCATCGGATCAACGTTAGCGGTCGCTTCATCTAGAACTAAGATTTTGTTGTTTCTGATGATAGCTCTCGCTAAGCAGAGTAGCTGCCGTTGACCTACACCGAAGTTGGATCCACCCTCCGCCATCTTGCCGTATAGATTGTGTCTCATCTCTTCCACTGCATGTTTCAATTCCACCTCGTCTAAGGCGGCCCAAAGAACCTTATCACTGTAATCATCGAATGGATCCAAATTTTTCCTAAGCGTCCCCGAGAAAAGCACAGGTTCTTGTGGAATAATAGATATCTTAGACCTCAACACCTTCAGCTCAACAGTATTTATATCGACATTATCTATGATTAAGCTTCCTTCATTTATTGCCAGTCTGAATAACGCTTGGATCAAAGATGACTTCCCAGCACCCGTTCTTCCAACGATGCCAATTTTTTCTCTCGGTTTTATCTCGAAATTAAGATTTTTAAGTACACAAGGAGCTTCCAAGGAGTACCTCAAACTCATATTTTTGAACGTTAGGCTTCCTGAACTTGGCCAATCTGCTGGAGGTTGTTTTCTCTCGTTATCTGATTCTATGGGTAGGTCGGTATATTCTTGTGTCCTTTCTACTGAAGTCATTTGGTTTTCTAGTTCGCTCCATTCTCGCATTACCCATTGGAACATTCCTGATAATGTAATTGCCTGAGTCAGTGAAAGACCGACATTTCCTCCCAATGAATCACTTTGGGAAAAAACTATACTGATGACGACCAGACTAATATAAAAGGCACAGTGCAAGTCCAACCAGAATCCAAAGCCCCTACTGGCAGTTAGGAACATGAAATATGAAGAGGTGTAGGCATCTTGGAAATTATCAAACTCTTGGGTGAGAATTTGCTCTGCTCCAAATGCTCTTATTGTTGGAAGTCCTTGTAGGGAGGCTGCTAAATGGGTAAAAATTGGACTTCGAGTGACAGCTTCGATCCGTTTAATATTTCTACTGGATACAAGAAATATTTGCTTAATTTTGTAAAAAATACAAACAATAACAGCCGTGGGTATCAATATCCAAGGATTCAAAAGACTAATAACTATTGTTGTAGCCACTACAGTAAGGCCGACCCATGTTGAGTCTGCAGTCGCTAGCGGTAGCAATTCATCTAAAATACCAATATCCTTGGAAAATCTGTTTAAAATCCTACCAGATGGATTACTATTAAAGAACAGCATCGGTGAGCTAATAATTTTTCTAAACATTGCGTTATGGACATTCGTGGAAGCAGTCAAAAAATAGCTAAAAGATGCGAGGGATCTAGCAACAGCCAGAATTACTGTTGCGATCACAAGGAAGCTGTATATATGCAACGGACTAATTTGGTCCATCACGTGGGGCCAGTAACTTTCAGTGGATTGGTCCTTATATTTCAATATTGTATCTAAGTTTGAGGAATTTGAGGTTTCATTTATGAAAACCGCTTGTTTTCTTCTCCACTGTTCTATATTTACCCATAACGTTAGAAATATGTCGGATAAAGAAACCAAAACTTGTGTTCCAACGAAACTTAGTGCCAATATCAAAGTTTTGAATAAATTTCCTCCCGCCTTTAGATAATTTCCATAAACTCTCCAAGACACTCCACCAGATCCAATCGTTTCCTTTTGTTGGTCAATAGATATGTAATCTTCTTCTTCGTAGTTGATCGAACTAATAGATTCAGATTTTGACAGTTTCCTGTCATGCTCCAATTGATCCATTTCAGTTGCGGTGGCTAATAATTTCGTAAAGGCATTATCACCATTTTTCAAGTTTTCGAAAGTACCAGAAGCTCTAATTCGACCATCATCGATTAGGTAGATAAACTTTGCTCTATACAAATATTGTAACTGATGTGTTACTAATACCACACACTTTTTCCTGAGATAGTTGCAAATGCATCTGTTAAATATTTGTTTGCCAACTTGTGTATCAACTGCAGATAAGGGATCATCAAGAAGATATATGTCTGCTTCCTTGTAAATAGCTCTGGCTAGATTGATTCTAGCTCTTTGTCCACCACTTAAAGACGTACCTCTTTCCCCAATAAGAGTTCTATCACCATGTGGAAACAATTTTAGGTCTTTTTCTAGAGCACACACTCTTAACACTTCATAATATTTATTTTCATTGTAAGTTTCTCCAAAAAGGATATTTTGGCGAACGCTGGAAGCGAATACCCAAGGTTCTTGTGAAGCAAAGGAAACAGTTCCAGTTACTTTGACTGTGCCTTCTAATGGTTTTAGCTCTTTCAAAATAACTTGTAGAAGGGTTGATTTTCCTGCACCCACTGGACCTACTAAGGCTACAAGTTGATTTGATCTGGCTTCAAAGTTGATTTGTTCTATTGTGTTTTCTGGATGTTTTTTGATCCATTTTACAGCTGCATTTTTTATTTTAATACCTACAGGCCGTACTTCGATGACATCTGGCTTGTTGTTTTGTAAAATCTCGTTGTTGTTGATTTGTTTGTGCATTGGTGTATATTTTACATCTAGTTCTTCAAATAATAAGAACTTCTGTATCCTGGATATGGATACTAATGTCTCAGCAAACTGGGTCATAGCTTGAGGAAACTGCAAAGTGATTATGGCTCTCATGTATGTATAATATGACGTTACTGTAAAGGCATATGATGCTGTGAGAACGTTTCCTGATAATATGTATGTTAAAATGCATACAAGAATAGCCGCTCGTGATAGTGCGATGTTGAAGGTTATAGTTAGAGCCTTTATTATTGAGGTATGTCTTATTTGGTCAATTTCCTTCTTTCTGATGAATTCAATCAATTTGGCAAATGGTTTTTCCCATGTGTACATTTTAATAACCTGAATTCCTGAGATAATTTCGTTCATCAGTCTTATTCTTTCGTCAGTCTTTAAAGCTGTTTTTAATCTAAATTGCGAAGTCAATTTCGCCATATAAACTTGTGCAGGAACAAAGCACAATAAGAACATACTTCCAGACAAACCAGTTATCCCGGTTTCCTTATAAAGCAAAATAGCACAAGCGATTCCAACACATGGTGATAACCACAAATAATGAATATGTTGTCCACTGTAGTCGAATCTTCCCACATCATTGGACAACAAATTGACCATCTGACCAACGGTGGTTTGCGACAAAGCTGTTTTGCTTAGTTTTAGGGCTTTTCTGTATATTAGAGCACATGCCGCTACTCTTATTTTCATTCCTAAAACCATAACTTTTAACTGATAGTGGTGTATGCTCACTACTTGAGCAAATGTGACAATAATTAAAGTAATGGCACAAACCACAATATTATCGCTTTTGGCGCCATCTTGGAAGTACGAGACCAGCTTTGATATCAAGAATGGTTGAGCTATCCGCAATAGCTCGAACCAAGCGTTGAACACTGCATATATTGCTAACTCCCATTTGAACACTGAACCTATAGCCAGTAGAAGAGAAGGCCGTTTCTTTTTCAGTTGTTTCTTCCATGCTTGTTCTAGTTTCGTACCAAGATATAAAGATTTTTGTTTTTCGACAGTGACGTACATATCGTCTTCGGTGACTTCATTTTTGAGTCCTCTAAAGAGGTAGACTGGTAACCAACAAAAAAATAATTTTGACACGCAGTTTGTTGTTTCGATTGGATGTCGTTTTCTTTTCTTCTGTTCTAAAAATGACACTTCCTCCATCACTTGTTACACAATCTTTTAAGTATTTACAATTATTTCCAGTACGAATAGTCACTAAAGTACACAACACAAAGTATTAACTCACTTTGTTAAAAAATGTCACAGTTAACTTTCACACTAACGATTACTCTTTTATTATATATATGTACGTTTTAACATTTTAAGATAAGTAATTCCACAAAAAATACGAAGTTCAAATAACTGCCACTTATCAATTTCGTTTTAATATTGTAAATAGAAGAGTGCGGACGAGATTTCTATTTATTTAATGAAATATTGTATTTTGCAAGATGTAGGCTATCACTCTTTCATTGTTCTAGTGTCGTGTATCCCGTCGTTACACGAGGATAGTGGAAAAATTTGTTTATTTTTTATTTATCTAAATTTTAGTGTACTTTGTAGTGT

>Dvv ABC-C T51687_c0_seq1

CAGTCAAAACGCGAGGCACGTGGTGCGAAAACAGTCATCTCGTATGTGACCGAATATTGTCTGTATAAAACCCTATATTTTATATTTCCACCTGAAACTAACAATCCTAACTACAGTCCACACCCTCTGAAAGTATATTCATACTCTCACATATGTACAAGTTATCGTACAGCAAAGAGTGATCAAATTCTTACGAGCCGAATGATAAAATATAAATTTTTTGGGACTGTCTTAACCTTCCATAGCTAACACCCGGTCGGTGAGACCGTTGATCTCTTTAGTACCTTTAAGGCCAAAAGATATCCGTATCACATATACAGTGATAGAGCATTTACGCGTTTTGAGTTATCAGTGTTTTGGTCTGAGAGACCGGTGTTAGAGTCTGCGTTACTTATATTGAAATATGTATAAAAAATGTAAGTGTAAAACAGGATTTAGCTGCCATAAATGTAAAAATTCGTAAAAATTCAGTTTCACCAAAGACTTTTTTAATTTCCTCAGATCCACGTTACTTCCTACGCCTTTAGAAATATTTAGTTCATTTTCTTGATTTTGAAGACCTTCAGAGAATCTATATTTTTCTGTAAAAATACTGGTAAATGTCAGTTTTGAAAATACATGCCATAATTTTGACATGTCGGTCTCTGAGACCCCTGTTATCTTCTATGTGTAATTAGTTAAGACCATTTAGATAAGATGCTCATCCACTGTAACTCAAATTGGCGACAAAAATTAATCTTACTTTTGTTACTGTCAGTAACCATGTTATTGGGCCAGTAGAAAGATTGTGACACTTACCTTTTAAATACATACAACTTAAACTTGACAATGTCAGATGATCGATGACAAATGACTGATGAGAAATGATCGATGACAGATGACTGATTACAGGTGACGTTTAGTGATCTGCCACCTGTCATTTTAGCCAATTCAAGTGACCCTAGACTACGATTAATTAAAAAACTAGCACGATTAGTCAACATTGGCAACTTAATTGATAAGTTAAGACGTTCTATTGCTTTTTTGGAAAATTTGCTTTTAAGCTAAACCGGCGTCTCTCGACATACTGGCAAATATTGAATTCTCATTGGCTAGCAGTCGCGCAGGGCTGTCGAATTCTGCTATCCTGCCTTTATCTAGCACGATGACTCTGTCTGAGTCCATGATCGTGTTTAATCTATGTGCGATGGTAAGTACTGTGCATTCTTTGAACTCCGTTCTGATTGTCTTTTGAATGAGATCGTCCGTTTCTAAATCTACTGCTGCTGTGGCTTCGTCCAAGATGAGAATTTTCGTTTTTCGGAGTAGAGCTCTGGCGAGGCAGATTAGCTGTCTTTGGCCGACTGACAGATTTTCACCGCCTTCGGTGACTTCGTGATTAAGGCCAGCTGGTAGTCCTTTCACGAATTCTTTAAGATGCGCATGTTCCAAGGACCTCCATACGTCTTCATCGCTGTGTTTGTTGAATGGGTCCAAATTGATACGTAGGGTTCCAGAAAACAGTACGGCATCTTGAGGGATAATAGTAAGTCTAGAACGAAGAGTGTGTAAACCAAGTTCGTCGATGCGGATTCCATCGATTAGGATGTTTCCTTGCGCAGCTTCGATTATTCTGAATAAAGCCAACGTTAGACTCGATTTTCCTGCTCCAGTTCTTCCTACAATACCAACCTTCTCTCCACCCTTGATTTCGAAGTCCAGATCGTGCAACACGAGATCCAAACCGGGTCTATATCTGACCGAATAGTTTTTGAATTGGACTGTACCTTCTTCCGGCCAGGCTGGGGACGTCTGCTTGCTAGGGATTTCCCAAGCGGCTTCCTGCGGGGCTTCTCCGTACTCTTTAATTCTTTCGACGGCAACGATGTTGGTTTCCACATCAGACGTCATTCTGACCAGCCAGTTGAGTGTTTGTGTAATCTGTAGCGAATAGGTCACAGAAAGACCGACCAATCCTGGCGCCTGATCTTTCTTGAGAACAGCGAACAGAGCAGCGAAAAATATAATTAAGTTTCCAATCATTTCGAGCCTGACTGCCAACCATCTATTCGATATAATTCCGGGATAATAACATATTTGATTCATATCCACTTTGTTTTCCGATTCCACAATAAATCTTTCCTGTTGATTGTACGCTCTGATAGCATGTGCTCCAGAGACACTTTCTCCAAAGTGCGAATAAATCGGGGAACGTGACACAGATTCTAACCTCTTTACTTGTCTAGAGGTAGCCACGTAGAACCTTTGCATGAAGTAGTAAATGAAACATAAGGGGATTATCACTAAAATAAACACTGGTGTGGAATAGCTGATTACAGCTAGAGTACCTACGACCGAATAGAAACAAGTGATCCAGCCTCTCATTGTCATGGGCAACACGTTGTCCAACGTGTCAATATCTTTGCTGAATCTATTCAGAATCCTTCCCAAAGGAGTCACGTCGAAGAATGTAGTACATGGAGCTTTTAGTACGTTACCGAGTATCAAGTTATGCAAAGCTCTTGCTGAGTTTAAAGATCCCACGTACAGCGCCAACGAGGCAAACAATATCGTTACAACTTGTCCAAGACCTAGAGCACCATACACCCCGAGATACATATCTCGTCTTGCAGTGTCAACGGTATCGTTGACGACGATCTTTTGATCATCTGACCAAAGCCCCAACCAAACGTTGGAGCCGACACTGAAACCTTGGTACACTAAATTAAAAATCAAGGTGGCCAACATAAACACCAATCCGATAGACTTCAAGTAATGCTTGTAGACGGCCCAGCTGACGTTGCCCGTTTCTGCTTTCTCGGTTTCTATTAGCTTCTCGCCTTTCTTTGGACCTTTCTTACCGTCATCTATGGATAGTTTGTGGTTGCTTTTGTCCACGCTGTTGAGGCGTTGGATAGATCCGTTGCCGATGTGGTCGGAGCCTGTTTCTGATTGAGATTCTGAGACGCGAGACCTGTGCCTGACTAGTTGTCTTGCTACTTCTTGAGATAATGGTGTGTCAGCGAGTTGGTCTTTGAGTTCATCGAGTTCTGCTTCGGTTTCGGCTTCTTCTGTAATATGTTGGAGAAGGAATTCGGCGAATGCTCCTTTTCTATCCAGGAGTTCCTGATATGTTCCCGTCTCGGAAATTCTACCGTCTTTCAGAACGATGATTTTTTCAGTTTGTGGCAAATACGTGATTCCGTGTGTGACGAGGATTTTGGTTTTGCTTTTTAGCAATCCATGGGGACCGATCACCTTTTCGAATATGTGTTTTCCAACGTGACTATCTACAGCACTCAACGGATCATCCAAAAAGTATATATCAGCATTCGAGTAGACTGCTCTGGCGACGCTAACACGTTGTTTTTGACCTCCAGATAAATTGATACCTTTTTCGCCGATTTCCGTCTGATCACCAGCCGGCAACATCTCCAGATCTGGATTCAGGGCACAAGCTTCCACCACTGCGTCATACAGCGTTTTGTCGTAGGGTTTTCCGAAGAGGATGTTATCTCGAAGGGTGGCGTTCTGGATCCAAGCTTGCTGGGAGACGTAAGCGACTTTTCCATATGAGTTCACACGGCCTGAGAGTTTGTCCATTTCTCCTAGGAACGCTGAGATCAAACTGGATTTTCCTGAACCGACTGTTCCGACGATGGCCGTTAGAGTTTTTTTCTTGATGTTAATGTTGATGTCCTTCAAGATTGGTCCTTCGCCCCAGGAGAATGTGCCGTTTTCGATCGTAAGTGGCTCTTCAGAGGGTTCGTGCAAAACATTATCAGGATCAAGTTCTTCAGCATTCATGAATTTGTTTATTCTTTTAACTGACACCCAAGTTTGTACTAAATTTGACAACATCATGGGCAACATGCTGAGTGGAAATCGTATGATATTGAATAGAGAGATAGAAACATAGGCTTTATTTGCGTCTAATACATGTTTTTCATCCACCAATACGTATGTAGCGAAAGTGACTAGAGAGACCAGGAATGGAGCACAAGACCAGATGAACGACGTACCAGCATTCAAATAAGCAGCTTGTTTCAACACTTTAATTTCTTTATTTCTGATTTTCAATACTTGATCCTCAAAACTGTGTTCCCAAGCGTACAGCTTCAAAACTTTAATCCCGTTCAAAATTTCGTTCATCAGCTTGACACGTTCATCCTTGTTCTTCATCTGTTTCACTTGTAGTTTTTTGATCTTGTTAGCAATGAATCCATTGACGGGAATCAGTATTATCATCACAGCAAGTCCGGCGAGTACAGAAGGTCCTAGTTCCTTCCAGAGGAAATATAAGGACAGGCAGATCTGTAGTGGTGCAGACCAAATCATGTTGAGGTAACCAATTAGATCCATGAATTTTTGAGCATCAACAGCCATCAGATTAACGATTTCTCCTACTGTGCTTTCTTTTCGGGCACTGTTTGATATCTTCAATGCTTTTCTGTAAATCGTTGATACCAAGACAGTACGGATCCTCATTCCAACCACAAACATCCTGTTGAAGTACTGAGCTAGGAAAAGGGTTTGAATGGTGGCTGTGATGAAAAGTAGAACAGCGTAGAGGTAACCCTTCCACATTTCTTGGTTGAATCGAGTGTAGGAAATAAGGAAACCTAAAATCTGGGGGCTCACAAAAGTGAGCAGATCTTGTATCAACTTCAACAGCGCTCCAAAGAAATAGATAGGTCCGAAACATTTGAACAGTGCCGGTAAGATGGAGGCTTGTTTCTTCTTCTTGTTATTAACGAAATCGACGCTGGCCGAATCTGATTTAAATTTGGCATGGGCAACTTGTGTGGGGGAGGTGCTTTCGGCTTTTTTTAAAGTTTGACTCCAATATTTTTCAAAAACGGGTACCAACTCCCTCGAACTATCTTCGACATTGATATCCCACAGGTCTTTGGTTTCTAAAGGTTTTCTGAAACCTTTCCAAGCCAGCGGGTCGAACCATGCGAAGAGTAGACGTGATAAGAAGCTGGCACTCTCTTCTGGTGACGGATTCTGTGATTGTGGATACTTTCTTTCCAAAGGTGCCTTGTCCGCGAAACAGTTAAGAAGAAACATCAACACGACCAGAGGAAAATATATCAGGTAGCTGGTGTAAAAATAGTAATAATCGGGCAATATCTCGTTCCTTTGGCTTCTTCTGATTTCGGTCCTGAACTGAGGAATGGCACAAAGGACGACGAAGAACCAGAACAAGAATAACAGTCCGGACGTTTGTAGACCGTGCTTTCTATTGTAGAAAATAAAAACTCCGGTAAGGGCAAATGTTAGTATTTTGATCAACGGCGAGTAAATATCCACATCATTCACTTCGCTGCCTCTGTTACCTGCCGTTTTGAATGAGTTTACTATATCACTAATTGTTAAAACTATTAGAGTGGCTGTTATTGCTAATTTTGATAAATTTAACCAGTTCCATGGTATATTGCGTCGTTTGCTGTTGAATATGTAAAATACTTCTAGGCCGGCGAAGACCCATAGAAAAATACACGGTATCCATACTAGTACAGTTTTCTCGAAACACGGTGTGAACTCTGGGTCGTTTGTGTTCCATGTTAGGTTTGTGTCCCAAAAAGGAGATCCACAAAATGAACTCAACGCTGCATAAGCGTCTTCTTCAATAGTTGCATTTTGTTGTGAGGAGTTGCTAGTTAATTTTGGTAATTCGGTTACAGTTGTCGGGAGGGTGGTTAATTCTGTTAAGTTTGCCATATTTTAAAATACGAGGGTACAGGTAGCTGCAGTAAAATATTCCCTTTTTAATATCATCAATAGAATGCAATTTCAACAAACACCTTGAAAACCATCCACAATGCACATTTACTTGAACACTTTATGTAACATTAATTTAATAAAAGCGAAACACCGCTTGATTAACTGTAAATGGAACAGCGAGAGATCGTAATAATTTTATTCACAGCATTTCAGTAAACTGTAATTTAAAACTGTAAATCTGTAAAACCTCGCGCACGAAGTATTTTGTATTCCTCGAATCACCGAATCGCCGACCTGACCGACCTTACCTC

>Dvv ABC-C T17573_c0_seq1

GAAAATCTTTATTTTTAGAGATACAAAAATATTTGCTACATCTAAACACTACCACTCAATTTGACTTTTGTTTTTCCTCACCATCACCCAGATGAGAATTTCTCATCATTTCTGAAAATAAACTGGTTTTGTTGTCTAACAATACTTTGGGTGAATCAAATTCAATAACGTTACCTTTATCTAAAACTAATATTTTATCACAATCTAAAATAGCGTCTAATCTATGAGCTATAATAAGTACAGTACAAGAACTAAAGTTTTCTTCTATAATTTTCTGGGCTACTTGTTCAGTTTCAGGATCCATATTAGCTGTGGCCTCGTCCAAAACCATAATTTTATTTTTCCTTATTATAGCTCTGGCTAAACAAATAAGCTGTCTTTGTCCGGTACTAAAGTTGACATCGTCAACATCGACTTCGAGTTTTGGAACTACGCTGTCTAGTTGAACTTTGTGAAGAGTTTTCCAAATTTCCTCGTCAGAAAATTCTTTTAGAGGATCTATATTACTTCGTACAGTTCCTGAGAACATAATGGGATCCTGTGGAATAATGGATATATGCTGTCTTAGGAATTTGAGAGAGAGTTGTTTCAGCTCTACCCCATCGACGAATATCTTACCTTGGTAATTGTATAATCGATATAATGTGGATATAATTGAGGATTTTCCGGCACCAGTCCTACCTACAATACCAATCTTTGTTCCTGCCTTAACAGTAAACGAAATATCTTTCAAAACTTTTTCGTGAGAATTCGTGTATGTCATAGACACGTCTTGATAAACGATTTCTCCATTAGTTGGCCATTTTATATTATCTGTTCCAGTATTACTTTCACCTTCGATTGTTGTATACTCCATGGCTCTTTCAACGGACGTCATGCTGTTTTCCAATTCTGACCAAGCCGCTAAGCCCATGTGTACAATTCCACTTAGCATACCCGCTTGATTTAGAGTAAGGCCAACGTCTCCAGAATTTGTTCCTGTATCAAAAAATAAAAATCTGACTATCACAGTGGTTGTAAATGCTATTGAAGATATTTCCATCAAAAACGAGAAAGCGGCCTTAGCGCATATTGATGTGTAGAAGGCACTACTAAACAAATCTTGATGTCTATCGAATTCATTCTTTAAAATATCTTGTGCTTTATAAGCTCTTATCGTCGTCAAACCTTCCATGGTTGAGTTTAAATGGCCTACTAGTGGACTGCGAGTAGCTGCTTCAAGTCGTTTTAAACTTCTTGATGTTCTAATATAAATACTTCGGAGCAATAATGAAAAAACGGCTAATACTACCGCTGGTATAATAAATTTCCAGTTTATTGTTGCTATTAAACCTACGACACCAACCAAATGAAACAAGGCATCAACCATGTGCGACATCATCATCGACAAATGTTCATCGACAATACTCAAGTCCTGAGAAAATCTGTTCAATATATTTCCAATGAAGAAACTGTCGAAAAATGCCATCGTGGAGTGCACGATCGATGTAACCATCTTTTTATGGAGGTTCACAGACGCTCTAAATCCTACCCTTAAGATGATGTAACGTTTTAGCAGTTCAACGAATGAGTAGCCGATGACCAAAATTGTGTACAAATTTAAGGACTTGGTGGCCTTTACTTCCAGCCGTCCAATATTCCGGATGACTTCAGCTGATATATTGTACGTGCTATTATAGTATATTGCTGTTTGGTTTGTTAAGTCAATGTCTTCGAAGTTTATTGAAGTATTTTTCAGATGTTTCTCTTTTAAACCTTGAATGGTCGACTTTTCGTTGATCCAATTGGTTAACATTTTCTGTGACGTGCTTTCCGTAAGAGTGGCTCCGGCAAAAGTAAACAATAATAAGATTGCGAAAATAAATCCTCCACCCATTTTTATATACTGGATATACAAATCAAAGTCAACGGAACCTTGCTTTTTGTTTTCATGATAAACTTGTCTTTTCCGTAACTGCGGTTTTTCAAGAAGTTCAGTTTTTTCGTCAATAACTTTACTTTCTTCTGTCTCCATCACCACTTCTTCAATTTCTTCGTCTTCTAAAGCTTCCAAAAGAATATCTTCTGTGGCGTTTGCTTGATCTCCAATATATTTAATAGCTCCATCGTGTAAAATAACTAACTTATCCGCCGCATGAATATGTTTTGCGTTGTGCGTAACGAGCACCACACATTTATCTTTCAGAAGTCCTTGAATACACTGGGTGAAGATTTGTTCTTGGACTCTAGTATCTAAAGCAGTTAACGCGTCGTCAATTAAGTAAATATCACTGTCTCTGTAAATAGCTCTTGCTAGGTTGATCCTTGCTTGTTGTCCTTTACTTAGATTCATACCTCTGTCAGCAACAATTGTGTCGTCACCCTTTTCTAAAATCTTAAAATCGTATTCCAAAGCGCAAGCCGCCACAACCTGTTGGTACTTCTTGAAATCATATTTTTCTCCAAACAAAATATTTTGTTTGATCGATGAAGGGAATAGCCAAGGATCTTGTGAGGCATAGCTTTTCCGTCCCCTTGTTCTGACCTCCCCTCCGTCAAGAATCGGGTAGTCACGTAACACAACCTTTATCAGAGAGCTCTTGCCACATCCTAACTGACCGGTAAGAACATTGAGACCTTTTTCCAACTTTAAATCTACATTTTTCAATATATCCTTGTTCCTTAAATTTACGCTGACGTTTCTCAAATCAATTTGTGGATCATCATCAGGTTTGTCGATAACGTCAGGTAGCTCTTCGAGCTCCAAGATGCGATTGATTCTTTTAAGAGAAGCTGAAAGCTCTCCAATTCTTGTAAATCCCATGGAAAACGCCATCGCCATAGTGAATCGTAAAGTTCCAAATATTCTCATTATGTAAAATATATCTTCAGCGCTCATATCCTCATGTATATAAAGGTATATCATCAGCATAGCGTAAAAAGCAAACTTTCCAACCAAGCTACTCATTATCATCAACGATATTTTGGCATAGGCTGATTTGAGTAAAATTTTTAATTCTTTAAATCTAGCTTCTCCTATTTTATCGGCAAAAACTTGTTCCCAGGTATACATCTTAATCGTTTTGATGGTTGATAAACTTTCTTGTGTTCTTTGTAATCGTTCGTCAGTTCTTCTTGACATCTTTAGTCTTAGATTTTTTATTATTTTCGCAACGTAGCCTTGTACTGGTATAACGAGTACGAGCATTAGAATAGCTACTACAGAAGCTACTCCTGTCCTTTGGTAAATTAAATAACACATTACAAAAGTTTGTAATACAGATATCCATAAGTCGTTAAACATCCATATAGAGTGTTCAAACTGTTGAACATCTTTGTTAATGACTGTGACGATATTTCCCAAACTCGTGTCTTCTAAAGCTTTTGGACTTAACTTCAATGCTTTTCGATAAATAAGAGAAGAAAACGAAATGCGGATTTGAAGGGCTAACTCTGTTAAGTAGATGTGGTAATTTTGAAAGTAGATTGTGTGAAAAGCTTTAACTCCAATCATGATTCCAGCAAAATACATAGCATCGTGTATTGTCATTTTGGTTTGTCCAGGTTTGAAGTATCCTACCAAATGTGAAAGAGCGTCAGGTTCCAACGTACTGGTCAAAAGCCTTCCCGACATATGAAATAGTCCTAGTAATATATACCTCAATCCATACATATGCCATATTATTTTGAATATAGATACCTTCTTGTTTGGATCTTTAGTTTTAGATCGACTATTGTACTGGTGTTCTAGTTTGTCTGTACATTTTTTTGATCGACACTTCTTAATAACGTCATATAGGTCGTCATCCTCGAGATCCTGTTTAAATCCTCTTGTAAATAATTTTCCGGTATATCCAAATGTGAGAAGAGAGAATATATTTGCATTTTCCCTGGGATGGGGCGGCCGTTGCACTTTTTCACAATGATCCATTTTGATCAACTAACCACATTGAGAAACTGGATTTATAAAACGGATGAGATGTTATTGTTGGA

>Dvv ABC-C T48952_c0_seq2+T48952_c0_seq1*

CCTGAATGTTCAATCATTTTGTTAAAGATACCACCTTGTTTTGTTCTCAAGGCTTGTGGAGTATCAAACTCTATAATTTCACCATGATCAACAACCATTATCCTATCACAATCCAACACAGTATTTAATTTGTGAGCAATGGTTGGCACTGTACATTGTGAAAAATGAGTTTTAATTGTCCTTTGTAAGAGACTGCAAGTATCTGGATCCATGTTGGACGTAGCTTCATCCAAAACAATAATTTTATTTTTGCTGACCAACGCTCTGGCCAAGCATATCAATTGGCGTTGTCCAGAACTATAACGTGAACCATTCTCAACAATGACATCATCTAAACAGGTAAATAAATTTTTTAAATGGACTTTTTCTATTGCGGACCAAATTTCTGCATCCGTATGGGTTCCTGATGGATCCATATTTGCTCTTATTGAACCTGAAAATAATATCGGATCTTGTGGAATGATACCTATGCTGGATCGTAAATATTTCAAACATAAATTCTTAATATCTTCATTGTCTATTAAAATACGACCCTCAATATCATAAAGCCTGAACAATACTGAAATAATAGATGATTTTCCAGCCCCTGTACGTCCAACAATTCCAATTTTTTCTTTGCTTTTAATGGTAAAACTAATATTTTTTAATACTCTTTGTTCCGATGAGTTGTAAACTAAAGAGACGTTTTCGTACTTTATTTCTCCAAACGATGGCCAATTTTTCAACGTACGTCCTTGCAATTTATCTTCAGATTCAATGTCGGTATATTCTAAAACTCTTTCTATACCTGTCATGTTATTTTCTATTTCGGTATATTGCCTAATAACATATTGTAGAAAACCCGTAAGTAACATAGCCTGAGTTATTGCTAATCCAACGTCACCAGCTTGAGCATCTGTCTTAAAACAAACAAACTTTATTACTATGGCAGCAAAAAATGTAGTTGATACCATATCCATGATAAAACCGTAAAACCTAATGGCGCACGTCATCATAAAAGAAGTGGATGTATAATGATCTAGGTGTTGATCAAATTCTGAAATCAGTAATGGCTGCTTCTCAAATGCTCGAACGGCAGTTATTCCTTCAAGCGTTGCATTTAGATATCCAATCATTGGACTTCTAGTTGCAGCATCCAGTCTTTTTATGCCTCTCCCCGTTGGTAGATAAAACTTCTGTATAAAATATAACTTGACAAATAAAAATATTGAAGGAATGATAAAGAGTTTGTTGACGGAAATAATTAAGTACAATATTCCCAGTAATCCTAGAGTTTGCCTAAATATCTCGTAAGTATTCAAAGGAATAACTTCGTCCATAGTCATCAGATCTTTCGAAAACCTGTTCACAACATTTCCCACAAAGTGTTTGTCGAAGAAGCTCATAAAAGCGTTTATGACGGATTTCAACATTTTGCGATGTAGATTTCGAGACGCTCTTATAGCAAAGAAGAAGTTCATGTATATTCTAGTAAAGGTGAGTACTACCATCACTACTGTCAAGAATGTATACATCACAAGGTAGTTGTCTCTTTTATTTATGATGTTGATATACTCTTCAGTGTCAGTTTGATTGGAGAGCGTTAAATTCGTAATATTAGGTTCCAGATTAACCCACTTGCTTACAAGTTTTTCTGAGTACGACAGAGCTGCTTGGGCAGCAATAAAAATAATTGCTAGAATAACTAACATGATAATGCCACCAGCGTATTTGTAATATCTCAAATAAACTTTAAGGCTTACAGTACCACTCTTCTTTTCCTCATCGTACAAATTCTTATTCTCATTTTTATCTCCCCCAATTAGCTGAGTTCTCTCATTCGCTTCGTCCTCAAGTATATCTTCGTCTGTAACATCAATATCGTCGTCAAAGTAGTTCATTTCAACATCATCGATAAAATAAGTTATTCTTTTGTCCAAAGTTTCTTTTTGTTTTTCCAAAGACAGCGTTCGACCATCTTCAACAACTAGCGTATTATTTCCGTACAACAATTTGATGTGGTTGATGTTGTTGGACACCATGATAACCACTTTGTCTTTTAAGAAATCCATAATACAGTTTCTAAATATATAATAATTTACTTTTGAGTCTAAACCAGCTAGACACTCATCTATTAAGTATATATCACTAATACGATATATCGCTCTTGCCAGATTGATTCTGGATTGCTGCCCTTTGCTGAAATTGACACCTCTATCTCCTACTATAGTTTTGTCGCCTTTTGGTAGTTTCTTTAAATCTATATTAAGAGCGCACAATTTAAGTACTTCATTGTACCGTTTTTCGTTATAAGGTTCTCCAAAGAGTATGTTCTGTCGTAAAGTGGATGGGAAAAGCCAGGGTTCTTCTGACGCGTAGGAGATGGTTCCATCAATTGCCATTTGCCCACTGCTTACTGGATATTCACCCAGGAGAACTTTGATAATTGAGCTTTTACCACTACCAATGTTGCCAGTGATGAGATTGACTCCTTTTTCGGCCGAAAAAGAGATGTCATTAAGAATAGTTTTTTCTCCGACTTTGGCACTCACATGCCTCATGTAGACTCTCGGTGAAATAGTCTGTGATCGTCTGTCTACGACTTCCTCCAGTTTCAAAAAGTGTGACAATCTTTTAAGGGAAGCTCTTAAGTCCGAACATTGAGCTATACCAATTGGGATCGATACTGTTATGAACGATTTGAGACTTTGGTAGCAAGTTTGAATAAAATAGACTGTCTCTGCGTCTGTAAAATGACCAGACCATATATAGGTCATGATCATAAAGAAAAAGGAGAGGCTCGTTGCTGTGGATCCAACAATTAGCACCAAAGATTTTAGATAGAAGACCGGAGATAAGTTTTGCAGTTCTAACTTTCTTAATTCGTTAAGTTTTGTCGTAAAGAAGTTTTCCCAAGTGTACATTTTAATAGTTTTTATATTTCTTATAGTTTCTGTTGTCAGTTGCAGTCTTTCGTCAGTCCTCTTAGCAGATTGGACCCTTTTTGAGCTTACTACTTTTCCTACAAACAATTGTAATGGGATAGTCAACATGTAAAAGCCAATTCCTCCAAACACAGACCATTGTATTCTATTAAATATAACTCCTGTGATGATTATTGTTTGGATGACACCGATCCACATATCGTTTACAAATATCAATGCCATTTCGAATGCAAACACATCCTTCGTTATCAACGTTACAATTTTTCCAACAGTTACGTGATTCCAAGCATTGGGCCCCAACTTCACTGCTTTCCTGTAAACTAGTGCAGCTACAGAAGTCCTCACTCTTATACCTACTTCTGTAGTAAATTGTTGATAATTGTGGTTATAAATAACACTAAATACGTTTAAACCTACAACTAGGGAAGCATATATATACAAATCTTTTGTTGTCACATCTGTTTGGTTGGGAGCGTAGTACGCTACCACTTTACTAAGTGCTCTTGGTTGAATAAACACTAAAACTGTTTTCACAACCAATTGTATTAATCCCAGAATTAAATACTGCATACCAAAGCAGCTGATTAAGCACCTTACCAAGGAGGCATCTTCTCCTTTCTTTTTCCGTTGCCTCATCCAAGCATCTTCTAGATTATTTCCCAGCTGTTCTGATGCCAGCCATTTTGGGATTTCATATATATCACTATCATCAAGTCCTTTTTTGCGTGCTTTTTTAAACAGGGGGTAGTTATAGAAAAATGTTAGAAGACGAAAAATGGAAGCTTTGGTTCGGGGATTTGTTTCTTTGGCATTTCTTTTTGACGAGTCCATATTGGATAAGGACAGTCAAAAACCATAAACGCCTATCTCGAAAAATTATTAGTAGTTGTAAATGTCTCGAGTTGTTTCCTTATCTGTCTTATCGGCGATAATCCACTCATGATATTACCGATATATTTGTGTTGATTTGTCTAATAGATATACCGGGGCAGGTGTAGGTATTCATACCTGTTAGGTGGATCTTTCTGTAT

>Dvv ABC-C T44708_c0_seq1

CGCCCAACGTGGGGCGCCATTTGACTATCACAGCTAACTAAAATATTATTTTACCATAGCATTTCAATTTTCTGTTGTAGTTTACCATGACAAAAAAAATGAGGGATAATACTGGTATATCGTTGTCGGTAGAATATAACAATACCTTCTATAGCCGTAAAATATTACTTAAATTAATACACGTTAACTGGACATATCACCCTGTGCTACCATTTTACTAAAGTGTCCTATTTTATTTTCCAGCAACTCCTTTGGAGTACCAAACTCTTTTATCTGTCCTCGATCCAGTACCAACACCTTATCACATTCTAATATAGACTGCAATCTATGAGCGATTGTGAGGACAGTACTATCAGAAAAGTTATGTTTAATAGTTTCTTGTATTAGATTTTCTGTTTCATGATCCATAGTAGATGTAGCTTCGTCTAAAATAACTATCTTCGATTTAATTAATATAGCTCGTGCTAAGCAAACCATTTGCTTTTGACCAAGGCTGAAATTAGAAGCATAACTGCTAACTTTGATATCTAAATTTGTTATAGAAGTCTTTAGATTGGCTTTTTCTAAAGCTTCCCAAAGATCTTTTTCTTGAAACTCACCAAATGGATCTAAATTAGATCGGATAGTACCAGAAAAGAGAATAGGATCTTGCGGGATGATTGCTAGATGTTTTCGTAAATGTTTTAAGGGTAGTAGCTTTATATCAACTCCATCGATGGTTATTTTACCGTCTACTTCATAGAGTCTAAATATTGTGGATATTATGGAAGACTTCCCAGCCCCTGTTCGGCCTACTATTCCAATTTTTTCTTTCGCCTGAACTTGAAAACTCAAATTGTTCAAAATTCTTTGAGTAGAATTATAGGATAAACTAACATTTTGGAAACTTATGGTTCCGTCTTTAGGCCAATGTTTTGGTACGCTGCCTTGTGTTGTTTCAGACTTTAATCCGGTATATTCCAGGGCTCGTTCAGTAGCTGTCATTAAAGTTTCCAAATCAGCCCAACATCTCACTGCTCCTTGTATTAGAGCACCTAAATATATCACTTGGGTCAAGGCTAAACCAATGCTTCCAGCTGTGGCATCTGTTTCAAAAAGAACGAAACTACAAATGACCAGCGTCACCATAACACCAGCCAACATGCTCATATAGAATCCCATTCCGAACTTGAAACACAGTAGACTGTAGTGGGCCGAAGTGAAAACGTCTTGATGCCTCTCGTACTCATCGATGATCATATTTTCTGCTTTATAGGCTCTAACAGTGGTAACACCTTCCAGAGCAGCATTTAAATGACCTATCATTGGACTTCTTGTTGAAGCTTCAAGTCTTCGCAAATTTCTGGTTATTGGAAGATATAGCTTTAAAAATAATAACATTACTCCAAACGTTACTGATATATAAAAGAAAAAATATGGATTTACTGTCATAAGTAAAGTAGCAGCTGCACCAACATTGATTGCTACCTCTAGACAATTTAACAACTGAAAGGGTAAATTCTCATCAACATTAATCACATCCTGGGAAAACCTGTTTAATATATTACCAATAAAGTGTGTATCGAAGAAAGCCATCACTGAATGTAGTACATTCTTTATCATCGCTTTATGTATATTTATCGATGCCCTTTTGCAAAAGTCTAGAAAAGCGAAGGTTTTGAGTAGTTCTAGAACTAAGGAGAGTATTACCATCACGGTATACATTTGGAATGTAGAAGCAGCTTGAACTTCAGCTTGTGTTAGGTTTGTAGATAGTTGGGTTATATTTTGGCCAATAATGTTAGATATGTTGGCTTTTATGTTAAGAACTTTCTGTTTATCATCGCTCCACACAGATATCAACTTTTCGGAGTAACTTTCAGCAGCTTGCTTTGAACCAATTAGCAACAAAGCAAATGTAAACAGGGCAGCTCCTCCCCCATAAGATATATATTTAATATAGACAGATTTATCTACGGCACCTTTCTTCATTTCTTCTTCATATATATTTTTATAGTTACTTTGCTCGGTTTCCAGAAATCTGTCAACCTCCATAGACTCCATTAATCCATTTTCTTTGATTAAAAATTCTTTCGTTTTATATATGGTATCTGCGAAATTATTTACTTCCTTTTCAGTGTTTTCCTGAGGGTTATAGCTCCCTATAATGTGTCCTTTATCCAGTACATAAACAGCATCTGCTTGTGTTAGCGTAGATTTAGATTGGGTAACCAAAATGCATATCTTTTCCTTTAAAAACTCTTTAATGCACTTTTTGAAGATGAACTGATGAACGTGTGCATCTAAAGCTGAAAGACAGTCATCCAAGAGGTAAATGTCAGCTTGTCTGTATATTGCTCTAGACAGATTGACTCTGGCTTGTTGGCCTTTGCTTAAGTTTTGGCCATTATCTGTCAGGATCGTTTCGTCTCCCTTTTCAAATAAGTTTAAGTCATATTCTAGTGCGCAAACTTTTAATACTTCTTGGTACCTCTTTTCATTGTACTTCTCACCAAATAAAATATTTTGCTTGATAGTACTTGGAAAACACCATGGATGTTGGGAGGCATATGATACAGTTCCGCTACAAACAACTTCACCACTACTTAAAGGGTACTCTTTTAATATTGCTTTTAGTAGGGTACTTTTACTAGATCCTACTTTACCGGTAACCAACGTTAGTCCAGAATCAACCTTAAAACTGATTTTTGACAATAGTTCTTCTTTTCCTACACAAACTGTAATGTTTTTTAACTCTACATATGGCTTAACATTGTTTTGTTTACTTTGATGATCTTGATCTAGTTCTTCGGCTATCAGTACTCTATTGACTCTTTTAATTGTGACAGACAATTCTGCAAATCTTCCTACAGCTTCTGGAAGAAGATATCCAAAAAATATTACGATGTTGTCATAATGGGCAGTTATGAAGAAGATTATAGAGGCATCTGGCGGTTGTTCCATCCAAATATAACCCATAATCATTAAGTAGAAGCCGATTTTGGTTGAGATAGCACCCAATAGCATTAGTATCATACGCAGATAGAAAACTATAACCATTTTTGCTACTTCTCTCGTTCTAGCCAAGTTAATTTTTTGGTAAAAGTAATCTTCCCAAGTATACATTTTAATGATTTTTGTTGCAGATAGTACTTCCTGACTAACTTGTATTCTTTCGTCGGTCATCTCACTTGTTTCGTTGCGCATTTTAGTCACCATTTTACCAAGAAAAAATTCTATGGGAATAATGCAAAAGTGCATACCCACTGCAAAAAACGTGAGTACACCAAGGCGAGCATATAGTAAATAACATGTGACACTAATAACTACAGTAAATACCCAGATATCATTAAAAGCAAACATAGATCTTCTGATTTGCTGGACATCTTTGGTTATCAACGTCACAATATTTCCCAAACTTGCTTTGGTCATGGCCGCAGGGCTGAGCTTGAGGACTTTTCTGAACAAAAGCGAACACAAAGCTGTTCTGACTTGTATACCCATTATAAACAGGAAGAGATCGTAGTTGAAGAAGTATATTTTCTGCAAAAAGTTGGAGAATAGTAAGATGGCAGCGTTGAAATAAGCGTCGGATTTGGATAGTTTTGTTTGTCCTGGTTGAAAGTAGGAAACCAAGTTTGTGATGGCGTTGGGTTCAAAAATGCTTCTGAATAATTCACAAAACAATTGAGTTATTCCCCAAAGAAAATACTTCCATCCAAAAACTTTACACAAGGTTAGAAAGAGAGAAGGATATTTTCCATCTTTTTTTCCCTTTTTCCATTTCTTTTCAATTTGTTCGGCACAATGTTTGGATGCACAATTTTTTGAAACTTCATAGAGGTCCTCTTCTTCTAAATCTTTCTGCACGCCTCGTTTTAAGAGATCTGTTATGTAGCTAAATGTAACCAAGGACCAAATATTGGCCGAATCTCTAGGATTTAATCTTCTTTTCTTAATTTTTGAGGAATCCATAATTTCTAATTTTTATTGAATAATAAAAAATAAATTGTTGAAGATTAGTCGAAAATTCTTTTGACTTAGTATAGTTCCAACAATCCCACCATTATGCCAACTAATCATCCTAAGACTTCACCATCACCAATTACTGTCACATTATGCAGTTAAAAAATAGCAATTTGCCAAAATTTCCAGAATTGTCCATACTAATAACGATTCGGTTACTTGGTCTCACTCCTACATTCAATTAAATAATGCTGTGTTAATAGCGGAAGTAATTTTCGAAATTATAAGGTTACAGATGCGGATTAAGATTTCGTGTTCTGGGTTTAGGTTCTCTATTAGACGATGTCGCATATTTAAATTTTAAGTAACTAATATAATATATTTACCCGTATAAAGACAATACAAGC

>Dvv ABC-C T41801_c0_seq1

ATTTAATTATATTCACGTAGATCTTAAGAAAATTACATATGAACTGGTCCTGCTTTCACTTGTTACCACCTTTTGTATTTGATACAGAACTGAATGCTTAGGAATGATGTTGTGTAAAGTGCTTATACGCCATGTCTTCCAAATGGGCTGCGGTCGCATCTCCAGTTTGCTGCACTAGATCATAGAATTTTCCACGCTTAAGTATAAGTCGATATGGATGATCACATTCCACTAAATATCCGTTGTCCATAAGTAATACTTTATCGGTCTGCATGATTGTTTGTAGCCGATGGGCTATGGTAATAAGGGTAAAGTTGGCAAACCTCATCTTGAGAGTGCGCTGAATTAGTTCATCGGTTCTTTGATCTACGTTAGCTGTAGCTTCATCTAATATTGCTAGCTCATTTTTTCTTAGTATTGCTCTGGCCAAACATATTAGTTGTTTCTCTCCTGTACTAAAATTGTTTCCACCTGACGTCACCACTGAGTCCAAGGATGTTATGCAGTCCTTTAATTCTACCTGTTTAATGACTGTCCATAAATTATCATCTGTGAACTCCCCAAACGGATCTAAATTATACCTCACAGTATTGGTAAATAAAACAGGATCCTGTGGTATTATTGATACCCGTTTTCTTAATATTTCCAATGGCATCGTCTTTGTATCCACTCCATTGATTTGTACTGAGCCACTAAATTCGGAAAGTCGAAGTAGAACGTTAATCAAAGAAGATTTGCCAGCTCCTGTCCTTCCAACAACTCCCACCTTCTCTCCTGAAGCTATACTAACATTTATGCCGTTTAAAACTAAAGATCCTTTGTTGTAAAACATCGACACATTTTTAAATACAACCTCAGCTCCGGAATAACCCGGTTGAAAGTTATATACACCATTTTTTTTGGTTTCAGAAAATTCTTCAGGTTCAATTTTACTAAATTCTATCATTCTTTCTATACTCATGAGATACTGTACGAAGTCCAAGGACTTTTGTGTAGCAAACTGAAGGGCACCCATTAGCTGAAATACTTGTAAAATGGCCAGACCTGCGACACCTGGAGTAATTTTGTGATCATATTCGTTGAGAAGAATAAGTGCAAATATCACTCCAAATAAAAAGAATGCACAAACTGTATCCACAATTACAGAAAATGCAGCGCCACATAGAACATTTAAACGAAGAACACTTGTGTGGTTATTCAATAATATAATAAATTCTTTTTGTAGTTTTTCTTGATTTTTGGTTGCTCTTATTGTAGTTATTCCACTTAAAGTGTTGTTAATGTAAGTATATATTGGAGATTTGGCTGTTATTTCCAAGTGTTTTAATTCACTTCCAATATATTGATACCATTTTAAAAGCTTGGCATAAATGAAAACAATCAAAACGAAAAAAGACATATAATAATCCGAAACTATTACTAGAATAATTGATCCTATACCCATAAAATATACCTGAGAAATATCTTTAAGATTGTCTGCAAATCTCACATCGACTGCTGTAGTATCTGAAGAAAATCGATTCAAAATTCTCCCACTTGGATGAGTATAAAAGAAGCTCAAAGGAGCGCTGATAACTGCTTTGTACATGTTTTTGTACATATTTTTGGATGCCGTATTAAAAAACTTTGTTAATAAAATATTAGACAATATTAACATCAAGAATGCGCTCGATACGATGGAACAATATATAGTAAAATTAGTATTGAATTCTGTAGTCCACTGAATATCTCTCATATGATGAACATTGGAGTTTTCTGTTACATTTTTTGATTCAGCGTGTGTGGAATAAGATTGTTTTGTCCAATATAACAACCAATAATCAGTCAATCCTAAGAGAAGTTGTGCACTGAATACAGTTAAAAATAGGGGAATAATGCAAGAACCAGACTTAATGTATTCAAACAGTGCTCTCCTTGGAGTTATTTTTGTTCTATTTTCATTACAAACATAGTTCAATTTCTGGTCTGATTCATCTTTAAGGTTCAATTTTGACGTCACAGTAGTTTCCTTTGTTTCATCGGCTTTATTTAATTTTTGTATCCATTCAAAAATTTCAGGTTTTTTAAATTTTATCTCTGACAAACTATCAAATGATTCTAAATTGCCCTTGTCTACAATAATAATTTGATCAGCTTCTTGTAAATACTGCAAGTGGTGTGTTACTAATAATCTAGTTTTGTCTCTTAAATAACTTTTTACACATTCATCGAAAATTCTTTTTCCTACTTCAGAATCCACAGCAGACAACGGGTCATCCAAAAGGTAAATATCAGCATCTCTATACACCGCTCTTGCTAGATTTATCCGAGCTTTTTGTCCCCCACTCAATAGAACACCTTGTTCACCTACACAAGACAGGTCCCCATTTCTTAGCTGGTCAAAATCAGGCAATAAACTACAAGCAGAGACTACTTGTTGGTACTTATTATAATCATAATCATTGCCAAAGAGAATGTTGTTTTTAAGTGAAGAGGTAAATAACCAAGATTCTTGTCCACTGTAAGACACTGAACCATTCATCTGTATGTTACCAGAAGATAGCTTAAGCTCTCCTAGAAGTAACTGCAATAGAGTGGATTTTCCAGAACCTACAGGTCCAATGATTACGGTAAGCGATCTTGATGGTATGTTTATGTTATTGGATGATATAACAAATGATGACTTTGTAGACCAAGATGCTTTGATTTGATCCAACTGAATACCAGACACATCTGCATTAATTTTGCTATCGATATCAACTTCATTCATAAGTAACAACTCTGAAATTCTAACAAACACATTAGTAATTTCAAACTGTAGTAGCATTGCTTGGGGCATAAATGCCAAGACTCCTTTTTTCAATATCACCAGAAATTGAATGCAAGAGAATATTATTTCGGGGAATAGGATTCCATTGACATACAACATTGTTAGGACTGAAATTGCACTGGCTAGTTTTAAAACAGCGAAGTGGTAATAGCCTATTTGAAACAATAAATATTTAGTTATATTTCTAAGTTCTTCTTTTCTATAATCGTTGATGATTTTGGTGAATGAGAGTTCCCATACATACATTTTGATAGCTTTGATTCCAGATATTATTTCTTTCATCATGTTTACCCTGCTATCTGCAATTTTTGATGCTTTAAGACGATAAACTGAAGAGATTTTACTTCCTAAAACCATGATTGGTAACAGGATAGTAAAGAATAAGGCACCACTAAATGCCGCTGTACCCAGTAAATACCACATACTCGCATATAATGTTAGGATTTGTAGTGGTGTCAATATCAGATATCCCAAATATGTTGTGTACTGAACAATCCTATCTAAATCATCAGCTAAAATATTTATGATCTTTCCTACGGTTGCTTCTTCCATTGACTGAAGATGCAAAACCAAGAGCTTGTTATATATTAAAGAAGACAAACCTGCCTTCATCCTAATTGCAATTTTTCGTAGCTGGTAGTGGAAAATATAATCTGAATAATAGTTAATTACGACAAGGACAACAACCATGCCTCCTAACATTATTCTTTCTCTAAATCGGTACTCTTCTGTAAAGGTTCGGTTCAAAATTGTTAATAATACACATGTAATTACCCTCAAAATAACAAAGTTGACTCCCGTGAATGTGTAAATTATTGCTAACTGTTTCCGAAAAATTCCCATTACAACTTTCTTTAAATTCGGCTTTTCTTTGTTTTCTTTTGATCTTTTCACTTCTGCAGCCCATTTTTCCTCCAACTGCTTTAAAAGAATTTTGGCATCATCCTCAGCATTAACAGCAAAATAAGAAAGTTTCAATGTACCATTAAGGATTTTTCTAAAAAATGGAATAGTCCATAAAAAGAATATACGTGATAATAAACATGCTTCATACTCGGGGCTAATTTCTTTTGTTTCAGTTGCTACGTCCATTACTTTAAAGTTATTTACACACTTTAAAACTGTGGTAATGTTTTGATAATAAAGCTAATTACTTATTGTAAATTACTAAAAAAGTATAATGGT

>Dvv ABC-D D11014

GTGATGGGATCGCCTATACTTGTAACATTACCAATGGCCGGCCTTAAACTCGGCAAACCTGCCGAACCACGTTGACTAGAGTGACGTACGGTGTTGCCAAGTTCTTGAACAAATACATAATAATTACCAAAATTCACTAGATTGCCGTATAGATGTTGCCAAATCCTTGACTGAACACGTAAGAATTTTAAAGTTGACATTAAAAATAGAAAAAAAAAATGAAAATAAAACTTTTTTTAAAATGCTCAGAGTCCTTCATTTACCGGTATTGTATAGTCCGAAAAATTGTTTGGAATTATCACAGCACTATCACCAGGATGAAACTGAAAGAGTAAAGGCAAGATTTGTAGTTTTAAATGATTCGACAGGACGTAGATGAATACTATGAACTCTTCTCCTGAATATTTGAAATCTACAGTAAATGCTTGTCAGTTAGCTAATCTCTAAGTAGTTATGAATCCTCTCCAAGTAATTTGTTTAGTTCGTCCAGTCTTTTCGATCGTTCTTCGTTGTTTTCTGCTTTTAGAAGGTCCTCCTTTTCTTTCTTCAAGGTAAGTCTGCTGGTGTGGTTCAACTGACTAAATTCCCATGATCCTGTACCGTCGAATTGAAGGATGTGCGTATGGAATTTCCATAGTGTAGGTCTATGTGTGATGGTGAGTAGAGTAATTCCCATGTCTATAGCACTTTGGTATATGAAGCTTTCAACATCAATGGAGACTGCAGAAGTGCATTCGTCCAAGAGAGCGTATTTCGGTTTGTGATAAAACAATCGCGCAATAGCCATCCTCTGTTTTTCACCACCGGATAAGATGTCAGTCCAATCTTTCATTTCATAGAATCCGTCCCTCTCGACGATATGATCTAGATGTACCATAGTCATTATTTTCAGTAAATTTTCTTCGGTAATACCTTTCTTTACCATATCCGTGTAAGTATCTGGGTATATTACTTGGTCTCTTAAATTACCTATTACCATATAAGGCCTCTGAGGAATATAAAACATTGAATTTTTAGGAGTATGAAGCTCTCCACCGTAAATAGGCCATAATCCACTCAATATTCTGAACAAACTGGACTTTCCACAGCCATTTGGACCCGTAATTAGCAAGTGTTGTCCCGGAGTCAATTCAAGACTTAGAGACGGACACACTATATCGCAATTTGGAGTTACTATAGGAACATTCTTTAGGATGATCTCGTTATTGGTTGAGTAGATGATTTTACCTTTGGCTAAAGGTTGATCACCACGGAACTCAATCTCGAAATCAGCAGACTTCTCTTTCTTAGCGACGAGAGTCTTATGGTAAATTCCGTTGCTGGCTTCCTCTAAAACCTCGAACATGTTGGCCACCCTGGCCGTATGACCAGCTAACTCGACGATGTTCTTGTAGCTGCTCATGAGCCGTTCTACAGCATCTGAGCCAGTTATCAACAGGTTCTTGGAGGTAGTGAAGTAGTGCGTTCGTTCTGATACGCTATCTTCTATTTGGTCGTCGGCTGTGTCTTCAGCGATGAGAGATTCTGATTTGGATGAAAAATCTGGTATGGAAAGAAGACTTTTGTTGGAGATTTTGCGTCGATTGCCGGCTGCTAGGAGAATAGGCAGAGATACGACTATTATACCTGCTCCAGACCACACATATTTCATCAAGAATTGCTCCAACATGATGAACCATAATTTCACACCGAACATGTGTTCCAAGTGTTTAGATAAAACTCTAAAAGCTTGTCTAAGTTGACTTTCTTCGACCTGATGGCCTCCGTAGAAAGCGATTTCTTCGGCATTGCTGACGATTCTTCCGTGGACGTGTCGTAGATATCCTTTCTTCTCCGCTTCTTGGGCTACCAAGTGGCCGAACCTTGGAGACACAAACCTTAAAAGTAAAGCTGAGAACATCACCACTCCGCAAATAATCACAGGTCCTATCACAAGATTCGAATGGCGACTCTTCACCAAATTGGCTAAAGCTATAACCATGAGAAGTATATCGAAACATGGCTTCGTTATCTGTCCGTAAAGGTGCGACACGGTGTTGGCGACAGTTTCTATGTCATCTGTCAGCCGCTGCGCGCAATTGTCTAATCTGCCGTCTAGCACAGTCACCCTGTAGTAACTCTGGTTCTTGAAGTATAGTTTGTACGAGTGGTCTACCAGTCTGGTGCGGAAACTCAAGGCTATCCGACTTTCAAGATATCTTATCATGCTGTTAATAAACGTAGCTGGAATCGCTACGGCAAACCATTTTCCTAGCTGCTTAACGAAGTTCTGGGGGTCTTTCATCACAATATACTTAACTATAGCACCTTCTAAATTAGCGACATAAATGCTCAAAAACGTCCTCAAAAACAAAAACGTCGTATGCCCGCTGAGTAAAACCGTTTCAGTACAAATGAAACTGGGTATCATTATCCTAACTAACTTAATAAATTGTAAGATAAAGGCTAAATTGAAGTTCGGTATACTATTTTTAAGCCTTCCTTTGAGTTTCTTGTTCTTCACTAGGCCATTCTTACGTTGCAAGTCTTTTTGAACTATGTGGTTGTTGTTGAGATTGTCCTTTGGTTTGTGTATCAAGGAGTCCACGAAGGGGTAGCCGACTTTGTAGGCGTAGGTGCAAAGAACGGCTGCGATGAATGCTCCGGAGAAGACGGATTTGTTCTGCTTGAAGGTGCTTTCACCCTGTTCCAGGAACTTGGAGATTACTGTTGGCATGTTGTTTGGTCTGGTTTGATCTTGGTGAGTATTTTAAATTACTAAAGCTATTTGGTCAAATTGCGTTGGTGGTTTGTTTAAGCCTTAGGTTCCTTGTTCCATGGTTTCAGATCTTATTGAATATAAACAGCACCTAAACTACACTATAAAGTGAAGAACCTCACTCACTTTGTGTCTGTATTCGACCGAAGGTTTTAGCAATGGTAATTCTGCAAGGAATAATTCGAATAATAAAATACAAACACGGTCAGTATCGATTTTGGCAAGGTCGACAATAATTACACCAAACACTGAAATCTCGAGATTACCTGAGAACGTCGATGTATAAACCGAGAATGAAAACAAAAGTGTCTGTACAAACCAAAATACTATCTAAACAACAAACTGACCGGAGGAGACAGTATTCTGACTGAAGAATGTTACTCTGCTCGTTTTGTGTGAATAGAATGAATAAAGCAAAACAAAAATTGGAACGAGGTAGCGGAGCGGGTATCTCACTCGGACTCGGTCG

>Dvv ABC-D D11628

AGTGTTCTTATTTTATGTAAAAAAATATACACTTTCACACACTGGTGATATACACTAAGGTATCAATTGCCCTTGTGGTATATAGCTTTGTGTAGTTTGTTTCGACATTTGTTTCTGGATTCTACATTTGTTTATTGAGAAATTCTCCTAAAAATATATTTCATAATTCGGACACTATGATCCGAATTGTTCATCACAATTATCAATTGGTTTAAAACTATATCCTCCTCGCCCGTCTAAATGTAATACGTATTCGTGATGCTGCCAAAGTGATTTTCTATGTGATACTGTAAGTAACGTGATGCCAACATCTCTACAATACTTATACATACTGCCCTCTACATCGACAGATACAGCGCTTGTGCATTCGTCCAAAATGGCGAATTGAGGTTGGTGGTAGAATAATCTGGCCATAGCTATCCTTTGCTTCTCTCCACCACTCAAAACATCTAACCAGTCCGCTACAGCATCTAGACCACCCTCTCTTTCCAAAATGTATCCGAGTTGCACCCTTTGCAAATACTCCTCCAACTTGGCATCTGTCGTTCCTCTTCGCGCTGCTTCTGCCCCAGAATGCGGGTATGTCAGTTGATCCCTGAGGCATCCTAACGTCATGTACGGTCTCTGTGGGATATAGAATAATTTCCCCCTAGGTGGTTTCGTGAGCTCACCACCGAACAGAGGCCAAAGCTCTCCTAAAATTCTAAACAAGGAAGACTTTCCGGCCCCATTGGGACCACATACCAACACATTCATGCCGGAGTTAATTTCAAAAGTGATTTCGTTAATGAGAACGTCTCCATTGGGGGTTATTAAAGGAACTTTGTGGAATTTAATGATGTTGTTTCTGAAAATTAAYTTTCCGCCGTTGATCTGTAAAGATTCTGATCCGGAAACCATTGTTCGTTGGTATTTTCCAGAGTTAAGTTCACTCAACACTGTCTTTAATTGTGTAACTCTAGCTGTGAAACCTGCTAGGCGGGTTAGATCCCTTCCTGCTAGGACCAATCGACCAATTGCTTCGGCTAACTTTACTAACATCCTACCGTACGTGTAATACAATCTTGATCTTTCGTTTGTTCCAAGAAGCCTAAAGCTGTGTCCACTTGTCATAAATGGCAACGACACGACCCAAAATCCTACTACTCCGGCAAAATATTTAGCAACAATGTTGTCCACGACGCCCATTGCGACTCTAAACCTTAAGAACTTCCTTAAGTGATTTAAAAGCTTGTTGTAACTGGCCATGAGAGTTGCCTTTTCTCTAGAGTTACCGTTGTAGAAGGCTACTTCTTCTGAATGGGTGATTAGACGTGAATTGATATGCCGAAATTCACCTTCTAATTTTTGTTCTCCCGCTGTTAATCTTGCTGTTGGTTTTCTCAAATTCGTTAATAATACGCCAGAAACTAATAGATACACCAGCATAATTCCAGGAGTGCCCCCTCCCAATGTTGTCGATAACTTATAAACATAAATACAAATATCCAGCAAAGGTTTGGCAGTGTTGCAATACAAATCCGTAACTCCTTCACAAAACTTGTCTATGTCGGTCGTCAACAGTTGATCTGCGTTGGATATCCTATTGTCCAGATTTGATATTCTGTAGTAAGTGTAATTCTTCAGATATTCCTCATAGAGATGTCGTGTCATGTTAGTCCGTAGTTGGATTTTCAGAGCACCTATTGAGTATTTCAGGACATTGTTTACAACTGAGATTATTGGAATAGCTAAGACGTAGTAGAGCAGGCGTTGCTTGAAGAGAGTTGGGTCCATTGCGATGATTGAACTTTCGATTTTAGTACCATGATTGATCAGCCACAAATCGCACATAGATCGGGATACTAAACTTAAGGCGATTAAGAAGAATAAGCCACTTTCTTGGCTGGTCCATCCTGGGCAGGCGATTCCAAATAGTTGCGATAATTGGGAGAAGAATTTCTTGTCTACTTGTGCTTTGATTTTAGTTTCTCCTTTTTCCGATATTAAATATTTCACCTCTTCTTCTACGGCTTTTTGTACTTTTTTCTTGTTAAGTTTTCCCTGTTTATTCCTATATTTCAAAATAATAAGCAGAATTGTTCCTAAGGCTCCTGCTCCAGCAAGAGCCTTGTGTTTGTTTAAAACTTTGCTATAATTAGGAGCCATCACTAACACATTTTTATTAATAAACTTATAAAAAAAATTAGCGTAAAATCTACCGTTCACTAAAATCGAAACGGATTAATTACCCAACTAACTTCTCCGCCGCTCGCGCTTGAATAAAAATAAAATGAAGTAATTTTAAAAGTTT

>Dvv ABC-E D2830

CTTTAATTATACTCAAGGGATACAATTTATTAAAGTAGTAAAAAGTAGCTCAATATCCTTTGCCTGCAATCGATTATTGACACGTACAATTAATAATTATAACGAACGTTTTCCTGTCTAACTGGATACAAAAACCAACGAATTAATCCTCCAAGAAGAAATACTGCCCGGCCCTCTTCTGCTCCACGTCCTTCACCGACTCAAGCTTGTTGATCCTAGGTCGGAAATTGTTAGGATCACGTCTGAAAGTTATGCCGAGGAGTTCCAGGAACCGGTTCATTCCGGCCAATAAGGTTTGAGGGGCGTGGGCTGTCGTCTTCACTGAGGGAGACCCTTCGAATACGATGACACGATCGGCCAAGTAGGTCGCCATTATGAAATCGTGCTCTACTACGAAACCGGTCTTCTTCGCGTGAAGTATAAACCGTTTTATGACCTTAGCTGCTACCAAACGCTGTTCAGAATCCAAATAGGCACTAGGTTCATCAACAAGGTATACATCAGCGGGTTTTCCAAGACAAAGCGTCATTGCCACTCTCTGCAATTCTCCTCCAGATAAATTCTGTACTTCTTGATCGATGATGTCTTCTATCTTCAAAGGCTTCATTACATCTGCTATAAATTGTGGATGAATATAGGCATCCCTAATTTTCTCATGAAGCAACTGTCTTACCAATCCTGTGGATTTAGGACTGATCTTTTGGGGCTTATAACTTATATGCAACTGGGGTAATTCACCTGATCCACTATCAGGCTCCAAATTTCCAGCCAACATTCTAATAAAAGTAGTCTTTCCGGTACCATTCTCTCCCAACAGCACCAAAATTTCAGAATCAGAAAACTGACCCTGGGCAACCTTCAACTCAAAACTACCCATCGTTTTAGTCATAGTTGGGTACTCGTAGTGGTTCATACGTTTAATTTCTTCTTCTGTAGCCGACTCTGCTACTTTAAAAACTAAAGATTCATCTCGGAAACGGAGATTTTCGGTTGGGACAAAACCGTCCAGGAAAATGTTGATACCTTCACGGACAGAGAAAGGCATAGTTACTACACCATAGGCACCAGGCACTCCATAAAGACAGCAAATGAAGTCTGACAGGTAGTCAAGAACACTCAAGTCGTGTTCCACCACAATAATAAACTTGCTGGGATCTATCAGGGATCTGATAGTTCTGGCAGCATTTAAGCGCTGTTTGACATCTAGATATGATGAAGGCTCATCGAACATGAATATGTCTCCATTCTGAATACATACCATGGCACAGGCAAATCTTTGGAGTTCTCCTCCAGATAATGCGGCGATTTCACGTTCTTTTATGTGCAGCAGATCCAACATCCCACAAATTTGATTCATATTGTCTAGTTCATTTTTTCTGTCTAGAAGTTGTCCCACAGTGCCTTTAACTGCTTTGGGAATTTGATCAACATACTGGGGCTTTATAAGAGCCTTCAAATCATCCTCTAGAATCTTAGTGAAGTAATTTTGCAATTCGCTACCTCTGAAATGGCTTAGAATTTCTGTCCAATCTGGAGGATCCATATATCTTCCTAGATTGGGCTTTTGTTTTCCTGCAAGAATCTTCAGGGCAGTAGATTTACCAATACCGTTAGTGCCTACGAGTCCAAGTACCTCACCAGGACGAGGTATAGGTAACCTATGGAGCTTGAATGAGTTCTTACCATATCTATGAGTAGTTTCTTTTTGCAAATTACTTGGTAAATTAATAATAGCAATGGCTTCAAAGGGGCATTTCTTAACACAGATACCACAACCAATGCACAATTCCTCAGATATGGTAGCAATTTTACTGTTTGGTACAACTTCAATGCACAGTTTTCCAAGCCGGACCACTGGACAAGATTTCTTGCATTCCTGCCTACATCTTTTGGGTTTGCATTTATCTGCATTGACAATGGCTATACGGGTGAGTTTGTCAGACTCTTCCATTCCTTTTCGTTTGGACATTTTGCTAAAATCTGTCCTGTTTTTTTCCTTTAAACAGAAATGAAGACGGCAAACGTCACCTTTTAATAATTTGCCATGAGGAAAGGAACAA

>Dvv ABC-F D2701

GGTTAACATATTGTTACCAATTTTAATATAATAATTTAGAAATGTCGAAAAAACGTGGGGCCAAGAAGGGAAAAAATTTGGATGATGATTTTGAAGAAACCTCCAGTATTATTAGCGAAAAAGAAAAAATAAATTCGAAAACTCCCAAAAATAAGACTACTAAAAAAGGGAAAAAAGGAAAGGATGACTGGAGTGATGACGAGGAAATTGAACAAGGCAAAGAAGTTAATCAATCTGAACCAGCGGAAAGTAAACCAGTAGCCAAGAAAAAGGGCAAAAAGGGCAAAGGCAATAAGAATGATGACTGGTCAGACAAGGAAGAAGCGGATATAAAACTTTCAGACAGTGAAACTGAGTCAATGCCAGTAGCAGTGAAAAAATCCGCTAAGAAAAATAAGAAAAAGAAAGATGATTGGTCAGATAAAGAGGTTGACATACAATTATCTGAAAGTGATATTGAAGAGGCACCTAAAATTGTTAAAAAGTCAGCTAAGAAAAATAAGAAAAAAGATGATTGGTCTGATAAAGAAGATATAGAAATAGAAACTAAATTATCTGATAGTGAAGAAGAACTAGCACCAGCTATTGTTAAAAAGTCTGCTAAGAAAAATAAAAAGAATAAAAATCAGGTACGTGATGAATCTCCAGATATTGAGGAAATGGAAGCACACGATGAAGGTTTTAAGGAGGATGACGTAAGCGAGGAAGAAATTGTAAAACCTGTTAAACAAGACAAAAAGAAAAGCAAAGGCAAAGAATCAAAGAAGGATGAAAGTAAAGTTGATGTAGAAGTTAAAGAAGAAATTAAGGAATCTAAAGAAGATTCCCCTATAGTAGAAGACAAAGTAATAGAAAATGGCAAACCTGAAAAACAAAACAAGGAGCTAGAAAATAAAAAGGATAATGATGTAGAAGAATTAACAGAAAAAATAAGTACTACGCAAATAAGTGATGATAAATTAGATGAATCTAAAGAAAAGAAACTCACGCATAAAGAAAAGAAAAAAATGAAAAAACTACAGGAATACGAAAAACAAATGGAAACCATGTTGAAAAAGGGTGGACAGGGGCATTCTGAATTAGATAGTAATTTTACTGTATCACAAACTCAAAAGACTGCTGGACAATTGGCTGCATTTGAGAATGCAGTAGATATAAAAGTTGAAAATTTTAGTATTTCTGCTAAAGGTAATGATTTATTTGTGAATGCCAATTTATTGATAGCCCAGGGCAGACATTATGGTTTAGTTGGACCTAATGGCCATGGTAAAACAACACTGCTGAGACACGTAGCACAACGTGCATTTGATATTCCCCCAAATATCGATATTCTCTATTGTGAACAAGAAGTCGTGGCAGATGATAATACTGCTGTTGAAACCGTCCTAGCAGCCGATGTCAAACGTAATGATTTATTAGCCGAATGTAAAAAATTAGAAGCCGCTGCTAATAGTGGAGATTTAGAAATTCAGGAGAGGCTTAACGAAGTTTACAGTGAACTTAAAGCTATAGGGGCCGATTCTGCTGAACCTAGGGCTAGGAGAATTTTAGCTGGTTTAGGATTCGATAAGGAAATGCAGGATCGTGCTACCAAAAATTTCTCTGGAGGTTGGAGAATGAGAGTATCTTTAGCAAGAGCATTGTATATTGAACCAACGTTACTTTTGCTTGATGAACCGACTAACCATTTAGATTTAAATGCTGTAATTTGGCTTGACAATTACTTGCAAGCTTGGAAAAAGACTCTGTTGATCGTTTCCCACGACCAGTCTTTCTTAGACAACGTCTGCAATGAAATTATTCATTTGGATAACAAGAAATTATACTATTATAAAGGAAATTATTCCATGTTTAAGAAGATGCATGTGCAAAAGAAGAGGGAGATGATCAAAGAGTATGAGAAGCAAGAAAAGAGGATAAAGGAATTGAAGTCGTCAGGTTCTTCCAAAAAACAGGCGGAAAAGAAACAAAAAGAAGCCCTTACTAGAAAGCAGGAGAAGAATAGAACTAAGATTCAGAAACAAGAAGAGGATACAACTCCCACAGAACTATTACAAAGACCTAAAGACTATTTGGTCAAATTTCGCTTCCCAGAACCTCCACCTTTGCAGCCACCTGTTTTAGGATTACACAATACAAGATTTGCCTATCCTGGACAAAAACCTCTATTTGTAGATACAGATTTTGGTATTGATATGAGCAGTAGAGTCGCAATCGTAGGTCCAAACGGTGTTGGCAAATCAACATTCTTGAAGTTATTAACAGGAGATCTATCTCCTGATAAAGGAGAAAATAGGAAAAACCATAGATTGCGAATTGGTAGATTCGATCAGCACTCTGGTGAACACTTAACGGCTGAAGAAACACCTAGTGAATATCTGATGCGTCTTTTCGACCTTCCCTATGAAAAGGCTCGTAAACAATTAGGAACATTCGGATTAGCTAGTCACGCACATACCATTAAGATGAAAGATTTATCCGGTGGTCAAAAAGCAAGAGTGGCCTTGGCAGAACTCTGTTTGAATGCTCCCGATGTATTAATTTTGGATGAACCGACGAACAATTTAGATATAGAATCCATAGATGCCTTAGCTGAAGCAATTAACGAATATACCGGAGGAGTCATAATTGTGTCTCACGACGAGCGACTTATCAGGGAAACCAATTGCTCTTTATATGTCATAGAAGATCAAACCATCAACGAATTAGAAGGAGACTTCGATGATTACAGGAAGGAATTATTGGAAAGTTTGGGAGAAGTCATCAACAGTCCCAGTATAGCAGCCAATGCAGCGGTTGCCCAATAATAAATATAATTTTGTCCTTGAAATATGATTTTTTTAAATTCTGCGATCATGATTCGACCTTGTTTTCCAAAAACTTAAAACATATTAAGGCTTGTCACGCACAGGGAGCTATACTATAATATACACACACCTTATATGGAATCATCTGATATTTCTTACTATTTCGTACGAATTTAAAAAAACTAACACACGAAGTCAAACAATATTTATTTTAAAGAAATTTAATAACAAATACATAACAATTAAATAAAACAATAAAGTATTTAACAAACAAATTGAAAGTAGTTGTTTTAAAGTCAATAGCATACAAAATTTCAATGTAAAACGCATAATGTTTAAATTGTTTTTATTTTTTTTTGTATTTTGTGGTAGTCAGTGTTATCACCTCTAGTTCTTATGCAAGATTCAGTTTGCGTGGGCACGCTCCTAATCAAATTATCAACATTTTGTTGTGGTAGGTTGCTCCATCTTTTAAGAGCAGCTCGTACTAGCCGTGCGGTGTTTTGTGGATTATCCCGGCCAGCTCTAATTTTTCTTTTAAGCATATCCCACAAATACTCTATAGGATAAAGCTCGGGTGAGCAAGCAGGCCACTCCAAACAAGGGATACCTTCTGCTTCAATGATGTCTGTAGTCACTCTAATGGTATGTAGAGGTCCATTATCATGCAATAAAATTAAATTTTCTCCTGTTGCACCTCTCCAAAGCCTGACTACAGGTTATCAAAATACCTGTGAACAGTTAAAGTTGATTGGATGAAAATTAAAGGAGTTTTTTTAACGGATCACAATTCCTCTCCAGAACATTACACTTCCCCTTGTATATTTGTGAACAGATCTGACAGTTTTCGTTCTTGCTTGTCTTCCTCGACCTCTAAGTACACGATTTCGTGGGTCATCTGATTTTCCATCTGATTACATTAAATTCTGACTGTTTTTGAAAATAGCACATTTTGTCAATTCCCAATGTTCCAGTTTTGGTGGTGAAGACACCAATTTAGGCGATCAATCTTGTGCTGCCTGGATAACTCGAGAACCYATAAYTGTCACCTGCTGTATACTTCTTSGTACGAACTCTTCTTCTTATCCT

>Dvv ABC-F D802

TCAATTTGTTTATTACTCTGTTTTAAATACTTTTTATCACAATGATGTTCAAGAGGACCTACCAATCACGATTAAAAACTGTCGGTAAATTTTAGCTAATGATGTTAAATGCTAAGAAATGTGCATAATTTTCTAGTGTAAATTAAAAAAAGCCTAATTCTAATATTTTTTTAATAATCAATCCTTTGAAGCCCATAATATAACAATATGCAAATATGCCTCACCGAAAAAAGACTTTAGTTAAATAATCAAAAACTTAATAGCATTACAAAAGATATACATCATCTTCCTAATTAGATGACTAACGGTTTTGAGTGTTATAAAAAAGCTAGCATGTATGGCTTCCAAAGACCGATTTTAGTCGAATTTTATATAAAGCACATAAATAAATGTATATTTAGACAATAATAAACAATCTAATTATTAGTATAGATCAATGAAATACTTGGATTTGAATATCACTTTTGTATTCGTAACATGATTTAATGAAACAACTTATATATGAAACACGTTTATTAAATAAATAAAACCAAGGCCGGCCAATCATTTAATTTCGACATACCATACAATATGCTTAAATAATTTTATGTTAAAAATGAATCTAACGTGATAAAAGAATTACAGTTTCTTCAATAGCTCCTAATAATCTGAAGTATTGCTTTAAATGTTTATCATTATTTCCGTTTTGCCGCATCCTTCAAAATTTTAGTTTTCAAGTGATCTTTGTATGAAAGAATGTCGCCTTGCCACTTCGTTACTGTTCCTTTTTCGCATACCCAAATTTCTTCCGCAACCTGACTGATTAATCTAAAATCGTGACTAACAAGCACCATTCCTCCTTCAAATTCATTAATAGCATCTGCAAGAGCATCGATCGTTTCCATATCCAAGTGATTCGTAGGTTCATCAAGAAGAAGCATATGAGGTACTTGCCATGCTAGCCAAGCGAATACTACCCTGCACCGTTGACCGTCAGATAATTGTCTAATAGGGCATACCTGTTGACGCCCTGTAAGACCATACCTTCCTATGATTTTTCTCATTTCTTCTTTTTCTTTAATATCAGGGAATTCTTTCATCATATATTCAAGCGGCGAGAGGTCCAAGTCTAGCAGTTCGTGTAGATGCTGATGGTACCGGGCAATACGTAAATGAGAGTTTTTCCTAATCATACCTTCTGTTGGAGTAAGATCACCATATAATAATTTCAAGAGAGTACTTTTTCCTGCTCCATTGGGTCCCACCAACGCTAACCTAGTGTCCAGATCTATACCGAATTCCAAATTTTTATAAATCAAAGGTGTAGAATCGTTATATCTAAAACTGACATTTTGTACCATAATTACTGGTGGAGGTATAGTACCACAACTGGGGAAATAAAAAGTGACAATTTTGTCACTAGTCACTTTTTCTGTTAGACCCTGAGCTACCATTTTGGCTAGCGTTTTTTCTTTGGACTGCGCTTGTCTCGCAAGCTTTGCTGATCCGTGACCAAATCGTGCAATATAGTTCTTCATATGGTTGATTTGATCTTGTTCCCAATTATACTGTTTCATTTGATTTTCCAAAAGTTCCATTCTAGTCTTAACAAAGGCGTCATAATTGCCGGTATAATATTTGAGCCTTTTTTTATTTATGTGTATTATGTTTGTGCATACTCCGTTTAAGAAATCTTGCGAATGTGATATCAACACTAATATTCTCTTATAATTTCTTAGTTCCTCTTCTAACCATACACAGGCATCCAAATCCAAATGGTTGGTAGGTTCATCTAATAAGAGAAGATGTGGCTTGACATAAAGAGCTCTAGCTAGTGCAATACGCATTCTCCAACCTCCACTGAAATCTTTAGTCTTCTTATTCTGCATTTCTCTGGTAAAACCAAGACCATGTAGAATATTAGCAGCTCTTGCTTCAGCTGTATCAGCTGCCATATCATCCAATCTCTCGTAGATATCCATTAGTTGTTCCTGAGATTCGTCATCTTCACATGCAACTAGCTCCTCTGCCAACTTTTCCAACCTTACTCTTTCTTCATCTACCTCCATAACACATTCAAGGGCTGTTTTATCAGAAGCAGGCATCTCCCTAGTTAAATGGAAAATATCTATATGATCTGGAATGGGCACTTCTCTGTTACCAAGTACTGCCAAAATTGTGGATTTTCCACTTCCATTTAAACCTAGAAGACCATATCGTCTACCACAGTTCAATTCCAATAAAGCATCTTGTAACATTTCACAACCATGGAAAGTAATGGAAAAAGTATCTATTTTCACATCTCTAGATTTTGGATGCACTGCTAAAGAACCAGTACATGCACGAGCCTCTGCATTCAAACGGGCATCCGCTTCAAGTTTTGCACATAAGGCTTCTTCAGCACTTAATTCACTCGAGCCATTTGTTGCACCATTACTGAGACCATTTGTTTTAAGAGTGTCATTTTGCTCTTCTCCTTTTTGTTTTTCATTCTTTTTTCCTGCTTGTCTGGCTTTTGCTGCATCTTTTTTACGTTGTTGTTCACGTTTCTTAGCGTCGGAGGGCATTGTTATTAATTAATTTGAATACTTTATGCTCGATAATACCTAAAAATAAAATCGGCTCAAAATTACATGTGTCCTCTAATTGAATGGACACAAAAGAGGTCTATCGTCTATGGCTG

>Dvv ABC-F D9935

GTTTTAGGTTAGGGTAACTTGTCACATAAATTTTTATAAGAAAATCTGGATTTTACTATTCTAAACAATGGGGACGTGTAGTGAGTACATTAAAAATGTTTTTCCTGCGATAGATGAAGAATCTAAACAATATGTAGAAGGTGTATTATTAAATGGTGCGGATGATTTTGAGGATAGTGAAGAAGTATATGATGCAGTAGGAGAAGTTCTAAAAGAAATTTCGAACGACAAGTCCGAAGATGATATTAGAAATATCTGCAATGATCTTCTATGCATGTTAAAACCGGATAAAGGAGAAAAGGCTACTAACGGGGCTATGAAAGTTTTAAATGCGCCAGTACATTTGGGGTCAATGGTAGACAATACGGATACAAATATTGATGATGTTAAAAGTATATGGCTTATACAACGGGATGATTCTCTGAAAGTCGATGCAAGAAAACTAGAAAAAGCTGAAGCTAAGCTACAAGAAAAATTAGACAAGAGAACGAAAGAAATAAAGGTTATAGCACCGCCCAAACTACAGACCGCTACAGCATCGCAAGTTACCAGCAAAAAGGACAGCAAACTAGAAGCCAAAGGTACCAATAGGACACAGGACATAAGAATAGAAAACTTCGATGTTGCGTATGGAGATAGGGTTTTGCTTCAAGGAGCTGATCTAACTCTTGCTAGTGGTAGAAGATATGGTCTTGTAGGTAGAAACGGTTTAGGAAAAAGTACTCTCCTAAGGATGATTTCTGGAAGCCAATTAAGAATACCCTCCCACATATCAATTCTTCATGTGGAACAAGAAGTTGTAGGTGATGATACTGTAGCCTTAGATAGTGTACTAGAATGTGATACTGTGCGCGAAGAATTGTTAAAAAAGGAAAAAGAAATTAGTGCTGCAATTAATAGTGGATCGGTTGATCCACAACTAAACAGTCAATTGACAGAAGTGTATAATCAATTACAAAACATAGAAGCCGATAAAGCACCTGCCAGGGCTTCTATTATCTTAAATGGTTTAGGTTTCACGTCCGAAATGCAACAGAACGCCACTAAGACATTTTCTGGAGGGTGGAGGATGAGATTGGCATTAGCTCGTGCTCTTTTCTCTAGACCTGATCTCTTACTTCTTGATGAACCTACTAACATGTTGGACATTAAAGCGATCATCTGGTTAGAAAACTATTTACAAAACTGGCCTACGACGTTACTGGTAGTTTCTCACGATCGCAATTTCTTAGACACGGTGCCTACTGATATTCTACACCTTCACTCTCAAAGGATAGAGGCCTACAGGGGAAATTATGAGCAATTTGAGAAAACAAAGACGGAAAAATTGAAGAACCAACAACGAGAATATGAGGCGCAAATGCAGCAAAGACAGCACGTCCAAGAGTTCATCGATAGGTTTAGGTATAACGCAAACAGAGCCGCATTGGTGCAGTCTAAAATAAAAATGTTGGAAAAACTTCCTGAATTGAAGCCTATTGTGAAAGAAACAGAAGTTGTATTAAGATTACCAGAGACAGAACCATTGTCGCCTCCCATTTTACAACTAGACGAAATATTGTTCCGGTATAACTCCGAACGAGTTATATTTAGCAACGTCAACCTCGGAGCTACCATGGACTCCAGAATATGTATCGTCGGTGACAATGGCGCTGGTAAGACTACCCTTCTCAAAATCATCATGGGAATATTATCTCCGACATCTGGTATGAGAAACGTGCACCGGAATCTTAAATTTGGATATTTTAGTCAGCATCACGTGGATCAGTTAGATATGAACGTCAATTCCGTAGAACTTTTACAACAAACATATCCCGGTAAACCAATCGAAGAATATAGAAGGCAACTGGGCAGTTTTGGTGTTTCCGGTGATTTAGCTCTCCAAACAGTATCGAGTCTCTCTGGAGGTCAAAAATCGAGAGTAGCCTTCGCTACGATGTGCATGGGCCGACCTAACTTCCTAGTTCTCGACGAACCTACGAATCACCTGGACATAGAAACGATAGAAGCCCTCGGCAAAGCCTTAAAGAAGTACACGGGAGGTGTAATTTTAGTATCTCACGACGAACGTCTGATAAGAATGGTCTGCAGCGAATTGTGGGTCTGCGGAAACGGATCGGTCAAAAGCGTGGAGGGAGGTTTCGACGAATACCGCAAGATCGTCGAGCAGGAGTTGGAGGCTGCGGCGCAGTCGAAATAGCATTTCTAAACAAATTATTTATACGGTAATAAAAAAAATAAAAGCACTGTGATTGAGGCATGTTGAAGCTGAGATTGACTGTTCCGG

>Dvv ABC-G D9811

GCGCACTATTTGCGGACTTTGCATACCTATATTATTAATATCTATATGGAATCATAAGATTCGATTCCAGCAATAAAACTGCTGGTAAATAACTTTTCCTTGTATTTTGCTAATTAGCCCAGAGTATTAATGAACATTAGTAAAACACATTTTTAATTACAACTGAACACTGAACAAAATCAGGAAACATCATTTTGAACTAAAGAAATTTGAAAATATACACTGACTTACGCCGCCACAGGTATAAAAAACCATTTTTGTTAAATATTGGGATGTTCCTTGCAGGCAAAATGTTTGCATTTATCGCGTTTCGATGTGAATTTTTCTGTCGGATAATCAACTGCAAATTTTACATCGGCCACGTTACCTACCATTTTCAGTTGGCTCTGTATCTTCAGAATCTTCTGAAAATTTTGTTAGTACCATATCCAAGGCTGTTTGAATGTGTTTATAAAAACCTTGACGCGGTCGGGATTGTATAAAGGGTGTGACTAGTTCAAGGGCCAAATTTTGAAGAAAGAGCCTCCTTGGAAAAGATTTCCTTTTTCGTAGTCTTCATACTTATCTATGTATARGGCAAATGCATTTAGCCGCCTATTACTCCAAAATTACTCAGAATTTCACATAATAGACGGTATTTACTTAATATATAATTGGAACATGGAAGGCCAAGGGCGGTCGTCTTTGACCCCAAATTCAGAAAATATTTTTTATTCGTGAAATATTGGGACCGTGCAGGTTCGGCAAAGCGACACCTGGTTTCTACGCTCAGCAACATTATTCGCACTTTTAATTATATTGGCCAATCATATTGGTCCTGGTTACTGGATAATTGTCAAGGTCATAGTCCAAAAAATAATAAGGAAAAATACGATTCAGGTTATGTTATTAAAACGTAAACTATATGTAATTATATGTATTAAATAAAATTAGTTATTAAAATGCAGTACTACAAGCAAAATACAATTACTTAAATTTACCTTTATATAATAATTGCATATCATATCAATATTGTGGAGCAATATATAATTTTTCTTCTTCAATGACAGTAGGTATGAAATATACGTCAATTTGACAATTTCAATTGACAATATGAATTATTTAAGAAAGTTGCAATATTTCTCCGCTATTCGTGCACGATCGTTTCTCGTATCTCCTCCAGGTACTTGCACACCTTGAATATTGGTTAAACAATATTGTCCTACTGAGATAAAAATTTGTTTATATATTGGATAATATTGTGACTTAAGGTCTTTTGCCTCGGCATATAGCAGCAGATAAAAAAGTGTACAAAATAGTATGTGCCCACATAAGCGCAAATAAAAACACAAAAATGATGATGACAAAGATGATTAACAATTTTTTGGTAACAAGTAAATTTCTATAAAACTCATGAAACTGACTGGGGATCCACTGTAAAAGGATTAGTATGTGATCATTTTTCTTTTGTGTTATACCTTTCGGTTTTTCATAACGTAGTGGTAACTGTTGTGAAAGATTGGTCGCAGTTGCTTTTATTTTTTGTTACTGTCAACAATTTATCAAGTAAAACTCTCTGTCAGTCAAAGTGACTAACACTTGTATGGATATACTGCTGTTTACCTCGCGCGAAATAGCGCGCCCGATAAACAAATCCGACAGCTGATGGAGTTACCACGCTTTACTTCGTTCTCCAAAGGATATACTACTTTTTCTTATAAGAATATAATATAGGCCACCAGTAATACTTCGCATATCATATTTTGCCACAAATAAAATTGGAACATGTTAAGCTTAGCAAAAGTATTAAGTCCAAATTAGAAAAAAATGGAGTTACTACCCTTTATCTCTTCGATACAGATATAGATCTTCTGTCATATTTTCAGTAGTTTGTTTTCTTTTCCTCGATAAAATCATATTATCTTTTACACAGACATAATCTTAAGCTTTGGAATTTCAAAATAAATTTTTACAAAATAAGTTATCAGTATCAATACAATACATCAGTAAATATCAGCAAAAAATCAACATTAATTCAGAAAAAAATCAAAATTCACTCAATTTAAAAAGTGCACAACAGGATTTATAAAAGACCTCGTTAGATACCAAGCCTTATTGAAATATATTCTGATATTTATTAGTCATCCATGGAGCTTAAACATTATGAAAAATAAAGCTGAGTTTAACAATTATGAAAGGCATATGAAATTCTCTTACTGGGCATACAACCCTGGGCAGACAAGTCTTTGTCTTTTGGTGTCATCTACAGTTAAACTTCAATTCCGAACGACTAAATACAGATGACCTACAGATTCAAATTTTTGGGTTTTGTAATAATAGGACCTACATCATTTTTTGTAGTATAGATTGGTACTATATAGACTTCTGGACTATATAGGTGGTTCAAATCTATTACTTATCAAAAATAAAGCATCAGATATGGTTTTGCAGCTATTTCCCACAAGTATTTCATTATTAATTTCACGTAGGTACACCAAACATGCCGACATTAATATTTATATCTATAACAAACAGACAGAACCCGTACAAAGATTCCAGTATTTTGATTGCTCGATAACAAGCGATCTTGCCGTGAGGTCGAAATTCATACAAAGCATACTCATAGGTATAGAGTATGCTAGGCCCGCTTTTCAAACAATGAAAAAATTCCTAATAAACTCTACCATATCGTTGGATATTATGTACCAATGTGTAATGGTTATTCCATACGAATGGTTATTCCACGAAGTGGAAACATGGACTTTCGGAATTATACTAGTATGAGGCGTATGGTGTGGGTTGTTTGACGAATGTTAAAGATCTCTTGGACAGAAGAGCACGTGATCAACAACAAGGTGCTGACAAGAATGGGGACTGAGAGAGAACTCATAAATATTAAAAAAAAACAGAAACGGGAAGAAGAGGTCCAGGAAGAAGAAAATACTCTTGTCCGAAGAACGTAAGAGACTCAACAGGCAAGGACATACATTATATACTAAGAACAGCTCAAAATAGAGAGCAATTTGCTGTAGTTATGTAATAAAGTAATTACCTTCAGTAATGGAGAAGGCACATTAAAATGAAGATTTCATTGAAAGTAGATATGTGGACCATCTTGTAGCAAATACACACTTCTTAATCTTCTTGATCCAGTAAATTAACAATTGTATTGAATATAGTATTCCGATAATAAGTTATTGTAAACAAAATAACTCACTAACTCGTTTGTCGACGCTCGTCGTTCTTTTTTTTGCAAAATCACAGCCACAAGATGAAGCTAGCAAATAATAAAGAAATAAAGTGAAAAATAAACTTAGCATTTTACTGTGACCTAAAACGTGTATCTACTACTATACACTGCGAGGCATAAGAAAATTATGCTCATTTTCATAGTTCATTTTTTCGTGAACAGATTGACGGATCTGTATCTGTATCTGATCTAATCATCTACAAGAACCTGCCTAATACTGCCTTTGAGAAGCCACTGCCCTGAAGAGCGACAATGTTGTCAAGAAAATTGCCCCATAGGGTTGTATGGCCCTATAATTGTACACTGCATCACATAATATTTGAAATACGCTGTAGAGTAAATGGACTGGGCATACGAAAAATCTGTATTCTGTACACACCATTATTTTAAGTAAAGACAATATGCGCAATACATAAAAACATAACAGCTTTCAAGTATATAATAAAAAATACGTATAATTTAACAATAAAGATTGTTGTTTCACAGTTTCTGCGTGGTTTATCATCAAATCTTTTGCATGTTAAAGAATCATCGCTTATTTAATCTCAACCATAAGGTCAAATACGTCACAGCATGCATAACACACCAAACAATAACAATCAATATAAAATTTGAAACTAAATCTACATCTAAAATATCCATTTCCGAGAGGAACTTGTGAGGATCTTTATAATGACAGTATTCCTCCTCTATTGGGCAAACCAAGATCTCTCGGTTAAAGCCGTAAATACTATAAACGACGCTTTGGAATGCTGCTCTAAAGTAACTGATGAAGAAGATCGGTTTGAACATGGTAGAGGTGTCTATTGCTCTGATGCAAAAACCGAAGACTGATAAGAAGCAGGCTAACACTGGGGCGATGAATACAGCTACCTTTACTGGTGTTGTGGCTCCTATAAAATATCCCATGGATTGGCCACAGAGAGAACACGCTGTTACAAAAATGACAAACAAGAATAACCTAAAGTCTAATGGCTGGTTCGTTAACCAATAAGATACTGCTATGTACATCCATGTACATATAACTTGGAAAGGAATTTCAACTAATATCACAGACAATAAATATGGAGTTAGCTTATACCAACGGTTAAAATGCTCTCTAGAGAGTATCTTCATTTCTAGAGGAAATGATAAAGTTACGGGCATTTTACCAGTATATACTGTGAGTAAAAGAGTACCATACAAATATACATAATTTGCTAAAACTGTATCCGCGGCACTTCCAACGTTTCTATACAAATAGCCAAAGATCAGACCAATAACAATGTGGGCCAATACTCTGTTCAGCGATGGACCGTAACATCTCTTTATTATCAGCAGATTCCTCTTATACAATAACAAAAACTGCATAATGATTGCTGCGGGCACTGCTTTCGTTTCGTCGGAATAGAGACCTACTTTTTTTTCCTCGAAGAGATCTCTCCCTTTTAAGTCATCGTTTTCACCACTTTCCATACTTTTATATTGATACGATTTGACACTATCTGCCAATTTGTTAATATTTGCATTGTGATCTCCGATTGATACTTCTATTAAGTAATCAGCTGGATTGTGGTATGGGGGACATTTTAGATTGAGTTTTTCGAAATATGGTACTACATCGCTAACCCTTCCATCATATATGCATTTTCCTTCAGAGAGAGCATAGAGTTTATCGAACATTTCGAATATTAACGCTGAAGGTTGATGAACAGTGGCTATTACGGTTCTTCCTTCTGAGGCGAGGTTTTTGAACAGAGACACGCATTGAGTACACGACAAACTGTCCAGGCCCGTTGTTGGTTCGTCGAGAAACAATATCGGTGGATTACTAAGTAGCTCCAGTGCCACGGCCAACCGTTTCCTTTGTCCACCAGATAATTGTGCTGTTAGTGTATGTTGGCATTCTTCTAAACCCAGCATTTTCAGAATCCCTGCAATCTGTTTGAATTTATAATCGTTAGAAACACGGTAACCTAGTTTTAGGTGAGCAGCAAACGTCATGGCCTCCTTCGCTGTCAAAGCCATTCTAAGTTCTTCATCTTGTGGAATATAAGCTGATAATTTTCTATACCGGGGGCTCTGGTCTCGAACGACATCGTTTAATTTTACTGTTCCGGTGCTTCCTTTGGTTATATATCCTGCTAACACATTAAGAAGAGTGCTCTTTCCTGCACCAGAAGGTCCCATTATTACAGATAGTTCACCTGATTTGAATTGACCGGAGACCCCATGAAGAATTTTCTTCGTTTCTTTCTTGAACTTCGTAATGGACCATGACGTGGAGTAGAAAGTGATATCCTCAAAATTCAAGTCTACTGCCGTCTTCTTGGGGAATACACTGCTCATATTTTCCTGTGTACTTTTAAAACGGTAATTGTTGTTGTAGTTGATGGCTAAGCTCTTGGTGTCTGTCATGTTGACATTTTCTGTCAACGGTATTGACGATGGTGCAGTGTAGTTGTGTGATGGGTTGATTTTGATTTTGTCATCGTCGATATAACGCGTAATTTCCTCAGTTCCAATCATCCTGAAGATGGTAACATTGAACTTCACACAAAAAAAAAATTCACAAAACGTTTGAAATGGATACGTATCGGAGAAGAACACAACAATACTCGCGAGCGCGAGTTCCGGCAGATTCCAATATTTCAACCGTCACACAACAACTGCACGTTGAGACCAAACGAATGAGTCTG

>Dvv ABC-G D3712

CAAATGGATGACGTCACTAGTACGATATATATGTCAAAAAATCATAATTTAAAAATCGAATAACTTTTGTATATTACATTTTTTTTCTAATTCTGTAAATAAAGCAGTTTACAAGGGGGTCAAAATATAGTCAACAACCCCGCACACTCACCGGGGCCGATCGACCGGCTCCGTCCCATCATACAGCCGACCGACCATAATAACCCCTACTTATATTTAATTTAGAATGTATGCACTGACCCCCAGCCCCAGCAAATGGATTTAATCACCTTCGTAGGAAAGCAACCCTGACATCCTCTCAGCAACCCCCGTTCCACGCTTCGCCCGACCGACCGCAGCAYGCCTCTCCACACAATCACAGCAATCAACTGTGAAAAATTCAGCTCCAGCAAACTCAAAGATACTTTTCAGCGCTGCTTCTTGGACTATTAATCTTACAGTAGCGGAATGCTTAAAAACAGGCAAAAAATGGGTTCTGTAGTCACTTATTGGTTAGATCATTATTTGATATATTATGACAACTGACATATAATAGAGCAAAATACATCGTAGCCTGTAGAACCAAAGCAATGAAAGGTTTAAAGAATATTGTTTCCTACGTAAAATAATGAATCAATCAATTTTACATTTTTTTAGACGCTAAACATCATATAAATATAAAAGAAAGTTTACAAACAATAAATTCTGTATAAATCATAATACGTTTAATTTTAGTACGTCATAGAGAATATAAATAGAAGCAGTACTAGGAAAATAGTACGTTTAAAGATGATGAAACGACGGTAACTACTCATCGGCCATTTTTTATTGGAATATCCTTCATACCAGTATCTAGAGCTGAATAAATAAAAGAACACAGGTTTAATTCTAGGCATCACGCTGTTAATGAGTCAGAAGATAAAAAAATAGCTGCTAAGTACAATTCTGGCAAGAACAGCTGTATTTTTGAAATTCAGTATGAGAAATTGATTGTATATAACATAATATTACATTATTATTAAAGATACCAATGTACAACCTGCGTCCTTTCTATATAAAACCACGATTAATAACAATATTTAACATTATTAAATATATCCTAAACTTAATGGTTTGATTTAAGTTTCCACTTCAAGAAGAGGTAAGCGGAAACTCTTAAAAAGAAGAATATCACAACTAGAGCCAAAATGTCGAACCAGTAACTCGATTTATCCATGTCCAACTCTTCGAGAGTTGTGAGTGCCTTTTTAAAGTGGCAATATTGAGCGAAACAATCCAGGTTTGGTCTGTTGTATCCGTATGTGGCAAGCGCAGTCCCTTCGAATCCGTATCTGATGTAGGACAGATACGTTATCCAGCGAAGGTAGATGGGAATAGCATCGAATGATACAAAGAATCCGGAGAACAACAGGAATGGTACTGACATTACTGGAGCTAAGAATACACCGTTCTGAACATTCATAGCAGCTCCTACTACGAGTCCAACACTTTGGGCCACGAATGATACTAACAAGGAAGACAGTAAAAACATTCCAAATCTAGCCGGATCCAAGGGTTGGGAAGTCATGAAATATACTATCGTTACGTAAAGTATACAGAAGATGGTCTGGAATGGCATATCTGATATCGTTATTGCCAGATAATAAGACCTCAGGGAATACCATCGATTAAAATGTTCCTTTAGTAAAACTGGCATTTCAAGTGGGAAGGACAAAATGGTTATAGTCATGGAAGTGTACATCAAGAACAACATGTTGAAAAACAGGAAACCAAGATTACTAAGTACTTTGGACCCATCGTTTCCAATTTTGAAGTATAAGGCACCAATTAGGAATCCAACCAAAATATGGGCGAATAAACGTAGGTACATCAAAGTCCAATCTCTTCTACTGAACAATAACGCACGTTTTAAAATAATGAAAAATTGTTGGAATTCCGAATTACCATATCTGGGCTGCTTAACTACAACGGAAGTATTTAATAAGGCACTATCAACATTAGCTTTTTCGACATCTTTAGAATCTATTCCAGTATCTTCGTTAACGTTGTTTGATTTTCTATTGCCATTCACGGTACTGCCCTCTTTGGACAATTCATCAGAAATAAGGGTTTTCAAGTTATCTTTGGCGTATTGGTAAGCGTTATTCAAACCGTCGCTCATTTTGAAACCTACTATTTGGCTAGCATCTCGGATGTCATTCTTACCATTTTCTATAGCGTTAACTAACGTTCTCGTGTGATCTCCATATTCCCCACATGCTACTTCTATAATATAGGAGGCTGGATTGTGGTATGAAGGACACTGTAACTGCAGACTTGCCAAAAAGGGCACCAAGAATGTCGTAGAACCTTGGTAAACACATTGACCATCAGCGAGTGTATAAAGTTGGTCGAACATTTCAAACAGTCTAGCTGAAGGTTGGTGAATAGTACATATTATAGTTCTTCCACCTTTAGCTAAGGTTTTCAACAGTGATATACATTGAAAACATGATGAAGAATCCAATCCACTAGTAGGTTCATCGAAAAACATAACTGGAGGATTACTAACTAATTCTAAGGCGATTGACAATCTCTTCTTTTGGCCCCCCGACAACCCGCTTGTCATTGTTTTCTTATGATCCAGCAGTCCAAGTGTGTCTAGGATTTCGTTAATGATATCTTCTCGATCTTTTGGACTTTTGGCGCCAATTTTAAGTGCAGCTGCGACAGCCATAGCTTCATCCACTCTTAGATTAGCATGTAGTTGGTTATCCTGCATAATATATGCCGATAATTTTCGGAATTGACTAAGATCTCTATCACTGCCGTTCATTAACACATGGCCTTTTACCCCTATGGTTTTGTATCCAGTGAGTATATTGAGTAAGGTGGATTTTCCAGCACCAGATGGTCCCATAATAGCGCATAGTTCACCGGAACGTAGCATTCCACTGACATTTTTCAAAATCTTTTTCTCTTTGCTCCCTTGCTTAACCACATATTCCAAGTCCGAAAAAGCCAAATCAACTTTGGGTCTCTCAGGTAAATGAACTAACTTTTTTGGTTGATTAGATACGATTTGCAACTTCACATCAGACTTATTTTTTAAGAGTCCTTCGGTGGCCATCTTAAAAAGATTTAAAACAAATAATTTAGAGGACAAAGAAGCGACTACCGTTTAAATAAAATGTTCTAATCCACTAAAACACCACTAGAAAAGTTA

>Dvv ABC-G D14042

CATAAAAATATTCAGTGAAATGACTTTTGGTACTTTGTTATTTAAAAATTGTTTAAATATGGTTTTTACATGGCTTAAAAGAATTGTAAGTGTATTAAATATTAAATTACTAAATATTCGCCATATCTTGATATAAAAAATTTCTACATAAGCTTCAAAATCCCATGTATGGTTCAGATAATGTAGCAGTGACAGAATTTATGATTTTAAACTAGACAATATGGAGATTAAATGTCTAAAACTGAAAGTTTATTTAACACGTTAATGAATTAGGAAATCATTCCCTCACATTGTTTCCTACTTCTTTGTAAAGTACTTGTCAACTCACATTTCTGATCTCTGTTACACCATCTGCTTGATTGGCGCACCAAAATATAACGAACTGTAACAAATATGTATACCATATTCCTACATAATTATAACATATAAGATCACCAGCTCCTTTGAAATACTTGTGTTACTACCTACCCTAACAAATAATAAATTGATCTAAAATAATATAAATACATTGATTTCGTATAAAACTGTGACATTTTTGGTTTACATGAAACTAGATGGGCAAAAATCTCGACATATCACTGGAATTACTATCTTACACTCTTATCCCAGGAAATTTCAAGAAGAATAAGTATACTCTATCTTTTGAGTCATCGTTACAGACGTTGGAAATTATCCATAATCCAACATCAGCGAAAACAAGTTTAAAAAGCCCAACAAAAGGTTTGTTTTGACATAATTGTCCAATATGCTGACAAACAATTGTCTAATATACTGACAAACCAAATTGCACTGACAAAAGCAATAAATGTGTTACTTAAAATATGAAGATTGTTACGTTCTACACTCTGTAGACAAATATTTTGGAATTAAACATAGATTTTTACGATTTTTTATACTATTTCTAAAACTCTACATCAAAGGTTCCAAAATTAAAATAATGTATTACAGTGGGAAACAGTAACCCCATCTACAATTCTAACTATATTAGCTATAGTCAATCTGTAATCATAAGTTAATCTGTTTCTAGAGATCAGATAAATATCACATTAGGCTACTAATAACTTCAACTTTTTTACAAATGTGTTTTTACCTCATCAAAATTCTTACTAATTCTTTTGAGCTTAGCACCAGCTAGACAGTCTTTAATCGATAGATGTAGCTGACCCACTAGTATTCCTTACAGTATTTCCACTAAATTATCACTTTGTATATAATTTTAAATTATATACTGCTTATTTTGTAATAAACACAACAATATATGTCTCAAAAAACAGAGTATACCCACTTGATGCAATACAACAATTTAAACAGCTAAATATTGCCCTTAATTGCTAATGATGTGTTGTCGAAAAGGTACGAATTATTGAAGAAAACATTTAGGAATACAAAATTGTAATATTAAAAATGCAGTTATAAACTTTATTTCCATTGCTATGGTACGGAATACTGTTCCAGTACATACAGAAATAAACAAGACTTAATTGGTCAGTACAATATATAGTTACTTTATTGCCCTAGAATATTTAAACTTTCGAAACAAGTTTTTACGTTATTATAGATCTAGAATATGTCTAGTTTCATTCAATAACTTTTCTTTATCGCTACAACAGCCCTGTTGGTTGTATATCGTTCTGAGGCGGTAATTTAGGTTATGTGAGGTTAACTCTGCAATAATTTCAACAGAGGCAATAAAGTATCTATTTGACAAGTGCCTAGTAACCTCAAAGTCCCATCCTATTACATAAATTCTCTATAAAAAGAAAATTTTACCGATTTTGCATCAATTTAATGCGAAGCATAAAGTACGCTATCACCCTAAGAAAGATGAACAGTCCTCCCAGCACCCCGACGTCGATGAAATAGCTGACCATGTCGTCTTTCATGGACATCTGCTCCAAGAACTTCTTGGGGTACTTAAAGTGGCAGTACTCGATGTTGCACGTCAGCTTCGGTCTATCCAAGCCGTAGATGGCGATCATACACGCTTCGAAGCCGTATTTCAGGTAACTCAGGTATGGCAGCCATCTCAGGTAGAAGGGAATGTCGTTCAGGTTTGTGAAGAAGCCTGAGAAGAGCACCATGGGAATGGTGGAGATTGGTCCGAGGAATACACCTCCCTCAATATTAAACGCAGCACCTATGAGCAATCCGAAGCTTTGAGATACAAGGGCAGTTAACACTGTAACTAACAATATCATCCCAAATCTAGTGACATCTAACGGTTGGGATGTTATAAAGTAAACACCTATTATATAGCACAACGTCATAACCACCTGAAACGGTATGTCCGCCAATGTTTTAGCCATATAGTACGCTTTGAGCGAGTACCAATAGTTCAGGTGCTCCCTTACGGTAACTGACATTTCCAAAGGAAATGTTAAAATTGTGGGCATCATAGCTGTGTACATCATAAACATGACGCAGAAGAAGAGGCACCCTGCGTTACTGGTAACTTTGGCAGCATCTTGTCCTATATCATAGTAAATTAGGCCTATAAGACATCCTATCACAAAGTGGGACACCAGTCTCATCCTAGTTAGCGTTTTGTCCATTAGAATCATGTACATACTTCTTTTGAGCAATATTGTGAACTGTTGGAGACCTGTCGTTGAAAAACCATTCTTTTCCGTTGGCGACAAATTCTCCGAAGAATCTAAAAGTGACGTTGTGCAAGTGACTGGCGTTGTGGGGGTGGTAGGCTTCACAGAACCATTTGGCACGGATATTACATCTCCCGAGGAGTTCTTCCCGTTGGCTTCTTTAGCAATATCATTGGAAACTATCTTACTACTTCTATGATCTGGCGTGGCGAATTTGGTGCAACGACCAGCGTTTACTGCCACCACCAGTTTCTGAACGTAGTCTCCATGCTCTCCACATGCTACCTCCATAACGTAATCAGCAGGATTATGATAACTGGGACAATTCAAGCCCATGCTTGACAAAAATGGCACCAATCCTAGCACAGGCCCTCTATAAATACACTGTCCTTCCGCCATCATGTAAAGATGATCGAACATTTCAAATAACCTAGCTGATGGTTGATGTATAGTGCATATTATTGTTCGACCTCCTCGTGCGAGTGACTTGAGTAGGCAGAGGCACTGGAAACATGATGAACTGTCAAGACCACTCGTTGGTTCGTCGAAAAACATTACTGGTGGGTTATTTACTAATTCCAAACCTATTGACAAACGTTTCCGTTGACCCCCCGAGAGATTGGAGGAGTTTATATCGATGCAAGTTTGAAGTCCTAAATTTTCAATAATTTCGTTGATCACAACTTTCTTCTCGGACAGTGTGACAGTCTTTCCTAATTTGAGATTCGCTGATACCATCATGGCCTCTTTGACGGTGAGATGTGGGGAAAGACAGTCGTCCTGCATGATGTAACAGGACATCTTCCTGAAGCGTCGCAGGTTGCGCTCTTTGCCGTTGATCATAACCTGCCCACTCAGATTTGACGTTTTGTAGCCAGCTAAAATATTCATAATGGTACTCTTTCCGGCCCCTGATGGTCCCATAATGGCCGTCAACTCTCCCGACTTGCATTTTCCACTGATGCATTTCAATATCGTTTTATACCCTCTTTTCCTTCCTTCGGATACCGAGTAGGAGAGATCTGTGAAGGTAATGTCCACGGGCGGCCTTTTGGGTAGGTGTGTGAGGGCAATCATTGGCCTTTTATGGTTGCTCGGACTGCTGTTGGGGACCTTACGGAGCGCACCCGCTCCGCTGATGGTTATTGGTGAGGAACCATTGTATATGTTGGTTTGGGAGCCACTTAAAACAGAAGCATTATTAAGAGATTCGGTTTCTTTCAGAAGCGGGGGAGCGTGAACAGTCTTGGTGTAAGCAACAGCTTGGTTTGTGTCTTCGGGATCAAGAGGAACATTTATCTTTATACATTTCGAATCGTCGGCCATCTTTTGCGCTTTGTCATAGATACCTCACACGGTAGCAACGTCGATGATGTTCGTCGGTTTTTCTCATCAATTTTAACTAGGATAATTAGGAGTCATTTTTCATCAAATAATAAGGACACTAACCCCCAGCACTAGCACTCAACATCTACCAAATAATTCGACCAAATCACAGAAAATAACAAAAATTAGCTAAGAGTTCAAGAAATTAACATTGTTAGAACAACTGTCAACAATCAGCTGTTCTGAACATCTATGACGTTT

>Dvv ABC-G D10897

GCTTGAGTCAACCACAATTCCGATAATCGAAATCTCATTTCGACAGGGTAAATGCTGTCGAAGTGATTTCTATTCACTATTACGATTGTAGAGATTCCGAAGTAGTTATGGAATACGGATTTGGACTATTTATATTCGACTTGGCCACTTCTATTCGATTTTACTGACTTCTATCATGGAAATGAGTTACAGAATAGGCGTGATTGACTCCTCTAAGAAATGCAACCCTAAATGTAACATTTGAATGGAGTACCTACATACCATAACGGATAATATCATAACGTAAGTATGAAATTATATTTCTTATTTCACTTTTAATTCCACTAGGTACTACCAAAATTGTATGAGAGTTCATGATTTTTACAGAATTGGCTCATTAATAGGTATAACCACTGTACTGAGTGACGATCTCATACCTTTATCTTTTAATAAATCGTAATCTTTGGAAAATTTTAAAAATATTATTGTAAATTTTCTCTTAGTTCCTTTGATTGTCACAAAAAATGATGTTTTCTAAAGAAAAAAAAATATTTTTTTGAGTAAAATTTAAATAAATGTCCTTCTAACAACACTGTTTACCAAAACCGAGGCTTCAATCACCTACTTCTAATAAAAGTTATTAAACCATAAACATGCTCTATCGCACAGATGCCAGTTTGTACCTCAGCACTACGTACGCCGCAATTCTCAAAACAAACAAGAAAAGCAGTAAAGCAATGATGTCATTATCGAACTGATCCGACCTGACATCTACTATTTCCAGGAAAGTCTTCGGATACTTGTAGAAGCAAAATTTGTCTTCGGGACATTGAAGAATACCCCTGTCCATTCCGTACACCGCCTGGACTACTCCTTCCAAGCCGTACCTTAAGTAAGAAGTAAAACTGCCGTAGTACATTATGGTCGGCAGATCACGTAGACGAACGCCAAAACCGGAGAACATCATCATGGGTACCATCAGGGTTGGTCCAACAAAAGTTCCGTTCACAACATCAAAATATGCGCCAATAGTTAGACCAACTCCTTGAGCTACTAGAACTACCAACATGCAAGTGACGAAGAATATCAGGAATCTGGCTTTATCGTTTGGTTGATCTGTCATATAGTATATTATGAGTGAAAACAGGAAGCAGCCAATAACCGAAACCGGAATATCCACTATGGTTACCGATGTGTAATACATTTTTAATGAATACCAACGGTTGAAGTGTTCTTTTATTAAAATCGACATTTCCTGGGGAAATGTTAAAATCGTCAACATCATTGTGGACATCATATGGTGCAATAATATCGCAAACAATAAATTAAAATTATCCAAAACTTTAGATCCATCACTTCCAGCTTTCCAATAGAGGCTGCCCAACATCAGTCCAACTATTATGTTTGTTCCAATACGAAGGTAGGTAAGCATCTTATCTCTGTAGGCTTTAATAAATCCCCGTCTCATAAGAACTCCTAGTTGATGGAATTGTGATGTGGGAAAAGAGGATHCATCACTGCTARCAACTCGTTTAATAGGTTTGGGACCTGGGTAGTTACAAATGGTGCTTTTCATCTTTTCAGGCTCGTTAAAATATTGGTAAGATTGTCCGTTCTGAATTCTTTCCCTCATATATTCAGGTTTCTCAGGTCCGTGCTCAATACATGCTAGTTCTATTACGTAGTCAGCAGGATTGTGATACTGCGGACAGGGAAACCCACAGTCTTGCAAAAATGGAACCAGTTTATTCGTACTACCTTGATAAAGGCAGTACCCATTTCCTACTACGTACACCTGATCAAACATTGCAAATAAAGTAGCTGAAGGCTGATGAATGGTACATATGATTGTTTTTCCTTGCATAGTTAGTTTCTTAAGTAGTGTTATACATGTTGAACAAGACGAACTATCCAATCCCGTGGTTGGTTCATCCAAGAACATGACCATTGGATTATTTACTAACTCTAAAGCTATTGACAACCTCTTTTTCTGTCCACCGGAAAGACCAGCTGCTCTCACTTTTTTTGTATTTTCAAGATTTAGTGTTTTTAAAATATTGTTAATTATACTCCGTTTATTACTGTTGCTGACACTGGAAGGTAGTTTCAAATCTGCAGCTACCCACATATTTTCGTCCACAGTGAGTAAAGGCTGCAGACGGTCATCTTGCGTAATGTAACATGACGTTTTCCTGAATTCCTTTAAACTTCTTGGTTGGCCGTTAACGTATACTGAACCTGTTACGCCACGTATCCTATACCCACTCAAAACATCCAATAATGTACTTTTGCCTGCACCCGATGGCCCCATTATGGCTATCAGCTGCCCGGGCTGGAAACGTCCTCCGACTTGATGCAAAATTTCTTTTGTAGTCCTTTTGGTAAATATATTCCCTTGCGTCGCACGAAAACTAATATTTTGAAACTCCAAGTCCACCGTATTTTGGCACATTTTGCCATTAGTACCATCGTCACAAATCAAAGTCTTTTCTTGGTTAAGATTCACCTTTACGGAAAAATTCACTGAATCGCCGTTGACCCCGTTCGATTTGACAACGGCGGCGGCGTCTTTAGATTGCCCTACAAAAGTACTCTCGTGCAGAACTGCCTCCATGGTGCGGATTGAAATTCGCGGACTGTCAGCGACTGAACCAGTAAGTACTCTCGAAGATTGTGATTGCCTATCTATTCGTATTTGTG

>Dvv ABC-G D22358

GTGATGTTGGGTTAATCCATAATATTTTGAGTCCACGAATCTACAGCTAAAACATTTCCAATTAATAATGAAACACCCTATATTGTTTTGTTTTATTTCATTATCTTTTATTTTGTAGTAATATTGATGATTTTTGTAATTTCAGTAAACTGTATTTGTTATTGTTTTTTTCCTAATCTTTGTAAGCTTTGTCCATAAAATTGTAAAATTTTTAGTGATAATAAAGCATATTTCTATTCTATTATATAGAAGCGTTTTTGCTTCTTTTGTTGAAATTTTTTTTAGCACTATGCCATATCAGTGGTGGAAAAAGGCCCGCGACATACTTTGATATGGGGCACCTGTGGAGCTAGCTACTGAAAGGACTATGAAACAAAATCAGCTCTTCAATTTTTCCATAGAATTTTTTAAAGTTAGGTGAAAATGCAATGCTTCCACTCAACCCCGTATAATTCCATCCAAGCAAAAAATTTTGTTACCGGAGTAATAGCAAACGGTACCAATACACGTACCCACAAACCGATGTAAGAATCTTGAAAATCGGAGCACAAATAACAAAGTTACAAGTTGTCAAACTTAATCAAAAACCCTTAGTGGCGGAACCTTGTGCCGATGAGTGTATATAAAAATAATTCTGACTACAATATTTTACAAAGTTATAACAATGATCCTTTAAAAATATCACTTAAATTAATAGACAGTTACCCAAGTTCCTAATATCCTATATAATAATATCACTTAAAATATAATAAGATTAACTAAAGACATAAACCAACTTTCGCTTATCATCGCGCTAGTCCAAACTGTGATTTAACCAATCTTCCAATATATTGTAGAGCTCTAAAGGTTTTGTTAGGAGTTAATCTTTGTCGTAATAGATAATAACTACCACCCCTGAAGAGGATTAAAATAAAAATTAGCACYAAAACGTCAACCCAATAAATAGTGTTTTCCATGCCCATGTCCCTTACGAATAAGTTGAGGTCTGTGTATATACAGTATTCTTCAGTATCCGGACAGCTTAGCTTTTCTCTATCTTTTAACATGGCGTGAATCAACCCTTCGAGCGAGTACCTGAGGTAGCTAAAGTGCATGAAGAATTTTATTATAGATGGAATAGTGTCGTAACCAGATCCGAATCCGTAGACAGCTAGTAGCATGAAAGGAACAGCAGATACTGGTCCCATAAACACAGCATTCACAACTTTCAACTGAGCAGAAATCAGAAGTCCAAGACTTTCGGAAATTACACTGGTCAAAATGCAAATGAAAAAGAACATTGACATCCTCCGAAACTCTAACGGTTGGTCAGTTAAAAGGTAAACACACGATACATACACAACTCCAAGACAAAGTTGTACAGGCACGGTCGAAAAAGACAATGCTGAGAAATATGCACTAAGTCGGTACCATTTGTTAAAATGTTCTCTCTTCACTAACTGAATTTCTTGAGGAAATTGCAGTAGAATTGGCATCATTGGAATATACATGAAAAATATAATGCAGCAGTAATAAAAACCGAAATTAAAGATGGTTTTGGACCCATCCTGACCCATTTTATAATATAGTGATCCTATTAGAAGAGCAAGGATTATATATAGGACAGTCCTCATTATTAAGTAAGTTTTATCCCTCCACATCTGCATCCATAACCGGGTGGTTAAGATCCAGAATTGGCTAAACCATGAAGACTCGTGATTAAAATCTGAAGAACTTAAATCCGCGTAAACTTCTTCTCTTATTGATATTGATGAAATACTGCTTCGATTTTCTAGACTATCGTTGGCAAGTTCGACTTCTTGTAGTTGTGGTAAGTTACCGTTAGTTCTATAAATATTACGACCATTGTCTATCACAGATACCATCCGTTCTTGGAAATTACCATATTCGTTACAGCAAACTTCTATAATAAAATCTGCTGGGTTATAGGTTTTAGGACATTCAATTCCTACTTTACTTAAATAGGATACTACTTCGGGTCCGTAACCTTGATACACGCATTGTCCGTCAGATAAAATGTAGACGTTGTCAAATTGTGAAAATATTTTGGCACTTGGTGTATGTATAGAGCAAATCACAGTTCGACCACCTTCGGCGATCTTTTTCAGTAATGAAATACACTGGGAACATGACAGATCATCTAAACCCGTAGTTGGCTCGTCCAGAAATAGAACAGGAGGATTGTTTAATAGTTCCAAAGCTATCGATAACCTTTTTCGTTCTCCACCGGATAACCTACTTGTACCAGTCCTTTTTGCTTTGTCTAATCGTAGTAAACTTAAAATTTCATCTATCGCTGAAGATTTATCGCTTAGACTCAACGTGTTTCCTAACTTTAAATTTGCTGCTATCATCATCGCTTCTTCCACAGTCAACAATGGTTGGATCATATCCTCTTGCATAATATACCTAGACATTTTCCTAAATTGTTTTAAATTTCTAGTCGCTCCATTTATCAAGATTTGCCCTGTCGCTCCCTGTGTTTTATATCCTGCTAGTATATTTAACAATGTGCTCTTTCCAGCTCCAGAAGGACCCATAATTGCTGTCAATTGTCCAGCCTGGAAACTTCCATTAACGCTTCTTAAAATTAATTTGGATCCTTTTCTTCCTTGGGGCACAGTGTAACTGACATCGTGAAATTCGATATCGACAGGAGGTCTTTTCGGCAAATGCCTCAGGGATGTGTTCCTAGAATGCAATTCCATTTTAATATATCACTAGCACTAAGTCATCTAACAAAAAACGTCAAAAACCGTACAGAAATCGAGAGGTAAACAAACGTAACGAAGTATTTAAAAGTATCGACTAGACTAGCACACGCGTTGCGCAGTTCACGTTTCAGTACAACATACGTCTATGCACATCTGTT

>Dvv ABC-G D23081

CGAGTATTTACGTTAGGATGCAATGTCAACATTCAGGTGCCACAACATTGCAATACGCTGCATCCTTTATGAAGAAAAATCAGCAAAATTTTATTTGATTTTGAGTGATTTGGTTCATAAATCCTTTCAATAAAATAAATTATTTATTTTGGCGAGTTTCCTCGTTTTAATATATTTTTCTGTGATAATTCGGGATGGAGCTTCAACAATTCGCGTCGAATAACTTAAGAATTAGTGATAAAATTCTAGATGTAGAATTCCAGGATATTTCGTTCGTTACCAAGGAGAAAAATGGTTATAAAAAAATTATTGATGGAGTCTCAGGAAAATTTCATTGTGGACACCTTACAGCCATTATGGGTCCCTCAGGAGCTGGCAAAACCTCGTTGCTTAATATTCTCACAGGATACCAGGTCACTGGAACTACTGGTACCATAAAATGCAATAGCTCTAGTAGAAAGCAAAAAGGCGTCTTACAATACAAAAAAGAATCATGTTACATCCTCCAAGATGATAGCCTACCAAATTTGTTTACAGTTGAGGAGTGTATGATGATTGCCAGTAAACTTAAAATTGCCAATATGGCCAAAAAAGCAAGGGAATTTTTGATTAACGAAATCCTAACCAATCTAAGCTTATTAAAGGCAAAGAATACTAGATGCCAATCTCTATCTGGTGGTCAAAAGAAAAGGTTATCAATAGCTTTGGAATTAGTTGATAATCCACCGATTCTATTTTTGGACGAACCAACTACAGGTCTTGACAGCGCATCCACCACCCAATGTGTAGATTTGTTAAAAAAATTAGCCAATGGAGGAAGAATTGTAATCTGCACTATTCATCAACCCAACACCCAAACTTATGAAATGTTCGATCAGGTTTATATGTTGGCCAAAGGTCGATGTGTTTACCAAGGGCCAAGTACCAATACTGTTCCTTTTTTAGCTTCAGTAGGGTTGCACTGTCCTCAATACCACAACCCTGCGGATTATATTATGGAAGTCGTTAGTGGAGAATATGGTGATCATATAGATCAATTAGCAGTAGCAGCACAGGATAAAAAGTGGCAAAATATACCTACAATTAAACTTAGTGATACTCCAGCTATAGACAGTAAAGATAATAATATCATATACTCCGACGAAAACGTTACTCTCTCTAAATCACCTTCAGAATGGAAAAGGTTTTTTATATTATTACAACGAAGTTCTGTTCAACTTTATAGAGATTGGACGATATCTCAACTAAAACTGGTACTTCATTTGTTAGTAGGATTATTTTTAGGTATAACTTTCCAAAATTGTGGTAGGGATGCCACAAAAGTGATAAGTAATTTAGGATTTTTACAAGTCGGTATAGTTTATTTGGCTTATACGTCAATGATGCCTGCAGTATTAAAATTTCCTACCGAATTAGTAATATTGAAGAAAGAAAGTTTCAATAATTGGTACAAACTGACAACGTATTACGCTGCCTTTTTGGTTTTCGACATACCTCAACAGATGTTATTTAGCACAGTTTACTGTATTGGATGCTATTTTGTCAGTGACCAACCATTGGAGGTTGAYCGGTTTTTCAGTGTACTTTTTGTTCTAGTGCTAGCATCATTGTCCTCGTCAGGATTTGGACTGATATTGGGAACAATCACTAATCCAATTAACGGAGTATTTTTTGGAGCAGTGGGATTATGCTTCTTTATCTCAGTTGGAGGATTTTTCATAATGTTCACGCATATGTCGAATGTAATGTACTTATTTTCGTATATTTCGTATATCAGTTTCTCAGTAGAAGGAGTGATGCAAGCTATATATGGATACGGCAGAGGCCAGCTTCATTGTCCAGAAGAAGCAGAGTTCTGTCAGTACGTTTCATCAGAGGTGCTTCTCGAAGATATCGGAATGAGTAAACCTAACTATTGGATAGACATTATCTACCTTACTTGCACATTCCTAACTTTCAGAACCATCGCCTTTGTAACACTTAAAAGAAAATTAGCTAATCCGTAATAGCTAGAAAAAGGAGTTATATTTTTAAGCTTATGTTAAGTAGGTCGGAATATTGGGAAAAATACACAATATAATTATGTAGGACTGTGTCTTATGGTATGGTTGGAAAAATGAGCTGCTTATATAACATTAAAAAATATTACCAATTAACAACATAACTAAATAATTTAAGTGGTAGTTGGGATAATCCGCTATATGCAGCGCGGATAATCCGGAGTGTGGATTATTCGCTCCATGAGTTTTCCATTACTCAAATGAATTTTTGACGTTTTATTGATAGAGCGTCACCGTAAATCACTCGACGGAAACTCTATAATACCGCATTTACACTCAGTCCTGTTGGACAGGGAATTGGGCAGGGAAGTGGGCAGCGTGAATGTGTAAATAGCTCACTTACGCTGCTGCTGCTCATACTACTGCYATTGCACGGCGCTCCCTCAAAATTCGGTTTTAGAGATGGACGTCAAAGGACTGGCAGCGCGAGCGCGAGTGGGTATTCACATGCAACTACTCTCTCTCTCACTTGCCGCCGGCAAAGCGGCAAGTGAGTAAAGGAGCAAGACGATGCTGTCGTCACATTTCATTCTTGAGATTCG

>Dvv ABC-G D13051

TTTTTTTTTAACTTCAAACATATTTACTATACACATAAAAATTATAAATAGTACACTTACGCTGAGAGTATATTATTATAAATATTAGTAGTACATCTAATACTGATACAAATTCGGTACTAAACTTTTGGTCCCAGAGTAGTTCAGTACTAAAAGTACTGAACATGGGTATTTGAATCTCAGACATTTATTATCTAATATACCTACTAATAACCATAAAAACCCGTATAATTAATATTATTGTTTGGCATAATGTGGCATAAAATGTATGAAATATAGTTACTATTGAAAATAGATTTATTATAAAAAGTAAAATATCCATTATTTTGTTAACATAATATTTATGCTGACAATAACTAATAAAATTTAACAAAAATACGAAAAGGAGTACAGTCAATGCCCCAAGACAATGTTTTATTAGTATAGTACAAACGGAATTCGGTCAGAATCTGCAGCCATTATTTAAGCTTTTAATCCCTGAGTCATATTACCGAAAACAGCGATGAATGGAAATATAAAGGTTATGAATTATAAACTCACAATAAGTTTTACGAACGTAATTGTTCAACAGACGAGGATTTACGAAGAAAAAATAACGTTTAGTCTGCTAATCACTTTACCCGAATTGAAAACATTGCGTTCTTCACACAATGTTGCCAAACTGCATTTTTTTATTTTCGGTTTAAATTATCTAGCCGAATTACGAGTCTACTATACACCTTCTTACACCATCACGGCAATCACAATTTCATTCCTTATTAACTTATAGGAAAATAAATAGACCTACAGTTTATAAGCCCATTCATGTTTCTTTCTGCTTGACAATTTTCGCCGCATAGTACACCAACTTGTTTCTTAGTTCGGAAGTCATTCGGTACTTCAATGATATGTACGCTATGATTCTAAACAGGACAGTGTAACCCAATATGTAAGCAAACTGATGGAGTGGTACATTTTGGCTCATTCCCATGTCTTCTAATAACTTTTCTGGATTCTTGTAGTGACAATATATGTCGTTGCAATCCATTTCTGCTCTGTCGTTTAAAAGCGTCGATGATATTCCTACCAGGCCATATCGTACATAACTTAAAGACATGAATGCTTTCATCACTGGTTCGATGCCATCTTTATAACCCATTCCGTACACGGACAAAGCCAAGAGAATAGCTAACCAGCATGGAGCAACCACAGATCCGCTCAGTATACCACAATTGGATCCAATTGCATATCCTAATCCCTGAGAACAAAATCCGACGTTGATGGCGATCATAGTAAACATTAAGAATCGTTGCCATTCTATTGGTTGACCCGTCATGAAAAATACTATTGATATGAATATCATTCCATAACCAACCAACATTGGAATGTTTACTATCGTACTAGCAAGAAAGTACGGCTTTAAGCTAAACCATCGATTAAAATACTCTCGTTTTAACAGTTTGACTTCTATCGGAAACAGTAACACTGGCACCATGACATGAGTATACACGAAAAACACGTTAATCGTTACACAATACTTAAATATTGGTAGAACTTGACTGGCTTCATTTCCAATCTGGTAAAATATTCCACCGACTAACGTTGCTGAAATGACGTGATGGAAAAATTGTATGATCCACATGGATTTATTTCTTTTCATCTGTAAAGCCATGCGACACAAAAGCACGGTGAATTGTGTCCAGAAAGACGTTGGGTATTCTATGTCGTGAATATGCATACCTGTTTGTGTGGTTTCCTGGTAAATTTCATATATTCCTAATGTCTTATGACTTTGTAGAGGTTTTAACTTTTTATCTTTCATATTTATTTTACCGTTTTGAATTTGGTTTTGCAGTATTGGAATATTGTCTTGATTGGTTTGGATTACTTCTATAATAAAATCAGCTGGTGTTGAGGTTTCTGGACATACACAATTTACCGACGACATAAAAGGTACTAATTGATTTGGCGACCCGTTATAAACACAATATCCATTAGCCATAATATATACTTGATCAAAAATTTGGAAGAGCGATGCTGGCGGCTGATGGATTGTACATATCACAGTTCTTTCGAGCCTAGTTATTTTCTTAAGAAGATCTATACATTGTTTAATAGATACATTATCCAATCCCGTTGTGGGCTCATCTAAAAATATTACAGGGGGGTTATTAACTAATTCTAAAGCTACACTCAGTCGTTTTCTTTGTCCACCGGAAAGGTATTCAGTTTTGGTATCGTAACATTTTTCTAGACCAAGAAGTTGGATCACTTCATGTACCACAGCCACTTTTTGTGTATGCCCTATCGACGCGCTCAATTTAAGATTAGCTGCTACCATCATTGATTCTCTGACACTCAACCTCGGTTGCACTAGATCTTCTTGCATGATGTACGACGAGAGCTTGGTGAAGATTCTCATATCTCGAGGCTTTCCGTTGATTTTTACACTACCTTTAACTCCCGCCGTCATATATCCCGCCAAAATATTAAGTAACGTTGATTTTCCTGCGCCAGAGGGACCAAGAATGGCAGTTAGTTCTCCAGATCTAAACTTTCCATTGATCGATTTTAATAATTGCCTCCATCCCCCTTTATAACTGGAATCAGAAATCGAGTAAGTTAAATCACAGAATTCAACATCAATGGGTGGTTTTTTAGCGATTGTATTTAACTGAGTTAAAGGTTTAAATTTCGGCAACAAGACCTCCATTGCACTACCACTGGCTTCACTCATGTTTGTCAATTAAACTACTGCCGTTTTGACTCGAACACGCAACTCAACAATAAATCCTATGGTTCTGGGAAACCGAACTGATTTGCTGCAATCGACCAACTAAATAGTTTCTTAGTTCGCGCGGAGGCAGTTTATAAATCAACTGGCAAGCTGTAAGGAACGTTTCAGAACTACGTATAGAGCAGAAGGTCGATAAATGCCGCGTGCTATCCGTTGGTAATGGAAGCCCATTCGTTTCGACGGTTTTTTG

>Dvv ABC-G T38769_c0_seq1

ACTCCTATCGTATAGCTGGCATTTTCCATGTCTTTGTCTTTAATAAACTGCTCAGGATATACGTAGAGACAGAAATCCTTGGACTCGCAGGGTAGTTTTCCTCTGTCATAGCCGAATATCGTTAGTACCAACCCTTCAAGCGCATATCTGGGGAAAGAAATGTCAAACATCCATTTAAGTTGGTGCGGACAATCACGAAGTTGAACGAAGTATCCGGAAAACATGATGAATGGTAGAAGGAAGAAAGGACCTATTACTACGCCGTTCGTTACACTCAAACAGGAGCCCACAACCAATGCCCAACTTTGGGAGATAACCGAGACCAAAATGCATATAAACAAAAACGAAGATACTCTACTCCATTCCATAGGTTGTTGTGTACATAAATAGGTTATTGTGGCATACAAAACGGTAGCCAAAATTTGGACGGTTATATCTGAAATAGATACTGCTGCATAATACGATCGTATGGCATACCATTTATTAAAGTGTTCCTTAATTATTATAGGTAATTCTGAAGGAAATGTGGTAACTACACAGTTGAACGCCGTCATCATAAGGAACATAACCGTAAAAAACATAAAACTAAAATTGTCATTAATATTAGCGGCATCCAGCCCAACACCGAAGTACAAAATTCCTAAAAATAGCGCTATGGCAGAATGAGTCCCCAATCTGAAGTACGTCAAAGTTCTGTCTCTAGAGAGCATTAGAAAAGATCTCTTCGTCAGCACCACAAACTGGTTTATCGTTGAAGTTGGATAAGAATTTAAGCCGCACTCATCCCAAGTATATTCGATTTCGTTTACGATTGTTCTTTTTGTTGCTGCAAGAAAATCTTCTCCAATATTGGGTTCTTTTATTGAATTTAGTTTCATTGTACTAGCACTTAATTTCGCATAACTTTCATTGATTCCATTATTCGACTTAGTTGAGAGAGCATGTGTAAAATCCCCATAATCTCCTGAAGCTACTTCCAATAAGAAGTCAGCTGGATTATGTTGAGTAGGGCAAATCAAATCAAAATCTTCAAAATATGGTAAGAGATTATTCGGTGATCCTTGGTATATACATTTACCACTTGATACAACATACAGGTGATCGAACAATTTAAATATCGTGGCACTGGGTTGATGAATCGTACAAATAATGGTTTTACCACTTTCCGACATTTCTTTCAGCAATTTAACAAGACGAATTGAATTTAAACTATCTAATCCACTAGTAGGTTCATCAAAAAACATAACTTGTGGATCTCTAAGCAGCTCTAATGCTACAGAAAGACGTTTCTTCTGTCCCCCAGATAGGGCGTCTGTCCTGGTGTGTTTTATTTCCCAAAGAGTGACGGATTTCAAAATGGATGCTATCTTTTTATCTTTGTCAGTCTTAGAGGCCGTAAGCATTAAATCAGCAGCCACTCTCATGGATTCTAATACCGTTAGTAAAGGTTGGAGATCATCATTTTGCATTATATAACACGATTGTTTCCTAAAAGCAACATTGTTTCTTGGTTCATCATTGACTAAAACTTCACCCAGTATGCCTGTAGTCGCATAGCCTGATAAAATATTCATTAGACATGTCTTTCCAGCACCTGAAGGTCCCATTATAGCCGTCAATTGCCCTCCTGGAAAATTTCCTGTGATATCATCCAGCAGTTTCCTTTTCGTTCTTGAAAATAATATTCCGTCATAAACACTGTAAGATATATGTCTAAATCCTACATTTATTAGAGACGCTGGATCATTAACTTGATTTTCTGGTAGTTCCAAAGAATGCAAACGGAAGTCTTCGTCATGTATCTTATTGCTCTCTTGAGGTGGATCCACAGTTCCTAAGATATCACAAACTGTATTTTCACCCATTTTAGTTCTGTTGCATCGTTTTTATTGAATATTCTAGCACAATCCACAAACTGTTCAAATATACATCGACACAATCATTGAATTTGTCTATCCAACCGGCAGGTAC

>Dvv ABC-G DvvW

AAAAAGACATTCGTATTTTGTGTTACGTGAAACGTCTGATATACATGTATGTTGTATTTTTTGTAATCGTTTTATACATATACTCGTATATTTTATCTGTGAATTTTTGTCTATTATGGCTGTTTACAATCGTGGAGGCAGGGAAGAAGAATCCTCAGCCTCAGAATCGTTATCGTTAGCAGCTAGGCTAGGTTTAAGAGTAGTTAGAGATTATGGGACGGATTTTCGCTCAGTGGGAAGAGTTATTCCAATTGAGGAGCGGATGAAATTTACCTGGAACGATATAAATGTATTCGCAATGACCAAAACCATCAAAAATAAATTTTGTTGTTTCGGATTTATGTCTCATGGTTATGATGAACACATATTAAAAAGTGTTGACGGAGTAGCGTGCTCAGGAGAGTTATTAGCCATATTGGGCTCAAGTGGGTCTGGAAAGACCACTCTTTTAAATGCTCTAACATATCTACCAATGGATGGAATGGTCGTTTCAGGAATGCGGTGTATAAATGGTGTGCCCGTGGATGGTCAGAGGTTAAGAAACGTGTCAGCTTTTGTTCAGCAGGATGATTGTTTTATAACAACCTTGACAGTTCGAGAACATTTGGTTTTTCAGGCTCTGGTAAGAATGGAACGCGGTGTGTCATATAATCAAAGACTGATAAGAGTTGATGAAGTATTGTCTGAATTGCTGCTTAAAAAATGCGAAAATACCATAATTGGCTCTACAGATAAAGGAGGCATCTCAGGAGGAGAGAGAAAGCGGCTGACGTTCGCCTCAGAAATGCTGACAAATCCGCATATAATGTTCTGTGACGAGCCGACCTCTGGCTTGGATTCTTTCATGGCGCTACAAGTCGTCCAAGCTCTAAAAGCCATGGCTCAAAATGGGAGGACCGTTATTTGTACCATTCACCAACCTAGCTCCGAGTTATACGTCTTGTTTGACAAAATTATGTTGATGACTGAAGGAATTACTTGTTTTCTGGGAACCAGAGAGGATGCTGATGGGTTCTTTATAACCATGGGGGCCGGTTGTCCGAGAAACTATAATCCTGCAGATTATTTCGTAAAATTGCTATCAGTTATTCCAGATAGAGAAGAATCCTGTAGACAAGCCATTGCTTTAATAGCGGACAAATTTCACAATTCCAATTTGGGTAGGAGGCTAGCCGCAGACTCTGCTTATATAAGAGCTCAGGAAGAAGTCGACAACGCTGTATGGCTTTCTAATAACTTGAGACCATACAGAAATTCATGCTGGGCACAGTTTCGCGCGGTTCTGTGGAGATCGTGGATAAGCATGTTAAAAGATCCGCTGATAATCAAAGTTAGGTTTTTACAAACTGTGATAACATCTTTACTTATAGGAATGATTTATTATGGACAAGAACTTAACGAAGAAGGAGTAATGAACATTAATGGTGTCCTTTTTATATTTCTAACCAATATGACTTTCCAAAATGTTTACGCTGTAATCCATGTATTTACCGCAGAGTTACCAGTGTTCCTTCGAGAGCACAGAAGTGGCATGTATCGCACAGATGTATATTTTATCAGTAAAACATTAGCAGAACTACCCTTCTTTATAATAATACCTGTAGCGTTCACCACAGTATGTTATTATCTAATTGGTCTAAACGGAACACTAACGAAATATTTTATAACCTGTGGAATTGTCATTCTAGTTGCCAATGCTGCCTTAAGTTATGGTTATTTAGTTTCTTGTATTTCAAGAAATACATCTATGGCCTTGACGCTGGGTGCACCATTAGTCATACCTTTCTTGCTTTTCGGTGGATATTTCATGAACCTGGGTTCTCTACCACACTACCTCAAGTGGTTGTCTTATTTTTCATGGTTTAGATTCGGAAACGAAGCCCTTATGATAAATCAATGGGAAAATATAACTGATATTGATTGTTCAACCAACAGCACGATCTGTCCCAAAAATGGACACGTTATTTTGGAAACATATAATTTCGCAGAGGAAAACTTTACTGTGGATATTATAGCACTGTGTATGCTTATACTTGGATTCCGATTCTTTGCATACTTAGCCCTGTTAAACAAAACATGTTCGTGTTAATAAAAACAGATCGTTTTTTTTTATATTATATTTATTAACGTATTCCTTAGCATTGGGAAACGAAATTTTTATAATTTTTTTATGAAATTTGTAGCTTAGATATATTGACTGTCTATGTTTATATAAGCATTTTAAAATAAACGATTTTATACAT

>Dvv ABC-G T49457_c0_seq1

GTCGGTAGGGCAAAAAATTCAGTACACCTAGAACGGCCTCGGTGACCTCCATTCACCATTAAAGCATTTCCGCTTCCCAATGAAACACCCTGTATAATATTAAACTTATCATTTATATCAGAAATAAATAGATAATAATAAAATATTTCAAATATTACAGATCTTATGTACAAAATATGTCTAAATGTTAACAATTTAATATTTATTGGTAATGCTAGAGATATAAACAACATGTTTCAGCTATAATATCTATAAAACTGTTTTAGTGTTTTTCAAACAAACTCATAGCTTTTTCTGTGCATTGGCACATTAACTTTTATATTTTTTATAGTGCCCTTTCTTCTTCTATAACTTTACAATCTTCTTTACAATTTCTTTAGTTAAAATTCGTATTACAGCAACCTATTAACTATTTAAAATTTCATGGTTTATTTGCATCGTTCTTAGTGTCTTGTCTACGCCTTCGTCTGCTTCTGCACAAATTGAGAGCGAACATGAAGCAAGAGATCACGAAGAAGATACAGTAAAATACGCCCATTGCTATAACTGGGTTACCATAGTCGAAGGTTAAGTTATCAGACTTTAAGTAACCAAAGCTTTTTAGACTATTTGTTCCATTTGGAGGTGGACATGGCAATTGGACTATAATATCCTGGTGTTGAATCTGTTTATTTCTGCAAAGGGTGGTTATTGTCCCTTGCAAACTCTGTATGGCTTCTGTAGAAAGTTCTCTATTTAGTAAATATGGTATCAGCCACTCGCTCGGGCTTATTTTTTCAATCCATTGTAAATACATTGGAATATCTTTGAGGTGCAATGAATATCCAGCACTCAGGAAGAAAGCTGAGAGTACTGTACCGCAGAATATTGTGGCTGTGTTGCTCAAAGGGACTGTGTAGATAAATGCCATCAAAAATATTTGGATGCAGCTAAGATATAACAACATTACCCCTATATAAATATAGAAACCATCGTAATTATTCAAATGCTGCATATATAAACCAGTCATAGAGTAACTAGGGACTACATAGATCAGCCATACAAACAATGATGGAAATATATTGATGATGGATTTTGTTATGATATAGATGACTCTACCATACAGTCCATCTTTAATATCTCTCTCAATCACCTTTCTATTTCTTCTAACTTCATTGACAGTCATCGCTAGCAATAATGGCCAATGTACAATGCACATGACGCTGTAGTGGTACCCGTACCTGTCGTTTAATATAAGTTGATGATCGGTGGAAGGGATATCCCAAAATATGGCGCCAAGAATAAGGGATAGACTGGCTGAAAGGACTATCACTGTGAGCCAGCTGAGGAAAGTCGCGGGTTGGGTGTAGATCATGGATTTCGTAAACAAAGCAAATGCAGCTACAAAACAATTACAGTTTCGTACAGTGAGCGGCAAAGATGAAGGTGGTCCTGGGTCGCTTAAGGGTTCCTGTTTTTGTCTAAATATTTCAGCCAACTGTTCAATTCGTTGTGATGATTCTAACATAGCCTCCGCTGCGAGATCGTCCAAGGTTACGAGATCAAGGTAGTAATCCGAGGGATTCTTAAAAGCCGGACAGGGATACTCAACTAGAGCAAAATATGGCAGCATGTCCCGTCGTCTTCCACTATACATAGTCCTCCCCGCCGACACCAACAAAATCCTAGACAGCATAGTAAATATCTCGTAAGTGGGGGGATGCATCGTCAATATCACTATCCGCCCCAAAGTACTACCTGCCCCACCGCTCGCCCATTGTTTTAAATATTCGACTAAAAAAAAGGTATCGAAAATGTCCATGCTTTTGGTGGGTTGGTCTAAGAGTACGATATCAGTGTCGAGGATAAGGTGACACGCTACGTTCAGTCTTCTTCTCTCTGAGATTGTCATCATAGATACCTTGGTGTTTCGGACTTGCTCTAAGCCTAGATCGTCTATTAAAACGTTTATCCTATCCATAGATTCAATTTTTAAATACCCTAATTTCTCTGTTGGTTTCTTAAGATCATAATGTAATCTTAAAGTCTGTACAACTGTCATATCCTTACATAAATTCAAGTCATTTTGGACATAAGCTACTCTAGATTTCAATGTATGCGCTCTGACGCTATGTCCATTTAAAAATATTGTTCCTAAAGCTGGTGACGAGAACCCAGCAATGATGTCCAACAAGGCGGTACCTTCTTCCTCGGAAGTCGTCATAATGGCCATTATTTCGCCCCCTCGAATTTCAAACGATATTTCGTTAAGAAGAAGGTGTTGACGGGATTTGGTGCAAGACTGGGAGCTTGGGAAAATGCCTAATCCATGTGCCTGTAAATGTGGAAACATAAACGTGGTTCCAGATTCATCATCTTGATTGAGATAATCGGTTCGAAGAATAGATGGTGGTCCTCTTCTTGAATGAGGCACAGAGAAATGACTTTCAGTTTTGGACATTGGTCTTCCCATATGACCCATGTCACTATGGTCCACGACGTCATGATTGTGGTGTCCATAATCGGCAACACTGTTTCTTCGAGGATCTATTAGATGGCGCTCACTATTATGACAGGATCTATTATAACCTAATGCCAAAAGATTGGCTTCCGAAATGCTCTTATTTCTATTCCATTGACTGTTTGAATGTCGTATATCACCTTGGTGCATGGCTGCCAGTGGGATCCCAGGACTTGTTTGCTCAGCGTGGTACACATTTTGCATTCTAAAGTCCGGATCCGATCGGTATTTTCGATTTTGCTTATAGTTGTGGCTCGTTTCATTGTCAGAACTATCATATCCAGAACTTCCATCTCTTGGTCCATGACTTCCTGCTTTCGAACGCCGAATGTTATGCGGTCTAGTGGACGAGTTTCGTCGAAAATGTTGTGGAGTACCAGACCGATTAGAACCATTATGGTTCGGTGGGAACACCCTCGGAAGGCCGAATTTTAGGTAGGTGTACATGTTGGAGCCCAGGGCTGACTTAGGACCATAACGAGGATGAGAAAGGATAGACTGCACCGTTGTATCTCGAAGCTGAAAATTGCCATAAGGAAGTGGACTCTTATCTGTGGATCCCAAAGCACTATCAGTGAAATCTGAATTTAGATTTTGCCTATATATCGACCAAGCGTGCAGATCTTCACTGGTAGTTCCACCCGAAAAGGCTCTTGATTCCGGGTTAGAAGGTACCGAGTATTTCCTCTCCATTTCGAATCCTTGCGAGCCCCCTCCTTGGGAGGGGGTGCGACTACGCATCATGGTCTATGTTATCTCAGACCTTTCATTCCCATCTCTGTAAACCCTATTAACTGAACAAAAATAAGTAAATATTGGAGTTACAGTTAGTCCAAGTACTAGAATCAACAACTTCGTACACTAACCCGGTACCACCAACAATCATCCAATGAACCGGTTAAACACAAATAACACTACACAAACGACAAATGGAGATGTTTTGGTACTACTTTAGTACCTTTAATTAGCTTATGTATGCGTGCACCAACACAAAAACCGATATGATTTAACAAAGTGAAGTCTGGTTGTCTGATGTTATTTTGGAATCTACTACGCAAACACACAGTACTACCAGTACCACAGAATG

>Dvv ABC-G T36869_c0_seq1

AACTTTATTTAAACCTTAATCTATTCCCTAAACTTTGCCTTAATGAAAGAAGGCAAAGGAATCAGATACAAAAACAAATTAAAAACGATCATTCCCAGTGCAAACCCACAGGTGAGTCCAACATTGAAATCTTCTTCCAGAACTCCGCTAAAAATGACCTCCGTGGGATCTCTGCTGTATCGTTCCGTGAGGAAGGCTTGTCCACTGGCGTATCTGCAGTAAGCATTGTTGGCTCCGTTCAAGATAGAAGTTTCGAAGCTGATTTGAGTGCAGTTGTGCAGGAGATCAAAAGGAAGGGCTTTGCTGAGGGCAGAGTGGGTAAACACTTTGCGATTTAGATAGGCAGCAGCGTAGCGTGCTTGAGTCCCGTAGGTGGCGTAATACAGCCATTCTGGTAAGCCTTTCATTGACCTTAATATCCCGCTGCCAAGAGTAATGCACACCACTGTCAGATAAATGCTAAATATAGCGGCTTTCATGTAATCCTTCACAATCATTAAAATCGCCATGGTTTGCTGTTCTGCGAAGATGTAACAAGCCCATAATATTAGAGTGAAGTACACGAAATCGACAGGATTGTCGAATGATATAATTAACGGGAAAATTATAACTGCAGACAAAACTGTCGAAAGAAATGAGAAAGGCAGCGAAACCAAATTGTAGGTTAGCAGAAACAACGTGCCTCCATATAATCCTTCTTGAGTATCTTGGTAGTACCTCGTCCTATATATAGGATAGATTAATATGGTGTTTATTATTCCAACGAAGTAAACACAGCACAGACAGTTCAAAATTAAGCCATTTCTTGATATGAATGTATGTTGCCAATCCTTCATTTCTCTATAGAACAGCCACATCAAGAAGAAATATAAAGGTAGCGCAAAAGTACGCAAAAACGTTTGTCGCAGTCCAGCTCTTTTAAAACTCACAGTTGCCGCTAAAAGTCGCACGTAAATCGTCCACCCAGTTGAAAACCTTCCAGGTCTTCCATGCATGAATGGAACCTTCTCGTGTTGTCCATGCTCGTGGTTCGGATTGGGCGAGATCATGCTTGGACTTTTCTGGAAAATAACTCCTTCGTTTTTAAACTTCTCCACTAATGCTGCTATTTGGTAATTGCTTTCTACGAATCGTTCTCTGGATCTTCTGTCCACTGTAGAGAGACAAAGATAGTACATCAAAGGATTTTCCAGTTGTGGACAAGGAAAGCCAATAACGTTGAAGTACTCGAGCATTTGCTTGGTACCTCCAGTGTAGACGACATCTCCAAGACACAAGTACAACACCCGATCCAAGAACGGGAAAACATCAGATCTAGGCTTCTCCATCGTCAAGATTATAGCAGTTCCATACTTTTTAGCTGCATTTGAAAGTATTGAAATTATTAGGTATGTGTTTAGTGGGTCCAGATCCCAGGTGGGTTCGTCTAGTAATAGTACCACTGGGTTTTTGATTAGTTGGACACCAATCATCAGTCTTCTGTATTCACTTTTTGTCAAATCTTCAACGCATTTTTTAGCGACTTGTGACAATGCTAAATCTGCTATCACTTGCTTTACTTTAGACATTTTTAAATAGCCAGTGAACTTAGTCGGAGTATAATACAACGTCTGTTCAACATTTAGTCCTGGTATAAAATCACATTTATGTGTAACATATGCACATTTCTGCTGGAATAAACACATACTGACTGGATGGTTGTTCAGATATATCTGTCCTCTTATAGGGCCTTGTGCTCGTCTTGAAATAACATCAAGTAAGGCTTTCTTCCCACTACCTTTTGATCCCAGCACTGCTAGGACCTCACCAGAATGTACCAACATGGACACATCTTTTAAGATTACTGCAGTTTTAATGTTGCCAACCATTCTTTGAAGAACAGAGCGCTTTTCTACCTGTCCAGAATAAAAAACATTGCACAATTCCAATATATAGCCACCAGACAACATCTTCAAAGATTAATATATAAATAAATAAAAGAAATGATATTATTGAACGAATCTGTCAAAACACAACTAAAGCAGTACCATACATGTAGTACCCATCCTGAAACTGAAGTTATTAAAACGAGTACCGCTCGCGCGATAGTCAAGCCACGTAGACCAGAACCACATTCGCCTCCCCAACTTAACAATTACGATCTAAATTTGACTGGTCAACTGTAATTGCCCAATAATATCGTTAATTACGATACAAAATGGTGTCTACCTGTACAGGTAAAAA

>Dvv ABC-G T79525_c0_seq1* (st)

AAGGAAATTGATGCAGCACACTGTACATGGGATTAAATGTATTTTCGCTAACAAACATAAAGATTATTCCTGTCACAGACTGTACACCATTCTGTGTCAAGGCATCTGTCCCAAGATAACAGAACCCGACTATGAAACCAATCAGCATTCTTTGTGCAATTTTCATTGCCTCCAATGACGGGTTTCTATAAATTTCCAAAATCCATCTGTACGTTAACCAAAACAATTTTGACAGCCAACGAATCTCTTTGAAATTTTGACGCAATTCAAATTTTCTTTGTGTTGCAACTCTGCCCATATGAAGTTCATATTGTACTACTATTTCGACTTCTTTTGCTTCATCACTAACAGCAAATTGGTCGCATATTCTTTTAACTGTTTGTCTACAGTTATCTTCATAACCCGGAGTTGTGGATAACGTCTTAATATAAAAATCAGCAGGGTTGTAAGATGTTGGACATACATAACCCATCCTCTCAAAGAAGTCAAGAGCATTGTTTGTAGAACCCATATAAGCTATTCGTCCATCAGCAACTAAAATTAGTTGACTAAACATGGCAAATATATCTGAAGAAGGTTGATGTATGGTACACAAAATCGTTTTTCCGGTAATGGCCATTTGGTTCATAATTACCACCAATTTCTGAGCAGAATACGAGTCTAGACCAGTTGTAGGTTCGTCACAGAATAAAAGAGGTGGATCTGTAAGCAACTCTGTGGCAAATGCCAATCGCTTCTTCTCTCCACCAGATAATGATTTGGCCTGATCTATACCTCCAATTTTAAGATCAATGCACTTCATTAGTCCAAGTTGTCTGAGAATGTCGTATATTAGCTGTTTTTTATCATTTCCCGATAGTCTTCTATCAAGCTTTAAATTGGCCATTATATTCATGTGTTCCCTTACTGTAAGATAAGATAGCAACATATCCTCTTGGTGCATAAATCCGCTTAAATATTTCATATAGTCCCCAATTTGTCGACCGTTTATTAGAATGTTGCCTTCAGTTAATATTGAACCTTCAGTTCTATATCCTAAAGCAGTCATCAAAGTACTTTTGCCAGCACCGCTTGATCCCATTATAGCTACCAAACTGCCTGCTTTAACAGCTCCAGTAACTCCGTTAATTATCTGTTTGTGCTGCATCTGGCCATTTTTCGTCGTTGACGAGTATACTGAAAGATTTTCCCAAACTAAAGTTACTCCCTCTTCAATAGGTGACCATCGTGAGTAGGTACGTTGCCTTTTCTTCGCCAAACTAGCTGAGGCATATATGTCAAATTCGTTATACGATCCAGACGATCCATGGCTTTGAAATAAATTCAATGGGGTCTCCTCAACATCAAGCATTTTATTTGCAGTTGTAGAACAATTGAAAACTTTAAATTAAAAACAACGCCAGACGCGACTTTCTATTATTTTATCTTGAATATTAAACAATACACTTGATATACTAGAAAACAAATATTGTCTTTGCGGACTGTGTAAATTTACCTCGTATGAAACTTTTTGTGGTTTGCTTGTACAGAAACC

>Dvv ABC-H D20789

GCAAACGTGGCTACTTTGTAGAAAAATCAACATATTCACTCGCAAGCAACTCGAAAACTATTAACCYGGTAAAAGAACTTTATAGAACAAAAGTTGCTCGGAATTAGTCATTCTATCCATTTCCGGACTTAATTCGAACATATATTTTTCACCCACAAAAGGGGGTTAAACTCACCCCTAATGCAAAAGCACACATCGGCACAATATCACTTCTTTTCTTTGACCTGTTAGCCATGTGTATGCCAAATTCCATGTCAATCCTAGAGTTCTTTAAAACTTAGAGGTTTTGCAATATTTTACCTTTAAAGAACGTAATATTAGTAAATACCAACCGCTAGCACTACAATAGTTTCTGTATTTTTCAATAACTTTTGTCGTTGAAAATATACAAATATGTAAATATATAATTTATTTTCTACAATCACTCGAAATTGTACCTATGGTAAAATATGTAATGCCAAACTTGATTAAATTATTTTTGTTTTTTCCTATAAAAATATTTACTGACTATCGCTTTTTCTTTATCAAATACACACTCAAAACTCCGAAGAATACTGTCCACATAAACCCTACACCCATCCCGCTGTATACTTGAAAGTTGTCAATCGACCAACCTCTTTTGGATACGTTTCTCAATGATTCGATGGCCATCGTAAATGGAAGGCATCTGGAGAAGATTCTTAAAGCTGGAGGCATTCCCTCTGTTGGCCACATCAATCCACTTAACATCATCATTGGAAGAAATATTCCTGTCAGAACAGTATTGGCCATGGAATGATCAGTGCTGATAACAGATACCCAAAAACCATACGCCATTCCACAAATACCTTGAAGATAGACCATCACGTACATTAACCACAAACTTCCTGAATATTCTTGTTGGTAAATCAAATAGACGACAACCAATAGCTCTGCCGTTTGAATTATGCAAATCGAAGCCTGCAACACCAAATGCGTAATGGTAATCTCTAAAGATGTTACTCCTGCAACAATGGATCTGTCCCACACACCGTCGTGTCGGTCGGTAATGATTATCTGGGATGTCATAATTGCACCCATAAAGAACATGATGGTAATAAGACTTCCCGGTATCATAAAAACTGTGTACGGCTCATCTCCATCTCCATAAATGAAATTTATGTTAACAGGCAAGTCGCCAAAGCCAGGAACAAAATCACAATCATCAAATAAGCTGTTTTGGAATTTGGTGTATAAATCAATGAGCTTGTATTTTAAGGTAGCTCCTATTTGGCGGTTTGACATATCCATCCAAACTTTAATCTCACTCAAAGACAAAATATCTTTCTCGATATCCTTCCCTTTATCGATTCTTTCTTCTAAAAATGATGTAAAATTCGAAGACATGTACATAGCTCCTACTATCTTGCCATGCAAGACTGCATCTTTGGCATCCTCGAGAGTCTCAAAATGAACCTTTTCTATCATGGGATGGTCCAGATACGATAAAAACCGACAGCTGATATTCCTAAGCTGACAGGCTCTATCGTCTGTAGCAGTTGCCGTACCGTTAAATGAAAAGCCTGGGCATGTAACGCTCATCGCTTCGTCGTTAACGATTCCCAAGGGTATGCTTCTAATGTCTCCACCTACGGCTCCCATAAATACCCCTACTTGTAGAATTGGAAATGTCATTAAAAATATAATACCCGTGATGTTCCTATAAAATTGTTTTAAATTTTTATCAAACAATGCTTTCATTCGACTTTTATTTAAGGCATTTCTTGCCTTCAGTATTTTCTTTTTGGCTAAAATATCAGTGGAACCATGACCGATTTCAAATGTAGAAACCGACGTTGCTACGCTAGTGGTGGATCCTGTGGTGTCATTAGCTAACATAGAATTATTCTGATCATCTACAACTCGATGTGAGGTTAGTTCTTGTAATCGTCCTTCTTCCTGTCTTTTGCTGAGCAACAAAAATACGTCTTCCAAAGTCTCACTATTAAACAATGTCAATAACCTAGTTGGTGACTCCTCTGCTAAAAGTTTTCCCTCTCGCATTAGACCAATCTTATTTGCCTGTCTACATTCCTCTATGTAGTGCGTAGTAATTATCACAGACGTATTATCCTTTTTAGTAATATCTACTAGATGCTTCCATATACGATCTCTTAACACTGGATCCACTCCAACCGTGGGTTCGTCCATTATTAAAAGTTCAGGTTTATGGACCAAAGAAGCTGCTAGGGAAACTCTCCTTTGTTGACCTCCACTACAATTTTTTAAATACCTATCATCTGGAGGCAACTCTAAAAGAGTATGAAGATTTCGATAACGTTTTGCTATTAAACTATCTTCCATCGAAAAGATTCTTCCAAAATAGTAGATCGCATCTTTGACTGTAAACTCGCCAACAAGAGCAATGTCCTGTGGCATATATCCAACTCTGGGTCCAGGTACTCCGCTGCCAGCTTCTCCCGGTTTTCCACCCAAAACCCAAATTTCACCTCCATCTATTTTCTTTCGGCCTACTATGCTGCTTAATAATGTAGTCTTTCCGCAACCACTAGCTCCCAAAAGACCGTATATACTACCCCTTTCTACTTTCATACATATTCCAGTCAAGACTTCTTTTTGTCCATATTTTTTCACTACTCTATCAACAAACACAGCATAGTCGTCGTGCATTTTCGATTTTGAAGAAAGTGAAATTATGCGCCGTACTCCGCCAAACTGTTAGAACATATCACATAACTAATAAATTTAACAAGCAAATTACACAGGGGGGTTAAAATGCACTTTTAATTGAGCAGTTGTGTTTTACAAAACAA

>Dvv ABC-H D5118

TAAATTAAAAAGTTAATACTTTAATCATAAAAAAATCAAAACCTCGTGTTCCTATTCACCCCACCAAGTGGGGTGAATGGGATCACCTGTATTAAAATCTATTGTGTTCCTATTCGTACATTTGTAGGGTCAATAAAAATAAAAAGTGTCATAACGTGTAAAGTAACATAAACGAATAAATATAAAGTTTGTATTGTTGGAAAAATATATTGTTGAAATGAAAAATATTTTTGAATATTTTCTAATTTGGCATTTTTCTCTGAAAGACTATTAACCAGTCTCTTTAATAGTCTAGGAAACTTGTCTTTAGCTATTGTGGATTCATTCCTGCCTGGTACTGCTTTTCATTCCTCAACTATTTTTCGCCACATAATCTTAATGGGCCTTGTTGAGTCTGTACATGCATTTAAACTTTTTGTACAACAGTTCTGATGATTGCAGAAAACCGTATAAATATTTATTTCAAATTTACGGAATCCACATTTGGTCGTTCGAGCCAGATGCTGATAATTAGTGCCCATAATAATTGGAAAAATTATATTCATTAATACTACGCATGGATGAGAACCCTGGTAGAAGTTTGTTTTGGATTGGAGAGGATAACTAAACGTAGAAACACGTTTTGCAAGGCATGGTTCTTTCTATTCTCTTTGTGTGGATCAATATAATATGTATAATTTGTGGTTAGATGAAATATTGTTTCGTTTGGTCTTACTCTATGGAGATATAAGATAAAAAAAAGTAACTTCTAATATGTGGGGTGAATGGCAACACGGTAAATATTATGTTCCTATCCACCCCACATATCTCAAATTCATGACGTCAACAGTAATAATTGTTGTCAACAAAAAAGGTTTACAGATAAATCAAAATACCATCATGTTATGTTGTTGTAAAAGTTAGAATAAACAAAAAAACAATACTCTTCAATCTATTTTGACAGACATTTTGTACTTACTGAGAAAGTTTTTTAGGATGAGAAATATCCTCAACATTAACGACTTTGTTTGCACGCCGAGAACATAGGAACCAGTGACGCCAACAACACAAACCTAAATTTTCTAAAGCCATATTACGTTAGCCAACTTGAAAAAAATTAAATAGCCCAACGAAAGTGATATATATGTTTAGGAAGATAAGTAATTGTTTAGTGAAGTTTTGTTCCTATTCACCCCATTTTACGGTACTTGCTAGGTTTAAAGCTAAAACAATTACCCTTAAATTAAGAGGCCACATCAAATGACTATTTTACAATTGTTTTTTACTTAATTTGTTATAATTTAATTGTCAAACACCAGTTTGTTAATTTTTAAAAAGGAACTACATATCTATTTTCATTTAGAATTAAATATCTCTAATACATAGTTTAGGAGATAGAATCATTTACATAATTAAAGTTTTCAGCCTGGTACCCTAGAAATATCGAATCTACGCACCAAAAAACTTAAAAACACCATGGTACCAAAAGGACATAAAAGCGCATACTCAACCTTCTTGGTTCCTACACTCCTAATAATGAAATACTATTTGTTTTATAATATATTATTCTTGACCTCATAACGCAACGGTACCTTTGTGATTATTTCAATATCAAATTTCAGCTTTAATTTTAGTATTCCTCAAAAACCTATCTACCTAACGCCATAAAATACCATAAAGACAGTACGCGAAATGAGTAAAAAATATACAAAAATTAAATTAATACTAGTACTTACAGTATTTAAGGGGGCAAGTTTATAACATAGATTTACTGTCCATCACTTAAACCAATTTTTGGCTGTATTTACTGGATTCGCAAAAACACCCGTCCAAACAATTAAATAGAGTTTTTAGATCTGATCTGTTTATTAAAAAGTAGAATTATACATACAAAATTTCCCACGCGGTAAACAATTTACTAAATGGTTTTCAACCCCTCTTAGATCTTAGCACCAGTAAACTAATCGTTAAAAATAGTACTATCCATACTATCGTAGCTATAAATCCGTAATAGACGTCGGGTTCTCCTATGGACCAGCCCCTGGTCATCATGGATCTCAAAGAGGTCGTGGCCAGGGTCAGAGGCAGGAACGTGGATATATATCGCAGCACCGTTGGCATTCCCTCTATCGGCCAGATGACACCGCTCAACAGAAGAGTTGGGTAAAAGCTACCCAGAGCCAGCTGGATTGCATTTCGTTCGAGCTCACAGATGGCAGAGATGACGAATCCGAAGCACATACCACACAATCCTTGAAGAATCGTAATAACTACGACAAGGAAGATGTCTCCTTTGCATTCAACTTGGAAAACGATAATCATGAAGATAAGTACGAGAGTTGTTTGGCCGCACATTACCACGAACTGAGTGATGACGTGGGAAAATAGAATTTCGCTTGGTGTTACACCGGCTACCCAAGATCTGTCCAATAATCCTTCCATTCTTTCTATAATTAAAGCAGAGGATGTCAGAGCCACAGCCAAGAAAAACACGATCGTTAATATTACACCAGGTGCTACGAAGTCTGTAAAGGACGGCTGGTTAGAGCCATAGATAGGTTCCTTAAACGATATAGGTATCTCGGCTAATTCTTCATTATATTCACAATCCCTAAATATATCTTTCGTGAAATTTTGGAACGCCAGCTGAAGATCTCGTTGAAGTATAATACCTATTTGTTGATTTGACATATCCAGCCAAACTCTCACTTCGCTTTGATCCAACGTTTCTTCGTCCGCGTCTTTCCCTAAGGCCATCCTAGCTACCAGAGCGTCGGTAAAGTTCTCGGTAAAGTACAAGGCACCCCACGCGTTGCCCGTTCTAACCGCCTCTTTGGCTTCTTCGGGCGTCGCATAATATTGTTTGATGATCGTTGTCGTATTTATTGTGTCTACGTATCGACAACTTAGGTTGCCGAACTTACATCCTTTTTCATACTCGCATTCCTGATAAGTCAAATTCGTAAAGTTCTTTTCGTGATTTACTATGGCTAGTTTGAGTCCTTTGGGATCTCCCCCGATGGCCAAACAGAAGAGGATAACTTGCATCACAGGTAGAGCGAAGATGAATAACATCACCCCGACATTCCTCCACATTCTGAGAAAATTCTTTTGGAGGAGGGCTCGAAGTTTTCCCGTCGTTGTGAAATCTGAACAGCTACTACAGTCGTCGCATGCTTCTTTGATACCATGAGTTGCCGAAGAAGGTGGTTTTCCCATATCTAAATGTCCGTTGCTATCAGGAACTAAAACTTCTTTGCTTTGATGGAAATTCAAGCCGACCACTCCACTCTCCTCCGTCACGGAGACGGACTCTTTCTTGGACCAATTAAGTGTAGCTAAACTAATGTTATTAGCCATATTTTGGTCATTAGCTGCCGCACCTACTTGAACTTGCTTCCTAGATAGTTTTAAAAATACGTCTTCTAAGGAGGTACATTGATACATCTGTAGCAAAACTTGTGGGGATTCTTCTGCCAGGAGCTTACCGCTCCTCATAAGGCCAATAGTATGTGCTTGCCTAGCTTCTTCAATATAATGTGTTGTAATTATGACTGTCTTGTTGCCGTCCTTTGTGATTTGTACAAGGTGATTCCATATACTTTGCCTAAGCAAAGGATCCACACCAACAGTGGGTTCGTCAAGGATTAAAAGTTCTGGATCGTGCATTAGAGCCACCGCAAACGACACTCTTCGTTGTTGACCACCACTAAGATTCTTAACCATACGATTTTGACTAGGTAAATCCAAGAAGTTAAGTAAAAACTGCAGTCTTTCGTAAATTTCTTTTGACTCCATACCGAAGATCCATCCAAAGTACATCATCGTTTCTTTGATTGTGAATTCTCCGTAGAGAGCTATTTCCTGAGGCATGTAACCCACACGCTTACCCGGTACTCCCGAACCTTTTGTACCTGGTTTTCCTCCCAGTACCCATATCTCTCCAGTGTTGAGTCTCCGCCTTCCCACGATGCAGGACAAGAGAGTCGTTTTCCCACAACCTGATGCGCCCAGCAGACCATATATTGTTCCCTTGCCCACAGTCATGTTTAAATCACTCAACACGTGGTTGGGTTTCTTCTTCGAGCCATAGTGCTTGTACGCGTGCCTTACACTCACTGCATTCTGTCTTCGGTTCCATACTGTGGACTGTTGATGTACTAGAGGCGGCCTTTCCACATTTACACCCACCAAATCGGGCCCAGGAACTTGTGGCTCCACCCGATCACCCATTGTCGTACCCTCAATGCTTAATGCTCTCCTATAGATTCCTAACAATCTACCCAAAACATTCACTAAATTATTCCACGAAAACATCTACAAACGATTAACATTCCACTACGTTCCTCTGGCACATGACACTGATCACTAACTCACCAACACAGATAATTAGGAATATTAAACCAAAAAGTAATTATTATCAGTTTACAACAAAAGGTAATTGATATCACTTGTTTTCTTGGACACTTATCATTATTGCGAATATATTAATGGTGGTCTATTTGATTATTATTATAATTGCTTTTAAAATACCATTAGCTAACGTTATTTCATGGACTAAACACTAAACTTTGGAGTTGTAACTGGGATAAAAATAGCTCGGACTCGACCACGTTCAGTGTTAGCGTGTCAAGATCAGCGAAAATTAATTTTGTTTTTATAATAGATTCATATATGCATACGCATTAGCGAAATTAGACCAGTGATAGGAACATCAATTTTATCCGAGAATGAGTCGCAAAATGCATATGATTAGCTAGCTATACAGGGTTTGG

>Dvv ABC-H D18290

ATAGCTAGAATGCAGGGAGGAAGAGTCCCCATAGAATTAATAAATATTGAAGCTGGCACAAAAAGCATAAGAGGACGGTCACGAAGAAAATGGATGGAATCGGTAAAAAGAAGACTTGGCATTGTTGGGAGTACAAAACTAGAAAACAAGATAGGAAGACAGGAAGGAAACCATTGAAGCCTTCGGCCTCTAGGGTCTGTTTGAGCTGATGTTCATGTATATGGTAGCAAGTCAGTCAATAACAAACAAATTCTATGATGTCAATACAAAACCGTCGTTTGTTTCAGAATAAGCGTTCACAAATCATAAGATGGAAAGGTAGAGAAGAAAAACGAAACTTGTACAATTAGGTACCTACGTACTGCGTACAAGAAAAGTGAAACAATATTACAATAATTTAAAGTATTTAATAACTGTTTATAATATTCCTTATTTCTTTTCTCATCTTTTTGTTTTCGTCAAGTAGATGTTCAGCGCTAAGAGAAATAATATCCAGAAAGACAGTATTCCTATTGAGTGAAGGGTATGTAAGTTGGTAAGAGTCCATCCTTTCCTGATGACATTTCGTAATGCTGATGTTGGAATTGCCAATGGCAACCACKCTATGACGAAATATATATATTTGGGTAATGCTTCCGCGGGCCACAAGATACCGCTAACTAGTACAAGTGGATTGAAGATTCCTGTAACTATTGTGTTTGCTGTTACATGATTATCGCTTAGAACTGATATTGCAAATCCTAACATGATTCCAGCTAAACCTTGTAGGTATAAAAGAGCGAAAATTAGCCATAAAGACCCGATATACGGTTGTCCAAAAATTACAAATACAAACAACAAGAAGTCCAACGATATAATAATCATAAACACGTTTTGATACAGAAGATGTGACAAGCTTAGCTCCAGAGCTGTAACACCGGCAACGATGGAGCGGTCCCAAATACCTTCACATTTTTCATCCAAGATTATCACGCAGGTCATCAAAACTCCCATAAAAAATATCATCGAGGTTATTATTCCAGGAGTGATAAAAATTACATATTCTTCATTCTTATTTCCGTAAAAGCTTTCTTCTACTCTTAAAGGTATTTGTACAGCTTTCGATAAATATCTGCAGTCATCGTAGATCTTTTCGTGAAATAGCTGGTACTTATCAGCAATTCTTTCTTTAATAATTCCGCCTACCTGAATAGATGTCATATCCATATAGACCTGCATTTCTTCATCTGGCAAATCGTACTTATCTACTCCATAATTAACTCTAGCTTCAATTAGTTCCGACAAATTCTGATGCATATGTAGGAACCCCCTTATTTTTCCTGACTCCAAAGCTGACAAGGCTTCATATTTTGAATCAAAGCGAATCATGTAAAATAATGGTACATCCAAATAGTCCTCAAACAGGCAACTGGCACTATTAATATGGCAACTGAATTTATCGTACGGCACAGCTGATTCGTTTCTGGGAAATGTTTTGCAGAACTCTTGAGATCTTTCGTTGTTGACTATACCGAATGGATTTCCTTTTGTTTCTCTGCCAACTGCTGTGAGAAAAAACAAAACCTGAAGTGCGGGGAACACAACGAGAAATATTGTAGATATTAAATTTGATTTGAGCTTTTTATAATTTTTAAACAACAATGACTTGAATCTGATCATTTCGAAAATGGATTGTTTTTTTCTTTCAGTTCTCGTTTGTTGAGGTGCCTCACTAATTACATCCAATGCTGCATTTGTTCGTTCAGTAGATTCATTTATGCCTGTTTGAGATGACTGAAAAGCCAAATTATCTACTCCAGAAGTTTGTTCTGTTTCTTCTTGTTTAGAACTTAGAATAACAAAGACTTCTTCTAGCAGATCACAGCCGTACCTTTCTAAGAGCGTCTCCGGGTGTTCTTCTGTTAATAACTTTCCCTTTCTCATTAGAGCAATCTTATCAGCTTGTCTAGTTTCTTCTATATAATGTGTTGTTATTATCACAGTCGTTTTGTCCTCTTCAGTTATTTTCGTTAAATACTTCCATATTCTATTTCTAAGAATTGAATCTGTACCAACAGTAGGTTCATCCAATATAAGTAACTCTGGTTTATGAACTAACGTTACAGCAAAGGACACTCTTCTCTGTTCTCCTCCGCTACAATCTCTTACACATCTGTTATCCGTTGGAAGGTCTAATAATTCAATCAGTTCATCACAACGACTTTTTATTATGTCATCCTTTAAATTGTAGATTCTTCCAAAGAAATAAATAGAATCCCTTGCACTTAATTCTCCAACTAATGATATATCCTGTGGCATATACCCCACTCTATCTGCTAGGACTACACTACCATCCTGTTTTTCTCCCAGTACATATATATCCCCTTTATTTACATTACTCCTTCCAATTATACACCTTAATAAAGTTGTTTTACCACAACCACTGGATCCCAAAAGTCCGTATATGGTACCTTTTTGAACGTTTAAACATAATTTGTCCAGCACTTTGTTTGAACCAAACGATTTTTCTACATCCTTGACATATAGAGCAAGATGGTTATTCATTGTGTTCATGATAGAACACAAACT

>Dvv ABC-H D11818

CTAGAACAAAATTAAGTACAAAATTAAAAACTCCTAATGGAATATACACCCCCTAACCCTTCTTGAATTTAAGTAACAGAATACTGATCGTCAAAAATATAAAGATCCATATGGTCAACGATATAAAACCGTTGTAAACTGTGGGATTACTGATGGGCCATCCTCTTGCTAGTATACATCTCAAACTCTCTGTGGCTTGGGTCAAAGGTAGTACCACCGACATCCACGATATGTACTTATGCATGGCTTCTATGGGCCAAATGATGCCACACAACATTACGATCGGTAAGAAACTGCCCATAGCTAGGTAGGTAGCAGATCTTTCATTTTCACAACTGCAGGCCACCACGAAACCAAAACACATTCCACATATACCGGCCAGTACAGTCAAGGTTGTCACTGTCGTCCAATCTCCTCGTTGCGTTAAACCAAAGAGAATGAAAGCGACAAGAAGTACTGCGATGGACTGTCCCAACATGACGACGAACTGGGTGATAATTTGCGAAAACAACAGCTCCGTTCCGCTGATGCCGTTGACCAAAGATCTTTCCAAGATTCCTTCGTTCCTTTCCATGATCATTGAACCGGACGTGAGGGCAACAGCCATGAAGAAGATGATAGTCAAGACTACTCCAGGTGCTGCAAAATCGGTAAAATCTGGGTCTTGGTATCCATAAATTGGTTCATTGTATCGAATAGGAACGCCAACACTTTTTTCATTATATCCACAACTTTTCACAAATTCAGATATAAAGGTGAGGAATCCATACAGCATATCTCTTGTAAGGAAAGTTGCGATGTTTTCATTTGATTTATCTTCATAGACACTGATAGTAGAAGCCATTATATCTTCTGGAGGAGTATCTCTCGAATTTTCAATTCTACTCCAAAGCGCGTCGGTGAAATTGTGTGGAACGACTACAACACCCCAGGACTTTCCTCGTTCCACTCTATGTCTCGCTTCTTCTTCCGTTTTCATAAATTCCCACGTTATAGAATAACTCTTAGCCACTTCGTTCAAGTAATTACAACTTATTTTTGTACCATTACAATTTAGAGGTTGCTGATGACAAGTCTCATCAGGGAAATTAATCTCGTTATTGACAACTGATACTTTTATTCCTTGAGGGTCGTGTCCAATAGTCCAACAGAACAAAATGGTCTGACTTATTGGTAAACCGATAATGAACATCATCATGGGAATATTTCTCCACATCCACAGGAAATTTTTCCATATCAAGGCGCGCATGTGATGCGATTGGACAAATTTAAAGTAATCTTTGAAGGACACTTCCGGTTCCTCGTCTGGTGGTAGTTCTGGAATAATGTGTTCTGCCGTGGGTTCAGGAGCAATAGACACTCTTCTTCCTGTCCTACTAGTAACCGAAACACTGTCTCCGAATTCCCCAGAAATCTCTCCCAAGTCGTCGTCCAACACAGCAGCAGGATTAACAGCTCCACCTAGTTCAGGCACCGTTATCGTCTCGACGACACTTTTTGCTATACTGGAGCGTCTTCGTTTTCCCATGTTTTGCATCACGCTGAGTTTCAAGAAGACGTCTTCCAAACTATCCACCCCGAATTGTGTTATCAGTCGTTCTGGGGATTCCTCGGCCAAGAAGTAGCCTCCACGCATAAGACCAATCAAATGAGCCTGCCTAGTCTCATCAATATAATGCGTGGTAATGATCACAGTCGTCCTACCATATTTAGTTATTTCAACCAAGTGGTTCCAAATATTCGTTCGAAGCAAAGGATCAACACCAACGGTGGGCTCGTCCAAAATTAGTAACTCAGGTTCGTGAAGAAGCGTAGCTGCTAAGGAAACTCGCCTCTGTTGCCCTCCGCTGAGGGTTTTGACCTGCCTATCTGCGTCCGGTAACATCAAGAAGTTGATGAAAAAATCAACTTTGGCTTCTACTTCGTCTGTGGTCATGCGGGATATCCAGCCGAAGTATTTTAGAGTTTCTCGGATTGTGAATTCGCCGTTCAGTGCTGTCTCCTGTGGCATATATCCTACTCGTGGACCAGGAACTCCACTGCCTCTCGAACCAGGCGTACCACCCAGTACCCATAGTTCTCCGGAATTAAGTCTTTTTCTACCTACAATACAATTGAGCAAAGTCGTTTTACCACAGCCACTAGCACCCAAAAGTCCGTATATACATCCTTTGGGCACTGTCATGTTAAGATTGTCCAATATAACATATGGATTACTTTTGGTGCCATATTTTTTCACAGCCCTCCTGACACAAACCGCTTGTTGTCGTCTGGAATTCAATGTAGAAGCTTGAGTTTTGAACTGCCTTCTGCGTAGTTCGATGTCACTGGGACGTTCCATATTGGCAAGAATCGTCCCTTAATAAAATAAACTTTTTGGACGTAATCCAACTTTTAGAGGATATCAATGGCACTTGACACTAACGCGGTTTTTTTTTCAAATCACGCCGACATCTGAACGAGTCAAAGAATTGAAAAAAATCACCTCGATCGATGTAGACCGCACGATTTGGCGGACTGTTGTGTTTTGTTTCGCGATGCGTGGCGTTCTTC
