# Supplementary material for: Structural and functional insights into the Diabrotica virgifera virgifera ATP-binding cassette transporter gene family
Source: BMC Genomics. 2019 Nov 27;20:899. doi: 10.1186/s12864-019-6218-8 (PMC6882327; doi:10.1186/s12864-019-6218-8)
Supplement: Supplementary file 8 — Additional file 8: Amino Acid Sequences of D. v. virgifera ABC transporters. [file 12864_2019_6218_MOESM8_ESM.docx]

**Additional File 8:**

**Amino Acid Sequences of Dvv ATP binding cassette (ABC) transporters**

>DvvABC-A_49125

MSLLQLEVVVWKNYIIRKRHWFLTIIESFLPVCLFLLIAYGRSQITGLNKIEVPNPTYGRIDGINYNIDVAGTYILYTPYNEYYTNITTKMLTKLGMPSDHIQRFGSENELLGYYYKNPNYTTVAIIFDDKNPKHFNYKIRYHKNYYQPEFLVTDRMYQRVYGYSPGSEYVYRDEDFLKIQLALDMSFLEENLRSSNIEDTYSIQIQEFPYPPHKVDSAITTLFLEFLPLITLFSFIFLCPAVLKRVVEEKHSGTKELMKMVGMKSWMLWFGWFIYAMIPMFFAVSVISIFMKVPMFGSDSPLVEFANGGILFVFLILYCMAAVAFCFAISSFFSRPTIALVAGILVWILSFFIPKYACGLDEANKLSWLSNVLLNLLPNMSLHYGYSIISVFEEREVGINWSNFFKPGSGSSDDITMLNVYVMLIVDIVIYTIFTFYMDGVNPGKYGVRKSILFPLQNFMKLCRKPSITTVPVDSETVPLEKVEAGHNLAKGIQINHLHKRYKQKQAVNNLNLDIYKNQITVLLGHNGAGKSTTMSIITGLIPATSGSVTINGLDINTDMDEIRKSLGLCPQHNLLFTDLTVKEHLLFFAKLKGKSTKEANIEAKSLLAKLNMPDKEHSMAQTLSGGMQRKLCLAMALIGDSKVLILDEPSSGMDPQSRRELWDLLLQWRGEKTILITTHFMEEADALGDWIAIMSDGSLNCYGTPMFLKKKYDTGYHLNLMIEEDADIDAISRRVKHFMPDAHLTSNNGNNLVFVLPYQNTNMTGLLGDLEKNRSELHLSNISITITTLEDVFLKTRQEIEHSSDDTSSVATQDDIQPHASLTLITLLKKKVNFSLKKWSTYIVPVGVAVVLFSLTVFLSSGNDYYSERGPELPLKLSSYKQTSVYYSGDTSNEKINTLMKYYMSAVESQRSLPFKVDNVEKSILQRSLENIAYYKEHMIAGANFVYDRDFTLVALYNGIATHSTPISLNLITDSLAKTLLGPEYGISVSNWPLPQVQERLSSQEYSEAKVAVLWLILLPVGCLFIHGIFIIFPHTEISTRFLQIQYMAGVKPFFYWLVNWIADMTFYIFLMFILSFLLWICSPVFQHNGTELGYLFSIFLCYGIAGIPFAYIFSRKKTASGAFALFVIMGMFLGIILTLTIAVLLESQDEYYVNIGNKIKYVCFFLLPQVSLSDALVSFIRRTVNIYNFKISPQRLRVMCNQMESHACCVESSIECQNYKSYNNLFSEHYMFMIGCGVFYLTINIILDTYFMKKLKAMVMRQCNLILKSFKDKDTALIPKNDKYVNEDVGDGYNTLRAKKIMKLYAGKQIVKNINFTLKHGHCLGILGVNGAGKTTTFKMLTREEVVDDGEIKIELDNNKQPLDITGSEYLETIGYCPQSDSLNFVLTGRQLLSTVAKLRGVTDETMIDRFLEAFDLKQYADIPCGHYSGGNKRKLSLAISLIGNRKFVLLDEPTNGVDPATRRKCWDLIKLMKGNSGNKIGFILTSHSMTECEALCDELKIMKKGSFVEEGRLVDLKNRYGGFTLKLKLVSNQGQPINLVDDDVDEVDGVISNKFKSVDDLKSYFTNHDRGEIKDEHSGLLHVYIKDKTKKWSDIFQEVEALKTHNSHLIEDYAISEASLEDVFLKVAREDEEDVTKKK

>DvvABC-A_50718

MGEKLDKFLLLMWKNWLLQYRKPIQTIIEIVAPVLFSFLLVYIRSLSDPTYEHERIYKPFCAFEGNFNLSLPLFCNDPDAVQAGPSLFSSMQTGDSNFSLTILYTAPEDKYYDDVIGFFKLIPKVNTQRVISSGELERISLAGNSSYYGIEFGGDRDNVEVNIRFPGETVYQLDSIGRQNWRTNLVYPVFQSSGPRSPDNATGSAPNYFAEGFLAVQHFVTTALLLADKGITHEDPQEIFNFLMDKSNPFPLIKMQRFPFASWFSDQLLTALTSMLGMIVMLSFVYTCINTVKSITTEKEKQLKESMKIMGLPNWLHWLAWFVKCFIFVIISVALMVVLLKVRWYSNTEFTVFTYADPFVLFVFLLLYCCSMITFCFALSVFFSKANTAATMAGLAWFISYAPYLFMQNNYTDLALSTKLLTSLLPNTAMAFGFQVVLMYEGTGDGVTWSTLFKPNTPDDTLSLGLIWIMIIINTIIYLLIALYIEAVFPGEYGVAEKWYFPFTKKYWCGKGAVSDYNNYNDHSANEFFETEPNLRPGIQIFNLKKAFGKKTAVRNLSLNMFENQITVLLGHNGAGKTTTMSMLTGMISPTNGTAKINGHDIRLDIAGVRNSLGLCPQHNIIFDELTVEEHLYFFSKLKGLSKSKITAEIDKYVKLLDLEPKRKEKSSTLSGGMKRKLCVGMALCGNSKVVMLDEPTAGMDPSARRVLWELLQKQKEGRTILLSTHFMDEADLLGDRIAIMAGGELQCCGSSFFLKKKYGAGYSLIMDKAKTCNPHKVTELLKKFIPEIEIHSNVGSELTYLLSENNAPVFEAMLKQIEQESNELGIRSYGISLTTMEEVFMKVGADHGQEEIYNEKQKKDKKEKNPILTSADLGPESLSPSYTGGLHLIVNQFVAMLMKKIIGTFRSWVLLAIQIFLPMINVIIVMSVPSGSVPSVLPAMPLNLARFTDPITLIERFDDPQNYTNNFYGVLNGYGMSPTNIDNITRIMLDLTATNPNVVRRSYIIGSTFTEQEFEFGIIKTIHPVITAWFNNDPYHSPGIALGMALSSVYNHYTNGHIEFVNKPLPFKAATQIDNIVGGQSQGFQFAFNIGFSMAFISSFYVIFLIRENVSKSKHLQFVSGVKVYIFWFVNIICDMFVYLLVCSVLLITIYCFQQDGFKTSGDMGRLFFLLLLFGWSFMPIYYVASLIFTVPSTGYTRMTLVGIFIGNAAFLLVEVLKTQSTNLKRIGETLDNVFLVFPHYSLATGINKCYAIYSYNTLCETVFDSCAQNNFTKDECISKFPSTVADICTNLNDNYFSWNNNGIAKNVTYSLISGVLWSILLFIIEYKFIARLMYYINQKFFPKQPILIQDEDDDVSKEKERIHMATDHDIRQTNILVVKDLTKYYNNFLAVNGLSIGIEKSECFGLLGINGAGKTTTFKMMSGDETVSYGDAWIEGKSVKTQLKQVQRNIGYCPQFDALLDDMTARETIIMYCLLRGIEFKRTQRIANFLSRDFDFHRHLDKKVKEMSGGNKRKLSTVLSLIGDPPVLFLDEPTTGMDPATKRYLWDSLCKIRDNGKCIVLTSHSMEECEALCTRIAIMVNGNFKCLGSTQHLKNKFAEGYTLTIKLKKLPESGGLVHADTESLEKYIKDKFPYAHLREKHQELLYYYITDTSMAWSTMFGILERAKRSDLNIEDYSLGQSSLEQVFLTFTKHQNPEGDDVKKKN

>DvvABC-A_18330

MSAESNSNRKRNHSYTVLQDGSDIGFQEPRGAMAENLDKFILLMWKNWLLQYRKPVQTAVEILAPVIFSILLVVIRSLSDPVRHETVVYPPFCTIPIALRENKTGITICPSYDGVPYMDRNQSDGGNSSNNPFEKFALVYSPSNPPIDQVMNYFRLAFKNVVALESSQALEKYFITNSSNITFAGIQFDDSYKTLKSLDDVKHLQVSIRFPGETRLKLDPFNYNNWRTNLIFPIYQQPGPRLYNLITGAAPSYYREGFLGLQYYLTLSVLLAKSNVTYATDDYLDLISWVIENQFPLVNMRRFPNAPWYEDILLTALKSLIGIIIMLSFVYTCINTVKSITTEKEKQLKESMKIMGLPNWLHWTAWFVKCFMFLLISSVLMVIFLKVRWYTNTNFTVFTKADPFVLLLFLMFYNCATITFCFALSVLFNKANTAATIAGMVWFLSYSPYLFMANVYDTLTLTSKLVASIGSNTAMAFGFQVILMYEGTGEGIQWNNIFTPNTPDDSLTLGLILIMLTVDSIMYLLIALYIEALFPGEFGVPQPWYFPFTAQYWCGHPIYRGVEDFDNGAIKGEFFETEPENLKPGIQIRNLKKVFNQKVAVRNLSLNMYEDQITVLLGHNGAGKTTTMSMLTGMITPNGGTAKISGYDIRTDMEGVRKSLGLCPQHNIIFDELTVAEHIYFFSKLKGMRKGEIKSEIAKYVDLLELQEKRNSKASTLSGGMKRKLCVGVALCGNSKVVMLDEPTAGMDPAARRALWNLLETQKDGRTILLTTHFMDEADILGDRIAIMAGGQLQCCGSSFFLKKKYGAGYSLIMDKSQECDPRRVTQLLKKYIPDIEINSNVGSELTYLLVDDHVHVFEPMLRELETESEMLGIRSYGISLTTLEEVFMKVGADHGQEEMYNHEHGNIVQNGTAQNGFTNGINGTHKTNNGTHTMVPTYSNGFSLLLNQIIAMLLKKFVSTVRSWILLGIQVMMPTLFLIIAFVVARKNKMTGNLPAMPLSLSKFENPVTLVENGTSDYLPYYMKVLEDYGYPATIVDNITSVLLDKTEHHPILVTRRYQAAATFGESGIPDLPFNLPNLTAWFNNNPFHSPAVSLSLMLNSIYRKLGGCDDCTIEFTNSPLPYSAATQASQLLTVQNIGFQLSFNIGFSMSFVASFYVLFVIRENRCKSKHLQFVSGVKVYVFWLTAAFCDMLTYLFTVFVLMITMVMFQEDGFKSGSDISRMFFILFYFGWAFLPMFYLSSYFFQVPSTGYTRMTLVSIFGGNAAFLVVQVLQSPGLDLQYIGNALHWLFLIFPHYSLATGINESFKVYAYNNICANLLKTCEEQHIPKKTCISIMRNERIREICEDADLNYFKWKAPGIARNMVYSFLTGIILFALLLAIEYKIFSRVHYYLTQKHFTKKPIPVEDEDSDVSKERERIHVATEIDIKQNYTLAVKDLTKYYKNFLAVNGLCVGIRKFECFGLLGINGAGKTSTFMMMTGDTSISYGDAWVNGKSIKQHLEEVQKIIGYCPQFDALLDDMTAEESIIMFAMLRGLPFKDTFKLADYLSKEFDFTRHLKKKVKELSGGNKRKLSTAIALIGDPPLLYLDEPTTGMDPATKRHLWNALCKIRDRGKCIVLTSHSMEECEALCTRIAIMVNGNFKCLGSTQHLKHKFAEGYTLTIKLKKIAESSSEGLSETEPIEKFICQRFPGAQLRERHQELLNYYITNKSVPWSKMFGILEKGKRSDLNIEDYSLGQCSLEQVFLLFTKHENST

>DvvABC-A_266167

MAYGGKSLFFSQLKAMLKRNLLLKKRQKLKTIGEILFPIYFLALLVVIELVLPDPNLPEISTPRGEEYLFKSFDNGESHKIAYAPNTTTNINFVNNVTETWKKMSNSKSSLEWVPYSAEDKVQEAYDQDHKFAPMAVIFKSDVPYVHTSLSYQIRTNPYYISTPSSNDIGWDKQSCRGGTNPNSNMEDGSTCPANTYYFSGFLALQTLLDYTRIQLDNPTQDNIPYISLEIFPKAATTVGDFANMIRIIIPIYMVLALSQFVTYLIILIVGEKEKKIKEGMKLMGLMDKVFWLSWLIIYACFVILLATICVLLLFAFKVFVHVNLLLIFILMVLYGISLIMFAFMLTPFFDKARTAGVLSSFILVLIIVLYFVEQIVSGKHPQYLWALSLLSPAGFASALDKVAISDIQGKGLDFSNMWDNHGSGVPFGGSLVMIMVDIVLYAIIGWYLDNVIPSSYGVKRSPIFFLMPSYWRSSHNQIPPNQLNTESSVDVEPVPREMQDKEAIRIINLNKSFTQCRKPTVTALDGINLSIYKGQITAILGHNGAGKTTLFNILTGLSSPTSGSALVFGYDVSNPNEMDKIRRMTGVCPQHDILFDDLTPREHLEFFAAIKGISNRQSAIEKIIREIDLLDKIDTASRSLSGGQKRKLSIGIALIGDPKIIILDEPTAGVDPYSRRHLWNVLQNVRRDKVILLTTHFMDEADILADRKAVVSKGKIRCCGSSLFLKNKFGIGYHLTFVLEDKSNENAINHLVLQFVKMARKDRRHGKELSFILPHNAVENFASLFAAIEHEISIKSDLGISSYGVSMTTLEEVFLGLQKEEEYGDATVYQDLPQSSEGISNENRALTEGIRSLEAFRCTPSTSQNLRTLIGLRLLRLRREKRKLWMVIILPIIFTGLGLYLNKVIDTGKKYEPPKTYPMPLDLSHYLGYNISIYNGSPADLTTFKNQLEKNGAELDTYDGEFTSLLDIAPHYASFNIHDFSSSSQNISVLYNGSYSNNLPMFINLISNTFYSMAATTGKIAVTTYPFELPPTNVSPTPASPGNFILGMIFLFAPIVLAVDMVYEREIKARNQLRVNGLPFTVYFTSFFLVQILLMATITILLIILIVVMKPPTFSNSSITVLAIWVILYCPASVLFCSCVSYMFDKSESAQSVMPNVSTLLGLIPYISVMYAKESVATILHYVFAFTDMMYVPYGMLYYIQKINLECISDPHCEGTTFSSFMIPEIIVLFVTLIIQIPLLFLIMLILDVKKNGGQVLDIFRSKKISENIVEESRDVGVHEDRDVKNERQRVNNLIRDPQNNRSVITVENLHKVYQKGVKSGICSRSAESLKVAIKSISLAVDSAEVFGLLGHNGAGKTTAMKIITAEEAPTRGRVQIVGRNITSSANTAFQYLGYCPQHDAQWKNITVKEHLELYSHIRGIPKNEIKRTVDLYLTGLQIHEHKDKQAEKCSGGTRRKLSYAMAMIGNPKIVLLDEPSTGMDPQSKRFLWDTVLASFRGSRGAILTTHSMEEADALCSRIGIMVNGELRCLGSSQHLKNLYGAGYNLEVKLATQQGTDLRQKLKELENYVFHLFPNAVLQETFGDRLIFSVPQQSVPSLANCFRSLEDGKRRLNIEEYSFSQTTLEQVFLKFAQEGEHED

>DvvABC-B_21313

MGNKKNENDLKCEKNKTNEISYLRLFQYCTTLDIIFMSIGAICAALSGIVQPYSMTLFGDVTGAIVTYASNYNESLSEPEKTLLADELINAVWLFGMKSVGVGIGVILTTYISTVLFIYSASRQIFKIRKAFLEKTLNQDIAWFDQNRTGDFASTFTQNISKLEEGIGEKIGTFLFFESTFVAGCVLGLVKGWKLALVCMVSLPLSTTIMTIISWISTKFSAQEMESYGEAGTIAEEVFTAIKTVVAFDGQDKEIERYNKHLVDAKKNNLKRSFFTAVSNGCLWFFVYACYALSFWYGVTLILNDRHLPEHEQVYTPANMVSVFFSTLIATWNFGLGAPLLETFGTAKGAAQKIFFVLDSKPVIRKFANSGIVINDAALEVTFEDVHFSYPSRSDVKILRGINLTISKGETVALVGKSGCGKSTCIQLLQRFYDPDMGQIKINGIDIKQLNLDWLKQKISVVSQEPDLFSTTIAENIRYGKLNATQQEIENAAKKADIHTFIQTLPRGYQTVLGERGTQLSGGQKQKIAIARALVRTPEFLLLDEATSALDTTSEAEIQEALDSIRGTCTIIIVAHRLSTIRQASKIVVINEGKVLEMGTHTELMDMKGAYHNLVISQGLTETLEEKGNRRRSRKFSEANKSINEKEDEELEDTQINQPVAKNILWKVLKLNASEWFYILIGCLSSLITGASLPIYGLVFGGIMGIFANDNDGEVRSESNMYCLYFLILGVVTGVAMFWQTTSFSVAGEHLTLKIRSKTFEAMLRQEIGWYDQKSNGVGALCARLAGDAVAVQGAAGPQIGTTINFISTFILTCTFSFYFEWRTSFVLFSLCPVIFFSVYFEQKVLQEDATKNQKMLEASAKLAVEAIGNIRTVVSLGCEKVFMEQYIKELLPYQKMARKKSHYRGIIVGLARSLMLFAYVAGIRYGINLIISGDCPYGTIFIVCEVMIVGTWSVGNALSLSPNFQKGLVAACRIITLLERQPVVQNMPDALNFLWEDENVEYSEVYFSYPTRPSIPILKALNLLIPKGKTVALVGSSGCGKSTIIQLLERFYDPSYGKVEISDKNIRYVDLKSLRSQLGIVSQEPNLFDKTIAENIAYGANYKQVEMDAIIDAAKSANIHEFILNLPKGYETKVGSKGTQLSGGQKQRIAIARALLRNPKILLLDEATSALDNESEKIVQEALDNARKSRTCITIAHRLTTIQDADVICVLKEGNVVEMGTHKELLEKQGLYYKFYKLQSVESIS

>DvvABC-B_17742

MTEEKKHSXKDKEKIGIDAQFVNSEEPKEKIKNVSFPQMFRYASTYDKFLMVVGLISATGTGVLQPLNTILFGSLTGDIIAYATSIQINLPADQKKIAEDNFFDGIRYFALMNSLIGIGMFVFSYLATVTFNYSAMRQIFKIRSAYLKSILNQDVGWYDINQTGDFASRMSDDLFKFEDGIGEKVPVFWSFQVVFLTSLIIALVKGWELALICLTSLPATLITIGIVGLLTTKLAKNELEAYGAAGXIAEEALSLIRTITAFGGQKKEVDRYNKNLVEAKNNNIKRSMFSALGFGLLWFMIYASYALAFWYGVKLVLRDRTATNQIYTPSNMVTVFFSVMTGSMNFGIASPYIEAFGISKAAASKIFSVIDHKPTINLSKGNGKTLNVLIGNIQFKDVAFRYPSRKDVPILKGLSLNIKSGDTVALVGSSGCGKSTVIQLLQRLYDADSGEVTIDGKNIKEYDLTWLRSQIGVVGQEPILFGTSILENIRYGKDGVTEEDVIQAAKKANAHNFIKALPNGYNTLVGEKGAQLSGGQKQRIAIARALVRNPTLLLLDEATSALDNTSEAKVQAALDAASVECTTIIVAHRLSTIRGANKIIVLSQGVVVEEGTHEELMELKQEYYRLVTAQVKSSEQFEVAEKKKVVRAISLAESSTGSDHNIEATKEDNEDDFNENKDVSVFEILKMNAPEWPYILFAGLGSIVVGCGMPVFAVLFGSILGTLANGDPDFVRSETNKYCLYFVLGGLITMVSVFTQMYLLGIAGEKMTERVRSRLFKAMIYQEIGFFDKKTNGVGALCAKLSSDASNIQGATGIRVGTILQSIATFCLAIGLSMYYEWKLGLVTAAFTPVILIAMFFERRNTRGGNDSRDSALQKSTRTAVEAVGNIRTVASLGLEEKFQQLYESELMPHYKSSLKTVHWRAIVFGLSRSLLFFAYATAMYYGGFLIRDGLPYDRVFKVSQAQIMGTVSIANSLAFSPNFAKGVAAAKKVKSFLSRIPLIRDLPSSRQMVKASGNFSFSEIEFTYPTRPNVLILKGLNLDILNGKTVALVGESGCGKSTIIQLIERFYDPRSGEVKMDGVDLKDISLDSLRSHMGIVSQEPNLFNKSIAENIAYGDNSREVSMDEIIKAAKNANIHNFITGLPKGYETKLGEKAVQLSGGQKQRIAIARALVRNPKVLLLDEATSALDTESEKVVQEALDQAKKGRTCVTIAHRLTTIQDADLICVVANGVIAESGSHQELLQKEGLYYKLYTQKT

>DvvABC-B_19147

MNVYSVIVSSHNLSKSLKLLKLVNSRRYFSNMSQKISQNKTYISVEIPETTKGASRGFFSRIFKRNKEVKEKQEPRLKKSELKRLFSLAKPEKWKLTTAIGFLVVSSTVTMSIPYSLGKILDIIYVGSGDSDAARARLNQVCGILLGVFVLGAICNYARVYLMSTAGYRMTNALRRQVFGAILRQEQGWFDKRPTGELVNRLSADTQIVGSALSQNISDGLRSLVMVFAGTGMMLYMSPQLALVGLAIVPPVAVVAVLYGRFVRKISRKVQDSLADSTKVAEERIANIRTVKSFAQEPREITSYNISIENVLKYCYKEAKARALFYGMTGFSGHIIIISVLYYGGVMVSSNTITVGNLSSFLLYAAYIGISVGGLSSFYSELNKSLGAATRIWEIIDREPTIPTQGGLIPMTDVEGHIEFKNVKFCYPSRSDIEIFKSLVLDIQPGKTLAVVGPSGSGKSTLAALLLRLYDPIDGAVYIDNQNIKELDPAWIKKHIGTVSQEPILFSCSIKENILYGADDPQKINDDEFIRICKEANVYEFVQNLPEGFETVVGERGVMLSGGQKQRVAIARALIKNPKILLLDEATSALDAQSEHYVQEALDRVMKGRTVLTIAHRLSTIQNADIIAVLQNGQIVEQGKYEQLLQSNGPFRELVQHQTFSQLMDD

>DvvABC-B_39715

MATVLNLSNIPFKAINRHLKTVVYQKQCIFKVYGQNTKNVYLSQFYYTKEYSTKTEKKSNSSKGSGGILAGILSKIPSSAPKAQPIRGCFHPGASTLTRETISLQKQTPITGRQMVGAMLQYIWPENDKSIRDRVKLAVSLLIGAKVMNVCVPFIFKYSVDYLNVGNALNMETAPQTVATVATSLLLGYGIARAGAAGFNELRNAVFAKVAQHSIRKIAKNVFIHLHNLDLTFHLQRQTGALSKTIDRGSRGINFVLSAMVFNVVPTMFELALVSSILGIKCGAMFAGISLGCVGVYAMYTLSVTQWRTKFRVYMNKAENEAGNKAIDSLINYETVKYFNNEKYEAERYDSALQKYESASLKTASSLALLNFGQNAIFSAALSGIMILAANEIVKGNMTVGDLVMVNGLLFQLSIPLGFLGSVYREVRQALIDMQTMFTLMTMDSAVKSKPEAPYLHVDSKSSPIKFENVSFEYGPGKTIFKDLNLTIEPGKKVAIVGGSGSGKSTLVRLLYRFYEPSKGRILIGNQDIRDVDLDSLRRAIAIVPQDSVLFHDTIRHNLHYGDLQATEEEIMNAAKLAEIHSSIVTWPQGYNTQVGERGLKLSGGEKQRVAIARAILKNSPILVFDEATSSLDSITEHNILMALRNATKGRTSICIAHRLSTIMDADEIIVLENGQVADRGTHKSLLESHSGLYWKLWETQNQSHTEPEKKKSAV

>DvvABC-B_9796

MLNISYCPPNISVWDVWVDHGIPQCFMNTVTSSVLAIYILIAGSIQLHIYRRWGVESGTRFLPRGRLYYLQTFLILFIPVLEIVRFILTATVYDDKHIYGYMIVSLVLTTFAFPFSLWVLKVELYNILPSVQTRGHGIVLLLFWTLAFISENLAFINLTQENWWFKLKDLTDQLEMALFILRYIACLLIFFLGLKAPGIVNEVDYYMLGGHQRNVINPNNENASTWKNFWKKVKILAPFLWPKKNFTLQFKVLICFILLLGGRAVNLFVPIYQKLIVDSMEETVAKMLFRWDWVLIYVGLKFLQGGGTGGMGLLNNVRSFLWIRVQQYTTREVEVELFRHLHSLSLKWHLGRKTGEVLRVMDRGTDSINNLLNYIIFSIFPTIVDIIVAIVFFVSAFNIWFGLIVFTTMILYIVLTIVITEWRTKFQRRMNLADNETRSRSVDSLLNFETVKYYGAENYEVDAFREAVLKFQDEEFKSSITLNILNTVQNVIICGGLLAGSLLCVYMVVEPKTLKAGDYVLFATYIVQLYVPLNWFGTYYRAIQKNFVDMENMFDLLREEQEIIDAPGATNLSVPRGHVEFKNVSFGYLPEKLILKNITFSVPSGKTVALVGPSGSGKSTIIRLLFRFYDVDTGVIVIDGQNIKTVTQESLRRAIGVVPQDTVLFNNTVEYNIQYGRLTATVTDVIEAARGADIHEKILTFPEAYETKVGERGLRLSGGEKQRVAIARTLLKAPNIILLDEATSALDTQTERNIQESLNRMCANKTTIIVAHRLSTIIHADEILVLQEGEIVERGKHDHLIGQEGIYANMWRQQLENKDKESLENSSESAKSK

>DvvABC-B_13664*

AFTKLNMTATQVEDDQEKKQKNNVKFDNIAVNLKFKNKENTSTWSNFWKKLKILSPFLWPKKDFVLQLKVLVCFLLLGGGRVVNLFVPIYQKLIVDSMQGTVEKMVFRWDWVVVYVGLKFLQGGGTGGMGLLNNLRSFLWLKVQQYTTREVEVELFRHLHSLSLKWHLGRKTGEVLRVMDRGTDSINNLLNFLIFSILPTIVDITIAVIYFVSAFNVWFGLIVFTTMVLYIVLTIIITEWRTKYRRRMITAENETRTRSVDSLLNFETVKYYGTEKFEVQAFREAVLKFQVEEFKSNVTSNILSTVQNIIVCSGLLAGSLLCVYMIVEPHTLQPGDYVLFATYIVQLYIPLNMFGNYYSMIQRNFVNMENMFDLLRQEQEVVDPPDANDIVVSRGGVEFKDVTFRYLPEKVVLRNISFSVPSGKTVALVGPSGSGKSTIIRLLFRFYDVESGLIVIDGQHINTVTQESLRRSIGVVPQDTVLFNNTIKYNIQYGRLTASDADVIEAARGADIHDKILTFPESYETNVGERGLRLSGGEKQRVAIARTLLKAPNIILLDEATSALDTQTERNIQESLNRMCTNKTTIIVAHRLSTIIHADEILVLQEGSIVERGKHEDLITQGGIYASMWEQQLQNKDKEPAENKPESSEPKKRPPKR

>DvvABC-B_17837

MWRLIQQNLHNKLLFHNSAIFKKYFCQGHIKNYISRTVSQKPVPKSFIPSKVATFGVTLTGGLIIKLYISHNGVLCEAKKTRMAGYENKSDKNVKFDWARLWKYLKPHIWYFLAAIVGALAVALLNIQIPQVIGGVVNVLAKFSESRDSELFLNEMKRPVIKLISMYLAQSVCTFFYIFMLSNLGERMAYKMRTDLFESILKQDIAFFDQQRTGEIINRLTADVQDFKSSFKQIVSGGLRAATQIVGCSVSLIMLSPQMTFISLLCIPSVIAVGTVFGSLLRSVSRRAQAQVEKTTAVADEAVSNIRTVRAFAMEDQEKELFNTEADRAMVLNEDLGLGIGLFQAGTNMFINGMVLSTLYMGGYLLSTNQLSAGEVMAYLVASQTIQRSLAQISLLFGSVVRGVAAGSRVFEYINKTPKMALTGGKVLPYESVKGDIEFKNVCFAYPTRSQQIILQGFNLSVPSGKTVAIVGASGNGKSTVVALLERFYDVKDGSITLDGHDIRSLDPSWLRGRVLGLISQEPVLFGTTIMENIRYGKPDASDDEVKEAALLANADEFITSFPKGYNTPVGERGVTLSGGQKQRIAIARALLKNPVVLLLDEATSALDTESEKIVQQALERARTGRTVIVIAHRLSTIQNADLIVVLNKGKIVEMGTHESLKKLGGYYWSLAYQQQNSPAG

>DvvABC-C_41801

MDVATETKEISPEYEACLLSRIFFLWTIPFFRKILNGTLKLSYFAVNAEDDAKILLKQLEEKWAAEVKRSKENKEKPNLKKVVMGIFRKQLAIIYTFTGVNFVILRVITCVLLTILNRTFTEEYRFRERIMLGGMVVVLVVINYYSDYIFHYQLRKIAIRMKAGLSSLIYNKLLVLHLQSMEEATVGKIINILADDLDRIVQYTTYLGYLILTPLQILTLYASMWYLLGTAAFSGALFFTILLPIMVLGSKISSVYRLKASKIADSRVNMMKEIISGIKAIKMYVWELSFTKIINDYRKEELRNITKYLLFQIGYYHFAVLKLASAISVLTMLYVNGILFPEIIFSCIQFLVILKKGVLAFMPQAMLLQFEITNVFVRISELLLMNEVDIDSKINADVSGIQLDQIKASWSTKSSFVISSNNINIPSRSLTVIIGPVGSGKSTLLQLLLGELKLSSGNIQMNGSVSYSGQESWLFTSSLKNNILFGNDYDYNKYQQVVSACSLLPDFDQLRNGDLSCVGEQGVLLSGGQKARINLARAVYRDADIYLLDDPLSAVDSEVGKRIFDECVKSYLRDKTRLLVTHHLQYLQEADQIIIVDKGNLESFDSLSEIKFKKPEIFEWIQKLNKADETKETTVTSKLNLKDESDQKLNYVCNENRTKITPRRALFEYIKSGSCIIPLFLTVFSAQLLLGLTDYWLLYWTKQSYSTHAESKNVTENSNVHHMRDIQWTTEFNTNFTIYCSIVSSAFLMLILSNILLTKFFNTASKNMYKNMYKAVISAPLSFFYTHPSGRILNRFSSDTTAVDVRFADNLKDISQVYFMGIGSIILVIVSDYYMSFFVLIVFIYAKLLKWYQYIGSELKHLEITAKSPIYTYINNTLSGITTIRATKNQEKLQKEFIILLNNHTSVLRLNVLCGAAFSVIVDTVCAFFLFGVIFALILLNEYDHKITPGVAGLAILQVFQLMGALQFATQKSLDFVQYLMSIERMIEFSKIEPEEFSETKKNGVYNFQPGYSGAEVVFKNVSMFYNKGSLVLNGINVSIASGEKVGVVGRTGAGKSSLINVLLRLSEFSGSVQINGVDTKTMPLEILRKRVSIIPQDPVLFTNTVRYNLDPFGEFTDDNLWTVIKQVELKDCITSLDSVVTSGGNNFSTGEKQLICLARAILRKNELAILDEATANVDQRTDELIQRTLKMRFANFTLITIAHRLQTIMQTDKVLLMDNGYLVECDHPYRLILKRGKFYDLVQQTGDATAAHLEDMAYKHFTQHHS

>DvvABC-C_44708

MDSSKIKKRRLNPRDSANIWSLVTFSYITDLLKRGVQKDLEEEDLYEVSKNCASKHCAEQIEKKWKKGKKDGKYPSLFLTLCKVFGWKYFLWGITQLFCELFRSIFEPNAITNLVSYFQPGQTKLSKSDAYFNAAILLFSNFLQKIYFFNYDLFLFIMGIQVRTALCSLLFRKVLKLSPAAMTKASLGNIVTLITKDVQQIRRSMFAFNDIWVFTVVISVTCYLLYARLGVLTFFAVGMHFCIIPIEFFLGKMVTKMRNETSEMTDERIQVSQEVLSATKIIKMYTWEDYFYQKINLARTREVAKMVIVFYLRMILMLLGAISTKIGFYLMIMGYIWMEQPPDASIIFFITAHYDNIVIFFGYLLPEAVGRFAELSVTIKRVNRVLIAEELDQDHQSKQNNVKPYVELKNITVCVGKEELLSKISFKVDSGLTLVTGKVGSSKSTLLKAILKEYPLSSGEVVCSGTVSYASQHPWCFPSTIKQNILFGEKYNEKRYQEVLKVCALEYDLNLFEKGDETILTDNGQNLSKGQQARVNLSRAIYRQADIYLLDDCLSALDAHVHQFIFKKCIKEFLKEKICILVTQSKSTLTQADAVYVLDKGHIIGSYNPQENTEKEVNNFADTIYKTKEFLIKENGLMESMEVDRFLETEQSNYKNIYEEEMKKGAVDKSVYIKYISYGGGAALFTFALLLIGSKQAAESYSEKLISVWSDDKQKVLNIKANISNIIGQNITQLSTNLTQAEVQAASTFQMYTVMVILSLVLELLKTFAFLDFCKRASINIHKAMIKNVLHSVMAFFDTHFIGNILNRFSQDVINVDENLPFQLLNCLEVAINVGAAATLLMTVNPYFFFYISVTFGVMLLFLKLYLPITRNLRRLEASTRSPMIGHLNAALEGVTTVRAYKAENMIIDEYERHQDVFTSAHYSLLCFKFGMGFYMSMLAGVMVTLVICSFVLFETDATAGSIGLALTQVIYLGALIQGAVRCWADLETLMTATERALEYTGLKSETTQGSVPKHWPKDGTISFQNVSLSYNSTQRILNNLSFQVQAKEKIGIVGRTGAGKSSIISTIFRLYEVDGKITIDGVDIKLLPLKHLRKHLAIIPQDPILFSGTIRSNLDPFGEFQEKDLWEALEKANLKTSITNLDIKVSSYASNFSLGQKQMVCLARAILIKSKIVILDEATSTMDHETENLIQETIKHNFSDSTVLTIAHRLQSILECDKVLVLDRGQIKEFGTPKELLENKIGHFSKMVAQGDMSS

>DvvABC-C_48952*

MDSSKRNAKETNPRTKASIFRLLTFFYNYPLFKKARKKGLDDSDIYEIPKWLASEQLGNNLEDAWMRQRKKKGEDASLVRCLISCFGMQYLILGLIQLVVKTVLVFIQPRALSKVVAYYAPNQTDVTTKDLYIYASLVVGLNVFSVIYNHNYQQFTTEVGIRVRTSVAALVYRKAVKLGPNAWNHVTVGKIVTLITKDVFAFEMALIFVNDMWIGVIQTIIITGVIFNRIQWSVFGGIGFYMLTIPLQLFVGKVVSSKRVQSAKRTDERLQLTTETIRNIKTIKMYTWENFFTTKLNELRKLELQNLSPVFYLKSLVLIVGSTATSLSFFFMIMTYIWSGHFTDAETVYFIQTCYQSLKSFITVSIPIGIAQCSDLRASLKRLSHFLKLEEVVDRRSQTISPRVYMRHVSAKVGEKTILNDISFSAEKGVNLITGNIGSGKSSIIKVLLGEYPVSSGQMAIDGTISYASEEPWLFPSTLRQNILFGEPYNEKRYNEVLKLCALNIDLKKLPKGDKTIVGDRGVNFSKGQQSRINLARAIYRISDIYLIDECLAGLDSKVNYYIFRNCIMDFLKDKVVIMVSNNINHIKLLYGNNTLVVEDGRTLSLEKQKETLDKRITYFIDDVEMNYFDDDIDVTDEDILEDEANERTQLIGGDKNENKNLYDEEKKSGTVSLKVYLRYYKYAGGIIMLVILAIIFIAAQAALSYSEKLVSKWVNLEPNITNLTLSNQTDTEEYINIINKRDNYLVMYTFLTVVMVVLTFTRIYMNFFFAIRASRNLHRKMLKSVINAFMSFFDKHFVGNVVNRFSKDLMTMDEVIPLNTYEIFRQTLGLLGILYLIISVNKLFIIPSIFLFVKLYFIQKFYLPTGRGIKRLDAATRSPMIGYLNATLEGITAVRAFEKQPLLISEFDQHLDHYTSTSFMMTCAIRFYGFIMDMVSTTFFAAIVIKFVCFKTDAQAGDVGLAITQAMLLTGFLQYVIRQYTEIENNMTGIERVLEYTDIESEDKLQGRTLKNWPSFGEIKYENVSLVYNSSEQRVLKNISFTIKSKEKIGIVGRTGAGKSSIISVLFRLYDIEGRILIDNEDIKNLCLKYLRSSIGIIPQDPILFSGSIRANMDPSGTHTDAEIWSAIEKVHLKNLFTCLDDVIVENGSRYSSGQRQLICLARALVSKNKIIVLDEATSNMDPDTCSLLQRTIKTHFSQCTVlTIAHKLNTVLDCDRIMVVDHGEIIEFDTPQALRTKQGGIFNKMIEHS

>DvvABC-C_17573

MDHCEKVQRPPHPRENANIFSLLTFGYTGKLFTRGFKQDLEDDDLYDVIKKCRSKKCTDKLEHQYNSRSKTKDPNKKVSIFKIIWHMYGLRYILLGLFHMSGRLLTSTLEPDALSHLVGYFKPGQTKMTIHDAMYFAGIMIGVKAFHTIYFQNYHIYLTELALQIRISFSSLIYRKALKLSPKALEDTSLGNIVTVINKDVQQFEHSIWMFNDLWISVLQTFVMCYLIYQRTGVASVVAILMLVLVIPVQGYVAKIIKNLRLKMSRRTDERLQRTQESLSTIKTIKMYTWEQVFADKIGEARFKELKILLKSAYAKISLMIMSSLVGKFAFYAMLMIYLYIHEDMSAEDIFYIMRIFGTLRFTMAMAFSMGFTRIGELSASLKRINRILELEELPDVIDKPDDDPQIDLRNVSVNLRNKDILKNVDLKLEKGLNVLTGQLGCGKSSLIKVVLRDYPILDGGEVRTRGRKSYASQDPWLFPSSIKQNILFGEKYDFKKYQQVVAACALEYDFKILEKGDDTIVADRGMNLSKGQQARINLARAIYRDSDIYLIDDALTALDTRVQEQIFTQCIQGLLKDKCVVLVTHNAKHIHAADKLVILHDGAIKYIGDQANATEDILLEALEDEEIEEVVMETEESKVIDEKTELLEKPQLRKRQVYHENKKQGSVDFDLYIQYIKMGGGFIFAILLLFTFAGATLTESTSQKMLTNWINEKSTIQGLKEKHLKNTSINFEDIDLTNQTAIYYNSTYNISAEVIRNIGRLEVKATKSLNLYTILVIGYSFVELLKRYIILRVGFRASVNLHKKMVTSIVHSTMAFFDSFFIGNILNRFSQDLSIVDEHLSMMMSHMVDALFHLVGVVGLIATINWKFIIPAVVLAVFSLLLRSIYIRTSRSLKRLEAATRSPLVGHLNSTMEGLTTIRAYKAQDILKNEFDRHQDLFSSAFYTSICAKAAFSFLMEISSIAFTTTVIVRFLFFDTGTNSGDVGLTLNQAGMLSGIVHMGLAAWSELENSMTSVERAMEYTTIEGESNTGTDNIKWPTNGEIVYQDVSMTYTNSHEKVLKDISFTVKAGTKIGIVGRTGAGKSSIISTLYRLYNYQGKIFVDGVELKQLSLKFLRQHISIIPQDPIMFSGTVRSNIDPLKEFSDEEIWKTLHKVQLDSVVPKLEVDVDDVNFSTGQRQLICLARAIIRKNKIMVLDEATANMDPETEQVAQKIIEENFSSCTVLIIAHRLDAILDCDKILVLDKGNVIEFDSPKVLLDNKTSLFSEMMRNSHLGDGEEKQKSN

>DvvABC-C_51687

MANLTELTTLPTTVTELPKLTSNSSQQNATIEEDAYAALSSFCGSPFWDTNLTWNTNDPEFTPCFEKTVLVWIPCIFLWVFAGLEVFYIFNSKRRNIPWNWLNLSKLAITATLIVLTISDIVNSFKTAGNRGSEVNDVDIYSPLIKILTFALTGVFIFYNRKHGLQTSGLLFLFWFFVVLCAIPQFRTEIRRSQRNEILPDYYYFYTSYLIYFPLVVLMFLLNCFADKAPLERKYPQSQNPSPEESASFLSRLLFAWFDPLAWKGFRKPLETKDLWDINVEDSSRELVPVFEKYWSQTLKKAESTSPTQVAHAKFKSDSASVDFVNNKKKKQASILPALFKCFGPIYFFGALLKLIQDLLTFVSPQILGFLISYTRFNQEMWKGYLYAVLLFITATIQTLFLAQYFNRMFVVGMRIRTVLVSTIYRKALKISNSARKESTVGEIVNLMAVDAQKFMDLIGYLNMIWSAPLQICLSLYFLWKELGPSVLAGLAVMIILIPVNGFIANKIKKLQVKQMKNKDERVKLMNEILNGIKVLKLYAWEHSFEDQVLKIRNKEIKVLKQAAYLNAGTSFIWSCAPFLVSLVTFATYVLVDEKHVLDANKAYVSISLFNIIRFPLSMLPMMLSNLVQTWVSVKRINKFMNAEELDPDNVLHEPSEEPLTIENGTFSWGEGPILKDININIKKKTLTAIVGTVGSGKSSLISAFLGEMDKLSGRVNSYGKVAYVSQQAWIQNATLRDNILFGKPYDKTLYDAVVEACALNPDLEMLPAGDQTEIGEKGINLSGGQKQRVSVARAVYSNADIYFLDDPLSAVDSHVGKHIFEKVIGPHGLLKSKTKILVTHGITYLPQTEKIIVLKDGRISETGTYQELLDRKGAFAEFLLQHITEEAETEAELDELKDQLADTPLSQEVARQLVRHRSRVSESQSETGSDHIGNGSIQRLNSVDKSNHKLSIDDGKKGPKKGEKLIETEKAETGNVSWAVYKHYLKSIGLVFMLATLIFNLVYQGFSVGSNVWLGLWSDDQKIVVNDTVDTARRDMYLGVYGALGLGQVVTILFASLALYVGSLNSARALHNLILGNVLKAPCTTFFDVTPLGRILNRFSKDIDTLDNVLPMTMRGWITCFYSVVGTLAVISYSTPVFILVIIPLCFIYYFMQRFYVATSRQVKRLESVSRSPIYSHFGESVSGAHAIRAYNQQERFIVESENKVDMNQICYYPGIISNRWLAVRLEMIGNLIIFFAALFAVLKKDQAPGLVGLSVTYSLQITQTLNWLVRMTSDVETNIVAVERIKEYGEAPQEAAWEIPSKQTSPAWPEEGTVQFKNYSVRYRPGLDLVLHDLDFEIKGGEKVGIVGRTGAGKSSLTLALFRIIEAAQGNILIDGIRIDELGLHTLRSRLTIIPQDAVLFSGTLRINLDPFNKHSDEDVWRSLEHAHLKEFVKGLPAGLNHEVTEGGENLSVGQRQLICLARALLRKTKILILDEATAAVDLETDDLIQKTIRTEFKECTVLTIAHRLNTIMDSDRVIVLDKGRIAEFDSPARLLANENSIFASMSRDAGLA

>DvvABC-C_21020*

MEEVSFLEQKKRKRHPIETTNCVSKLFFCWLPVYLFRGLKNEVTEDDMYVTVEKQKSLYLGTKLEQAWKKQLKKKRPSLLLAIGSVFKWELAIYAVFNAWFELLRIAQPFLISKLVSYFQDGAKSDNIVVCAITLIIVTFAQVVSIHHYQLKVMVLGMKIRVAACALIYRKALKLSKTALSQTTVGQMVNLLSNDVGRFDYSGQHIHYLWLSPCVGIACAILLYKETGITGLSGSMFLLCFVPAQVYMAKLTSQFRLKTALKTDERIRLMNEIISGIQVIKMYTWEKPFAKLIEFIRKKEIDQIRHTSIIKALTITFNIALSRAAILVCILTYILSGNVLTASYAFTVTSYYTYMRAIITLQFPQAMTQFAETLVSISRIQKFLLFEELDVKYTPMHKQINNNEILQNNKPDVIEVRPVGIKIKNAAVKWIKKHPENTIEQINFEARSNQLVALVGPVGAGKSTLLQVILKELKPLEGTVKVTGTVSFASQEPWVFASSVRQNILFGETYNENKYYEVLRVCALEKDLKLFPHGDRTLIGERGTSLSGGQRARINLARAIYKEADIYLLDDPLSAVDTQVGKQIFNRCICNYLRKKCVVLVTHQLQYLYRAKFIYLIDDGRIRASGTFENLKNGDNAFTKLLATATEMDQLEHDRKLSKSESISSINYEEEDYISIDQQKETIGSGGVSWRVYGNYLKAGGNLFKTLILALSFVGTQVLVSLSDIFLTLWVNIEQWRRKQAVFINETSNSSNLDTILKYKDQSTESYWPHVMDQISPLHIYSFLVIATVILAVARSLASFSYFLTASTNVHNAMFRKIISSPMLFFNSNPSGRILNRFSKDIGILDELLPLATADSTWVGLTVVATTIVISLLNPWILIPTAVIVCIFYKIKQIFLVSSRNIKRIEAVTRSPIFTHLAASLQGLPTIRAFGAEQILTQEFDNFQDAYTSSYFMFLTASRGFGFWLDLHCAFYISLVVISIVFSQSDSLGGNVGLSLTQAITLSGMFQWVMREWSELENQMTSVERTQEYTDLPIESDNERKQPPADWPSSGSLTFKNMSLRYSLEAPCVLKNLNFEIKPREKIGIVGRTGAGKSSLIQALFRLAINEGSLIIDNVDINTVELKVLRSKISIIPQEPVLFSGTLRKNLDPFDDYSDKVLWAALDEVELKHAVEEMRHNLYGKMAEGGSNFGVGQRQLLCLARAIIRNNKILVLDEATANVDPMTDAIIQKTIREKFAECTVLTIAHRLNTVMDSNKVLVMNAGEAVEFDHPYTLLQNKKTIFYGLVRNTGKNMAAHL

>DvvABC-C_222633*

MDHCRKEQKKTNPRETANIFSLLTFAYVGSLFKKAFKLDLHEDDIYEVLNQHRAKKCGDLLEREWLSEKNRSKKPSVSRVMWRCYGKRYLFRGFIDFVFNTINSIIRPYVIAHFVAYFAPGQTVLTRNDAYFYGCGILVIHFVALFYNHNYMIWIQMFAIEMRTAFSSLLYRKALKLSSAGTTDTNLGNIVTLITRDVQAFVTVIFSVNDAWVSITQVLVICYLLFNKVGVVSFVGIGILISVFPFQVYISTWVTKFRLQTCQKSDERLQLSQEILSTIRIIKMYTWELFFTQKVTKARKEEMRKLVLGFYCRRVIIITGVMFLNFGFYMLIMACIWAGVSTDTTIIFYILSNFRDLRHFLAIVMPMGMGNVSEFVSSYKRLVKALKSEELVEEITQEDPKSPPFIKLKEATVKVRGHEILKGVSFQISPGLTVITGPIGCGKSTLFKTILKDIPLESGSVLTQGRISYASQDPWLFPSTIRQNILFGEPYQAKRYNEVITACALTFDLSLFEHGDETILTDRGLNLSKGQQARINLARAIYRSSDIYLLDDCLTALDASVQVYIFNECIKKFLKNKSCVLVSQNPSHIQQADYVIILEDGTVKDVGKPSEQIIREAKEVVDHGVDLQKQEKQQIKEDNSADFDNEVTDDIKLLEKDEKSTKKIYSEVKKQGSVDWRVYKQYFIYGGGFLFVLFNISMRGSTQFAVSYAERLLTKWVDKKQIVLNIEKNISTNPLSNDTLFPSLSLAKSIEQDTFRLYSVFVMLGTGMELLTTYALLEFCRRASVNLHKAMIKKIINSVMMFFDTHLIGNILNRLSQDMLSVDEYLSAVLDQCCRTIFNVGGIVFLVTLVNPSGAFIIALIFALAFLMRKMYLPAGRSLKRLEAASRSPMIGHLNATLEGVTTVRASKVQGILIEEYNRHLDLYTSASFTSMCSARAFGFFIDLICSLFIVFVVIKFLFFNKDNTAGDIGLTITQVSALSISITWCVRQFTELENYMTSLERILEYTNIKTENKEGAEINNWPSLGSISYQNVYLSYEGNTHYVLKDLNFEIKPTEKIGIVGRTGAGKSSIISTLFRLYEVQGKILIDGVDIKTLSLDFLRKHIVVIPQDPTIFSGTIRSNLDPLNEFEDKDLWNALQTVGINDSITNLEQPTNSNLLTFSSGQKQLLCLARALLRKNKIVVMDEATANMDHETDKLLHKIIKENFFDCTILTIAHRLHSVLGCDKVMVLDRG

>DvvABC-C_18126

MDIGFKLKHENPKKQAGLVKKLFFGWLVKLVKQGTKKQLEISDLYEPLDKDNSKTLGDCLERHWKNEILKSQIKKTSPSLLKAIVKAFYFEFLLYGIAWFVLNVLLRCSQPIILFHFIALFSGENREENQGDMYIYGGLLILVSVLSIFFMHHLQIGLASIGMRVRVACSSLVYRKITKLSHQTLGQTAVGQVVNLMSNDVHRFDLVLLPLHAFWAIPFQFVILSYFIWQQVQIASLAGLVSMVIISLPLQGYLGKLMGTLRANIAKKTDNRVKLMSEIISGIQVIKMYAWEKPFEKVIEIARKNEIRCVTLTSYLRGIFASCMVFLERMSLCFTLICYVLLGNNITAEKVFSLAQAFNILQLSMAIWYPLAVSHGAEALISIKRLKAFLTLEEKEVSRIKGLSTPGVVMSNVSSSWCDAGETLQDISLNIPPGFLCVVIGPLGAGKSSLLQLLLGELAIKTGTVLMGGEISYCSQEPWLFQSTIRNNILFGRPFDKQLYEKVVKVCALERDFQQFPERDETVVGERGVSLSGGQRARINLARAIYRQADVYLLDDPLSAVDTHVGKHLFNQCIVKHLRGKTRILVTHQLQYLKKANLIVVLNEGKIETVGTFEQLSRSKLDFAKIIVDSVEPGDKHEETTESSDLTNTVSNPRKASVTSTKSDLSESLEYFEENNFSDEVIEHDPQHTAWKEYFQATRRTGLLIVVFTMLVLAQTICSGTDLWVAFWTNQEVIRHSTSRPLESDVSSDEPILYEATPFDSSLNLTHNYEYKVEEPVVTSDGIFDYVYINNHVYHLVKTGYAIGFYGFLIVGVIILTLFRSMMFVKVCMIASVNIHSKMFSTLLRAPMRFFDTNASGRILNRFSKDMGSIDEILPRVLLESVQIFLVLIGILVNVSISSPYAIIAMLLLGICFLKLRSCYLSIAMSLKHIEGKVKSPMFSHVNSSLHGMATIRASNTEDILIKEFDEHQNVHTSAWYLTIVCISAFGLWMDIVCIIFLTCVILTFIFLQNFFKVNSSLVGLAISQSMTLTGMLQYGMKQTAEVINQLTAVERVLQYTHINTEGPFESPIETRPVEPWPKFGRVDYNHVYLKYSEDDPPVLRNVQFTILPGQKVGIVGRTGAGKSSLVAALFRLVNFQGTISIDGIDTKRMGLTYLRKKISIIPQEPVLFSATLRNNLDPFEEFTDEQIWKALEQVELKDISHSLDLMICEGGSNFSLGQRQLICLARAVLRNNRILVLDEATANVDHRTDSLIQTTIRNKFKDYTVITIAHRLNTIMDYDKVIVMSYGRVVEFGHPHQLLQLPDGHFHRMVLETGPVMSLQLKDVAMVAYSNLLKQEAASIR

>DvvABC-C_49513

MDSTEVQNNPNPRETAGILSIMFFGFTIPTFRTGMKKQIGVEDVYNTLRSDRSEYLGDRLEKNWNRELELVKQNKKEKPSLFKAVVRTFWLEYLLIGIIAAVSDIGIRSFQPSMLGGVLDYYTPGSKKSKLEAYFYGSGMVIMSLTSSILMNQYMTNAAHAGMKVRSSVCALIYRKATRLSRTALGDTPAGKVVNLLSNDVARFDFASMMIHQLWIGPVSTIPVIYFLYQRAQLPGIAGILVILIMSPLQATSAKLATKFRKMTALRTDERIRLMDEVILGIQVIKMYAWEIPFQKVIGLARKAEIKIIKKNSYIRALFMTLGMVTTKLALFATLVTITLTGGEITAAKVFVLISYFNMLSMTLTGILTRGIAEIAEMFTSIARLQKFLENDEYKYDQNKISSDPNENSKSMLSLKNIYAKWDASLSDCVLNDITLELNKGKLLGVIGPVGAGKSSLLQTLLGELDIDKGSINANGTYSYASQEPWIFSSTIRQNILFGADYDRKRYKEVIRVCALERDLEQFKDRDMTVVGDKGASLSGGQKARINLARAVYREKDIYLLDDPLSAVDIHVSKVLYDECINGFLKNKTRVLVTHQVHYLKNASHIIILNNGRIEGEGSFNDLANSDNLYAQLLTKEPEITEEEKVKQIEFARQVSRQSRKSRKSSMTSIISEKTIVEGLMEDSDEDSEDEENTKKLEMRDMQEESSKGKVLGSLLLRYFRTGANCFNVFLILSLYVLVQVSSSMVDYFLSIWTNIEDERTSASILLESNSSSTLLEDISTTIASNNINSTVVLADFPSTIPSTTLSDAALNTLNTTVKLEPQAWIQFPAWTTEECLYIYGILISISILMTLGRSFSFYSLVMSCSRNLHQILFKGVIYAKMRFFDTNPSGRVLNRFAKDIGSVDESIPRVLLDGGQMILQIFGSLILVGVANPYAIAMALILAIAAFFLRNIYLKTSKNLKRVEGILKSPVFTHLSATLQGLPTIRALRTEKILQAEFDKIQNYHISAGFMNFTCGAAFGISLSMCSNIFIAVLIFSILTFDKAMGLTGGTVGLAVSQASTLTGMIQMAMRMTADISNQMMSVERVLEYEQLPPEPQPKNPKPLPKNWPSSGKIDFQNVKLQYFEGGPTIIKDLSLNVKATEKVGIVGRTGAGKSSLIAALFRLANVEGKIAIDDIDTRDIYLRDLRSKISIIPQDPVLFSGTLRYNLDPFEEYSDEQLYKVLDEVELKDPNNIINRLENRVMDRGSNYSVGQRQLICLARAIIRNNRILMLDEATANVDPQTDALIQKTIRTKFANCTVLTVAHRLNTIMDSDKVLVMDQGTAAEFDHPHLLLQNSNGIFSKMVEDTGPGTCEQLKTVAKNTFDKNLLIEE

>DvvABC-C_14070

MDVVFKLKTENPKRRANFLSKLLFAWLVPIIKKGNKQTLEVVDLYEALDADKSKRLGDALEKHWDRQVLLAKEKSKKPSLMWALWKTFRLELIMYGILWCIQNVVLTSLKPLLIAQLVALFINYSDDVVTDIYIYSAAFIGVNVLIVFIYHHTAFGLQALGMKIRVAISSLVYRKITKLNQKALGQTAAGQIANLLSNDVMRFDLVVVPLHALWVMPIQVAVLLFIMWQQVGISSVAGVVAMAVVSLPVQGYLARLQGKFRERISKKTDKRVKVMSEVVSGIQVIKMYAWEKPFEAVIKLARGTEVKDITAASYLRGVYSSCMVFLDRMALFFTIVCYILLGNLITADIVFSLAQTFNILQMAMAIYWPWAVSQGAEALVSVKRVQEFLVMEEKEESKIDDLGKSGVILSKVNASWTPTTSTLQDISLQIPSGTLCAVVGPVGAGKSSLLQLLLGELPTKSGKVSIGGEISYSSQEPWLFQSSVRKNILFGKPYEKTWYDKVVKVCALERDFEQFPHKDRTIVGEKGVSLSGGQRARINLARAIYRNADVYLLDDPLSAVDTHVGKHLFENCIVNHLRGKTRILVTHQIQYLKKANLIVVVNDGKIEAQGTFNELMEYNLDFTKLLVAADETDDKEEDADSEGKLGKSGRASARSRRKSSAVSALSDFSESMYDDPEVIEEESGNTNIGTTFKQYILSTKSLCFVTFTTILLVIAQGINVLVDLWVTYWTSQEQIRHSNGTVISLQSADINEVELYPINQTTKIDATHRLVYNIENNGSQILKQISFDDIFDKIDLHGQIQKIIKTDYAIYIYSALMALAIILTIIRSFLFYKCCMLSSANLHKSIFHTLLKAPMRFFDTNPSGRILNRFSKDMGAIDEVLPKVLLDSCQIFVVMAGILVSITVSNYYILILLVPMGYIFLKFKDWYVATAKVLKHIEGITKSPVFSYVNSTLNGITTIRAANAQNILKDEFDENQDAHTSAWYLTIGCMSSFGLWLDIVSIIFMACVIGCFIILTKFTNVDGSLVGLAVSQGSVLIGLSQYGVRQLADFINQLTSVERTLQYTKIETEGPFETPKEKIPKGVWPKEGLLEFRNLSLIYVVNDPPVLNNLNFVIKPGQKVGIVGRTGAGKSSLIAALFRLAPTKGSIFVDGVDTKNLGLTDLRKKISIIPQEPVLFSATLRYNLDPFNEFDDEKIWKALEQVDLRESIDSLDFQVAEGGGNFSLGQRQLVCLARAVLKNNKILVLDEATANVDPRTDALIQATIRKRFKDCTVLTIAHRLNTIMDSDKVLVMSFGSMIEFDHPHNLLEIPDGHFHRMVLETGAGMTQQLKSIAKAAYNNKIE

>DvvABC-C_22628

MDSTKKHVKVSPEESASPFSVLFYWWILPFLKYGYQNDIGMKDVYNTTQADQSGPLGDELQENWEREILSYIDKQKNKPSLKNAIFRTFWKSFILSGAAIFVQFIIIKTLQPVVLAKYINFFDTNNKPYLGWIWGCGVVLLALANVVLYHSTMLATQRIGMRIRTAVSSLTYRKLLKLNHKSLGETAAGQLVNLMSNDVQRFDVCAASIHFIWIMPIYAVLTFYILYIYVGIIAAVTGMAFISLESIPLQGTISRWQGVLRYKIALRTDKRIKLMSELTSGIQVIKMYAWEKPFEKIVEMSRKYEIDVIAKTSYLYGILSATSVFTERLILYVTLIPFVLLGHRLTGGIAFSLANLFNNIQLVMAINFPRALSSYNEANVSIARLEKFLLLEEVEEETVVNEKHGDHVGCINLNNVTASWSPKSIVPTLIDIDLHLRCGTLCCVVGNVGSGKSSLLQLFLRELPITSGYMNIAGKISYASQEPWLFVSNVKDNILFGKSFNKKRYQDVIKVCSLERDLKQLPYGDKTLVGERGKSLSGGQKARINLARAVYTEADIYLFDDPLSAVDTKVGKHLFDECITKYLNGKTRILVTHQLQYMKKSDLIVIINNGKIDKVAKFHDLTEHELNLLQQTPEIDDKDKEKMPTILETKIPKVSSTATLQSASSLASSIPTEEPGETGELIEKGDLSTSLYWEYFRSGTGIGFLLFTGFMFIFSQIITNASDLWLSHWTNVEARRYVSTLNLSTEFISTTISSINNHTVTDMLKSVAFTNTNPQEIITTADPSVSASKLSSNMSSLELENFKSEGYYIWVYSALIIAVVILQIWRCFLYYQVCMSSSKALHNKMFHNILQAPMRFFDTNPSGRILNRFSKDMGAVDELLPSCQIDAIQILMVFVGILVMVVIVNPWMILTTIIIGPVLFILRKMYLKTAQSVKRLEGISKAPVFSHISASLFGITTIRASNAEKMVTTEFDILQDQHSSTWFLFLVSGRVFGFYLDVICCIFLAIVTIQFLLFRDENTLSGNVGLAISHSYILTGMVQMGIRQSVEVASHMISVERILQYTKLEKDGVFESLPAKKPPRDWPNKGKIIFKNTFLRYALNMTPSLRDLSIDIKSGEKVGIVGRTGAGKSTLIASLFRLAPVDGEIIIDDIETGGIGLHDLRTNISIIPQDPVLFSASVRYNLDPFEKHSDEILWKALENVELKGVVTDLNQPVSEGGSNFSAGQRQLICLARAIVRNNKILVMDEATANVDPQTDALIQKTIRERFRDCTVLTVAHRLNTIMDSDRVLVMDAGQAVEFDHAYRLLQNSDGYFAKFLKDAGPPMANKLRDIAKEDYYKKHTSPEVIVDDD

>DvvABC-C_20002

MDIGYKLKAENPKQNANIFSKIFFGWMIPLIRRGTKQNLEINDLYKTLKKDQSKRLTDALEKNWQKQVDKAKKKGGKPSVLMAISRTFAFEFMMYGILWAIQNVVLMSLKPILIAQLIELFTDDTSTRFREMYFFSTSLILVSLLIVFFFHHTNFGLQAIGMRIRVATSSLIYRKITRLNQKSLGETATGQIVNLLSNDVQRFDMVVVPLHALWVMPLQVAILMFIIWNQVGISSLAGVISMAIIALPVQGYMAKLMGMLRQKVSGKTDTRVKLMNEVIGGIQVIKMYAWEKPFEKVIKQARSSEIGDITKASYLRGVFSSFIVFLDRVALFFTVMTYVLLGNVISADIVFSLAQTFNILQTAMAIWYPVAISVGAEALVSAKRIQNFLIMEEREEASIEKIDKPGIILSNVFASWTTKGRTLQDISFQVPPGTLCAVVGPVGAGKSSLLQLLLGELPLKHGRVQLGGEVSYSSQEPWLFQSTVRNNILFGSPYEKRWYEKVVKVCALERDMEQFPQGDKTIVGEKGVSLSGGQRARINLARAIYRQADVYLMDDPLSAVDTHVGRHLFDQCILHHLRGKTRILVTHQLQYLKKAGLIVVLNDGKIEAQGTFEELMDSKMDFTKLLVAADETGEKHDKQEDADPEPVDFTRKFSSTRRFSVLSDASGEISLSMRSVDMTSDKNGEEEESAGDGKPFKDYLFATKNICFVIFVCVLMIMAQAFVVGVDLWLTFWTSQEAIRHENGTIVESTSPTVEIIPLHGDNFSYSYNYNYSYIPENNTNSFNNTKSFNINDIFDTVNVDGQLKKIIKTNWALYFYSGLIGLAIVFTLTRSLLFFKGCMMASVNLHSSMFHMLLKAPMRFFDTNPSGRILNRFSKDMGAIDELLPMGFLDTMQIMLALCGILVNITVSNAYIVIAIAILAAVFLKFRSWYISSARVLKHLEGITKSPVFSHINATLNGIITIRASNAQDVLIEEFDENQDANTSAWYLTIACMNSFGLWLDFLAIIFLAIVTFCFVILRKFTDVNGSLVGLAVSQCSALTGMLQFGMRQTAEIINQLTSVERVMQYTKLDTEGPFDTPEENRPRGVWPKRGQIEFRNLSLKYVENDPPVLRNLNFMITPGQKIGIVGRTGAGKSSLISALFRLAPLEGAIYIDGVNTKNLGLTDLRRKVSIIPQEPVLFSASLRYNLDPFNEFDDDKIWDALEQVELRDSVDSLDFHVAEGGGNFSLGQRQLVCLARAVLKNNKVLVLDEATANVDPRTDALIQATIRKRFKDCTVLTIAHRLNTIMDSDKVLVMSFGNMIEFDHPHKLLQIPDGHFHKMLLETGPVMSAQLKDVAMRAYQQE

>DvvABC-C_7536

MDSTKKYVKVSPEQKANLISKLFYWWFLPFFKFGYKHDITIKDVYNTTQGDLSAALGDALQKNWEDEIRNHEDKKQKKPSLKNAILKTFWKSYSRSGIALGFQFVVIRMLQPIVLAEFINYFDSNQEAYIGWWLATGVIGMAFLNVIITHSCTLDTQRVGMRVRIAVCSLIYRKLLKLSHNSLGQTASGQLVNLLSNDVQRFDLAAQFIHYAWIMPLTAGISFYILYRYVGIIAAVTGMVFITLESLPLQGSFSKWQGKLRYKIALKTDKRVKLMSEITSGIQVIKMYAWEKPFEKVVELSRKYEIDIITTTSYCYGVLSAMGIFTERMILYITVITFVLVGQRLTGDVVFSLAQLFNTVQLIMAIFFPRALSFYSEAKVSITRLEDFLLLDENEKIPEPEKISNPDELGEIQLTNISASWASKPIAPTLMDLNLHIQPGTLCCVVGNVGCGKSSLLQLLLRELPASRGQMKINGKISYASQEPWLFVSNVKENILFGKPFLKNRYHDVVKVCSLERDFKQFPFGDRSLVGERGTSLSGGQRARINLARAVYTEADIYLFDDPLSAVDTKVARHLFDECISKYLYGKTRILVTHQLQFMKKADLIIIINNGQIEKIAKFNELSENDLNALQQEPEADDKEKEKVPETGDKIRKNSTVPHFQSMSSLASSVFSDDPNEEDELIEKGAISNATYVEYWKSGGGVLFLFFTIFIFLVAQMITNASDLWLTHWTNNEAKRYHLSDSLHNATSNKTLERPLLMSTGLSDEMSTSATLLTDSTPSADSSLLSLSQKRYLTDhIRNATLEELNDIPSTEYYIYVYTGIILASVVFLTWRSFLYYQICMTASKVLHNKMFNNVLQAPMRFFDTNPSGRILNRFSKDMGAVDELLPRCQIDAIQIFMVMIGILAMVFIVTPWMIIPAVILAPLYYFFRVVYLTSAQSLKRLEGVSRAPVFSHISASLYGITTIRASNAEKMITTEFDILQDQHTSTWYLFIVSSTAFGFYLDVMSCFFLAIVTYQFLLFRTENTLSANVGLVISQSLILTGMVQYGVRQSAEVASNMISVERVLQYTKLDKEGPFETLPGKKPPRNWPEKGRIIFKNTYLKYAPELPPVLKDLNIEVNSGEKIGIVGRTGAGKSTLIASLFRLAPIEGTISIDDVDTAEIGLNDLRLNISIIPQEPILFSASLRYNLDPFEKHGDEVLWKALEDVELKGAISDLNQVVSEGGSNFSAGQRQLICLARAIIRNNKVLVMDEATANVDPQTDGLIQKTIRERFQDCTVLTIAHRLNTIMDSDRVLVMDAGQAMEFDHPYQLLQNPEGYFSKMVQETGPAMAELLSNVAKNDYIKKKGtLTSEVPQNLAIEDNKD

>DvvABC-C_47333

MESSNSVPGAQTETEEPILRRSTRVRKPVTRLNLWFLPFFKYGYKNDVELKDIYNATKPDMSESLGNQLQKNWEEQIKKCDQSQNKKKPSLKSAIVKTYLKMYTASGVMIFLQFIVIRMLQPIVLAEYINFFDKKHGIKPNEFGWLLASGVVIMAFLNVILLHNTFLNTQRVGMRVRIAVGSLLYRKLLKLNHTSLGKTAAGQLVNLLSNDLQRFDYAAVFLHYIWVMPINSAISFYIMYRTCNLVAALTGMGAIIVEALPLQSYLSRLQGQLRGKIAVRTDTRIKLMSEITTGIQVIKMYAWEKPFEKVVELARRLEIDSITKSSYIHGMLSAMSVFTERFALFIVVITFVLMEGRISGDIVFSMAQLVNTVQMIFAMFFPRALSTYAEAKVSIKRIEEFLLLEETTPKKKLGENDTLAGKPGEIELFNVTASWSENPITPTLVDLNLHLTPGTLCCVVGNVGSGKSSTLHLLLKELSPTNGKVNVYGNVAYASQEPWLFVSNVRENILFGRPYDKKRYQEVIRVCSLERDLKELPHGDKTLVGERGAALSGGQKARVNLARAVYSDADIYLFDDPLSAVDTKVGRHLFDECMVKYLKNKTRILVTHQLQYMKKADIIVILNNGRIEKAANFSALSQDELNVLQQEPEPEEDENEVKKERSKVERSQSTISEKTLRSEHYQSNTSLSVSEEPEDKSNERGEEEVEQGTVPFSIFVEYFRSGAGVFFLTFTAVAFIAGQAFTSITDLWLTHWTNVEGKYYADMLAANVENTSETNRNNLTNTLTTLLPLTTSADPVLLTLPTLPTNISVMESMLTISSVIDTTTVRIEAEQVYDQNRYIIIYTILMFSSMILLTLRSFLYFIVCMSSSRTLHNKMFSNILQAPMRFFDTNPSGRILNRFSKDMGAIDEMLPKTGIDTIQIFTVLVGVLVLVFIVTPWMIIPTILIGFLFYYCRNIYLASAQSIKRWEGVAKAPVFSHVSATLQGISTIRASNSEEMVIKEFDVLQDQHTGTYVCFLVSSTAFGLYLDIISTLFLGLLTYQFLIFSVDSTAGGNVGLVLSQSLILTGMLQYGVRQTAEAISNMISVERVLQYTKLEKEGPFESLTKPPRDWPEKGKIIFKNTFLRYSLDVPPALKNLNIEVNSGEKVGIVGRTGAGKSTLIASLFRLAPIEGTISIDNLNTAEIGLKDLRLNVSIIPQEPVLFSESVRYNLDPFEKYSDEILWKAIESVELKHAITDLNQTVSEGGSNFSAGQRQLLCLARAIVRNNKVLVMDEATANVDPGTDALIQKTIRERFRDCTVLTIAHRLNTVMDSDRVLVMDAGEAVEFDHPFKLLQNPDGYFSRMLRETGPAMEEMLKAVAEEDYNKKYGGIKGKKGE

>DvvABC-C_49618*

VIKMYAWETPFAQLVATARRHEIKSLRATAFMRGIFLSFIMYSTRISLFVSILAYVLSGYDISAEKVFVITSFYSVLRQSLTVFFPQGIAQVAEVRISIQRLNKFMQFEEIDMVREKFGRCSMSSSKSLDLSKSGNGVEKSERKGELGVFLNNATAKWSEMSVDNTLTGITIKVYPSQLLGVIGPVGSGKSSLLHVILQELKLVYGSVTVNGTVSYASQEPWLFAGSVRQNILFGQPMDKLRYKTVVKKCALERDFRLLPYGDKTIVGDRGVSLSGGQRARINLARAVYKQADVYLLDDPLSAVDTHVGKEIFEQCITGYLKNKTVILVTHQIQYLQELDNILYLEDGIPKTGSFRELQASGLDFTKSLGESTEEEETTKESEVMRQLSIKSVASVEVEEPKEVQEQKGTGTIGGYVYRAYFRAGGNCCVIFIFFFLFLLSQMFASASDYYLSYWVNLEQQDSAANSTQSTGLSSNETITNNFWHFSRETSIYIYSGLIVLLVIITLVRSFTFFAVCMRASTRLHDNMFVSITRATMRFFNTNASGRILNRFSKDMGSIDELLPTALIDCLQIGLALLGIIVVVAVVTPFLMIPTVAIGIIFFFFRIFYLRTSRNIKRIEGTTRSPVFSHLNASLLGLTTIRAFQAEEILKKEFDNHQDLHSSAWYAFISTSRAFGYWLDLLCMVYVTLVTFSFLVIGNEKFGGNVGLAITQAIGLTGMFQWGMRQSTELENQMTSVERVLQYNDIEKEGALESQPDKKPKPSWPEKGKLEFRDLYLKYFPQDPFVLKDLSFVVKPKEKIGIVGRTGAGKSSLINALFQLTETIGDILIDEVNISQIGLHDLRTKISIIPQEPVLFSGTMRKNLDPFDEFSDAELWSVLEEVELKEAVENLVAGLNSKMSEGGTNFSVGQRQLVCLARAILRKNKLLVLDEATANVDPQTDALIQQTIRTKFAECTVLTIAHRLNTVMDSDKVLVMDAGTLKEFDHPHVLLQDKDGIFYGMVQQTGKTMAETLHQLAKYSYEAMSHLHPDSFR

>DvvABC-C_45163

MTSDVIGRNWNWTSLCGPEGFQIWSQQRHDFGQCFQQLCFQIPVLSLFAVLSSYYFGRQTGFVSRGKLQRYCIITRNVVSLLLMFYPIMHAYNTIMTKTPNRENASFLVCAVQAISWCSHFLYTVGLRSRLGKSQRGPTAMGIVWCMVFAMTIVSLRSAYLENSQRPTENTKMELGITLYYTVLQIIYALSLIPGGSTTTLNFPERYTEITERQPLINANAYGRFSEEGDPNDLGVAMEDTNWLSRLSFSWVKSLVNKGVEDKIVTSDDLYDLPDSISSDNNSNRLENYLRIEHDTDSSREVIVIQPRKSSSLLAALHRCYAWQFYSVGILRLVADCSNFAGPILLNKLVDFIEKRSIDIKWGYLYAVLLVVSTSMSALCDSHFNFKMSVIGLKMRGAIINTIYRKTLSVRSTVLMSKLSVGEIMNYMSTDTDRIVNSCPSFHAVWSIPFQLFVSLYLLYNQVGLAFLAGVMFSIILIPINKCIANKIGDLSTKMMEQKDGRVKLTSEILRGIKAIKLYVWEQHFIRLITRQRDAELKYLKGRKYLDALCVYFWATTPVVISILTFVTYVLMGNKLTAATVFTSIALLNMLVSPLNAFPWVLNGLTEAWVSLKRIQKLLDLPYMNLNEIYDHKELEDNTDQNTEISISNASFSWEKATPVVQVPKGKGKGKRTLSKRGQGESSVQRRETIIFKLRDITLKIKKGEFIGIMGTVGCGKSTLLSSILGELAVESGSLAVRQVETGFGLVTQQPWLQRGTVRDNILFGKPYEEKKYKDIIFACGLIEDMQILPSGDKTEVGDSGGTLSGGQKARIALARAIYQEKQVYLLDDILSAVDVKVGKHIFNHCIIGLLQGKTRLLCTHHVKYLLHADRILLLENGAIKSIGKPVDVLRNIDDTLPVDLELEDSIQSEYTSSECTLENSLCVDNAQEKDKDIELFKEVSERGNLDFTVIISYWKGISHFVSISILISMTLMQVSRNFTDWWLANGVTSPVTNSTNLTVYLATATDYIDYVEDDDNMSEFLKVYVELACVNTLFTLIRSFIFAYGGILAATKFHKVLLKTVLRARCTFFDVTPIGRIINRFSSDTYTVDDSLPFILNIFLAQLFGLLGSLFITMYGLPWICVFLIPLVPIYTYLLNQYRITSRELKRISSVTLSPIYNHFNETLQGLTTIRAMRVIHKFKHDNNQHLEANLKAQFASQAAARWLGLRLQFIGVIIITGVSFIAVIQHQYDIADPGFIGLAISYALGITGSLSGVVNSFTETEREMVAVERINQYVESIPMESKYFVMDPPFAWPSQGVISFEHVTLHYRKHLPPSLMNLSFDTRPYEKLGVVGRTGAGKSSIISVLFNLVDITAGVITIDAVDIKKISLATLRSRMFCIPQDPFLFSGTLKENLDPLEEFKDDEIWNALGRVNLTDVINSLGGLDYKIDGSGSNFSVGQKQLVCLARAVLHNAKILCIDEATANVDEETDRLIQNTIRTAFRSSTVITIAHRIQTIFDSDRVLVMHQGEVVEFDKPDVLMADSSTYFYRLVQEA

>DvvABC-C_43960*

MEEVKVGGVPEKKLNPKDRSNFISRLFFCYLLPYFVKGYKRELTEDDMYRHRSEHDSSKLGQRLEERWIKHVAKNKNPAFWKVLMGTFFWEIFGQDILVILCEAVRMAQPFIISKLLIVYEKDPKENINEVYLYSGLIVATSFISVVLVHRFCLGLFQIGMKMRIASCSLIYRKALRLSKSALAETTIGQMVNLLSNDVGRFDTAPIHLHHLYIAPIQALIVMVFLYIVVGWTGLLGTFFLLLSIPLQSWLGKKTSQFRLKTATRTDERVRLMNEIISGIQVIKMYTWEYPFAKLVEYVRGNEIKFIRKTSVIRGILISIAMTLNDIAVAICLVTYVLTGNPLTASYAFTVTSYYRLMGSLTYFLPMAVSQGSEMWISMKRIEKFLQYDELNIENLKNSLNGKQNGKQDLQELTGSDKGSIHIKKASAKWLKSQYENNLENIDMDVAAGSVAAVVGSVGSGKTTLLHIIMKELELESGSVNVTGKISYASQEPWLFGGSIRQNILFGETYDQLKYNEVVRVCALERDFTLFPYGDRTLAGERGVTLSGGQRARINLARAVYKDADIYLLDDPLSAVDTHVGKHIFEECICEYLGSKCVVLVTHQLQYLKSIQNIYLLKNGQIAVSGSYNNIKDSNTHYSELLHDIEEEEEEIRRKSKVVQTKEEEAEGEAGPVLQREGKSTGIISGRVYRKYAQAGGHIFKAFSILFLFILSQALLSVCQYFVTFWVNIQQWKSSQNILNSTDTGVVVENVTAPLNLTTPPQSTDNTGFADWWLTPVFVSENTSFYFIGLNMVMVIIMNIRSSSFYAWAITASTKMHNMMFENIVYSPMRFFNINPSGRILNRFSKDIGALDEVLPMTIMDTLGIGLEVVAICFVLGSLTYWIMIPTILIGILFYVMKVVFLETSRDVKRVESVNRSPIYTHLTATLQGLTTIRAFKAEGILKNEFDSYQNNHSAAFFMYLASTRTFGFWLDFICVIYTALVLLSLIFIESETFGGNMGLALTQTLALTGMFQWGMRQWSELENQMTSVERVQEYADLKHEDDNYTVEIDETWPEKGKIEFKNLSLQYSPNDPPVLKNLSF

>DvvABC-C_48940

MVNKIMEKPKEKFNPLSKVNIFSYLTFGYVLELIKKGWKHDLNESDLYGLPKKCKSKFNGERAVKQWNKNPSLFKLLLSKFGLAYLLFCLAHVTWVEIRSIVRPYGMSKLIAYFDKKQQTISETQAFYCASLVIFIKFFSVFYTANINVFELKYLLRLKTSLQSLLYEKLLKLSSNSILETSSGNLITVMTKDIYSIETNLWVFKEFVIFFVQTTTVFYLLYSRIGVTAFVAVGMFAVALPVQGFLCKLVTKLRLTVGKHSDERLQITQEALTAIRVIKMYTWERFFIGKISKERSKELSALLKMFFVHFLIVIIGIFLSGIVFLALILTYTALGYDTNTELIFYIDSLFGELTLAMGIMIPINMSRTAELIASLKRINKVFQCQEISKIEKIYKDALIDINNVSVEIKNQKVLDDVSLKISKPGLHVITGVVGSGKSSIFKVIIGLHNLSEGYCKLGGTISFAAQEPWLFPSSIKNNILFGEKYDKARYEEVLKICGLDYDLAFMQNGDETIVAEAGLNLSKGQQARVNLARCIYKESDIYLLDDPLTALDANVQDVIFEQCILKFLKEKICILISQNPKHIENAENIFTLSHGKLVGDTNIIPAPTPDIGEEQKEPVQVSEAKKQSEAAKSSHEDDQYTNDDLFAEKNNTGKVGADVYKSYMRFGGGICFVVFIILLYTGSQVAHFTSEKLKAKWVDVQANDGSTMLPILGGPYKKQDLLLFTTFTTVFSSLYNLVTLYSLLRFCRNASYNVHKLMVSRITAAVMTFFDTNYLGNVVNRFSYDLNILDERLPMMFLHLFRAAFYCLGILVLIATVNWVFLFPSIIFISLVVTLRILYINTGRNLKRLEAATRSPLVGHINATLDGLSTIRAFDAEKRLTNEFYRHQDLYSSAVFTMRLSHTALIFYMGALSATFSTIIMAKFLFFPDDTSAGNVGLALTNIIRLSDMILWGINEWIEIETNMTSVERCLEYTKVKQEDDSGKTPKDWPTEGGVIYKQVELKYGDNKIPVLKNINFTIKPKHNIGIVGRTGAGKSSIISTLFRLYEYGGNIEIDGVDIKTISLSCLRKNIAIIPQDPILFSGTLRENIDPYKEHADDKIWSLLNQLSIENIQTLDSTVANFSAGQKQLICMARAALNNCRIIVLDEITANMDTENDNLIHSLVEKIFKNCTILTIAHRLNFILSCDMVIVMENGQIVEFDEPKTLLENEKSLFAKICDKASS

>DvvABC-C_217405*

MEESKGQTAEQKLNPIDRSSYFSNLLFCWLLPYFVKGYKRELTEDDMYRHRSEHDSSKLGDRLEERWIKHIAKNKNPAYYKVLIGTFIWEIFVLNVLVVLIEAIRMTQPFIISKLLTIYEKDPKENINDVYLYSGLIIATSLVSVILLHKFNFAMMQVGMKMRIASCSLIYRKALRLSKSALAETTIGQMVNLLSNDVGRFDQAAHHLHYFYIAPIQALIVMVFLYLFAGWTALLGTIFLLLSIPLQSWLGKKTSQFRLKTATRTDERVRLMNEIISGIQVIKMYTWEYPFAKLVELVRGNEVKFIRKTSVIRAILMSLAMTLNDVAVAICLVTFVLTGNPLTASYAFTVTSYYRLMGTLTYFFPQAVSQGSEMYISMKRIQKFLQYDELKTDAYFIPENSLNGNKNGKLAMKELPSSDKGSIHIRNASAKWLKSQPENNLEDIDMDVTPGNLAAVVGSVGSGKTTLLHIIMKELELQRGSVTVSGKISYASQEPWLFGGSVRQNILFGEKYDQVKYNEVVRVCALQRDFTLFPHGDRTLAGERGVTLSGGQRARINLARAVYKEADIYLLDDPLSAVDTHVGKQIFEECICGYLGSKCVVLVTHQLQYLKKIPNIYLLKNGQVAVSGSYDDIRNSNTDYSKVLANIEEEEEEIRRKSRIVQAKQEKDEEEEEVQVLQREAKSTGNISGRVYLKYAQAGGHLFKALCLIILFVMGQALLSMCQYFVTFWVNIQQWKALQNIASPTAPASLENMTAPLDITSPSPQPVDHTGFADWWLTPLFISENTVIYFSALNILMVVIMNIRSSSFYAWSITASTKMHNTMFENIVYSPMRFFTINPSGRILNRFSKDIGALDEVLPMTFMDTVQIGLEVAAICFVIGSLTYWIMIPTICIGILFYVMRVIFLETSRDVKRVESVTRSPIYTHLTASLQGLTTIRAFKAEDILKTEFDKYQNTHSAAFFMYLGANRTFGFWLDFICVIYIALVILSLIFVESETFGGNMGLALTQIMGLTRMFQWGMRQWSELENQMTSVERVQEYADLKHEEDNHTVEPDKTWPDQGKVEFKNMSLQYSPDDPPVLKNLSFVIQPSEKVGIVGRTGAGKSSLIQAVFRLTHIDGSILIDDIDTKSVALKKLRSKISIIPQEPVLFSGTLRNNLDPFDEYKD

>DvvABC-C_10132*

ISMDVSSKSLVAVVGAVGSGKSSLFQVILQELEPSEGYIEVNGKISFASQEPWLFGGTIRQNILFGQPYDKQRYEEVLMVCALQRDLTLLPYGDKTRIGERGITLSGGQRSRINLARAIYKEADIYLLDDPLSAVDTHVGKHLFEECICRFLADKCVVLVTHQLQYLKTVANIILLENGMVRASGSYEDIIKSNSIFTKLLENLEKEEEDEKNQKHVKNKLYIENQGEKEAPALIKEEKGVGKVTTYVYMNYIRAGGNLCKVVILLLAFILSQVLDTSSEYFVTIWVNIEQWVIQTYNSTTVLDKSNQSSTVIANPYDLTQDHWLTSFFTGNITVYVYATFVLLLLIVTYTRSIFFYRWGLSSSIVMHNKMFDNIIYSPMHFFNNNPSGRILNRFSKDIGIVDEVLPTTILDTLQIGLSVLSICLIIGTLSPWMLIPTIIVLVIFYFMRFIFLETSRDVKRVESINRSPVYSHLTASLQGLTTIRAFKAQDILAVEFDNLQNIHSAAFFMYLGATRTFAFWLDFFCVVYIGLVLVALLVVKEEKFGGNVGLALTQSMSLTGMFQYGIRQWSELENQMTSVERVQEYADLTKEIDEGVDPPKTWPEQGQIVFNKLSLGYSKTLPLVLKNLSFIVNPKEKIGIVGRTGAGKSSIIQAMFRLTFTEGSIIIDGIDSKLIPLRKMRSKISIIPQEPVLFSGTVRYNLDPFNEYPDEILWKALEDTELKYVVSDLPEGLDNKMAEGGINFSVGQRQLFCLARAIVRQNKILILDEATANVDLHTDGLIQKTIRKKFADCTVITVAHRLHTIMDSDKVLVMDAGEILEYDSPYILLQNTNGVFYSLLMQTGTNTAQNLIEIANKAYMLSQAANASS

>DvvABC-C_48300*

MDHCQRERRKPNPRETANIFSFLTFAYVGGLFRKAGKADLTEEDIFEVITNCKSKTCGDYLEQEWMNEKKLEKHPSLVRVLWNCYGLKYLLLGFIDFVVSTARSIMTPYFISHLVGYFSPGQTTYTREDGYFYGYSYLIYICLDTVYNHQYLLWVQTLGMEMRTAFASLLYRKALRLSPVAVAETSLGNIVTLITRDVQSFVAVIFTINDLWIAITQTVIICYMLYSKIGVASFVGIGVLLSSIPLQILITTFIAKCRLAGCKKTDERLQLTQEILTTIKIIKMYTWENFFSRKVSQARKVEMKKLTLAFYLKRVLILNGIiFTNLGFFMVILATIWTGVSTDTTVIFFVLSNFRYLRGWLGGAVPFGLGLGSEFVASFKRISKALNAEELNKDQHSEQLIEKPRVELDEISVQLKGEPVLDNVSFTITSGLTMITGAVGSGKSSILKVILKDLPLTKGFVETQGRISYASQDPWLFPSSIRQNILFGEPYNAQRYQEVVKACALLFDFNLFENGDETILTDNGQNLSKGQQARINLARAVYRNSDIYLLDDSLTALDASVQDHIFHECIQKFLKGKICVLVCQTASQIKEADNVIIMENGRIKDMGKPTARIIQQSYMIADQDDSIEKEVIHDSNQPTIEESSEKTGLIEAEQSTARTKIYKEQKKEGSVDWYVYKKFMIYGGGVFLILLNIFMRGFTQFTTTSADRLLTKWVDKKQAVMTIQNSIANISTEQFSYMNITLETAQAEEHTTFRLYYLCLLASSSLELLTTWLLLSFCKTASINIHKALVYQINNSVMRFFDTHLIGNILTRFSQDMMNIDESIAYNLDQFCRMLFSMGGILTLTIFINPAFAVCVIVVTIMLFWVRSCYLPAGRSMKRLEASSRSPMLGHLNATLEGLTTVRASNVQSILIEEYDRHLDLYTSANFTWYMSSRAFGFFVDMLSNFFLIFIIMDYLFFKRDITAGDIGLSLTQISQLSIVLTWGIRVFTELENHMTSLERVIEYTDIKTEKKDGTTVQNWPSTGSISYQNVYLAYGDSDSFVLKNLNFEIKPGEKIGVVGRTGAGKSSILATLFRLYEVKGKIIIDGVDIKTLALDYLRKNIVVIPQDPILFSGTIRSNLDPLNEFEDKDLWHTLERVGINTSIASLEQPINSSVMNFSSGQKQLLCLARAILRKNKLVVMDEATANMDHETDRMLHKIISDNFADCTVLTIAHRLHSVLVCDKVMVLDRGEMKEFDQPQELLKNRNGMFYKMVKQA

>DvvABC-C_47673

MDIAKENYNPNPRDGANVFSVLFFSYTIEMFRKGYRKVLDVDDLYNPIRSDRSTVLGDRLEKKWNSHLLKCNKTRKKPSLLKILVSTFWPEYLYLGCILALMDLLVRLSQPIMLGNMLSYFKPGTETTKEEALWYAGAVVVLNGISALLINQYIMNAFHYGMKVRAAVCSLIYRKALRLSHTALGDTASGKIVNLLSNDVSRFDIVSIFIHHMWVAPTSAIFILFFMYREAGYAAILGILTVFLVVPLQSYTGRLSAIYRKQTALRTDERVRLMDEVISGVQVIKMYAWEKPFKHLIKLARKAELKIVTKSSYVRALFMTFNLFTTRAALFATLLTMVLTDQQITATKVFVFMSYFNILSQTMSAMFVRGIAEIAELIVAIKRVQEFMLNEEFRPVVKYQNNNDKIFFNKTLINLKDLTVKWNLATKDDALKKINLAVNEGKLIGIIGTVGSGKSSLLQTILGELEVIYGSIEVNGTVSYASQEAWVFAATIRQNIVFGGKYDKHRYNQVVKACALEKDFKQFENGDLTIVGERGASLSGGQKARINLARALYREADIYLLDDPLSAVDIHVSKHLYDECINGYLAHKTRVLVTHQVHHLKDADHIVILNNGTIENEGSFEELSVSDNLYAKLLTSEVELTDEEKQKQTDTAKISRKISIRRSRTSLVSAASELSLTDAIIKEATGSDDEEEPEIRLKDLQEESSKGKVKESLFWSYLIHGGNIFFVAFVLLLYILSQVAASGVDYFVSMWVNIEEFRNQTLSRTSANKTEEVSIRSTVEWSTETCIYIYAGGLIALFVIAFARSMLFYKLAMWSSQKLHDVMFHSVIAATMRFFDTNPSGRILNRFSKDIGAIDEWLPKAILDASQIILMMVGSLILVAIINPYFLILVFFMGIFFLSMRHVYLKTSKNIKRLEGIMRSPVFTHLNATLQGLTTIRSFGAQPILMNEFDKHQDSHTSAWFMYIAASSAFGFYLDIICFIFIAIITFYLLNFGEALSLKGGEVGLAITQATALTGMVQWGIRQSAEITNQLMSVERVLEYKSLPAEIQPVVPKKPLPKWPEKGNVSFRNMGLKYIENGPLTLNNLNLSIQSNEKVGIVGRTGAGKSSLIAALFRLAKIDGEINIDGIDTKDLLLEDLRSKISIIPQDPVLFSGNLRYNLDPFEEYPDELLYKAIEDVELKDPANIINRLENKVMDRGSNYSVGQRQLICLARAIIRNNKILVLDEATANVDPQTDALIQKTIRKKFADCTVLTVAHRLNTIMDSDKVLVMASGTMVEFDHPHLLLQNSNGVFYKMVAEAGKSLGDQLRKIAKDSYQRTLSLPE

>DvvABC-C_5345

MNKNKLSDNRQEHPIQNSNFLSRIIFLYTLPIFWKTHKRGTFEEDELYEVLKKCKAEEAGNEVEKCIKDDVKNHGYLSLFRILCKCYGLKYFLLGMIQVIVRSIVIITIPLTLSKVIKYFQPGQDELSRSSAIYCAILLVLLNFLSVTYLHNYLLVLSELGIKVRTAFCSAIFRKSLRLTQKSLLEVSSGKIVTLMTKDVAAIENFIFYFNDVWVGIVQAIIVFYLIFNKMGVGSLIGIGFFLLVIPAQVLLGKLISKLRLESSKKTDERLQTTKEMLSGISTIKMNVWEFEFEHKINQTRKKEMKAILKIFLAKTTVLLIGSLTSKIAYYLLLTTFILLGNTISAELVYYITTLFLRIRHALNVAIPIGVTVTADMSAAMKRIQQMMTAEEVIVNDEIEVDQKKSYVSLNNLSIEVDDKVLLKDITLRFDSGLYIISGSTGSGKTVLLKSILGEFDTKSDHIIKYGTISYAAQEPWIFPATFKQNIVFDEPFDEERYNRILDICALGFDIEHFTLANNLKIGDKGANLSKGQKSRINLARALYRNADIYLIDDCLSSLDVNVQRHVFQKCIREFLSDKICIFVTQNLQYTNGNDKIIVLNEGKTFVSQNLSEEDDQDIKTILKKEQQLKNLIEVNNNADEVNNDETTEESSLLKKLNIGRNNMYQENKKSGKVDFYTYRKYGNYGGGILVIAIVLVFFIGSQFSKSYTEKILSNWVNHIHHNTTNINEENTVWDFSLKIYSAFTLSTTILSIFSAFLLFNFTRKASIKLHRCLSNTVINATMKFFDSHLIGNIINRFSKDLSIVDEQIPFAIYEFLEIVLSLCGVIILITLVNVGFIIPTAVVFILLYFLRKLYLPTGRNLQRLDTAVRSPMLGHLNSSLEGLSTIRAFKAEETISKEFDRHQDLYTSANYTSKCCQRAFGYYLDVCCTTFIGAIIAAFIIVGKETGVGDVGLAITQAFMLNGIAQYAIRLWADIENKMTSVERILEYVGIKTEDNDLRDIFKDWTSNNIIQYKNVNFRYDNSQQYILKNINITINEGEKVGIIGRTGSGKSSLITLLLRLFDYEGKITIGNEDIQSLPLTLLRSKISIISQDPVLFSGTIKSNMAPKVFGVYPDEDIWKVIHKLELTTIIDSLDFQICEGGKNLSAGQRQLICLARCLLHQNKIILLDEATSEMDQGSESLFNNILEKQILDCYSFRSTVIVVAHSLKTILNCDRVLVVQNGEIVENGAVSTLAQDEHSLFYNMLKSSGLLTIKC

>DvvABC-C_22413

MDSTKETYNPNPRDSSNIFSILFFGFTYPIFKKGMKKDFDVEDLYNPLKNDRSTLLGDKLERNWDKQKLKPKGKPSLLRAVAITYWYEYLRLGFLTLIGDVFIRVSQPYVLGLLLTYYNPHSKTTKEQALGYAGMIVTFNTLTSFIKNQYMMNSMHAGMRVRTSVCALIYRKAIKLSTTALGKSSVGKIVNLLSNDVSRFDSASMLLHQMWVGPVSAIIIMYIIFQDIGWSGATGVVTIVTIMPMQAYIGKLSAKYRRIIAGKTDERIRLMNEVIGGIQVIKMYAWEIPFTKLISLARKAEINVIRRSAYIRGVFMAFNLFNNRFALFATLATMVFTNKAITAARVYVFMSYYQILANTLAGVFVNGLTQIAELLVSINRIEEFLENEEHKELPALKRSVISNVNEEMVACKNMVASWNENSLDPVLKNLNFKLGKNTLLGVIGPVgSGKSSLLQSILGELDIVDGSLAVHGTLSYASQEPWIFSGSIRQNILFGSDFDKARYDEVLKVCELRKDFDQFPDRDFTLIGEKGANLSGGQKARINLARAIYKDADIYLLDDPLSAVDSIVSKVLYEDCINGFLAKKARILVTHQVYYLKTADHILVLNNGGIEIEGTYSELLKSDNPLTVHLTEEIEEILKRQKSQDSAIEEQEAKTMSTMDLSKVNGKMKAIVKEMQEETSKGKVKGSLFLEFFKSGMSICEIIFLVTLLTIAQASATVIDWFISFWTNIEEYQMTLNSTAANVLNSTVVDALNSTAVDAINSTAVDVLNSTTVNALNSTVVDESVPFYMNWSSQTCLIIYGAILVFCLVTTLSRSLSFYRFILNCSENLHGMLFTGVTNTYMRFFDKNPSGRILNRFSKDIGSVDEILPRMLFEASRVTLKMLGHLVLVLYVNPASVIVVIILGILFSFIRIVYLRSSNNIKRLEGRMKSPVFSHLTATLEGLTTIRAFKAQNILRAEFDKHQDSHTSAWFMFISTSSAFGFSLDVICLMFIGSLTFSLIGLGEYFNLTGGDVGLAINQASSLTQNIQFLVRFSADISNQLMSVERILEYKELIPEQQPEKLLLPPKSWPDKGIVNIEHLNMKYIDDGPTILKDVSVKINPKEKIGIVGRTGAGKSSLISAIFRLTPLEGKIYIDDINTKDITLKQLRSKVSIIPQDPILFSGTLRYNLDPFDEYSDETLYRALNEVELKDPSNIINRLENRVMDRGSNYSVGQRQLICLARAIIRNNKILVLDEATANVDPQTDALIQRTIREKFADCTVLTVAHRLNTIIDNDRILVLEAGEIVEFDHPYLLLQNKFGVFRKMVEETGTAMLRQFLETSSQNYQKLID

>DvvABC-C_18709*

MEMTKEKYNPNPREKANILSTLFFGYTYDVFKKGLSKTLEVDDLYNPLKIDRSKILGDHLQSNWDKQLEKAKKTNSKPSLLKAILMTYWLEYLKLGIFDITTDLVLRLIQPLMLGKLLDHFKPEAQVSKNEALMYAGIIVAINVLNFLIGNQYMVEGFHSGMRIRAACCAVIYRKSLKLSKTALGETASGKLVNLLSNDVSRFDLVSLLIHQIWIAPVLSLIVMVLLYQRTGYAGIVGVVAVFVIVPIQTYTGKLSAKYRKQTAMKTDERVRLMDEIISGIQVIKMYAWEIPFRKVIRIARRNEIKIITKSAYVRASFMALNLFTTRLALFCTLLTVILSNEPITASMVFVVMSYFNVISMAMSTMFTRGVSEIAECLVAVRRIRDFLLNEEYDPNRSTSGMNGNVKSVDDFKEIISLKNLTVKWNLSFSDNALENINLNVQDGQLIGIIGPVGSGKSSLLQTLLGELDITQGEMRVQGQISYASQEPWVFAATVRQNILFGEEYDKKRYQEVIQACSLEKDFEQFPNGDLTLVGDRGSSLSGGQKARINLARAVYREADVYLLDDPLSAVDIHVSKHLYEKCINGYLASRTRILVTHQVHYLKDADNIIILNNGRIEDEGTFNFLANSDNVYAKLLTAEPENKEEKKPERQKYSRQLSQRSRKDSMSSIISELSIADTLLSNDVDFDEEEAEKEPEFDVKDLQEQSSKGKVGGSLLFKYMLAGSNVFAVFICVLLYLGTQLAASGTDYWVSYWVNVEEFRNSSESLNSTAPRFIIPSIELTTDNCLYIYSVILGALFVLAMTRSFFFYKMAMLSSKKLHGTIFDNVIDATMRFFDTNPGGRILNRFSKDMGAVDELLPKAILDSSQILLSMCGSLVLIIVVNPYFLILIGVLSAVFGVMRHIYLKSSKNIKRLEGIMRSPVFTHLRATIEGLTTIRAFGAQSTLMDEFDHHQDYHSSAWYMFIVSSTAFGFYLDCFCTIFLAVLTFCLLLFGETFNLRGGEVGLAITQATALANFLQWGMRQSAEVTNQLMSVERILEYKSLDKEPQPVGPKKPAKEWPQKGEITFKDTCLRYFEGGPLVLKHLNLSIQPKEKVGVVGRTGAGKSSLIQALFRLAPIEGTIKIDEIDTKDITLNDLRLKISIIPQDPVLFSGTLRYNLDPFEEYTDEVLYKAIEDVELRDPANVINRLENRVMDRGSNYSVGQRQLICLARAILKNNKVLMLDEATANVDPQTDALIQKTIRKKFSDCTVITVAHRLN

>DvvABC-C_21941

MDSTKKHNNPSPQISANFLSKIFFCWIFPLFKTGFKKKLQPSDIYNTLNSDTCHATINTLQRHWENELMLHKAGKKDKPSLKLALFKTYAFPYSLQGVMVFLQVVLIKTLQPLVLAELLKYFDKTQKYDMFGEYSGWILGTATVFLAFIFALSYHHSTLGSQRIGMRARAACSALVYRKLLKLSQASLNKTPGGKLVNLLSNDLQRFDIASMYLNFIWVMPFQAAICFYIMYRSVGIAALAGTAFMLFEgIVMQGYLSRLQGTLRSKIAEKTDFRVKLMNELVAGIKVIKMYAWENYFAKVVETARTEEVRLISKTSAIKGLSLAFIVVTERIALYLTVITFVLLGHQISADKVFLSAQLYNSLQLYSCIMFPYALAGYAEVKVSLRRLEQFLLLEENNVQVQNTVVQQIGTIQAREACATWNEDLKDDTLNNLNLKLrAGKLCCVVGTVGSGKSSLLQMLLGELPIKSGQLEVSGDLSYASQEPWLFVSSVRENILFGKPYIKNRYDEVVQVCALETDFQQFPFGDKTIVEERGVSLSGGQRARLNLARAVYTSADIYLLDDPLSAVDTRVGKHLFEKCIKGFLGSKTRILVTHQLQFLKDADVIIVFEKGKIKKMGTFDELSEDYLKSLQENRSEEQIEDEKKDVSLNTRKQSNMEEIKQVLSQNEEGPKENNEETCEGSIPFSTYGKYFRFGTSCFGFCFMVILFVLAQAASNAGDLWVTYWVNTVAANSTTTPPLEIVVANNTSSGNYSKYNYSVVGENIPLEERFTHDSNYYIIIYSVTICLAVFLTPIRSLNFYRIIMNASRNLHNSMFSKILEAPMRFFDTNPSGRILNRFsNDMGIIDELLPKAMLDGTQVLLVLVGILVLVFIKILWMIIPAVVIGILFYYLRLFFLKTTQDVKRLEGVSRAPVYSHVIATLDGMTTIRASKCEDMVIQEFDGLMDNSSGAWYLYIASCEVFGFYLDCISTVFIAIVTYQFLIFDNPDPSSAGNAGLVLSQCLILTGMLQMGVRQTAEVANNMTSVERVLEYTTLEKETEGKKQLSVNNLIPRYKSLDKDWPQAGEVEFKDVFLRYTLESAPVLKNLNLVFRAGEKIGVVGRTGAGKSSLVSALFRLSPLEGSVKVDGVDTVNIELSKLRSTISIIPQEPVLFSATVRYNLDPFNTVSDEQIWDALEKVELKHSITDLEMEIREGGSNFSTGQRQLMCLARAIVRNNKILVLDEATANVDPSTDALIQKTIRKNFKYCTVITIAHRLNTIMDSDKVLVMDAGQAVEFEHPHILLKDYEGYFSRMLKETGTATEKALKKIAEEHYRQLFLDEQEASQEGL

>DvvABC-C_15305

MEMTKEKYNPNPRETANIISALFFGYTYDVFKKGLSKTLEVDDLYNPLKSDRSKVLGDNLQRNWDKQLEKSKKSNSKPSLLKAIVMSFWLEYAKLGIYTITTDLVLRLIQPLILGNLLDYFKPESQVSENEALTYAGILVAINILNILMSNQYMLEVFHSGMRIRASCCAVIYRKSLKLSKTALGETASGKLVNLLSNDVSRFDIVSLFIHQLWMAPVLSLIVMGILYQRTGYAGVVGVVAVFVIVPFQTYTGKLSAIYRKQTAFKTDERVRLMDEIISGIQVIKMYAWEIPFRKIIRIARRNEIKIITKSAYVRATFMALNLFTTRLALFCTLLTVILSNEEITASKVFVVMGYFNVLSMAMSTMFSRGVSEMAEVMVAIRRIRDFLINEEYDPNRTNFAMNGNVNSVDDFKEIISLQNLTVKWNASFSDNALENININISDGQMVGIIGPVGSGKSSLLQTLLGELDITEGSMRVRGQISYASQEPWVFASTVRQNILFGEEYDKKRYHEVIEACSLEKDFEQFPNGDLTLVGDRGSSLSGGQKARINLARAVYREADVYLLDDPLSAVDIHVSKHLYEKCINGYLANRTRILVTHQVHYLKDADNIIILNNGRIEDEGTFNFLANSDNVYAKLLTAEPENVEEKKQDRSKYNRQLSIRSRKDSLASIVSELSIADTLISNDIDFEEEEAEKQLEFDVKDLQEQSSKGKVGGSLLFKYMLAGSNSFAVLICVILYLGTQLAASGVDYWVSYWVNVEEFRSSSESSNSTTPTFIIPSIELTTNNCLYIYGFLLGALFVLAMTRSFFFYKLAMWSSKKLHATIFDNIVTATMRFFDTNPGGRILNRFSKDMGCVDELLPKAILDSSQMLLSMCGSLLLIVVVNPYFLILIGALSVVFGFMRHVYLKSSKNIKRLEGIMRSPVFTHLRATIEGLTTIRAFGAQSTLMDEFDHHQDYHSGAWYMFIVSSNAFGFYLDCFCSLFLAALTFSLLLFGEAFNLKGGQVGLAITQATALTSLLQWGMRQSAEVTNQLMSVERVLEYQNLEKEPQPVVPQKPAKEWPQKGEITFKDTCLKYFEGGPLVLKHLDLKIQPKEKVGVVGRTGAGKSSLIQALFRLAPIEGSIKIDDIDTKDISLNDLRLKISIIPQDPVLFSGTLRYNLDPFEEYSDEVLYKAIQDVELKDPANVINRLENRVMDRGSNYSVGQRQLICLARAILKNNKVLMLDEATANVDPQTDALIQKTIRRKFSDCTVITVAHRLNTIMDSDKVLVMDAGQISEFDHPHLLLQNKNGVFYSMVAETGRTTAEQLRKIASDSYQKLNALPE

>DvvABC-C_12562

MEEIHSKAKAKKKNPLDTANIFSSIFFFWLLPFFVKGYKKDLTEDDMYEHRNCHESGKLGDQLQVKWNKQLRKKKPSLIKACASSFVLDLLGINCLVVIAEVVRISQPFLIAELLKVYESKQLDEERSEIYLYASLIVLTSFVAANMGHNFNLRLMQLGMQMRVASCSLIYRKALKLSKSALAETTIGQMVNLLSNDVSRFDLAVYFFHNIYIGPIEILIVMYLLYVNVGGAALAGAIVLLLFIPFQSWLGKKTSQFRLQTANRTDERVRLMNEIIAGIQVIKMYTWEQPFAKLVEFSRKKEMKYIRYTSVIRSVLMSCIIMLHRSAIAASILVYVFTGNALTASYAYTVTSYYRLLYTVTNFLPTAISQAAELYVSSRRIQTFLLYDEVEDENYIALDEKSHKENGIKSLELTIRNKEPGIHLKNASAKWLKSSPENNLEKINMDVTPGNVVAVVGPVGSGKTTLLHIILKELELQSGSVDVHGVVSYASQEPWLFGGSIRQNIIFGQKFDQSKYDEVVKVCALQRDFTLFPHGDRTLAGERGVSLSGGQRARINLARAVYKDADIYLLDDPLSAVDAHVGKQLFEDCVTGYLSSKCVVIVTHQLQYLKKLKHIYLMRDGKVKLSGTYQDLKNSNTEYSKLLTDIKDEEETRKMSRTRTLSREEEMFEDNEIQILAKEAQSTGKISGRVYMNYIKASGHLFHIFLISMIFITSQFMESFSEYYVTYWVNIQQVNSQNLTETVTSNSNITTNLNITSNSSLPKALRTTVQPENPYEGWWLTPLFTSEYTAIYYLLVVISLIIMILGRSLYFYHLSLTSSTTLHNQMFQNIIYSPMRFFNINPSGRILNRFSKDIGVMDENLPMTLMDTVQIGLYVFSICLVIGSLTIWIVIPTVLLAVLFYLMRIVFVQTSRDVKRIEAVTRSPIFTHLSASLQGLTTIRAFKAQEILKKEFDNYQNLNTSAYFIFAGANRTFGFWLDFVCVVYIGLVLAALLLIKSEQFGGNIGLALSQAMALMGMFQWGMRQWSEFENQMTSVERVQEYADLKQEIDQPSNKPRETWPEKGEVEFSNMSLKYSPDDPFVLKNLTFVVKPKEKVGIVGRTGAGKSSLIQALFRLTHIEGSILIDGVDTKTISLKKLRSNISIIPQEPVLFSGTLRKNLDPFDEYKDEVLWSALEEVELKHAVQELPAGLDNKMAEGGSNFSVGQRQLLCLARAIIRNNKILVLDEATANVDPHTDGLIQMTIRKKFENCTVLTIAHRLHTIMDSDKVLVMDAGKMVEFDHPYSLLQNKQGTFYSLVMKTGKGTAQTLMSIAEESQKTK

>DvvABC-C_10642

MDTGNKTTKPRNPAENTNPISFLFFFYMFPIFKKTYKYKLTEEELFSPLKEHTSSRLGSKLEKAWKEEYRIHKKTALHRALFRIFGLRYTVLGLIRLFDELLLIVVMPYCIRTLVAYLEAGQTKITKDEALIYAAALVITLLLDAVMQQPNYMGLQHIAMKIRVACSSLIYRKTLRFSREALGNTTVGQLVNLLSNDVSKFDQLFGLTHYAWIGPIQVALGTWLLYREIGVSAFFGMAFLVAFVPLQIWLAKKMSVMRLKTALRTDERVKLMNEIISGIQVIKMYCWEKPFAHVIDLARRSEMRAIRSHSCLLGILYSFEVFVSRTAIFVSIVGYVLLGNYVSADKVFAITAIYNQMRTIITIIFSLSITALAEMHVTIDRIHKLMIFDEREQDSEDGYEKMNGNYQSKLNGLDGMKNGINGNGTAIELIKKVKEPKLMLSGVSAKWLAQSPENNLSDITFNVPPNKMLAIIGPVGSGKSSIINLILKELPVKSGKLEIDGKVSYASQEPWLFAASVRQNILFGEEYDEERYKLVVEVCALKSDFALFPHGDKTLVGEKGKALSGGQKARVNLARCVYKKADIYLLDDPLSAVDANVGKHLYDRCIKQFLSNKICVLVTHQLQYLRNADKIIIMKDGKMEMTGSYTELKKSGLDFAKVMEEFNEEAEEDKRMKSIKSKASIYDEPMEDEEDQVLEKEMQEKGTIKAATYYLYLKAGGGICSMLALGFLFIICQVVANAGEYYVTYWVNLEQDFSEKQRMNLTAPNETINRDLIMWSYTALIVGNIIISVVKAVYFMIFFVIASKNLHKYIFDKLIKATMRFYNTNPSGRILNRFSKDLGTVDEYLPSVIIDVIEIALLLLGAITLSAIVEPLLTVPAVVLMIIFYLLKIVYSETSRSVKRVEAITKSPMLSHLTASVNGLSTVRAFHAEKMLTEEFDNYQDSHSAAWFLYLASSKCFGLWLDIICIVFIAVAVFSLLLFRDTIHGGDLGLVITQYLGLMGSLQWGMRQWTELENNMTSVERILEYTRLETEPERKEPKNIPQPWPEKGLVEFRDVSLRYSPQDPPVLKGLNFTVQPKEKIGIVGRTGAGKSSTITALFQLYPLEGTVVVDGVDTTQIPLDLVRTNISIIPQEPVLFSGKMRENLDPFENYSDDVLWNALDQVELKDVISELPAGLNTEVTEGGNNFSVGQRQLVCLARALIRNNKILVMDEATANVDPHTDSLIQKTIRDKFADCTVLTIAHRLHTVMDSDKILVMNSGRVEEFNHPYLLLQNVHGVLHNLVDATGPSTAKNLENIAKESYEKKRV

>DvvABC-C_41602

MEEIRNDQPPKKSNPIERTNAISRLFFCWLIPYFAKGYKRDLTEDDMYKHRSEHDSGSLGARLERRWSKHIKNNKNPAYWKVLIGTFFCEIFLLNILAFTTEGIRMAQPFLISKLLKVYEEIPTEESMNDIYLYAGLIIVTSFLNVIMVHRFNLAVMQIGMKMRIASCSLIYRKSLKLSKSALAETTIGQMVNLLSNDVGRFDQATHHLHHFYLAPIQTTVVMVLLYLVVGWTALLGTVFLLLSIPLQSWLGKKTSQFRLKTATRTDERVRLMNEIISGIQVIKMYTWEYSFAKLVEYVRKMEMKYIRKTSLIRGLLMSMTTVLNKGAVAISIITYVISGNTLTASYAYTVASYYRLLGSITMWFPQAVSQGSEMFISMKRIQSFLLFDELEADKYFEPGQELKEVKKQHKYITDKVLQTSNGSIHIKKASAKWLKSQPDNNLENINMDVTAGDVVAVVGSVGSGKSTLLHIIMKELELQSGSVDITGTVSYASQEPWLFGGSIRQNILFGEEFDQEKYDEVVKVCALERDFTLFPHGDRTLAGERGVTLSGGQRARINLARAVYKDADIYLLDDPLSAVDTHVGKHLFEECICGYLGNKCVVLVTHQLQHLKNIDKIYLMTNGKIAVSGSYSDIRESNTEYSKLLADIEEEEEETRRKSQRVATKSEKPEKEGAVQVLQRESTGSGNISDHVYMSYARAGGNLIKVFLLIFMFFFGQALDSLSDYFVTFWVNIQQWNSTKVIINATDPDAVVYHSFENWWLTPVFTSKNTVYFYTALVASVVIIVNIRSLSFYLWCIGASTKLHNKMFMNIVYSPMRFFNTNPSGRILNRFSKDIGSVDEVLPTTLLDTVQIALAVAAITTVIGSLTYWIMIPTVAIAVFFYFLRVIFLETSRDVKRVESVTRSPIYTHLTASLQGLTTIRAFRAQDILKEEFDRYQNLHSAAFFMYLAANRSFGFWLDFICVIYVGLVIVALVFVKSEQYGGNMGLALTQTLGLTGMFQWGMRQWSELENQMTSVERVQEYADLKQEDDEHSIEPPKTWPHAGEIQFDNMSLYYTTDDPPVLKNLSFTIKPSEKIGIVGRTGAGKSSLIQALFRLTHIEGRILVDDFDTKKISLKVLRSKISIIPQEPVLFSGTLRNNLDPFDEYTDEALWNALEEVELKQAVEELPAGLANKMAEGGTNFSVGQRQLVCLARAIVGNNKILVLDEATANVDPYTDALIQKTIRKKFANCTVLTIAHRLNTIMDSDKVLVMDAGQAVEFDHPFTLLQNINGVFYSLVMQTGKATAKNLFAIAEQSKMLRDNNTVF

>DvvABC-C_12703

MDDGNCDELKNKGKNPQQSSNFLSNLFFCWGLKVFYKGWNKVLNEDDLYKPLEEHESHYLGDQLEAFWKLEKLEHSHPCLIRPIWKLFKKDILIHACCTFVLEFCIKLSQPLLLKKLIEYYEPNQTSVTLTEAYIYSLFIVLCSLFYVIFCHSFHLSLQHLGMKIRIACCSLIYRKSLKLSKKALVNTTIGQMINLLSNDVNRFDNLFRYWHFLWVGPLESIVVMYLIFEVAGYAGLAGFlIMLLFIPFQMVMGKLTTQFRQKTAGKTDERVLLMSEVICGIQVIKMYTWEKSFSKLIEHIRNSEIKQIRLTSYIRALHMSFSKFITRAAVFLCIITYTFTGKRLNAGYVYVISPFYNILKSAVSNDFPQAIIQTAEALVSIARIEEFLQFDEVYYEPIREPYQAKKITSGTSLIHSSKKKKSVGVYLENVSAKWIDTQEENTLMNINFNVGPQQLVAIVGAVGSGKTSLLQTIMKELPLSQGNKDTVGKISYASQEPWLFASTIKQNILFGEPWDPKKYERVIKVCALERDLSLLAHGDKSMVGDRGVALSGGQRARVNLARAVYKDADIYILDDPLSAVDTHVGKQIFEDCICSYLRGKCTVLVTHQLQYLKNVHKIYLLERGKIVVSGTFKEISESNTDFARLLVSNVEEEESDTIDEESSEIEYFENMSDEKPTEITEETVVVKLTSRAYSGYLKYAGGWFYGFWVLMLFVLTQFLASGSDYFVNFWVNREQETFQEEEGNKTLLLGYSDDVYMEENYNYVWKYSLGHLTPIQPDFFNSDRCIIIYSCIILSVITVTVMRSLCFYKMCMNASVKLHNTMLHKICNGTMFFFNTNPSGRILNRFSKDMGCIDETMPNVLVDTIQIALNVLAVNIVIGTVNPWILISTLAIGCLFYLFKVVYVSSSRNLKRMEAATRSPVFSHIHASLQGLTTVRAFGAQEILRNEFDKHQDLHSSAYYLFVCCNRAFGFWLDLICVGYIALVTFSFFIFGSAYGGNVGLAVTQAISLTGMFQWGMRQWSELDNSMTSVERVLDYINIKQESLEKQKEPPKKWPEYGMLEFQSVYLRYATNLPYVLNNLTFKIKPKEKVGIVGRTGAGKSTLISALFRLTDIEGKILIDGICTSDIPLISMRSKIAIIPQSPVLFSGTVRSNLDPMDEYDDKDLWRALEEVALKDVVSEMEMGLESKIGEAGSNLSLGQRQLVCLARAIVRKNKILVLDEATANVDPKTDALIQSTIRNKFANCTVLTIAHRLHTVMDSDKVLVMDAGSAVEFNHPHILLQNTEGIFCSLVKQTGKSMAENLCKIAEESYMNLQK

>DvvABC-C_14968

MEEVKKNRIQKKPHPLKRTNLFSHIFFCWLPGFLSKGLRKDLDENDMYQTRHSQQSRYLADQLVVAWKNELKKSKPSFLWALFVVFRLELFYYAFFNMFSDFIKIAQPMLISRLVSYFQPGATTNQTEIYINAFLIIVASLIQVTSVHNYQMLVMSLGMKVRVASCALIYRKALKLSKTSLAETTIGQMVNLLSNDVGRFDFSGQHIHNVWLAPCETVVVMILLYFYVGPTGLIGCVFLLSFIPFQMYMAKLTSQYRLKTAIRTDERVRLMNEIINGIQVIKMYTWEKPFAKLVEMVRKREITEIGHTSTIRAIMMSFNLTMSRSAVFLCVMTYVLTGNTLTASYAFTVTSFYAFLRVSVTQQFPQAITQFAETKVSISRIQKFLMYDELDRRTDSEKNGLNGVTEKISNKTDAKEVGVKIKNASVKWIKSLPENTLEEITFEAKSNQLIAVVGTVGGGKSTLLHVILKELDPIEGSVEVNGSISYASQEPWIFGGSIRQNIIFGQKYDEIKYEEVLRVCALEKDLALFPHADRTLVGERGVMLSGGQRARINLARAIYKESDIYLLDDPLSAVDTHVGKQLYTNCITGYLKHKCVILVTHQLQYLRTANCIYLFEDGKIRASGTYQALKNSDSAFTKLLASSKDEEKKDGARRISKAESVESEASEAEHEAIEQKREERATGTVSKRVYANYINAGGYWLKSVVLLCTFVAAQVFGSLTDIFLTTWVNVEQWRVDNKNNGAFTNKTLNESLANATYSTNDNKPPFWSTVLTEDNTLLIYSCLVILTIILAVTRSITFFRFCLRASTNLHNSMFGKIIFSPMLFFNTNPSGRILNRFSKDIGALDESLPNCLVDTIGIGLIVTGTTLVIASVNPWVLLPTGVILVIFYFIRQAFLASSRDIKRVEAVTRSPIFTHLSASLQGLTTIRAFRAEEILTKEFDHFQDAYTAAYYMFLTANRGFGFWLDLHCVIFIGMVVVSILFIQKETFGGNVGLSLTQAITLSGMFQWGMRQWSELENQMTSVERVQEYADLPKEKDEIKKEPPPNWPSMGHMKFENMSLRYSDDSPYVLKNLNFEVKPKQKIGIVGRTGAGKSSLIQAVFRLAQNEGHIFIDGIDSKSVELRLLRSKISIIPQEPVLFSGTLRKNLDPFDEHKDEVLWDALEEVELKHAVDELPAGLDSKMAEGGSNFSVGQRQLVCLARAIIRRNKILVLDEATANVDPMTDSIIQTTIRLKFSECTVLTIAHRLHTIMDSDKVLVMDAGEAVEFDHPHKLLKKKGVFYGLVRQTGTTMAENLQGIAEESFYKQIKDR

>DvvABC-D_11014

MPTVISKFLEQGESTFKQNKSVFSGAFIAAVLCTYAYKVGYPFVDSLIHKPKDNLNNNHIVQKDLQRKNGLVKNKKLKGRLKNSIPNFNLAFILQFIKLVRIMIPSFICTETVLLSGHTTFLFLRTFLSIYVANLEGAIVKYIVMKDPQNFVKQLGKWFAVAIPATFINSMIRYLESRIALSFRTRLVDHSYKLYFKNQSYYRVTVLDGRLDNCAQRLTDDIETVANTVSHLYGQITKPCFDILLMVIALANLVKSRHSNLVIGPVIICGVVMFSALLLRFVSPRFGHLVAQEAEKKGYLRHVHGRIVSNAEEIAFYGGHQVEESQLRQAFRVLSKHLEHMFGVKLWFIMLEQFLMKYVWSGAGIIVVSLPILLAAGNRRKISNKSLLSIPDFSSKSESLIAEDTADDQIEDSVSERTHYFTTSKNLLITGSDAVERLMSSYKNIVELAGHTARVANMFEVLEEASNGIYHKTLVAKKEKSADFEIEFRGDQPLAKGKIIYSTNNEIILKNVPIVTPNCDIVCPSLSLELTPGQHLLITGPNGCGKSSLFRILSGLWPIYGGELHTPKNSMFYIPQRPYMVIGNLRDQVIYPDTYTDMVKKGITEENLLKIMTMVHLDHIVERDGFYEMKDWTDILSGGEKQRMAIARLFYHKPKYALLDECTSAVSIDVESFIYQSAIDMGITLLTITHRPTLWKFHTHILQFDGTGSWEFSQLNHTSRLTLKKEKEDLLKAENNEERSKRLDELNKLLGEDS

>DvvABC-D_11628

MAPNYSKVLNKHKALAGAGALGTILLIILKYRNKQGKLNKKKVQKAVEEEVKYLISEKGETKIKAQVDKKFFSQLSQLFGIACPGWTSQESGLFFLIALSLVSRSMCDLWLINHGTKIESSIIAMDPTLFKQRLLYYVLAIPIISVVNNVLKYSIGALKIQLRTNMTRHLYEEYLKNYTYYRISNLDNRISNADQLLTTDIDKFCEGVTDLYCNTAKPLLDICIYVYKLSTTLGGGTPGIMLVYLLVSGVLLTNLRKPTARLTAGEQKLEGEFRHINSRLITHSEEVAFYNGNSREKATLMASYNKLLNHLRKFLRFRVAMGVVDNIVAKYFAGVVGFWVVSLPFMTSGHSFRLLGTNERSRLYYTYGRMLVKLAEAIGRLVLAGRDLTRLAGFTARVTQLKTVLSELNSGKYQRTMVSGSESLQINGGkLIFRNNIIKFHKVPLITPNGDVLINEITFEINSGMNVLVCGPNGAGKSSLFRILGELWPLFGGELTKPPRGKLFYIPQRPYMTLGCLRDQLTYPHSGAEAARRGTTDAKLEEYLQRVQLGYILEREGGLDAVADWLDVLSGGEKQRIAMARLFYHQPQFAILDECTSAVSVDVEGSMYKYCRDVGITLLTVSHRKSLWQHHEYVLHLDGRGGYSFKPIDNCDEQFGS

>DvvABC-E_2830

MSKRKGMEESDKLTRIAIVNADKCKPKRCRQECKKSCPVVRLGKLCIEVVPNSKIATISEELCIGCGICVKKCPFEAIAIINLPSNLQKETTHRYGKNSFKLHRLPIPRPGEVLGLVGTNGIGKSTALKILAGKQKPNLGRYMDPPDWTEILSHFRGSELQNYFTKILEDDLKALIKPQYVDQIPKAVKGTVGQLLDRKNELDNMNQICGMLDLLHIKEREIAALSGGELQRFACAMVCIQNGDIFMFDEPSSYLDVKQRLNAARTIRSLIDPSKFIIVVEHDLSVLDYLSDFICCLYGVPGAYGVVTMPFSVREGINIFLDGFVPTENLRFRDESLVFKVAESATEEEIKRMNHYEYPTMTKTMGSFELKVAQGQFSDSEILVLLGENGTGKTTFIRMLAGNLEPDSGSGELPQLHISYKPQKISPKSTGLVRQLLHEKIRDAYIHPQFIADVMKPLKIEDIIDQEVQNLSGGELQRVAMTLCLGKPADVYLVDEPSAYLDSEQRLVAAKVIKRFILHAKKTGFVVEHDFIMATYLADRVIVFEGSPSVKTTAHAPQTLLAGMNRFLELLGITFRRDPNNFRPRINKLESVKDVEQKRAGQYFFLED

>DvvABC-F_2701

MSKKRGAKKGKNLDDDFEETSSIISEKEKINSKTPKNKTTKKGKKGKDDWSDDEEIEQGKEVNQSEPAESKPVAKKKGKKGKGNKNDDWSDKEEADIKLSDSETESMPVAVKKSAKKNKKKKDDWSDKEVDIQLSESDIEEAPKIVKKSAKKNKKKDDWSDKEDIEIETKLSDSEEELAPAIVKKSAKKNKKNKNQVRDESPDIEEMEAHDEGFKEDDVSEEEIVKPVKQDKKKSKGKESKKDESKVDVEVKEEIKESKEDSPIVEDKVIENGKPEKQNKELENKKDNDVEELTEKISTTQISDDKLDESKEKKLTHKEKKKMKKLQEYEKQMETMLKKGGQGHSELDSNFTVSQTQKTAGQLAAFENAVDIKVENFSISAKGNDLFVNANLLIAQGRHYGLVGPNGHGKTTLLRHVAQRAFDIPPNIDILYCEQEVVADDNTAVETVLAADVKRNDLLAECKKLEAAANSGDLEIQERLNEVYSELKAIGADSAEPRARRILAGLGFDKEMQDRATKNFSGGWRMRVSLARALYIEPTLLLLDEPTNHLDLNAVIWLDNYLQAWKKTLLIVSHDQSFLDNVCNEIIHLDNKKLYYYKGNYSMFKKMHVQKKREMIKEYEKQEKRIKELKSSGSSKKQAEKKQKEALTRKQEKNRTKIQKQEEDTTPTELLQRPKDYLVKFRFPEPPPLQPPVLGLHNTRFAYPGQKPLFVDTDFGIDMSSRVAIVGPNGVGKSTFLKLLTGDLSPDKGENRKNHRLRIGRFDQHSGEHLTAEETPSEYLMRLFDLPYEKARKQLGTFGLASHAHTIKMKDLSGGQKARVALAELCLNAPDVLILDEPTNNLDIESIDALAEAINEYTGGVIIVSHDERLIRETNCSLYVIEDQTINELEGDFDDYRKELLESLGEVINSPSIAANAAVAQ

>DvvABC-F_802

MPSDAKKREQQRKKDAAKARQAGKKNEKQKGEEQNDTLKTNGLSNGATNGSSELSAEEALCAKLEADARLNAEARACTGSLAVHPKSRDVKIDTFSITFHGCEMLQDALLELNCGRRYGLLGLNGSGKSTILAVLGNREVPIPDHIDIFHLTREMPASDKTALECVMEVDEERVRLEKLAEELVACEDDESQEQLMDIYERLDDMAADTAEARAANILHGLGFTREMQNKKTKDFSGGWRMRIALARALYVKPHLLLLDEPTNHLDLDACVWLEEELRNYKRILVLISHSQDFLNGVCTNIIHINKKRLKYYTGNYDAFVKTRMELLENQMKQYNWEQDQINHMKNYIARFGHGSAKLARQAQSKEKTLAKMVAQGLTEKVTSDKIVTFYFPSCGTIPPPVIMVQNVSFRYNDSTPLIYKNLEFGIDLDTRLALVGPNGAGKSTLLKLLYGDLTPTEGMIRKNSHLRIARYHQHLHELLDLDLSPLEYMMKEFPDIKEKEEMRKIIGRYGLTGRQQVCPIRQLSDGQRCRVVFAWLAWQVPHMLLLDEPTNHLDMETIDALADAINEFEGGMVLVSHDFRLISQVAEEIWVCEKGTVTKWQGDILSYKDHLKTKILKDAAKRK

>DvvABC-F_9935

MGTCSEYIKNVFPAIDEESKQYVEGVLLNGADDFEDSEEVYDAVGEVLKEISNDKSEDDIRNICNDLLCMLKPDKGEKATNGAMKVLNAPVHLGSMVDNTDTNIDDVKSIWLIQRDDSLKVDARKLEKAEAKLQEKLDKRTKEIKVIAPPKLQTATASQVTSKKDSKLEAKGTNRTQDIRIENFDVAYGDRVLLQGADLTLASGRRYGLVGRNGLGKSTLLRMISGSQLRIPSHISILHVEQEVVGDDTVALDSVLECDTVREELLKKEKEISAAINSGSVDPQLNSQLTEVYNQLQNIEADKAPARASIILNGLGFTSEMQQNATKTFSGGWRMRLALARALFSRPDLLLLDEPTNMLDIKAIIWLENYLQNWPTTLLVVSHDRNFLDTVPTDILHLHSQRIEAYRGNYEQFEKTKTEKLKNQQREYEAQMQQRQHVQEFIDRFRYNANRAALVQSKIKMLEKLPELKPIVKETEVVLRLPETEPLSPPILQLDEILFRYNSERVIFSNVNLGATMDSRICIVGDNGAGKTTLLKIIMGILSPTSGMRNVHRNLKFGYFSQHHVDQLDMNVNSVELLQQTYPGKPIEEYRRQLGSFGVSGDLALQTVSSLSGGQKSRVAFATMCMGRPNFLVLDEPTNHLDIETIEALGKALKKYTGGVILVSHDERLIRMVCSELWVCGNGSVKSVEGGFDEYRKIVEQELEAAAQSK

>DvvABC-G_9811

MIGTEEITRYIDDDKIKINPSHNYTAPSSIPLTENVNMTDTKSLAINYNNNYRFKSTQENMSSVFPKKTAVDLNFEDITFYSTSWSITKFKKETKKILHGVSGQFKSGELSVIMGPSGAGKSTLLNVLAGYITKGSTGTVKLNDVVRDQSPRYRKLSAYIPQDEELRMALTAKEAMTFAAHLKLGYRVSNDYKFKQIAGILKMLGLEECQHTLTAQLSGGQRKRLAVALELLSNPPILFLDEPTTGLDSLSCTQCVSLFKNLASEGRTVIATVHQPSALIFEMFDKLYALSEGKCIYDGRVSDVVPYFEKLNLKCPPYHNPADYLIEVSIGDHNANINKLADSVKSYQYKSMESGENDDLKGRDLFEEKKVGLYSDETKAVPAAIIMQFLLLYKRNLLIIKRCYGPSLNRVLAHIVIGLIFGYLYRNVGSAADTVLANYVYLYGTLLLTVYTGKMPVTLSFPLEMKILSREHFNRWYKLTPYLLSVILVEIPFQVICTWMYIAVSYWLTNQPLDFRLFLFVIFVTACSLCGQSMGYFIGATTPVKVAVFIAPVLACFLSVFGFCIRAIDTSTMFKPIFFISYFRAAFQSVVYSIYGFNREILVCPIEEEYCHYKDPHKFLSEMDILDVDLVSNFILIVIVWCVMHAVTYLTLWLRLNKR

>DvvABC-G_3712

MATEGLLKNKSDVKLQIVSNQPKKLVHLPERPKVDLAFSDLEYVVKQGSKEKKILKNVSGMLRSGELCAIMGPSGAGKSTLLNILTGYKTIGVKGHVLMNGSDRDLSQFRKLSAYIMQDNQLHANLRVDEAMAVAAALKIGAKSPKDREDIINEILDTLGLLDHKKTMTSGLSGGQKKRLSIALELVSNPPVMFFDEPTSGLDSSSCFQCISLLKTLAKGGRTIICTIHQPSARLFEMFDQLYTLADGQCVYQGSTTFLVPFLASLQLQCPSYHNPASYIIEVACGEYGDHTRTLVNAIENGKNDIRDASQIVGFKMSDGLNNAYQYAKDNLKTLISDELSKEGSTVNGNRKSNNVNEDTGIDSKDVEKANVDSALLNTSVVVKQPRYGNSEFQQFFIILKRALLFSRRDWTLMYLRLFAHILVGFLIGALYFKIGNDGSKVLSNLGFLFFNMLFLMYTSMTITILSFPLEMPVLLKEHFNRWYSLRSYYLAITISDMPFQTIFCILYVTIVYFMTSQPLDPARFGMFLLSSLLVSFVAQSVGLVVGAAMNVQNGVFLAPVMSVPFLLFSGFFVSFDAIPIYLRWITYLSYIRYGFEGTALATYGYNRPNLDCFAQYCHFKKALTTLEELDMDKSSYWFDILALVVIFFFLRVSAYLFLKWKLKSNH

>DvvABC-G_14042

MADDSKCIKINVPLDPEDTNQAVAYTKTVHAPPLLKETESLNNASVLSGSQTNIYNGSSPITISGAGALRKVPNSSPSNHKRPMIALTHLPKRPPVDITFTDLSYSVSEGRKRGYKTILKCISGKCKSGELTAIMGPSGAGKSTIMNILAGYKTSNLSGQVMINGKERNLRRFRKMSCYIMQDDCLSPHLTVKEAMMVSANLKLGKTVTLSEKKVVINEIIENLGLQTCIDINSSNLSGGQRKRLSIGLELVNNPPVMFFDEPTSGLDSSSCFQCLCLLKSLARGGRTIICTIHQPSARLFEMFDHLYMMAEGQCIYRGPVLGLVPFLSSMGLNCPSYHNPADYVMEVACGEHGDYVQKLVVAVNAGRCTKFATPDHRSSKIVSNDIAKEANGKNSSGDVISVPNGSVKPTTPTTPVTCTTSLLDSSENLSPTEKNGFSTTGLQQFTILLKRSMYMILMDKTLTRMRLVSHFVIGCLIGLIYYDIGQDAAKVTSNAGCLFFCVMFMMYTAMMPTILTFPLEMSVTVREHLNYWYSLKAYYMAKTLADIPFQVVMTLCYIIGVYFITSQPLDVTRFGMILLVTVLTALVSQSFGLLIGAAFNIEGGVFLGPISTIPMVLFSGFFTNLNDIPFYLRWLPYLSYLKYGFEACMIAIYGLDRPKLTCNIEYCHFKYPKKFLEQMSMKDDMVSYFIDVGVLGGLFIFLRVIAYFMLRIKLMQNR

>DvvABC-G_10897

MEAVLHESTFVGQSKDAAAVVKSNGVNGDSVNFSVKVNLNQEKTLICDDGTNGKMCQNTVDLEFQNISFRATQGNIFTKRTTKEILHQVGGRFQPGQLIAIMGPSGAGKSTLLDVLSGYRIRGVTGSVYVNGQPRSLKEFRKTSCYITQDDRLQPLLTVDENMWVAADLKLPSSVSNSNKRSIINNILKTLNLENTKKVRAAGLSGGQKKRLSIALELVNNPMVMFLDEPTTGLDSSSCSTCITLLKKLTMQGKTIICTIHQPSATLFAMFDQVYVVGNGYCLYQGSTNKLVPFLQDCGFPCPQYHNPADYVIELACIEHGPEKPEYMRERIQNGQSYQYFNEPEKMKSTICNYPGPKPIKRVvSSDaSSFPTSQFHQLGVLMRRGFIKAYRDKMLTYLRIGTNIIVGLMLGSLYWKAGSDGSKVLDNFNLLFAILLHHMMSTMMLTILTFPQEMSILIKEHFNRWYSLKMYYTSVTIVDIPVSVIGCFLFSLIIYYMTDQPNDKARFLIFFVTCMLVVLVAQGVGLTIGAYFDVVNGTFVGPTLMVPMMMFSGFGVRLRDLPTIMYYGSFTSYLRYGLEGVVQAVYGMDRGILQCPEDKFCFYKYPKTFLEIVDVRSDQFDNDIIALLLFLFVLRIAAYVVLRYKLASVR

>Dvv_ABC-G_22358

MELHSRNTSLRHLPKRPPVDIEFHDVSYTVPQGRKGSKLILRSVNGSFQAGQLTAIMGPSGAGKSTLLNILAGYKTQGATGQILINGATRNLKQFRKMSRYIMQEDMIQPLLTVEEAMMIAANLKLGNTLSLSDKSSAIDEILSLLRLDKAKRTGTSRLSGGERKRLSIALELLNNPPVLFLDEPTTGLDDLSCSQCISLLKKIAEGGRTVICSIHTPSAKIFSQFDNVYILSDGQCVYQGYGPEVVSYLSKVGIECPKTYNPADFIIEVCCNEYGNFQERMVSVIDNGRNIYRTNGNLPQLQEVELANDSLENRSSISSISIREEVYADLSSSDFNHESSWFSQFWILTTRLWMQMWRDKTYLIMRTVLYIILALLIGSLYYKMGQDGSKTIFNFGFYYCCIIFFMYIPMMPILLQFPQEIQLVKREHFNKWYRLSAYFSALSFSTVPVQLCLGVVYVSCVYLLTDQPLEFRRMSMFFFICILTSVISESLGLLISAQLKVVNAVFMGPVSAVPFMLLAVYGFGSGYDTIPSIIKFFMHFSYLRYSLEGLIHAMLKDREKLSCPDTEEYCIYTDLNLFVRDMGMENTIYWVDVlVLIFILILFRGGSYYLLRQRLTPNKTFRALQYIGRLVKSQFGLAR

>DvvABC-G_23081

MELQQFASNNLRISDKILDVEFQDISFVTKEKNGYKKIIDGVSGKFHCGHLTAIMGPSGAGKTSLLNILTGYQVTGTTGTIKCNSSSRKQKGVLQYKKESCYILQDDSLPNLFTVEECMMIASKLKIANMAKKAREFLINEILTNLSLLKAKNTRCQSLSGGQKKRLSIALELVDNPPILFLDEPTTGLDSASTTQCVDLLKKLANGGRIVICTIHQPNTQTYEMFDQVYMLAKGRCVYQGPSTNTVPFLASVGLHCPQYHNPADYIMEVVSGEYGDHIDQLAVAAQDKKWQNIPTIKLSDTPAIDSKDNNIIYSDENVTLSKSPSEWKRFFILLQRSSVQLYRDWTISQLKLVLHLLVGLFLGITFQNCGRDATKVISNLGFLQVGIVYLAYTSMMPAVLKFPTELVILKKESFNNWYKLTTYYAAFLVFDIPQQMLFSTVYCIGCYFVSDQPLEVdRFFSVLFVLVLASLSSSGFGLILGTITNPINGVFFGAVGLCFFISVGGFFIMFTHMSNVMYLFSYISYISFSVEGVMQAIYGYGRGQLHCPEEAEFCQYVSSEVLLEDIGMSKPNYWIDIIYLTCTFLTFRTIAFVTLKRKLANP

>DvvABC-G_13051

MSEASGSAMEVLLPKFKPLTQLNTIAKKPPIDVEFCDLTYSISDSSYKGGWRQLLKSINGKFRSGELTAILGPSGAGKSTLLNILAGYMTAGVKGSVKINGKPRDMRIFTKLSSYIMQEDLVQPRLSVRESMMVAANLKLSASIGHTQKVAVVHEVIQLLGLEKCYDTKTEYLSGGQRKRLSVALELVNNPPVIFLDEPTTGLDNVSIKQCIDLLKKITRLERTVICTIHQPPASLFQIFDQVYIMANGYCVYNGSPNQLVPFMSSVNCVCPETSTPADFIIEVIQTNQDNIPILQNQIQNGKINMKDKKLKPLQSHKTLGIYEIYQETTQTGMHIHDIEYPTSFWTQFTVLLCRMALQMKRNKSMWIIQFFHHVISATLVGGIFYQIGNEASQVLPIFKYCVTINVFFVYTHVMVPVLLFPIEVKLLKREYFNRWFSLKPYFLASTIVNIPMLVGYGMIFISIVFFMTGQPIEWQRFLMFTMIAINVGFCSQGLGYAIGSNCGILSGSVVAPCWLAILLALSVYGMGYKDGIEPVMKAFMSLSYVRYGLVGISSTLLNDRAEMDCNDIYCHYKNPEKLLEDMGMSQNVPLHQFAYILGYTVLFRIIAYISLKYRMTSELRNKLVYYAAKIVKQKET

>DvvABC-G_38769

MGENTVCDILGTVDPPQESNKIHDEDFRLHSLELPENQVNDPASLINVGFRHISYSVYDGILFSRTKRKLLDDITGNFPGGQLTAIMGPSGAGKTCLMNILSGYATTGILGEVLVNDEPRNNVAFRKQSCYIMQNDDLQPLLTVLESMRVAADLMLTASKTDKDKKIASILKSVTLWEIKHTRTDALSGGQKKRLSVALELLRDPQVMFFDEPTSGLDSLNSIRLVKLLKEMSESGKTIICTIHQPSATIFKLFDHLYVVSSGKCIYQGSPNNLLPYFEDFDLICPTQHNPADFLLEVASGDYGDFTHALSTKSNNGINESYAKLSASTMKLNSIKEPNIGEDFLAATKRTIVNEIEYTWDECGLNSYPTSTINQFVVLTKRSFLMLSRDRTLTYFRLGTHSAIALFLGILYFGVGLDAANINDNFSFMFFTVMFLMMTAFNCVVTTFPSELPIIIKEHFNKWYAIRSYYAAVSISDITVQILATVLYATITYLCTQQPMEWSRVSSFLFICILVSVISQSWALVVGSCLSVTNGVVIGPFFLLPFIMFSGYFVQLRDCPHQLKWMFDISFPRYALEGLVLTIFGYDRGKLPCESKDFCLYVYPEQFIKDKDMENASYTIG

>DvvABC-G DvvW

MAVYNRGGREEESSASESLSLAARLGLRVVRDYGTDFRSVGRVIPIEERMKFTWNDINVFAMTKTIKNKFCCFGFMSHGYDEHILKSVDGVACSGELLAILGSSGSGKTTLLNALTYLPMDGMVVSGMRCINGVPVDGQRLRNVSAFVQQDDCFITTLTVREHLVFQALVRMERGVSYNQRLIRVDEVLSELLLKKCENTIIGSTDKGGISGGERKRLTFASEMLTNPHIMFCDEPTSGLDSFMALQVVQALKAMAQNGRTVICTIHQPSSELYVLFDKIMLMTEGITCFLGTREDADGFFITMGAGCPRNYNPADYFVKLLSVIPDREESCRQAIALIADKFHNSNLGRRLAADSAYIRAQEEVDNAVWLSNNLRPYRNSCWAQFRAVLWRSWISMLKDPLIIKVRFLQTVITSLLIGMIYYGQELNEEGVMNINGVLFIFLTNMTFQNVYAVIHVFTAELPVFLREHRSGMYRTDVYFISKTLAELPFFIIIPVAFTTVCYYLIGLNGTLTKYFITCGIVILVANAALSYGYLVSCISRNTSMALTLGAPLVIPFLLFGGYFMNLGSLPHYLKWLSYFSWFRFGNEALMINQWENITDIDCSTNSTICPKNGHVILETYNFAEENFTVDIIALCMLILGFRFFAYLALLNKTCSC

>DvvABC-G_49457

MMRSRTPSQGGGSQGFEMERKYSVPSNPESRAFSGGTTSEDLHAWSIYRQNLNSDFTDSALGSTDKSPLPYGNFQLRDTTVQSILSHPRYGPKSALGSNMYTYLKFGLPRVFPPNHNGSNRSGTPQHFRRNSSTRPHNIRRSKAGSHGPRDGSSGYDSSDNETSHNYKQNRKYRSDPDFRMQNVYHAEQTSPGIPLAAMHQGDIRHSNSQWNRNKSISEANLLALGYNRSCHNSERHLIDPRRNSVADYGHHNHDVVDHSDMGHMGRPMSKTESHFSVPHSRRGPPSILRTDYLNQDDESGTTFMFPHLQAHGLGIFPSSQSCTKSRQHLLLNEISFEIRGGEIMAIMTTSEEEGTALLDIIAGFSSPALGTIFLNGHSVRAHTLKSRVAYVQNDLNLCKDMTVVQTLRLHYDLKKPTEKLGYLKIESMDRINVLIDDLGLEQVRNTKVSMMTISERRRLNVACHLILDTDIVLLDQPTKSMDIFDTFFLVEYLKQWASGGAGSTLGRIVILTMHPPTYEIFTMLSRILLVSAGRTMYSGRRRDMLPYFALVEYPCPAFKNPSDYYLDLVTLDDLAAEAMLESSQRIEQLAEIFRQKQEPLSDPGPPSSLPLTVRNCNCFVAAFALFTKSMIYTQPATFLSWLTVIVLSASLSLILGAIFWDIPSTDHQLILNDRYGYHYSVMCIVHWPLLLAMTVNEVRRNRKVIERDIKDGLYGRVIYIITKSIINIFPSLFVWLIYVVPSYSMTGLYMQHLNNYDGFYIYIGVMLLYLSCIQIFLMAFIYTVPLSNTATIFCGTVLSAFFLSAGYSLHLKDIPMYLQWIEKISPSEWLIPYLLNRELSTEAIQSLQGTITTLCRNKQIQHQDIIVQLPCPPPNGTNSLKSFGYLKSDNLTFDYGNPVIAMGVFYCIFFVISCFMFALNLCRSRRRRRQDTKNDANKP

>DvvABC-G_36869

MLSGGYILELCNVFYSGQVEKRSVLQRMVGNIKTAVILKDVSMLVHSGEVLAVLGSKGSGKKALLDVISRRAQGPIRGQIYLNNHPVSMCLFQQKCAYVTHKCDFIPGLNVEQTLYYTPTKFTGYLKMSKVKQVIADLALSQVAKKCVEDLTKSEYRRLMIGVQLIKNPVVLLLDEPTWDLDPLNTYLIISILSNAAKKYGTAIILTMEKPRSDVFPFLDRVLYLCLGDVVYTGGTKQMLEYFNVIGFPCPQLENPLMYYLCLSTVDRRSRERFVESNYQIAALVEKFKNEGVIFQKSPSMISPNPNHEHGQHEKVPFMHGRPGRFSTGWTIYVRLLAATVSFKRAGLRQTFLRTFALPLYFFLMWLFYREMKDWQHTFISRNGLILNCLCCVYFVGIINTILIYPIYRTRYYQDTQEGLYGGTLFLLTYNLVSLPFSFLSTVLSAVIIFPLIISFDNPVDFVYFTLILWACYIFAEQQTMAILMIVKDYMKAAIFSIYLTVVCITLGSGILRSMKGLPEWLYYATYGTQARYAAAYLNRKVFTHSALSKALPFDLLHNCTQISFETSILNGANNAYCRYASGQAFLTERYSRDPTEVIFSGVLEEDFNVGLTCGFALGMIVFNLFLYLIPLPSFIKAKFRE

>DvvABC-G_79525*

MLDVEETPLNLFQSHGSSGSYNEFDIYASASLAKKRQRTYSRWSPIEEGVTLVWENLSVYSSTTKNGQMQHKQIINGVTGAVKAGSLVAIMGSSGAGKSTLMTALGYRTEGSILTEGNILINGRQIGDYMKYLSGFMHQEDMLLSYLTVREHMNIMANLKLDRRLSGNDKKQLIYDILRQLGLMKCIDLKIGGIDQAKSLSGGEKKRLAFATELLTDPPLLFCDEPTTGLDSYSAQKLVVIMNQMAITGKTILCTIHQPSSDIFAMFSQLILVADGRIAYMGSTNNALDFFERMGYVCPTSYNPADFYIKTLSTTPGYEDNCRQTVKRICDQFAVSDEAKEVEIVVQYELHMGRVATQRKFELRQNFKEIRWLSKLFWLTYRWILEIYRNPSLEAMKIAQRMLIGFIVGFCYLGTDALTQNGVQSVTGIIFMFVSENTFNPMYSVLHQFP

>DvvABC-H_20789

MHDDYAVFVDRVVKKYGQKEVLTGICMKVERGSIYGLLGASGCGKTTLLSSIVGRKKIDGGEIWVLGGKPGEAGSGVPGPRVGYMPQDIALVGEFTVKDAIYYFGRIFSMEDSLIAKRYRNLHTLLELPPDDRYLKNCSGGQQRRVSLAASLVHKPELLIMDEPTVGVDPVLRDRIWKHLVDITKKDNTSVIITTHYIEECRQANKIGLMREGKLLAEESPTRLLTLFNSETLEDVFLLLSKRQEEGRLQELTSHRVVDDQNNSMLANDTTGSTTSVATSVSTFEIGHGSTDILAKKKILKARNALNKSRMKALFDKNLKQFYRNITGIIFLMTFPILQVGVFMGAVGGDIRSIPLGIVNDEAMSVTCPGFSFNGTATATDDRACQLRNISCRFLSYLDHPMIEKVHFETLEDAKDAVLHGKIVGAMYMSSNFTSFLEERIDKGKDIEKDILSLSEIKVWMDMSNRQIGATLKYKLIDLYTKFQNSLFDDCDFVPGFGDLPVNINFIYGDGDEPYTVFMIPGSLITIMFFMGAIMTSQIIITDRHDGVWDRSIVAGVTSLEITITHLVLQASICIIQTAELLVVVYLIYQQEYSGSLWLMYVMVYLQGICGMAYGFWVSVISTDHSMANTVLTGIFLPMMMLSGLMWPTEGMPPALRIFSRCLPFTMAIESLRNVSKRGWSIDNFQVYSGMGVGFMWTVFFGVLSVYLIKKKR

>DvvABC-H_5118

MFSWNNLVNVLGRLLGIYRRALSIEGTTMGDRVEPQVPGPDLVGVNVERPPLVHQQSTVWNRRQNAVSVRHAYKHYGSKKKPNHVLSDLNMTVGKGTIYGLLGASGCGKTTLLSCIVGRRRLNTGEIWVLGGKPGTKGSGVPGKRVGYMPQEIALYGEFTIKETMMYFGWIFGMESKEIYERLQFLLNFLDLPSQNRMVKNLSGGQQRRVSFAVALMHDPELLILDEPTVGVDPLLRQSIWNHLVQITKDGNKTVIITTHYIEEARQAHTIGLMRSGKLLAEESPQVLLQMYQCTSLEDVFLKLSRKQVQVGAAANDQNMANNISLATLNWSKKESVSVTEESGVVGLNFHQSKEVLVPDSNGHLDMGKPPSSATHGIKEACDDCSSCSDFTTTGKLRALLQKNFLRMWRNVGVMLFIFALPVMQVILFCLAIGGDPKGLKLAIVNHEKNFTNLTYQECEYEKGCKFGNLSCRYVDTINTTTIIKQYYATPEEAKEAVRTGNAWGALYFTENFTDALVARMALGKDADEETLDQSEVRVWLDMSNQQIGIILQRDLQLAFQNFTKDIFRDCEYNEELAEIPISFKEPIYGSNQPSFTDFVAPGVILTIVFFLAVALTSSALIIERMEGLLDRSWVAGVTPSEILFSHVITQFVVMCGQTTLVLIFMIIVFQVECKGDIFLVVVITILQGLCGMCFGFVISAICELERNAIQLALGSFYPTLLLSGVIWPIEGMPTVLRYISTFLPLTLATTSLRSMMTRGWSIGEPDVYYGFIATIVWIVLFLTISLLVLRSKRG

>DvvABC-H_18290

MNTMNNHLALYVKDVEKSFGSNKVLDKLCLNVQKGTIYGLLGSSGCGKTTLLRCIIGRSNVNKGDIYVLGEKQDGSVVLADRVGYMPQDISLVGELSARDSIYFFGRIYNLKDDIIKSRCDELIELLDLPTDNRCVRDCSGGEQRRVSFAVTLVHKPELLILDEPTVGTDSILRNRIWKYLTKITEEDKTTVIITTHYIEETRQADKIALMRKGKLLTEEHPETLLERYGCDLLEEVFVILSSKQEETEQTSGVDNLAFQSSQTGINESTERTNAALDVISEAPQQTRTERKKQSIFEMIRFKSLLFKNYKKLKSNLISTIFLVVFPALQVLFFLTAVGRETKGNPFGIVNNERSQEFCKTFPRNESAVPYDKFSCHINSASCLFEDYLDVPLFYMIRFDSKYEALSALESGKIRGFLHMHQNLSELIEARVNYGVDKYDLPDEEMQVYMDMTSIQVGGIIKERIADKYQLFHEKIYDDCRYLSKAVQIPLRVEESFYGNKNEEYVIFITPGIITSMIFFMGVLMTCVIILDEKCEGIWDRSIVAGVTALELSLSHLLYQNVFMIIISLDFLLFVFVIFGQPYIGSLWLIFALLYLQGLAGIMLGFAISVLSDNHVTANTIVTGIFNPLVLVSGILWPAEALPKYIYFVIVWLPLAIPTSALRNVIRKGWTLTNLHTLHSIGILSFWILFLLALNIYLTKTKR

>DvvABC-H_11818

MERPSDIELRRRQFKTQASTLNSRRQQAVCVRRAVKKYGTKSNPYVILDNLNMTVPKGCIYGLLGASGCGKTTLLNCIVGRKRLNSGELWVLGGTPGSRGSGVPGPRVGYMPQETALNGEFTIRETLKYFGWISRMTTDEVEAKVDFFINFLMLPDADRQVKTLSGGQQRRVSLAATLLHEPELLILDEPTVGVDPLLRTNIWNHLVEITKYGRTTVIITTHYIDETRQAHLIGLMRGGYFLAEESPERLITQFGVDSLEDVFLKLSVMQNMGKRRRSSIAKSVVETITVPELGGAVNPAAVLDDDLGEISGEFGDSVSVTSRTGRRVSIAPEPTAEHIIPELPPDEEPEVSFKDYFKFVQSHHMRALIWKNFLWMWRNIPMMMFIIGLPISQTILFCWTIGHDPQGIKVSVVNNEINFPDETCHQQPLNCNGTKISCNYLNEVAKSYSITWEFMKTEEEARHRVERGKSWGVVVVPHNFTDALWSRIENSRDTPPEDIMASTISVYEDKSNENIATFLTRDMLYGFLTFISEFVKSCGYNEKSVGVPIRYNEPIYGYQDPDFTDFAAPGVVLTIIFFMAVALTSGSMIMERNEGILERSLVNGISGTELLFSQIITQFVVMLGQSIAVLLVAFILFGLTQRGDWTTVTTLTVLAGICGMCFGFVVACSCENERSATYLAMGSFLPIVMLCGIIWPIEAMHKYISWMSVVLPLTQATESLRCILARGWPISNPTVYNGFISLTIWIFIFLTISILLLKFKKG
