# Supplementary material for: Structural and functional insights into the Diabrotica virgifera virgifera ATP-binding cassette transporter gene family
Source: BMC Genomics. 2019 Nov 27;20:899. doi: 10.1186/s12864-019-6218-8 (PMC6882327; doi:10.1186/s12864-019-6218-8)
Supplement: Supplementary file 9 — Additional file 9: Table S3. Primer sequences used for dsRNA synthesis and RT-PCR analysis. [file 12864_2019_6218_MOESM9_ESM.docx]

**Additional File 9: Table S3: Primer sequences used for dsRNA synthesis and RT-PCR analysis.**

| **Gene** | **Sequences** | **Purpose** |
| --- | --- | --- |
| *DvvABCA_50718 -F* | TAATACGACTCACTATAGGGGAGATGGTGTGACCTGGAGC | dsRNA synthesis |
| *DvvABCA_50718 -R* | TAATACGACTCACTATAGGGACGGTGAGCTCGTCGAAAAT | dsRNA synthesis |
| *DvvABCA_50718 -F3* | TGATGGACGCGTCTAATCC | RT-PCR |
| *DvvABCA_50718 -R3* | CCATAGCTCCTGATTCCAAG | RT-PCR |
| *DvvABCB_39715-F* | TAATACGACTCACTATAGGGGGATGCGTTGGTGTGTATGC | dsRNA synthesis |
| *DvvABCB_39715-R* | TAATACGACTCACTATAGGGAGGATTCGCCCTTTCGATGG | dsRNA synthesis |
| *DvvABCB_39715-F3* | TGGTCGGCAAATGGTAGGAG | RT-PCR |
| *DvvABCB_19147-R3* | CACCGTAATGGAGGTTATGCC | RT-PCR |
| *DvvABCE_2830-F* | TAATACGACTCACTATAGGGCCTGGGCAACCTTCAACTCA | dsRNA synthesis |
| *DvvABCE_2830-R* | TAATACGACTCACTATAGGGTTCCTGGACGGTTTTGTCCC | dsRNA synthesis |
| *DvvABCE_2830-F3* | ACACGATCGGCCAAGTAGGTC | RT-PCR |
| *DvvABCE_2830-R3* | GGATCCTCCAGATTGGACAGA | RT-PCR |
| *DvvABCF_2701-F* | TAATACGACTCACTATAGGGGCCGCTGCTAATAGTGGAGA | dsRNA synthesis |
| *DvvABCF_2701-R* | TAATACGACTCACTATAGGGCTGGTCGTGGGAAACGATCA | dsRNA synthesis |
| *DvvABCF_2701-F3* | CAGGTACGTGATGAATCTCCAG | RT-PCR |
| *DvvABCF_2701-R3* | TGCCAAGGCCACTCTTGCTT | RT-PCR |
| *DvvABCG_14042-F* | TAATACGACTCACTATAGGGACGGTATGTCCGCCAATGTT | dsRNA synthesis |
| *DvvABCG_14042-R* | TAATACGACTCACTATAGGGGGAGGTAGCATGTGGAGAGC | dsRNA synthesis |
| *DvvABCG_14042-F3* | GCACGTCAGCTTCGGTCTAT | RT-PCR |
| *DvvABCG_14042-R3* | CAGGACGACTGTCTTTCCCC | RT-PCR |
| *DvvABCG_3712-F* | TAATACGACTCACTATAGGGCGACCTCTGGCTTGGATTCT | dsRNA synthesis |
| *DvvABCG_3712-R* | TAATACGACTCACTATAGGGCCATTCACCAACCTAGCTCC | dsRNA synthesis |
| *DvvABCG_3712-F3* | CGTAGGCAGTATCGAGATGACG | RT-PCR |
| *DvvABCG_3712-R3* | CAACCAACAGCACGATCTGTC | RT-PCR |
| *Dvvw-F* | TAATACGACTCACTATAGGGTGCTCTCGAAGGAACACTGG | dsRNA synthesis |
| *Dvvw-R* | TAATACGACTCACTATAGGGTCGACAACGCTGTATGGCTT | dsRNA synthesis |
| *Dvvw-F3* | CGTTCGCCTCAGAAATGCTG | RT-PCR |
| *Dvvw-R3* | CCATCAGCATCCTCTCTGGTT | RT-PCR |
| *DvvABCH_5118-F* | TAATACGACTCACTATAGGGGTGTTACACCGGCTACCCAA | dsRNA synthesis |
| *DvvABCH_5118-R* | TAATACGACTCACTATAGGGAAAGAGGCGGTTAGAACGGG | dsRNA synthesis |
| *DvvABCH_5118-F3* | CCCCATTTTACGGTACTTGCTAGG | RT-PCR |
| *DvvABCH_5118-R3* | AGCGGTAAGCTCCTGGCAGAA | RT-PCR |
| *DvvRPS6-F* | TTGAAGAAGAAGAGGTGC | RT-PCR |
| *DvvRPS6-R* | ACCTACGCCTCTTCAACATC | RT-PCR |

Underlined sequence corresponds to T7 promoter
